# The Versatile Structural World of Methanedi‐ and Trisulfonic Acid and Their Salts

**DOI:** 10.1002/open.70247

**Published:** 2026-06-30

**Authors:** Katrin Eppers, Alisha Mertens, Jan Chrubasik, Mathias S. Wickleder, Jan Langwald

**Affiliations:** ^1^ Institute of Inorganic and Materials Chemistry University of Cologne Cologne Germany

**Keywords:** methanesulfonates, organic‐inorganic hybrid composites, solid‐state‐structures, structure elucidation

## Abstract

The syntheses and structural elucidations of 16 novel compounds, derived from methane(poly‐)sulfonic acids are presented. The anhydrous acids, that is, methanedisulfonic acid (MDSA—(H_2_C(SO_3_H)_2_, **1**) and methanetrisulfonic acid (MTSA), the latter as two polymorphic structures (HC(SO_3_H)_3_‐I (*P*3*c*1, **2**) and HC(SO_3_H)_3_‐II (*P*2_1_/*n*, **3**)) have been crystallized and structurally elucidated. Methanedisulfonic acid was subsequently used to prepare four group I methanedisulfonates (AM_2_[(H_2_C(SO_3_)_2_], AM = Li (**6)**, K (**7**)·H_2_O, Rb (**8**) and Cs (**10**)) as well as the strontium dihydrate Sr[H_2_C(SO_3_)_2_](H_2_O)_2_ (**9**) and the ternary salt BaK_2_[(H_2_C(SO_3_)_2_]_2_ (**11**). The known trisulfonate K_3_[HC(SO_3_)_3_](H_2_O) was used for the preparation of the oxonium salt [H_3_O]_3_[HC(SO_3_)_3_] (**4**), which was isolated as a new polymorph (*R*3*c*). Subsequent neutralization led to isolation of the compounds AM_3_[HC(SO_3_)_3_] (AM = Li (**12**)·(H_2_O)_4_, K (**13**), Rb (**14**)·H_2_O, as well as the two ternary salts Rb_3_Ag_3_[HC(SO_3_)_3_]_2_ (**15**) and Rb_5_Ag[HC(SO_3_)_3_]_2_(H_2_O)_2_ (**16**). For a structural comparison between a mono‐, di‐, and trisulfonate, we also prepared the still elusive rubidium methanesulfonate, which crystallizes as the hydrate Rb[H_3_C(SO_3_)](H_2_O) (**5**). Different synthetic approaches, as well as an in‐depth structural analysis of all compounds, are presented. Furthermore, compound **8** and the oxonium salt of compound **1** were investigated concerning their thermal decomposition.

## Graphical abstract

The solid‐state structures of the CH_4_‐derived polysulfonic acids methanedisulfonic acid (MDSA) and methanetrisulfonic acid (MTSA) are presented for the first time. Additionally, a broad overview over possible MDS and MTS salts, containing mostly group I cations, is given and their solid‐state structures are analyzed, showing the structural versatility of these simple organosulfonic acids.

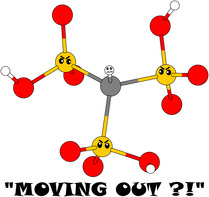

## 1 Introduction

Methanesulfonic acid (MSA) is the simplest organosulfonic acid and was first reliably reported by *Billeter* in 1905 [1]. With a pK_a_ value of –1.9 (0.1 M aq.), MSA can be classified as a strong acid [2]. After the development of optimized preparation pathways [3, 4] and industrialization of the production, MSA became prominent as a metal plating agent, an electrolyte, as a *Brønsted* acid catalyst, and for metal descaling [5, 8]. Recently, the groups of *Ess* and *Periana* could show that MSA can be directly received using CH_4_ and oleum with a Bi^V^ catalyst [9]. Besides MSA, the fluorinated derivative, F_3_CSO_3_H (triflic acid, TfOH) [10], is probably the most well‐known organosulfonic acid. It is still synthesized by electrofluorination [11]. Although TfOH and its derivatives have vast applications within research and industry [12, 15], their environmental impact and potential risks gain growing importance [16, 17]. The search for suitable alternatives continuous and the brominated and chlorinated analogs are already accessible and might serve as potential substitutions [18, 21]. Concerning their structural chemistry, organosulfonic acids are quite different. While many solvent‐free triflate salts are either unknown or were solved from powder X‐ray diffraction (PXRD) data, due to their tendency to form disordered phases [22, 24], a broad selection of methanesulfonates was crystallographically elucidated until today [25, 28]. Surprisingly, the solid‐state structure of the respective acid was unknown until very recently, when we were able to crystallize it by using OHCD techniques directly on the diffractometer [19].

With growing research interest in MSA and its salts, for example, for their optical properties [26, 29, 30], the attention has also increasingly shifted toward further sulfonation, leading to methane (poly‐)sulfonic acids and their derivatives. These compounds can serve as “small” polydentate anions for the complexation of high‐valent cations, act as electrolytes in emerging battery systems, and function as linkers in the construction of macromolecular frameworks [31, 35]. However, compared to the methanesulfonates, there are only a few reports on the structures of the higher homologs’ salts, that is, methanedisulfonic acid (MDSA) [36, 39], methanetrisulfonic acid (MTSA) [40, 41], and methanetetrasulfonic acid. The solid‐state structures of the neat acids remain unknown; only the oxonium salts were structurally characterized [38, 40]. This is especially peculiar since the respective acids are, either as aqueous solutions or as salts, already widely used [42]. Nonetheless, certain uses, like optical materials for the MSA salts, require insight into the solid‐state behavior of these materials.

**SCHEME 1:**
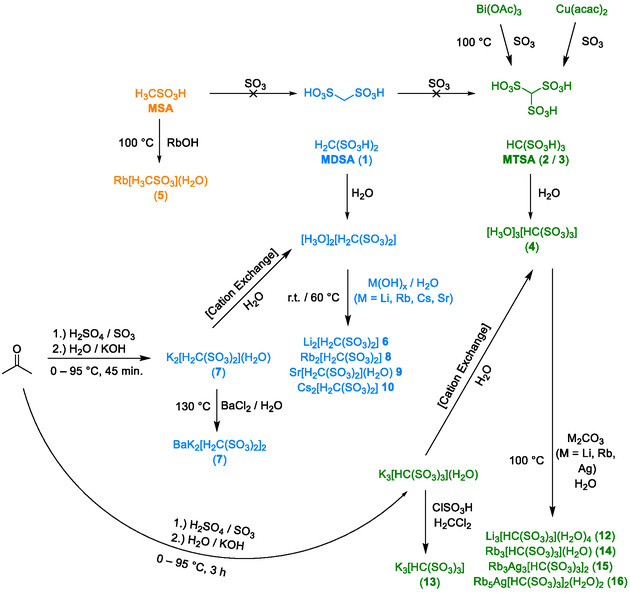
Overview of the novel compounds **1**–**16**, the reaction pathways toward them, and for their interconversion. Methane(mono)sulfonic acid, methanedisulfonic acid, and methanetrisulfonic acid and their respective salts are colored orange, blue, and green, respectively.

During our research concerning substituted sulfuric acids and reactions with H_2_SO_4_/SO_3_ systems, we were able to prepare several novel organosulfonates or organic sulfates in the past [43, 45]. The smallest scaffold for an organosulfonic acid is CH_4_, and accordingly, we also became interested in the synthesis of methanepolysulfonic acids. In the first part of the article, we present for the first time the solid‐state structures of methanedisulfonic acid (MDSA, **1**) and two modifications of methanetrisulfonic acid (MTSA, **1** and **2**). Subsequently, the anions [CH_3_(SO_3_)]^–^, [CH_2_(SO_3_)_2_]^2–^, and [CH(SO_3_)_3_]^3–^ will be structurally compared on the example of their rubidium salts. Additionally, a number of further salts of MDSA and MTSA were presented, in some cases with additional information on thermal decomposition and vibrational spectroscopy. With this quite large collection of compounds and data, we also intend to provoke further investigations on these fundamental sulfonates. All the new compounds and the respective reactions used for their preparation are summarized in Scheme 1. Detailed synthetic procedures and further information on the analytics can be found in the *Experimental Section*. Detailed crystallographic information can be found in the Supporting Information.

## 2 Results and Discussion

### 2.1 The Free Acids and Oxonium Salts

**FIGURE 1:**
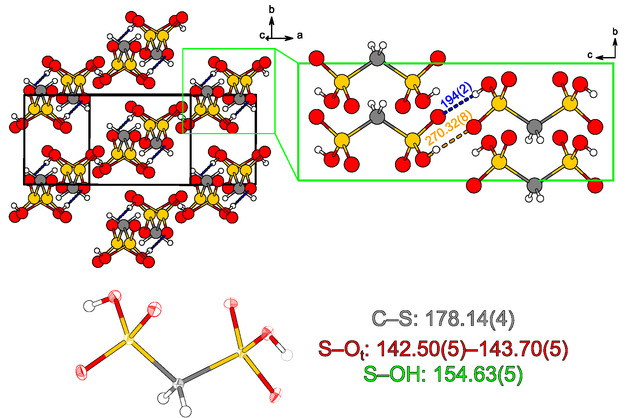
Filled unit cell of **1** (top left) and hydrogen‐bonding within the solid‐state structure, viewed along the crystallographic *a*‐axis (top right). O─H and O─O distances are shown as blue and orange dotted lines, respectively. The thermal ellipsoid plot (50% probability) of the asymmetric unit is given at the bottom along with the bond lengths. All interatomic distances and bond lengths are given in [pm].

We isolated single crystals of free MDSA (**1**) from a reaction of commercially available MDSA within a mixture of TfOH and Tf_2_O, that is, a superacidic environment, under solvothermal conditions. Thus, the absence of the solid‐state structure of **1** arises from the unsuccessful crystallization conditions applied so far. In contrast, we received crystals of free MTSA (**2**/**3**) from reactions with (heavy) metal acyl compounds, which is in line with modern synthetic pathways, although the respective mechanism of formation remains elusive [42, 46]. Our own work on acetonedisulfonic acid (ADSA) and its salts, which can be prepared from the reaction of oleum/acetone as well, suggests that the formation of ADSA is an intermediate toward MTSA. This would also fit the literature reports on sulfuric acid catalyzed enolizations [47, 49]. MDSA crystallizes in the monoclinic space group *C*2/*c* (no. 15) with four formula units per unit cell. The packing in the solid state is governed by hydrogen bonding between the monomers, as shown in Figure 1.

The donor–acceptor distance in **1** is considerably elongated compared to H_3_CSO_3_H (261.45(9) pm) [19], but the O–H–O angle of 172(2)° shows a high directionality, suggesting that it can still be considered a strong hydrogen bond [50, 51]. **1** shows a remarkable similarity to ADSA [44]. As for ADSA, every terminal sulfonic acid group in **1** provides a donor and acceptor functionality in proximity to each other, leading to the development of a strand‐like, 1‐D twisting chain along the crystallographic *c*‐axis (graph set notation *R*_2_^2^(8)) [52, 53]. The C–S bond length in **1** is significantly elongated compared to H_3_CSO_3_H (173.8(1) pm), as expected for the strong electron withdrawal introduced by the additional sulfonate unit, whereby the S–O_t_ (t = terminal) and the S–OH bond lengths are in excellent agreement [19]. The reported oxonium salt of **1**, [H_3_O]_2_[H_2_C(SO_3_)_2_], shows slightly differing packing motifs due to the oxonium ions ‘breaking’ the formation of the *R*_2_^2^(8) rings [38]. The thermal decomposition of [H_3_O]_2_[H_2_C(SO_3_)_2_] shows the release of water in a broad range between 390 and 525 K, followed by decomposition of the dehydrated acid between 535 and 575 K (see Figure S3). The fragments of the decomposition are, according to mass spectrometry, SO_3_ (and its fragments SO_2_ and SO) as well as CH_4_. Thus, the decomposition of the neat acid follows most likely the reaction H_2_C(SO_3_H)_2_ → CH_4_ + 2 SO_3_ (and further fragmentation of SO_3_).

**FIGURE 2:**
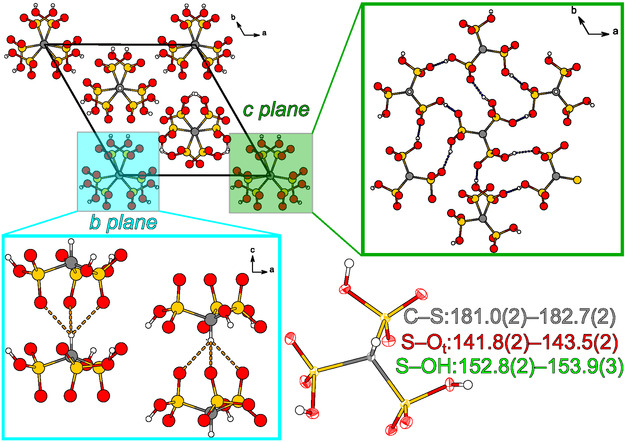
Solid‐state structure of (HC(SO_3_H)_3_‐I (*P*3*c*1, **2**). Packed unit cell (top left), depiction of the hydrogen bonding along the direction of the *a*‐axis (top right), and the unconventional H‐bonding along the *c*‐axis (bottom left). The thermal ellipsoid plot of the asymmetric unit is shown at the bottom right corner (50% probability) along with the bond lengths given in [pm].

The elongation of the C–S bond naturally increases even further when moving to MTSA (**2**, **3**). Figure 2 shows a summary of the observable packing motifs as well as the thermal ellipsoid plot of the asymmetric unit (ASU) and relevant bond lengths. We received single crystals of the trigonal modification **2** from the solvothermal reaction of Bi(OAc)_3_ with SO_3_ at 100°C. Compound **2**, which crystallizes in space group *P*3*c*1 (No. 158), shows linear alignment of the central carbon atoms stacked along the crystallographic *c*‐axis (C···C = 457.5(7) pm), while forming an extended network of hydrogen bonds. Within the *c* plane, a complex formation of classical donor–acceptor hydrogen bonds is observed, which can be described as an R_3_^3^(14) pattern using the graph set notation introduced before. The O···O distances range between 264.2(3) and 265.2(4) pm while the O–H–O angles show values of 176(5)°, 167(5)° and 160(6)°, which would normally be considered moderate hydrogen bonds [50, 51]. Thus, the increasing steric demand with the introduction of additional sulfonic acid moieties acts as a counter force toward the intermolecular interactions. Both classical hydrogen bonding and nonclassical hydrogen bonding, that is, the interaction between the terminal S–O_t_ atoms and the highly acidic C–H proton (O···H = 238(1) pm) or the strongly electropositive methyl carbon (O···C = 316.9(5) pm), govern the highly symmetric packing within this modification. This might also be the reason for the slight deviation from a perfect *anti‐*configurational packing (S–C–C–S torsion = 162.6(2)°), which would be expected if the repulsion of the oxygen lone pairs were the structure‐building driving force.

**FIGURE 3:**
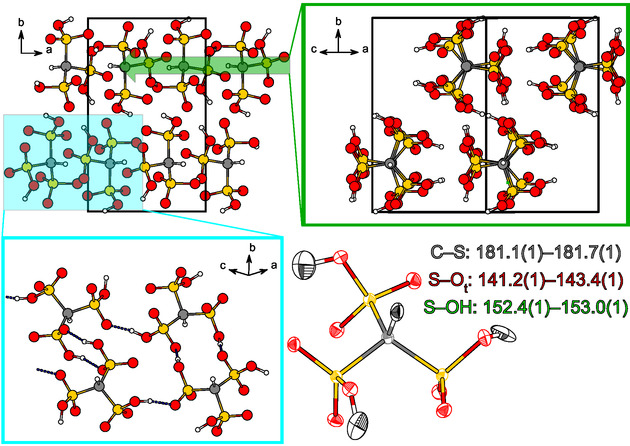
Solid‐state structure of [HC(SO_3_H)_3_]‐II (*P*2_1_/*n*, **3**). Packed unit cell (top left), depiction of the hydrogen bonding along [4.30, –5.79, 3.68] (bottom right), and the conformational view compared to **2** (top left). The thermal ellipsoid plot of the asymmetric unit is shown at the bottom right corner (50% probability) along with the bond lengths given in pm.

In contrast, if an ampoule of Cu(acac)_2_ with SO_3_ frozen on top of it is warmed to r.t. and left for 3 days, colorless single crystals grow at the glass wall, which were identified as the monoclinic modification **3** (see Figure 3). Due to the high quality of the measurement, we were able to refine the protons anisotropically using *NoSpherA2* [54]. As visible from the direct comparison of structures **2** and **3**, the latter one shows a significantly less ordered packing. Although the methyl carbon atoms are still packed in line within the unit cell, they are further apart (C···C = 464.0(1) pm). Additionally, a more complex hydrogen bonding network can be found within **3**, which, in terms of the [HC(SO_3_H)_3_] units, would be described as R_3_^3^(20) segments. The isolation of the less ordered, lower‐symmetric polymorph **3** from the reaction at lower temperatures gives reason to suggest that it is a kinetically stable phase, whereby the structure of **2** is thermodynamically favored.

**FIGURE 4:**
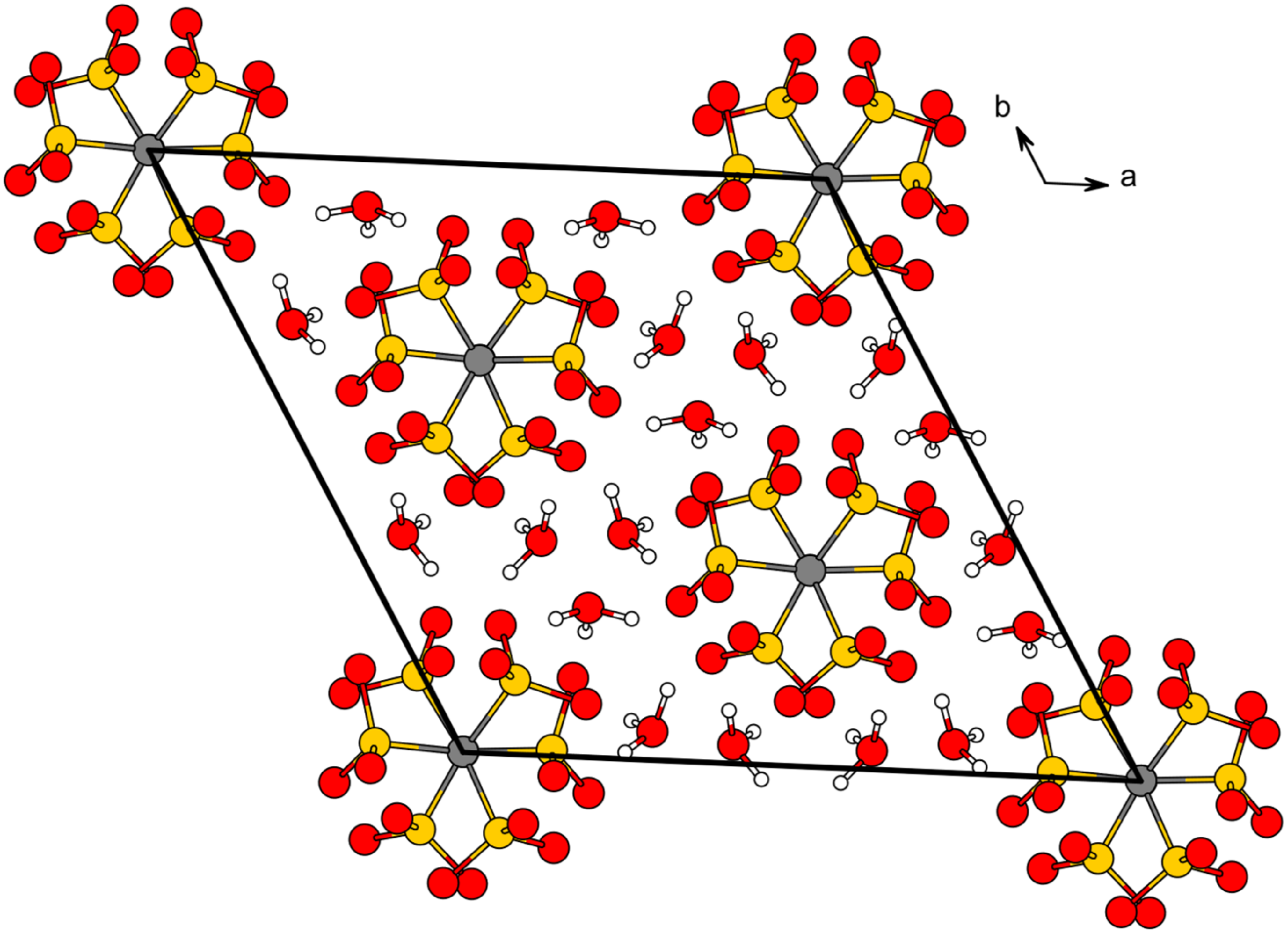
Packed unit cell [H_3_O]_3_[HC(SO_3_)_3_], viewed along the crystallographic c‐direction.

The strong influence of non‐covalent interactions on the packing in the solid state in the case of MTSA becomes even more apparent if the oxonium salt **4** is considered (see Figure 4), which shows a topotactic transition compared to the higher symmetric modification of [HC(SO_3_H)_3_]‐I (*P*3*c*1 → *R*3*c*) due to incorporation of the [H_3_O]^+^ cations.

In the presence of the oxonium ions, every terminal oxygen atom of the [MTS]^3–^ anion engages in hydrogen bonding. Thus, the S–O_t_ bond lengths elongate (143.3(1) pm and 146.6(1) pm), while the former S–OH bond slightly shortens (147.2(1) pm, due to the complete charge delocalization of the anion and the strong hydrogen bonding (O···O = 253.5(2), 261.3(3) and 262.2(2) pm, O–H–O = 163(3), 169(3) and 176(5)°). The additional space for the cations is achieved by the anions moving further apart (C···C = 488.7(4) pm), whilst maintaining the linear C···C stacking along the crystallographic *c*‐axis. This leads to an elongation of the one axis and an anion arrangement close to the perfect *anti‐*configurational packing (S–C–C–S torsion = 173.0(8)°).

### 2.2 Comparison of the Different Anions Within the Rubidium Salts

**FIGURE 5:**
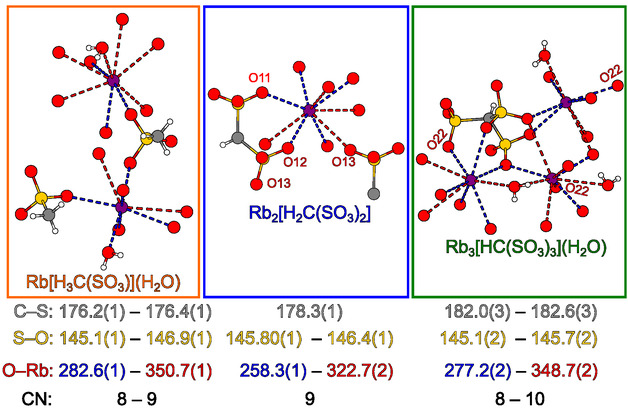
Overview of the cation–anion coordination, relevant bond lengths, interatomic distances, and coordination numbers (CN) of the cations for compounds **5** (left, orange), **8** (middle, blue), and **14** (right, green). The blue and red dotted lines indicate interatomic distances below and above 300 pm, respectively. All lengths are given in pm.

To have a direct comparison between the methane(poly‐)sulfonate anions, we aimed for the synthesis of a methanemono‐, di‐, and trisulfonate with the same cation. We chose rubidium as a model cation since, due to its large atomic form factor, SCXRD measurements of good quality are oftentimes easier to achieve compared to its lighter congeners, and we found the respective salts to crystallize better compared to the cesium compounds. We isolated the methanesulfonate Rb[H_3_C(SO_3_)](H_2_O) (**5**) and the ‐trisulfonate Rb_3_[HC(SO_3_)_3_](H_2_O) (**14**) as monohydrates, as well as the water‐free ‐disulfonate Rb_2_[H_2_C(SO_3_)_2_] (**8**). The cation···anion interactions and relevant structural data are summarized in Figure 5.

All compounds show similar coordination environments around the Rb^+^ cation, with coordination numbers between 8 and 10 as readily found for other (organo‐)oxoanionic rubidium compounds [22, 55, 58]. Intriguingly, the S–O_t_ bond lengths are in a narrow range for all compounds, suggesting that the charge delocalization over the terminal sulfonate groups is not influenced by increasing electron withdrawal at the methyl carbon and that all terminal oxygen atoms are similarly engaged in cation coordination. The C–S bond length of the methanesulfonate anion in **5** is markedly increased compared to its respective acid and the solvate Rb_2_[H_3_C(SO_3_)](H_3_CSO_3_H) [59], whereas the C–S bond lengths for the di‐ and trisulfonate salts are relatively unchanged compared to the parent acids. The Rb···O distances are in a comparable range for the two monohydrates **5** and **14,** while they are significantly shorter for the “closer packed” methanedisulfonate **8**. Although all three anions show multidentate coordination of the terminal sulfonates, it seems that while the size of the monosulfonate is not sufficient for a geometrically close packing, the size of the trisulfonate and the repulsion of adjacent anions are too large. Thus, the coordination sphere of the rubidium in both cases is saturated by additional water molecules. The formation of the hydrate cannot merely be traced back to the starting material since both **8** and **14** were prepared from the oxonium salts. Thermal analysis of the methanedisulfonate salt **8** shows, after release of some adhesive moisture, a decomposition temperature around 725 K (see Figure S14). The parallel monitored mass numbers of the decomposition fragments can be attributed to SO_2_ and SO, as well as to CO_2_. The decomposition residue is Rb_2_SO_4_, as validated by PXRD.

### 2.3 Additional Methanedisulfonate Salts

**FIGURE 6:**
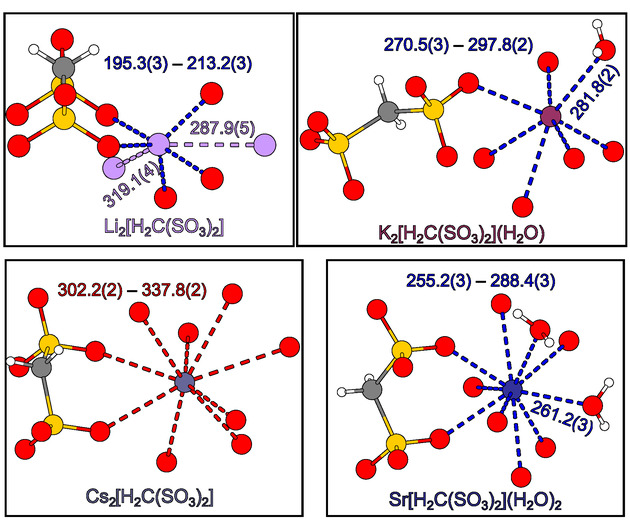
Overview of the cation–anion coordination and interatomic distances for compounds **6** (top left), **7** (top right), **9** (bottom right), and **10** (bottom left). The blue and red dotted lines indicate interatomic distances below and above 300 pm, respectively. All lengths are given in pm.

For all other compounds that we could isolate, the respective bond lengths within the anion do not differ significantly from what was already discussed and will therefore not be considered in detail. Instead, we will focus on the coordination environment and the interatomic O···M distances. For the remaining binary methanedisulfonates, these are summarized in Figure 6. Compounds **6**, **8**, **9,** and **10** were already isolated by *Backer*, but the respective structures remained elusive [60, 61]. IR spectra of the M_2_[H_2_C(SO_3_)_2_] salts are shown in Figure S28. Although the data quality of compounds **6** and **10** is modest, the vibrational modes of the anion do not change significantly for the different cations.

The lithium methanedisulfonate Li_2_[H_2_C(SO_3_)_2_] (**6**) shows a fivefold oxygen coordination around the cation. This is higher than that for “small” (organic) anions like formate [62] or fluorosulfate [63] but it is also found for the small (hydrogen‐)polysulfates [64, 65]. Notably, one of the Li···Li distances is remarkably short (287.5(5) pm), even below the one found in the fluorosulfate (296.5(3) pm). The compound can be prepared phase pure as judged by PXRD measurements with subsequent *Rietveld* refinement (c.f. Figure S10), but is hygroscopic and should be stored and handled under an inert atmosphere.

The monohydrate of potassium methanedisulfonate K_2_[H_2_C(SO_3_)_2_](H_2_O) **7** is a peculiar finding since the anhydrous salt K_2_[H_2_C(SO_3_)_2_] was upon the first methanedisulfonates that were structurally elucidated [39, 66]. Interestingly, **7** is not isostructural to its anhydrous salt (*C*2/*c*) but crystallizes in the orthorhombic space group *Pnma* (no. 53), thus being isotypic to the afore‐described lithium salt **6**. The C–S and S–O bond lengths are in good agreement with the values reported by *Truter* for the anhydrous compound. The potassium atoms show a sevenfold coordination of surrounding oxygen atoms, two less than for the water‐free salt, whereby the distances lie entirely below 300 pm, significantly shorter than known from reference compounds like the methanesulfonate, the disulfate, or the imido‐*bis*‐sulfate [67, 69]. We were not able to isolate the monohydrate selectively, but encountered it several times when we performed the synthetic route using oleum and acetone. Shortened reaction times seem to favor the formation of the methanedisulfonate, which would suggest that the subsequent sulfonation of acetone is kinetically controllable. Furthermore, if the reaction mixture is dried completely in vacuo, the anhydrous salt K_2_[H_2_C(SO_3_)_2_] can be isolated alongside KHSO_4_ as a byproduct from neutralization (see PXRD with *Rietveld* refinement in the Figure S12).

The general trend of increasing M···O distances due to stronger repulsion by the core electrons when moving to the heavier elements within a group is nicely visible for compounds **6**, **7,** and **10**. The cesium salt Cs_2_[H_2_C(SO_3_)_2_] **10** shows Cs···O distances exclusively above 300 pm and a ninefold coordination, whereby three “bidentate” and three “monodentate” coordinations are found. This mixed dentate coordination is known from other organic salts like the oxalate [70] and tetroxalate [71]. However, even compared to these small organic anions, the CN of 9 is relatively small, and the Cs···O distances are significantly shorter than those found within the known (hydrogen‐)polysulfates [72, 74]. The intramolecular O···O distances between the nearest terminal oxygen atoms of the two adjacent SO_3_ groups range for all compounds between 292.7(2) pm and 315.2(3) pm and seem, together with the O–S–S–O torsion angles around 0°, to foster the multidentate coordination of these cations.

As for the increasing M···O distances for Li < K < Cs, the stark effect of decreasing M···O distances when moving toward a higher charged cation is markedly shown by the comparison of the Cs salt and the strontium methanedisulfonate dihydrate Sr[H_2_C(SO_3_)_2_](H_2_O)_2_ (**9**). The Sr···O distances are shorter compared to the potassium salt **7** but significantly elongated compared to the methanesulfonate (249.7(3)–260.4(4) pm) [25], the oxalate (251.1(7)–269.6(7) pm) [75] and even the trisulfate (disorder, average = 255.73 pm) [76]. The CN of 10 for compound **9** is also higher than for the afore‐mentioned compounds (CN = 8). This might be a hint that the described denticity, though maybe suitable for promoting the smallest possible coordination for group I cations, cannot act in the same manner for the divalent group II cations. We originally aimed also at the isolation of the barium salt Ba[H_2_C(SO_3_)_2_](H_2_O)_*x*_ via salt metathesis, but instead only found the ternary salt BaK_2_[H_2_C(SO_3_)_2_]_2_ (**11**) (CN_K_ = 7, K···O = 261.3(2)–316.5(2) pm; CN_Ba_ = 9, Ba···O = 268.5(2)–303.5(2) pm, see Figure S18). Preparation of the barium salt by neutralization using Ba(OH)_2_ or BaCO_3_ is described in the literature but was not evaluated since we sought to elucidate the potential of salt‐exchange reactions. Thus, simple metathesis reactions starting from the group I salts might be disfavored due to the strong cation–anion interactions. Preparation of the sodium salt according to the method of *Backer* via reaction of Na_2_SO_3_ with H_2_CCl_2_ yielded colorless solids, but subsequent crystallization attempts toward single crystals of suitable quality for structure elucidation were unsuccessful.

### 2.4 Additional Methanetrisulfonate Salts

**FIGURE 7:**
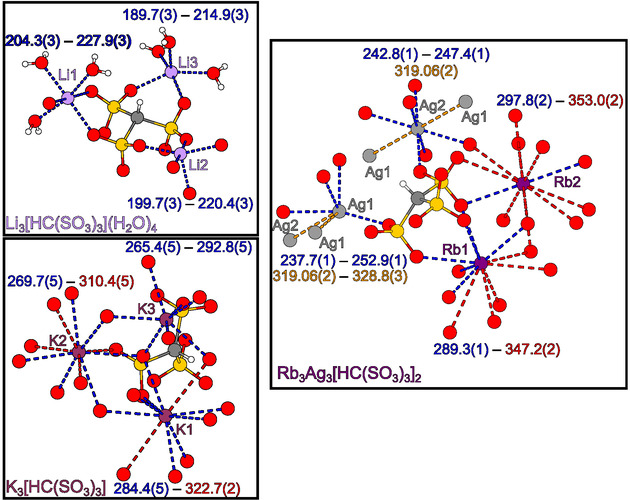
Overview of the cation–anion coordination and interatomic distances for compounds **12** (top left), **13** (bottom left), and **15** (right). The blue and red dotted lines indicate interatomic distances below and above 300 pm, respectively. The orange dotted lines indicate Ag···Ag distances. All lengths are given in pm.

Lastly, we want to shortly comment on the additional methanetrisulfonate salts which we were able to isolate during our studies (see Figure 7). A detailed structural description for MTS compounds in general was already published by *Oelkers* [40]. The binary lithium tetrahydrate Li_3_[HC(SO_3_)_3_](H_2_O)_4_ was reported by *Backer* as one of the first methanetrisulfonates [41]. Compound **12** can be readily prepared phase pure as judged by PXRD and subsequent *Rietveld* refinement (c.f. Figure S20). All terminal oxygen atoms of the anion are engaging in cation coordination, although with different strengths (terminal S–O_t_ bond lengths range between 144.4(1) and 146.3(1) pm). Two of the three Li^+^ cations show a fivefold oxygen coordination comparable to the methanedisulfonate compound **6**, while Li1 shows a sixfold distorted octahedral coordination. The different coordination motifs and less narrowly ranged Li···O distances compared to the methanedisulfonate are most likely due to the extended steric demand of the anion, which disfavors a denser cation/anion packing, leading also to a lower‐symmetric space group. Nonetheless, the Li···O distances are still remarkably short, with one Li···OH_2_ distance (189.7(3) pm) being close to the *van der Waals* radius [77, 78].

The anhydrous potassium salt K_3_[HC(SO_3_)_3_] **13** is similar to the hydrated salt of the methanedisulfonate salt, the “missing sibling” of the system.

Its monohydrate was also reported by *Backer* and later structurally elucidated by *Oelkers*. We received single crystals suitable for SXCRD measurement from the reaction of the monohydrate with ClSO_3_H in Cl_2_CH_2_, but **13** can simply be received phase pure by dehydration of the monohydrate K_3_[HC(SO_3_)_3_](H_2_O) in vacuo (judged by PXRD and *Rietveld* refinement in the Figure S22). **13** is not isostructural with its monohydrate (*P*2_1_2_1_2_1_) but crystallizes in the orthorhombic space group *Pbcm*. The bond lengths within the anion are in general agreement with the already mentioned trends and the literature, but the C–S bonds show an unusually large asymmetry (181.4(5) pm and 185.3(8) pm) [79]. The CNs for K2 and K1 (10) are higher compared to K_2_[H_2_C(SO_3_)_2_] and K_3_[HC(SO_3_)_3_](H_2_O). Several shared oxygen atoms are present, which is known from the monohydrate, where even a triply‐shared coordination is observed [40]. Due to its simple preparation, the potassium salt (anhydrous or as a hydrate) is a suitable starting point for subsequent chemical transformation, for example, toward the substitution of the methyl proton, as we have already shown for the brominated derivative K_3_[BrC(SO_3_)_3_](H_2_O) [21].

Upon our approaches to test whether ternary MTS salts are as readily available as the binary ones, we observed the formation of two different rubidium/silver compounds, namely Rb_3_Ag_3_[HC(SO_3_)_3_]_2_ (**15**) and Rb_5_Ag[HC(SO_3_)_3_]_2_(H_2_O)_2_ (**16**), from the reaction of the M(I) carbonates with the oxonium salt **4**. The existence of ternary methanetrisulfonates was already reported by *Oelkers* (Cs_2_(H_3_O)[CH(SO_3_)_3_](H_2_O)), but no ternary compounds containing a main group and a transition metal cation are reported so far; instead, several binary transition metal compounds ([Cu(H_2_O)_4_]^+^, [M(H_2_O)_6_]^3+^ (M = Cr, Co), [M’(H_2_O)_6_]^2+^[M’(H_2_O)_4_]^2+^_2_ (M’ = Ni, Zn) and Ni^II^ as an NMP solvate) have been reported [40, 80]. Compounds **15** and **16** are structurally completely different. The latter (see Figure S27) has rather high symmetry (*P*4_1_2_1_2) and shows an unusual tetrahedral oxygen coordination around the silver, as well as narrow‐ranged, very short Ag···O distances (237.0(3)–240.4(4) pm) that are shorter than those usually found for Ag^+^ in an oxoanionic environment (e.g., AgFSO_3_/AgOTf [81], or Ag_2_S_2_O_7_ [82]). It is at most comparable with the formate salt, which also shows a fourfold oxygen coordination and Ag···O distances ranging between 228.3(2) and 251.7(2) pm, but additionally close Ag···Ag distances of 291.76(5) pm [83]. Such short Ag···Ag distances are not present in **16,** and slightly longer distances are found within **15** (see Figure 7). The silver atoms within Rb_3_Ag_3_[HC(SO_3_)_3_]_2_ form linear Ag_3_ rods (Ag···Ag distance 319.06(2) pm, Ag–Ag–Ag = 180°) that grow in the *bc* plane and connect in the *ac* plane (Ag···Ag distance 328.8(3) to form infinite *zig*‐*zag* chains (see Figure S25). Although the Ag···Ag distances in **15** are not as short as found for other systems, the interactions can be described as argentophilic since they are still well below the sum of the *van der Waals* radii, and both the linearity of the Ag_3_ cluster and the electronic configuration of the Ag^+^ cation are the remaining requirements [84]. This highlights the significantly different structural influences of the monovalent coinage metal cations compared to the simpler alkaline metals.

## 3 Conclusion

We presented 16 novel compounds as the collected results of our investigations toward the methane(poly‐)sulfonic acids [H_4‐x_C(SO_3_)_*x*_] (*x* = 1–3) and their salts with group I and II cations. For the first time, the crystal structures of methanedisulfonic acid MDSA (**1**) and methanetrisulfonic acid MTSA (**2** and **3**) have been elucidated. We could show that, starting from the reaction of acetone and oleum, the potassium salts of both acids are readily available and can be transferred into the respective oxonium salts, thus enabling neutralization reactions toward several different salts. We compared the bond length changes and the different coordination motifs for methane(mono), ‐di, and ‐trisulfonate based on their respective rubidium salts and could show that, although the strong withdrawal introduced with the addition of another SO_3_ group rapidly elongates the C–S bond, all show comparable S–O bond lengths, coordination numbers, and O···Rb distances. Besides the nine novel binary salts, we could show that ternary systems can be isolated for both MDSA and MTSA. In the case of MTSA, we were able to show that the mixed cationic compound Rb_3_Ag_3_[HC(SO_3_)_3_]_2_ shows argentophilic interactions between the Ag^+^ cations.

## 4 Experimental Section

***Caution!*** Oleum (H_2_SO_4_·SO_3_) and chlorosulfuric acid (ClSO_3_H) are strong, oxidizing acids, which need careful handling. During the reaction and even after cooling down to room temperature, glass ampoules may be pressurized. The ampoules should be cooled with liquid nitrogen before opening.

### 4.1 Materials and General Procedure

If not stated otherwise within the respective procedures, all chemicals were used as received without further purification. Deionized water was used if not stated otherwise. 1,2‐Dichloromethane was refluxed with P_4_O_10_ for ∼5 h, followed by distillation and storage over molecular sieves (4 Å) prior to use. SO_3_ was generated by dehydration of Oleum (65% SO_3_) and subsequent distillation. Glassware was oven‐dried at 120°C prior to use.

### 4.2 Synthesis of K_3_[HC(SO_3_)_3_](H_2_O)

In an oven‐dried 250 mL three‐necked round‐bottom flask, equipped with a magnetic stirring bar and a *Dimroth* condenser, 25.0 mL (404.0 mmol SO_3_, 7.6 eq.) of fuming sulfuric acid (65% SO_3_, Merck, Darmstadt, Germany) was cooled to −10°C (ice/EtOH). Through a septum, 3.90 mL (53.1 mmol, 1.00 eq.) of acetone (technical grade) was added in small portions (∼2 mL/h) to avoid overoxidation (the mixture should remain colorless). After the addition was completed, the mixture was heated to 95°C and stirred for 3 h. The mixture was poured onto 200 g of crushed ice and neutralized with 67 g KOH (Thermo Scientific). The orange solution was filtered and stored at 7°C to initiate crystallization. After 24 h, the crude product was collected by filtration and recrystallized from hot water, whereby the mother liquor was subsequently stored for further crystallization, leading to an overall yield of 13 g (33.46 mmol, 63%) of K_3_[HC(SO_3_)_3_](H_2_O)**.**

### 4.3 Synthesis of H_2_C(SO_3_H)_2_ (1)

20.0 mg (0.11 mmol, 10.0 eq.) of MDSA (98%, BLDpharm), 20.0 mg Eu(OH)_3_ (0.01 mmol, 1.0 eq.), and a mixture of 0.2 mL (2.3 mmol, 230 eq.) F_3_CSO_3_H (Carbolution) and 0.1 mL (0.59 mmol, 5.9 eq.) of (F_3_CSO_2_)_2_O (Carbolution) were filled into an oven‐dried glass ampoule (3.3 borosilicate). The ampoule was sealed under reduced pressure (1·10^–3^ mbar) with a gas burner. The reaction vessel was heated to 100°C within 12 h, dwelled at this temperature for 48 h, and cooled down to room temperature (in the following r.t.) within 120 h. After cooling, a small number of colorless crystals of **1** were isolated from the mother liquor. The compound is hygroscopic and should be stored and handled in an inert atmosphere.

### 4.4 Synthesis of HC(SO_3_H)_3_‐I (2)

0.18 g (0.47 mmol, 1.00 eq.) of Bi(OAc)_3_ (Thermo Scientific) was filled into an oven‐dried glass ampoule (3.3 borosilicate). 0.20 mL SO_3_ (5.00 mmol, 10.64 eq.) was condensed on top, and the ampoule was sealed under reduced pressure (1·10^–3^ mbar) with a gas burner. The reaction vessel was heated to 100°C within 24 h, dwelled at this temperature for 48 h, and cooled down to r.t. within 90 h. After cooling, a small number of colorless crystals had grown at the upper end of the ampoule. The compound is hygroscopic and should be stored and handled in an inert atmosphere.

### 4.5 Synthesis of HC(SO_3_H)_3_‐II (3)

0.26 g (1.00 mmol, 1.00 eq.) of Cu(Acac)_2_ (97%, BLDpharm) was filled into an oven‐dried glass ampoule (3.3 borosilicate). 0.40 mL SO_3_ (10.00 mmol, 10.00 eq.) was condensed on top, and the ampoule was sealed under reduced pressure (1·10^–3^ mbar) with a gas burner. The reaction vessel was stored at room temperature. After 3 days, a small number of colorless crystals had grown in the middle and the upper end of the ampoule. The compound is hygroscopic and should be stored and handled in an inert atmosphere.

### 4.6 Synthesis of [H_3_O]_3_[HC(SO_3_)_3_] (4)

2.00 g (5.15 mmol, 1.00 eq.) of K_3_[HC(SO_3_)_3_](H_2_O) was dissolved in 10 mL H_2_O. The mixture was moderately heated to achieve complete dissolution and loaded onto an ion exchange column with a strongly acidic stationary phase (*Merck*, *Ionexchanger V*). The column was rinsed with several volumes of water until a neutral pH was reached. Another 200 mL of water was added onto the column, and the collected aqueous solutions were combined and reduced by a rotary evaporator, yielding 1.44 g (4.64 mmol, 90%) of (H_3_O)_3_[CH(SO_3_)_3_] as a hygroscopic solid.

### 4.7 Synthesis of Rb[H_3_C(SO_3_)](H_2_O) (5)

0.51 g (4.98 mmol, 1.00 eq.) of RbOH (Sigma Aldrich) and 0.48 g of H_3_CSO_3_H (4.99 mmol, 1.00 eq.) were filled into a preheated glass ampoule (3.3 borosilicate). The ampoule was sealed under reduced pressure (1·10^–3^ mbar) with a gas burner. The reaction vessel was heated to 100°C within 1 h, dwelled at this temperature for 2 h, and cooled down to r.t. within 14 h. After cooling, colorless crystals were isolated in an almost quantitative yield within the limits of PXRD measurements. The crystals are hygroscopic but can be handled under an inert atmosphere or in perfluorinated ether for single crystal selection.

### 4.8 Synthesis of K_2_[H_2_C(SO_3_)_2_] and [H_3_O]_2_[H_2_C(SO_3_)_2_]

0.50 g (3.16 mmol, 1.0 eq.) of K_2_SO_3_ (Merck), 0.50 mL of H_2_CCl_2_, and 0.50 mL of H_2_O were filled into a preheated glass ampoule (3.3 borosilicate). The ampoule was sealed under reduced pressure (1·10^−3^ mbar) with a gas burner. The reaction vessel was heated to 150°C within 6 h, dwelled at this temperature for 12 h, and cooled down to r.t. within 24 h. After cooling, colorless crystals of K_2_[H_2_C(SO_3_)_2_] were isolated almost quantitatively as judged by PXRD.

A solution of 1.00 g (3.96 mmol, 1.00 eq.) K_2_[H_2_C(SO_3_)_2_] in 10 mL of water was heated to 50°C until the solid was dissolved. The solution was poured onto an ion exchange column with a strong acidic stationary phase (*Merck*, *Ionexchanger V*). The column was rinsed with several volumes of water until a neutral pH was reached, another 200 mL of water was added onto the column, and the collected aqueous solutions were combined and dried in vacuo, yielding 0.36 g (1.69 mmol, 43%) of [H_3_O]_2_[H_2_C(SO_3_)_2_].

### 4.9 Synthesis of Li_2_[H_2_C(SO_3_)_2_] (6)

24.0 mg (1.00 mmol, 2.27 eq.) of LiOH (Sigma Aldrich) and 0.10 g of [H_3_O]_2_[H_2_C(SO_3_)_2_] (0.44 mmol, 1.00 eq.) were dissolved in 0.3 mL of H_2_O, and the solution was left to dry by slow evaporation, yielding a small number of colorless crystals of **6**. The compound is hygroscopic and should be stored and handled in an inert atmosphere.

### 4.10 Synthesis of K_2_[H_2_C(SO_3_)_2_](H_2_O) (7)

In an oven‐dried 50 mL *Schlenk* tube, Oleum (65%, 12.5 mL, 202.0 mmol SO_3_, 3.80 eq.) was added under an argon counterflow and cooled to −10°C (ice/EtOH). Through a septum, 3.90 mL (53.1 mmol, 1.00 eq.) of acetone (technical grade) was added in small portions (∼2 mL/h) to avoid overoxidation (the mixture should remain colorless). After all the acetone was added, the colorless reaction mixture was allowed to warm to room temperature before heating to 95°C and stirring for 45 min. The mixture turned brown and was poured on ice water. Subsequent neutralization with KOH and recrystallization from hot water gave 2.03 g K_2_[H_2_C(SO_3_)_2_](H_2_O) (7.51 mmol, 14%) as plate‐shaped colorless crystals.

### 4.11 Synthesis of Rb_2_[H_2_C(SO_3_)_2_] (8)

50.0 mg (0.49 mmol, 2.00 eq.) of RbOH (Sigma Aldrich) and 52.0 mg of [H_3_O]_2_[H_2_C(SO_3_)_2_] (0.24 mmol, 1.00 eq.) were dissolved in 1.00 mL of H_2_O, and the solution was left to dry by slow evaporation, yielding a small number of colorless crystals of **8**. The compound is hygroscopic and should be stored and handled in an inert atmosphere.

### 4.12 Synthesis of Sr[H_2_C(SO_3_)_2_](H_2_O)_2_ (9)

0.27 g (1.00 mmol, 1.00 eq.) of Sr(OH)_2_(H_2_O)_8_ (Thermo Scientific Alfa Aesar) and 0.18 g of [H_3_O]_2_[H_2_C(SO_3_)_2_] (1.00 mmol, 1.00 eq.) were stirred in 10.00 mL of H_2_O and heated to 60°C. After 15 minutes, the mixture was filtered, and the filtrate was left to dry by slow evaporation, yielding a small number of colorless crystals of **9**. The compound is hygroscopic and should be stored and handled in an inert atmosphere.

### 4.13 Synthesis of Cs_2_[H_2_C(SO_3_)_2_] (10)

0.05 g (0.33 mmol, 2.00 eq.) of CsOH (Sigma Aldrich) and 0.04 g of [H_3_O]_2_[H_2_C(SO_3_)_2_] (0.17 mmol, 1.00 eq.) were dissolved in 1.00 mL of H_2_O, and the solution was left to dry by slow evaporation, yielding a small number of colorless crystals of **10**. The compound is hygroscopic and should be stored and handled in an inert atmosphere.

### 4.14 Synthesis of BaK_2_[H_2_C(SO_3_)_2_] (11)

0.20 g (0.80 mmol, 1.00 eq.) of K_2_[H_2_C(SO_3_)_2_], 0.21g (1.00 mmol, 1.25 eq.) of BaCl_2_ (Sigma Aldrich), and 0.7 mL H_2_O were filled into a preheated glass ampoule (3.3 borosilicate). The ampoule was sealed under reduced pressure (1·10^−3^ mbar) with a gas burner. The reaction vessel was heated to 130°C within 4 h, dwelled at this temperature for 8 h, and cooled down to r.t. within 30 h. After cooling, a small number of colorless crystals of **11** were isolated. The compound is hygroscopic and should be stored and handled in an inert atmosphere.

### 4.15 Synthesis of Li_3_[HC(SO_3_)_3_](H_2_O)_4_ (12)

25.0 mg (0.34 mmol, 1.48 eq.) of Li_2_CO_3_ (Thermo Scientific Alfa Aesar), 71.0 mg (0.23 mmol, 1.00 eq.) of [H_3_O]_3_[HC(SO_3_H)_3_], and 0.1 mL H_2_O were filled into a preheated glass ampoule (3.3 borosilicate). The ampoule was sealed under reduced pressure (1·10^−3^ mbar) with a gas burner. The reaction vessel was heated to 100°C within 8 h, dwelled at this temperature for 10 h, and cooled down to r.t. within 100 h. After cooling, colorless crystals of **12** were isolated in quantitative yield as judged by PXRD. The crystals were stable under ambient conditions.

### 4.16 Synthesis of K_3_[HC(SO_3_)_3_] (13)

0.18 g (0.46 mmol, 1.0 eq.) of K_3_[HC(SO_3_)_3_](H_2_O) was slurried in 20 mL of H_2_CCl_2_, and 0.17 mL (2.55 mmol, 5.55 eq.) ClSO_3_H (97%, Thermo Scientific) was added, which led to precipitation of a fluffy solid. The overnatant solution was decanted away, and the solid dried in vacuo. A small number of single crystals suitable for SCXRD were obtained by recrystallization from ethylene glycol. The crystals were stable under ambient conditions.

### 4.17 Synthesis of Rb_3_[HC(SO_3_)_3_](H_2_O) (14)

0.16 g (0.68 mmol, 1.48 eq.) of Rb_2_CO_3_ (Sigma Aldrich), 0.14 g (0.46 mmol, 1.00 eq.) of [H_3_O]_3_[HC(SO_3_H)_3_], and 0.1 mL H_2_O were filled into a preheated glass ampoule (3.3 borosilicate). The ampoule was sealed under reduced pressure (1·10^−3^ mbar) with a gas burner. The reaction vessel was heated to 100°C within 8 h, dwelled at this temperature for 10 h, and cooled down to r.t. within 100 h. After cooling, a small number of colorless crystals of **14** were isolated. The crystals were stable under ambient conditions.

### 4.18 Synthesis of Rb_3_Ag_3_[HC(SO_3_)_3_]_2_ (15) and Rb_5_Ag[HC(SO_3_)_3_]_2_(H_2_O)_2_ (16)

53.0 mg (0.23 mmol, 2.09 eq.) of Rb_2_CO_3_ (Sigma Aldrich), 31.0 mg (0.11 mmol, 1.00 eq.) of Ag_2_CO_3_ (Sigma Aldrich), 71.0 mg ([H_3_O]_3_[HC(SO_3_H)_3_], and 0.1 mL H_2_O were filled into a preheated glass ampoule (3.3 borosilicate). The ampoule was sealed under reduced pressure (1·10^−3^ mbar) with a gas burner. The reaction vessel was heated to 100°C within 8 h, dwelled at this temperature for 10 h, and cooled down to r.t. within 100 h. After cooling, a small number of colorless crystals with different habitus were isolated, which were identified as **15** and **16**. The crystals were stable under ambient conditions.

### 4.19 Structure Determination and Crystallographic Details

#### 4.19.1 General Procedure

The single crystal structure determination was performed on a *Bruker D8 VENTURE KAPPA* diffractometer with a microfocus sealed tube using a multilayer mirror as the monochromator and a *Bruker PHOTON III* detector. As characteristic X‐ray radiation, MoK_α_ (*λ* = 71.073 pm) or AgK_α_ (*λ* = 56.086 pm) was used for the measurements. Before the measurements, the crystals were prepared in perfluorinated ether oil (Fomblin YR‐180) and selected using a light microscope with a polarization filter. A suitable single crystal was fixed on a MiTeGen micromount (150 µm polymer loop) and adjusted to the X‐ray beam and cooled down to 100 K in a stream of N_2_. The images with the intensity data were processed using the software *APEX5* [85]. The frames were integrated with the Bruker *SAINT* software package using a narrow frame algorithm. Absorption effects were corrected using SADABS for the multi‐scan absorption correction [86, 87]. If not stated otherwise within the structural chapters, the structure solution and refinement were done with the software *OLEX2* [88]. Dual methods using *SHELXT* were used for structure solution and full‐matrix least‐squares methods against *F* ^2^ using *SHELXL* for the refinement [89, 90]. All illustrations of the crystal structures were made with the program *Diamond 4* using the .cif as the input file [91].

#### 4.19.2 PXRD (Mo‐K_α1_ Radiation) and Rietveld Refinement

P‐XRD data were collected on a *Stoe* Stadi‐P powder diffractometer with a *Mythen* detector and a Debye−Scherrer geometry by using Mo‐K_α1_ radiation of *λ* = 0.70930 Å. The samples were grounded and sealed in glass capillaries with 0.5 mm diameter. The data were recorded in a range of 2*θ* = 0°–60° within 10.5 h. The collected intensities were treated by a Rietveld refinement in order to detect more reliably possible crystalline impurities. The refinement was performed using the software *TOPAS‐Academic* (*64 V6*) [92, 93]. The refinement process included the scale factor and lattice parameters, along with corrections for zero shift, specimen displacement, and background. Peak profiles were described using the pseudo‐Voigt function of Thompson, Cox, and Hastings [94], with the profile shape parameters u, v, w, and y refined accordingly.

#### 4.19.3 PXRD (Cu‐Kα Radiation)

Powders were measured on a Rigaku Miniflex with Cu‐Kα radiation, and data were collected on a *HyPix‐400 MF 2D* hybrid pixel array detector in Bragg‐Brentano geometry at room temperature. The samples were prepared on glass holders. Data were processed with *SmartLab Studio II*, version 4.4.295.0 [95].

#### 4.19.4 IR Spectroscopy

The IR spectra were measured on a *PerkinElmer* FTIR‐ATR (*UATR TWO*) at room temperature with a maximum resolution of 1 cm^−1^. Data were processed with the program *Spectrum*, version 10.6.1.942 [96]. For graphical depiction of the results, OriginPro 2024 was used [97].

#### 4.19.5 Quantum Chemical calculations

The IR frequencies for the isolated methanedisulfonate anion were calculated at the PBE0‐cc‐pVTZ [98, 99] level of theory using the software *Gaussian* [100].

## Author Contributions

J. L., A.M, K.E., and J.C. synthesized and characterized the compounds. J.L. wrote the original draft. J.L., K.E., J.C., and M.S.W. contributed to the final draft of the manuscript. M.S.W. supervised the project.

## Conflicts of Interest

The authors declare no conflicts of interest.

## Supporting information

Supplementary Material

## Data Availability

The data that support the findings of this study are available in the Supporting Information of this article.
