# Supplementary material for: The Versatile Structural World of Methanedi‐ and Trisulfonic Acid and Their Salts
Source: ChemistryOpen. 2026 Jun 30;15(7):e70247. doi: 10.1002/open.70247 (PMC13318925; doi:10.1002/open.70247)
Supplement: Supplementary file 1 — Supplementary Material [file OPEN-15-e70247-s001.pdf]

The Versatile Structural World of Methanedi- and Trisulfonic Acid and Their Salts.

Katrin Eppers, Alisha Mertens, Jan Chrubasik, Mathias S. Wickleder* and Jan Langwald

Institute of Inorganic and Materials Chemistry, University of Cologne, Greinstr. 6, 50939 Cologne, Germany. E-mail: mathias.wickleder@uni-koeln.de

Table of Contents

$H_2C(SO_3H)_2$ 2

$HC(SO_3H)_3$–I (2) 6

$HC(SO_3H)_3$–II (3) 10

$[H_3O]_3[HC(SO_3)_3]$ (4) 13

$Rb[H_3C(SO_3)](H_2O)$ (5) 16

$Li_2[H_2C(SO_3)_2]$ (6) 22

$K_2[H_2C(SO_3)_2](H_2O)$ (7) 26

$Rb_2[H_2C(SO_3)_2]$ (8) 30

$Sr[H_2C(SO_3)_2](H_2O)_2$ (9) 35

$Cs_2[H_2C(SO_3)_2]$ (10) 39

$BaK_2[H_2C(SO_3)_2]_2$ (11) 43

$Li_3[HC(SO_3)_3](H_2O)_4$ (12) 51

$K_3[HC(SO_3)_3]$ (13) 56

$Rb_3[HC(SO_3)_3](H_2O)$ (14) 63

$Rb_3Ag_3[HC(SO_3)_3]_2$ (15) 71

$Rb_5Ag[HC(SO_3)_3]_2(H_2O)_2$ (16) 79

E. Spectroscopic investigations 88

$H_2C(SO_3H)_2$

Table S1: Crystallographic data of $H_2C(SO_3H)_2$.

Empirical formula	$CH_4O_6S_2$
Formula weight	176.16 g/mol
Temperature	120(2) K
Crystal system	monoclinic
Space group	$C2/c$ (No. 15)
Unit cell dimensions	a = 881.36(4) pm<br>b = 471.74(2) pm<br>c = 1289.43(6) pm<br>β = 100.286(2)°
Volume	527.49(4) $Å^3$
Z	4
ρ_{calc}	2.218 g/cm^3
μ	0.965 mm^{-1}
F(000)	360
Crystal size	0.321 × 0.211 × 0.167 mm^3
Radiation	MoK_α (λ = 0.71073 nm)
2Θ range for data collection	6.422 to 75.55
Index ranges	$-15 \leq h \leq 14, -8 \leq k \leq 8, -22 \leq l \leq 2$
Reflections collected	15663
Independent reflections	1423 [R_{int} = 0.0206, R_σ = 0.0093]
Completeness	100%
Absorption correction	multiscan
Min. and max. transmission	0.828 / 0.94
Data/restraints/parameters	1423/0/50
Goodness-of-fit on F^2	1.078
Final R indexes [$I \geq 2\sigma$ (I)]	R_1 = 0.0151, wR_2 = 0.0473
Final R indexes [all data]	R_1 = 0.0160, wR_2 = 0.0479
Largest diff. peak/hole	0.44/–0.36 e · $Å^{-3}$
CCDC-No.	1046562

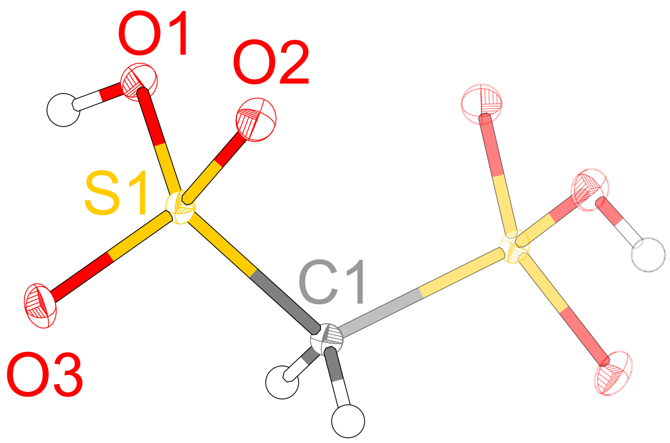

Figure S1: Thermal ellipsoid plot of the asymmetric unit $H_2C(SO_3H)_2$. Thermal ellipsoids shown with 50% probability. Atoms generated due to symmetry for representation of the complete molecule are shown at 50% visibility.

Table S2: Fractional Atomic Coordinates (×10^4) and Equivalent Isotropic Displacement Parameters (Å^2×10^3) for $H_2C(SO_3H)_2$. U_{eq} is defined as 1/3 of the trace of the orthogonalised U_{IJ} tensor.

Atom	*x*	*y*	*z*	U(eq)
S1	4170.4(2)	6584.5(3)	1374.4(2)	7.09(4)
O1	5536.9(6)	8212.7(10)	1052.5(4)	10.84(8)
O2	3155.8(6)	8640.3(10)	1686.3(4)	11.52(8)
O3	3570.2(6)	4564.3(11)	571.2(4)	11.43(8)
C1	5000	4560.9(17)	2500	7.90(11)

Table S3: Anisotropic Displacement Parameters (Å^2×10^3) for $H_2C(SO_3H)_2$. The anisotropic displacement factor exponent takes the form: $-2\pi^2[h^2a^{*2}U_{11}+2hka^*b^*U_{12}+\ldots]$.

Atom	U_{11}	U_{22}	U_{33}	U_{23}	U_{13}	U_{12}
S1	7.20(6)	8.08(6)	6.13(6)	-0.36(4)	1.61(4)	-0.24(4)
O1	12.34(18)	11.34(18)	10.06(18)	-0.42(13)	5.31(14)	-3.35(14)
O2	11.24(18)	12.30(18)	11.37(18)	0.52(14)	2.95(14)	4.44(14)
O3	12.03(18)	13.96(19)	8.26(17)	-3.50(14)	1.69(14)	-3.52(14)
C1	8.9(3)	7.3(3)	7.7(3)	0	1.8(2)	0

Table S4: Bond Lengths for $H_2C(SO_3H)_2$ in pm.

Atom	Atom	Length/pm	Atom	Atom	Length/pm
S1	O1	154.63(5)	S1	C1	178.14(4)
S1	O2	142.50(5)	C1	S1[1]	178.14(4)
S1	O3	143.70(5)			

[1]1-X,+Y,1/2-Z

Table S5: Bond Angles for $H_2C(SO_3H)_2$.

Atom	Atom	Atom	Angle/°	Atom	Atom	Atom	Angle/°
O1	S1	C1	105.00(2)	O3	S1	O1	109.88(3)
O2	S1	O1	107.21(3)	O3	S1	C1	106.06(3)
O2	S1	O3	119.07(3)	S1[1]	C1	S1	115.19(4)
O2	S1	C1	108.77(3)				

[1]1-X,+Y,1/2-Z

Table S6: Torsion Angles for $H_2C(SO_3H)_2$.

A	B	C	D	Angle/°	A	B	C	D	Angle/°
O1	S1	C1	$S1^1$	63.96(2)	O3	S1	C1	$S1^1$	-179.71(2)
O2	S1	C1	$S1^1$	-50.53(2)					
O1	S1	C1	$S1^1$	63.96(2)	O3	S1	C1	$S1^1$	-179.71(2)

[1]1-X,+Y,1/2-Z

Table S7: Hydrogen Atom Coordinates (Å×10^4) and Isotropic Displacement Parameters (Å^2×10^3) for $H_2C(SO_3H)_2$.

Atom	*x*	*y*	*z*	U(eq)
H1	5859(19)	7380(40)	630(13)	44(4)
H1A	4221(14)	3450(30)	2683(10)	18(3)

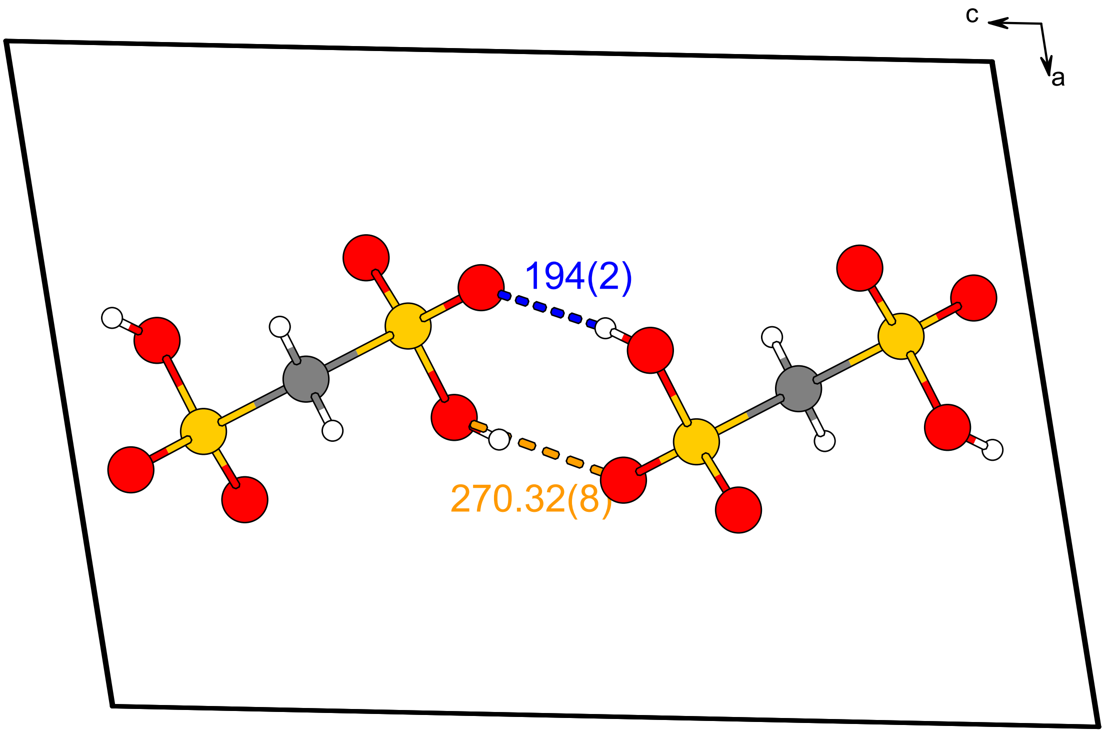

Figure S 2: Unit cell of $H_2C(SO_3H)_2$ viewed along the crystallographic *b*-axis. O···H and O···O distances are shown as blue and orange dotted lines respectively. The interatomic distances are given in [pm].

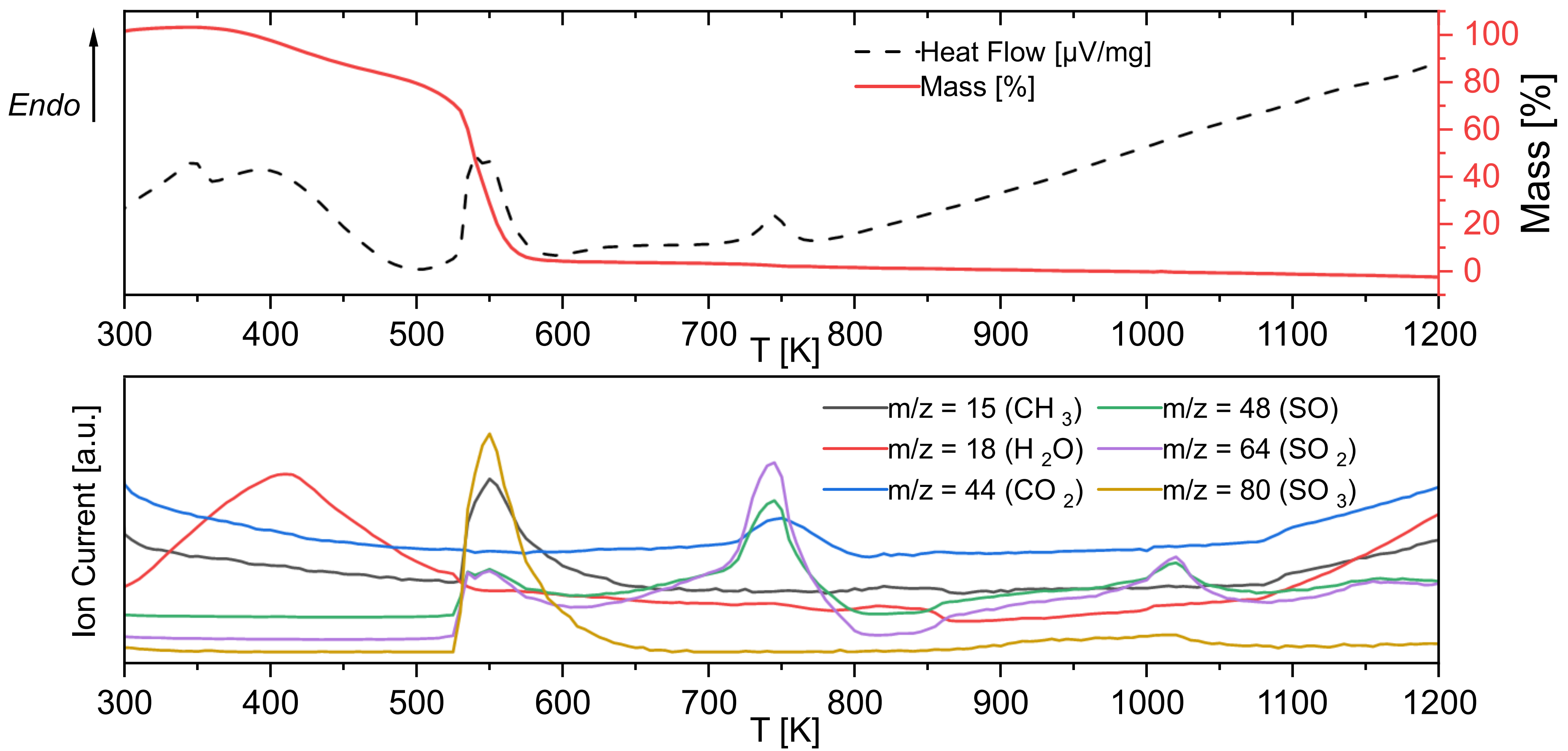

Figure S 3: DSC-TG/MS measurement of a sample of $[H_3O]_2[H_2C(SO_3)_2]$, heated with 5 K/min to a maximum temperature of 1260 K. The bottom part shows the detected mass numbers of the decomposition fragments: (m/z = 15 (CH3), m/z = 18 (H2O), m/z = 44 (CO2), m/z = 48 (SO), m/z = 64 (SO2), m/z = 80 (SO3)).

$HC(SO_3H)_3$–I (2)

The structure of (**2**) was refined using a basic twin law [-1 0 0 0 -1 0 0 0 -1] and a Batch Scale Factor of 0.44613.

Table S8: Crystallographic data of $HC(SO_3H)_3$–I.

Empirical formula	$CH_4O_9S_3$
Formula weight	256.22 g/mol
Temperature	100(2) K
Crystal system	trigonal
Space group	*P*3*c*1 (No. 158)
Unit cell dimensions	a = 1181.12(3) pm<br>b = 1181.12(3) pm<br>c = 915.01(4) pm<br>γ = 120°
Volume	1709.6(1) Å^3
Z	6
ρ_{calc}	2.309 g/cm^3
μ	1.033 mm^{-1}
F(000)	780
Crystal size	0.06 × 0.04 × 0.003 mm^3
Radiation	MoK$_\alpha$ (λ = 0.71073 nm)
2Θ range for data collection	3.982 to 60.968
Index ranges	$-16 \leq h \leq 16$, $-16 \leq k \leq 16$, $-13 \leq l \leq 13$
Reflections collected	24733
Independent reflections	2227 [R_{int} = 0.0180, R_σ = 0.0091]
Completeness	99.9%
Absorption correction	multiscan
Min. and max. transmission	0.704 / 0.746
Data/restraints/parameters	2227/13/137
Goodness-of-fit on F^2	1.190
Final R indexes [I≥2σ (I)]	R_1 = 0.0248, wR_2 = 0.0615
Final R indexes [all data]	R_1 = 0.0266, wR_2 = 0.0646
Largest diff. peak/hole	0.47/–0.40 e · Å^{-3}
Flack parameter	0.45(11)
CCDC-No.	2499370

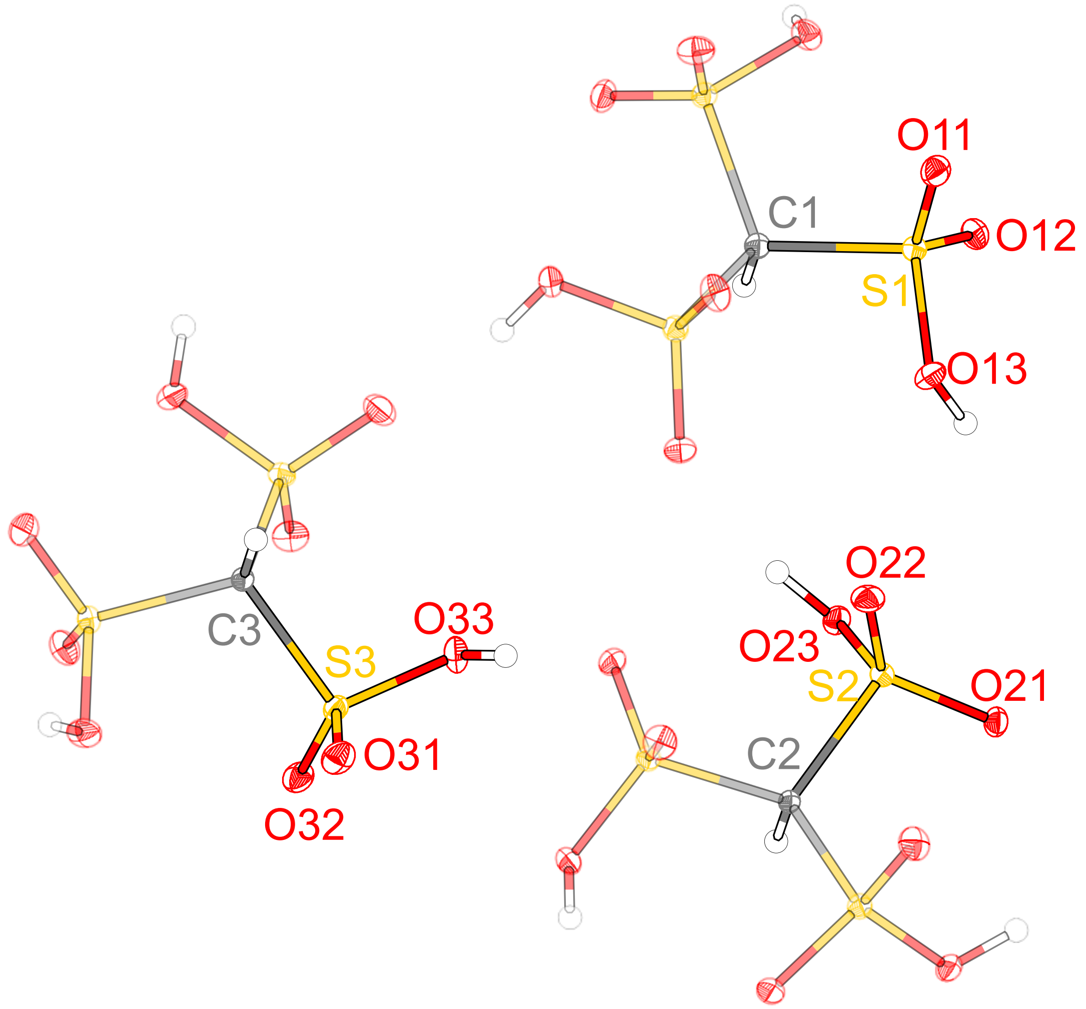

Figure S4: Thermal ellipsoid plot of the asymmetric unit $HC(SO_3H)_3$–I. Thermal ellipsoids shown with 50% probability. Atoms generated due to symmetry for representation of the complete molecule are shown at 50% visibility.

Table S9: Fractional Atomic Coordinates (×10^4) and Equivalent Isotropic Displacement Parameters (Å^2×10^3) for $HC(SO_3H)_3$–I. U_{eq} is defined as 1/3 of the trace of the orthogonalised U_{IJ} tensor.

Atom	*x*	*y*	*z*	U(eq)
S1	8405.4(7)	8699.4(7)	6584.7(8)	8.79(15)
O11	8507(2)	8491(2)	8093(3)	13.3(4)
O12	7978(2)	7639(2)	5566(3)	12.4(4)
O13	7567(2)	9352(2)	6399(3)	11.8(4)
C1	10000	10000	5964(5)	8.8(9)
S2	8229.9(8)	4642.8(7)	6068.2(7)	8.14(15)
O21	9141(2)	4213(2)	5731(3)	10.9(4)
O22	8088(2)	4943(2)	7539(3)	12.7(4)
O23	8531(2)	5768(2)	5023(3)	10.9(4)
C2	6666.67	3333.33	5441(6)	7.5(9)
S3	4659.1(7)	8237.3(7)	3406.0(8)	8.80(15)
O31	5709(2)	8612(2)	4408(3)	14.3(5)
O32	4860(3)	8131(3)	1895(3)	12.9(5)
O33	4062(3)	9127(2)	3582(3)	13.1(5)
C3	3333.33	6666.67	4016(6)	8.0(9)

Table S10: Anisotropic Displacement Parameters ($Å^2 \times 10^3$) for $HC(SO_3H)_3$–I. Anisotropic displacement factor exponent takes the form: $-2\pi^2[h^2a^{*2}U_{11}+2hka^*b^*U_{12}+\ldots]$.

Atom	U_{11}	U_{22}	U_{33}	U_{23}	U_{13}	U_{12}
S1	7.9(3)	8.2(3)	10.1(3)	0.5(2)	0.1(2)	3.9(3)
O11	10.7(10)	16.2(11)	11.8(11)	3.2(8)	1.1(8)	5.8(9)
O12	12.3(10)	7.9(10)	15.8(10)	-1.1(8)	-0.9(8)	4.2(8)
O13	9.4(10)	13.8(11)	13.8(10)	-2.0(8)	-2.3(8)	7.0(9)
C1	8.6(13)	8.6(13)	9(2)	0	0	4.3(6)
S2	7.5(3)	7.7(3)	8.9(3)	-0.3(3)	-0.2(3)	3.6(3)
O21	7.9(9)	10.6(10)	14.9(11)	0.8(9)	0.9(8)	5.2(8)
O22	14.8(11)	13.3(11)	9.9(10)	-3.6(9)	-1.0(9)	6.8(10)
O23	9.9(10)	7.8(11)	14.6(11)	2.5(8)	1.4(8)	4.3(8)
C2	6.2(14)	6.2(14)	10(2)	0	0	3.1(7)
S3	8.2(3)	7.0(3)	9.8(3)	-0.2(3)	-0.1(3)	2.7(3)
O31	11.1(11)	12.5(11)	16.3(12)	-1.2(9)	-3.4(9)	3.7(9)
O32	15.1(12)	11.3(11)	10.5(11)	1.8(8)	2.4(9)	5.2(9)
O33	15.6(11)	10.3(11)	15.8(12)	-3.0(9)	-3.1(9)	8.2(9)
C3	6.6(12)	6.6(12)	11(2)	0	0	3.3(6)

Table S11: Bond Lengths for $HC(SO_3H)_3$–I in pm.

Atom	Atom	Length/pm	Atom	Atom	Length/pm
S1	O11	141.8(2)	S2	O23	152.8(2)
S1	O12	143.5(2)	S2	C2	181.0(2)
S1	O13	153.8(2)	S3	O31	142.3(3)
S1	C1	182.7(2)	S3	O32	141.9(3)
S2	O21	143.4(2)	S3	O33	153.9(3)
S2	O22	142.3(3)	S3	C3	181.6(2)

Table S12: Bond Angles for $HC(SO_3H)_3$–I.

Atom	Atom	Atom	Angle/°	Atom	Atom	Atom	Angle/°
O11	S1	O12	120.26(15)	O23	S2	C2	103.85(17)
O11	S1	O13	109.05(14)	$S2^3$	C2	S2	110.45(16)
O11	S1	C1	107.91(18)	$S2^3$	C2	$S2^4$	110.44(16)
O12	S1	O13	109.35(14)	S2	C2	$S2^4$	110.44(16)
O12	S1	C1	107.29(16)	O31	S3	O33	110.27(15)
O13	S1	C1	101.30(10)	O31	S3	C3	106.97(18)
S1	C1	$S1^1$	110.80(14)	O32	S3	O31	119.77(16)
S1	C1	$S1^2$	110.80(14)	O32	S3	O33	108.46(15)
$S1^1$	C1	$S1^2$	110.80(14)	O32	S3	C3	107.8(2)
O21	S2	O23	106.48(14)	O33	S3	C3	102.11(11)
O21	S2	C2	104.75(12)	S3	C3	$S3^5$	111.01(17)
O22	S2	O21	119.65(15)	$S3^5$	C3	$S3^6$	111.01(17)
O22	S2	O23	112.34(15)	S3	C3	$S3^6$	111.01(17)
O22	S2	C2	108.41(19)				

^{1}1+Y-X,2-X,+Z; ^{2}2-Y,1+X-Y,+Z; ^{3}1+Y-X,1-X,+Z; ^{4}1-Y,+X-Y,+Z; ^{5}1-Y,1+X-Y,+Z; ^{6}+Y-X,1-X,+Z

Table S13: Torsion Angles for $HC(SO_3H)_3$–I.

A	B	C	D	Angle/°	A	B	C	D	Angle/°
O11	S1	C1	$S1^1$	-75.6(2)	O22	S2	C2	$S2^3$	41.2(3)
O11	S1	C1	$S1^2$	47.8(2)	O23	S2	C2	$S2^3$	-78.4(3)

O12	S1	C1	S1[2]	-83.1(2)	O23	S2	C2	S2[4]	159.1(2)
O12	S1	C1	S1[1]	153.5(2)	O31	S3	C3	S3[5]	-81.7(3)
O13	S1	C1	S1[1]	38.9(2)	O31	S3	C3	S3[6]	154.3(2)
O13	S1	C1	S1[2]	162.30(18)	O32	S3	C3	S3[6]	-75.7(2)
O21	S2	C2	S2[3]	170.06(19)	O32	S3	C3	S3[5]	48.3(3)
O21	S2	C2	S2[4]	47.6(3)	O33	S3	C3	S3[5]	162.4(2)
O22	S2	C2	S2[4]	-81.2(2)	O33	S3	C3	S3[6]	38.5(3)

[1]1+Y-X,2-X,+Z; [2]2-Y,1+X-Y,+Z; [3]1-Y,+X-Y,+Z; [4]1+Y-X,1-X,+Z; [5]+Y-X,1-X,+Z; [6]1-Y,1+X-Y,+Z

Table S14: Hydrogen Atom Coordinates (Å×10^4) and Isotropic Displacement Parameters (Å^2×10^3) for $HC(SO_3H)_3$–I.

Atom	*x*	*y*	*z*	U(eq)
H13	7020(50)	9070(70)	5660(50)	45(18)
H1	10000	10000	4830(30)	11(15)
H23	8180(60)	6240(60)	5300(60)	60(20)
H2	6666.67	3333.33	4310(30)	4(16)
H33	4390(60)	9670(60)	4320(50)	60(20)
H3	3333.33	6666.67	5150(30)	30(30)

HC(SO3H)3–II (3)

The structure of (**3**) was solved using SHELXT,[1] and refined against F^2 using olex2.refine in Olex2.[2] In addition, Hirshfeld atom refinement was performed to obtain accurate scattering factors for all atoms using NoSpherA2.[3]

Table S15: Crystallographic data of $HC(SO_3H)_3$–II.

Empirical formula	$CH_4O_9S_3$
Formula weight	256.22 g/mol
Temperature	100(2) K
Crystal system	monoclinic
Space group	$P2_1/n$ (No. 14)
Unit cell dimensions	a = 788.37(4) pm
	b = 1190.44(6) pm
	c = 884.30(5) pm
	β = 112.888(2)°
Volume	764.58(7) Å^3
Z	4
ρ_{calc}	2.226 g/cm^3
μ	0.995 mm^{-1}
F(000)	522
Crystal size	0.06 × 0.05 × 0.02 mm^3
Radiation	MoK_α (λ = 0.71073 nm)
2Θ range for data collection	5.88 to 72.82
Index ranges	$-13 \leq h \leq 13$, $-19 \leq k \leq 19$, $-14 \leq l \leq 14$
Reflections collected	16989
Independent reflections	3715 [R_{int} = 0.0236, R_σ = 0.0183]
Completeness	99.9%
Absorption correction	Multiscan
Min. and max. transmission	0.631 / 0.747
Data/restraints/parameters	3715/0/145
Goodness-of-fit on F^2	1.058
Final R indexes [$I \geq 2\sigma(I)$]	R_1 = 0.0288, wR_2 = 0.0618
Final R indexes [all data]	R_1 = 0.02377, wR_2 = 0.0697
Largest diff. peak/hole	0.67/–0.45 e · Å^{-3}
CCDC-No.	2517624

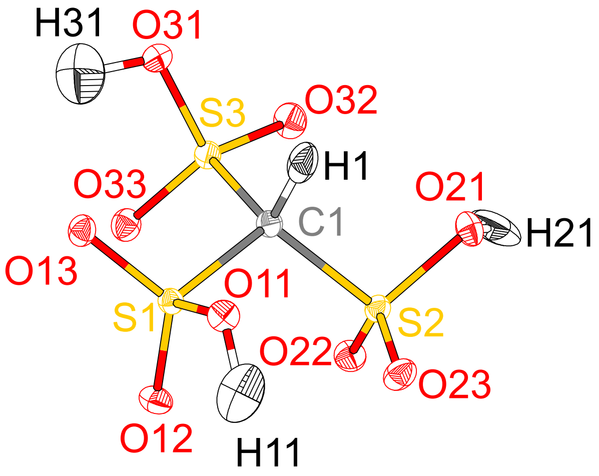

Figure S5: Thermal ellipsoid plot of the asymmetric unit $HC(SO_3H)_3$–II. Thermal ellipsoids shown with 50% probability.

Table S16: Fractional Atomic Coordinates (×10^4) and Equivalent Isotropic Displacement Parameters (Å^2×10^3) for $HC(SO_3H)_3$–II. U_{eq} is defined as 1/3 of the trace of the orthogonalised U_{IJ} tensor.

Atom	*x*	*y*	*z*	U(eq)
S1	4814.2(4)	2305.0(2)	5637.7(3)	11.16(5)
S2	7030.4(4)	1449.2(2)	3827.0(3)	11.79(5)
S3	6845.8(4)	3921.2(2)	4394.0(3)	12.09(5)
O11	5392.9(13)	1344.0(8)	6873.3(11)	15.00(15)
O12	3337.3(12)	1998.5(8)	4156.0(11)	16.05(16)
O13	4660.9(13)	3299.7(8)	6490.9(11)	14.77(15)
O21	9098.1(12)	1438.2(8)	4215.3(12)	16.00(16)
O22	5985.6(13)	1786.1(8)	2185.8(11)	16.27(16)
O23	6585.7(14)	428.6(8)	4448.7(12)	17.36(16)
O31	7525.4(13)	4644.7(8)	5945.9(12)	16.05(16)
O32	8165.6(14)	3935.1(8)	3679.2(12)	18.47(17)
O33	4954.0(13)	4183.2(8)	3385.0(11)	15.95(16)
C1	6862.1(15)	2520.9(9)	5217.3(13)	11.47(17)

Table S17: Anisotropic Displacement Parameters (Å^2×10^3) for [$HC(SO_3H)_3$–II. The anisotropic displacement factor exponent takes the form: $-2\pi^2[h^2a^{*2}U_{11}+2hka^*b^*U_{12}+\ldots]$.

Atom	U_{11}	U_{22}	U_{33}	U_{23}	U_{13}	U_{12}
S1	10.71(10)	12.21(11)	11.39(10)	-0.52(8)	5.22(8)	-0.38(8)
S2	12.66(11)	11.47(11)	12.61(11)	-0.21(8)	6.40(9)	-1.00(8)
S3	12.75(11)	11.08(11)	13.85(11)	-0.20(8)	6.72(9)	0.57(8)
O11	16.9(4)	14.6(4)	14.5(4)	-0.6(3)	7.2(3)	2.9(3)
H11	60(20)	43(18)	19(14)	6(16)	28(15)	2(13)
O12	12.7(3)	19.8(4)	14.0(3)	-2.4(3)	3.4(3)	-1.1(3)
O13	16.1(4)	14.6(4)	16.4(4)	0.6(3)	9.4(3)	-0.7(3)
O21	13.5(4)	20.4(4)	15.9(4)	2.8(3)	7.6(3)	1.4(3)
H21	17(15)	41(19)	50(20)	-12(13)	8(14)	3(15)
O22	15.5(4)	19.9(4)	12.5(3)	0.3(3)	4.4(3)	-1.4(3)
O23	21.8(4)	13.1(4)	20.5(4)	-2.8(3)	11.8(3)	-1.7(3)
O31	16.0(4)	13.5(4)	18.8(4)	-1.6(3)	6.9(3)	-3.3(3)
H31	50(20)	50(20)	50(20)	11(18)	21(18)	4(18)
O32	20.5(4)	18.1(4)	22.0(4)	-1.3(3)	14.0(3)	1.7(3)
O33	16.6(4)	14.4(4)	16.0(4)	2.4(3)	5.5(3)	1.5(3)
C1	11.7(4)	12.2(4)	11.4(4)	-0.4(3)	5.5(3)	-0.1(3)
H1	29(13)	17(11)	36(14)	-1(10)	25(12)	-3(10)

Table S18: Bond Lengths for $HC(SO_3H)_3$–II im [pm].

Atom	Atom	Length/pm	Atom	Atom	Length/pm
S1	O11	152.41(9)	S2	O23	143.2(1)
S1	O12	142.09(9)	S2	C1	181.2(1)
S1	O13	143.42(9)	S3	O31	153.0(1)
S1	C1	181.1(1)	S3	O32	141.2(1)
S2	O21	152.97(9)	S3	O33	144.3(1)
S2	O22	141.97(9)	S3	C1	181.7(1)

Table S19: Bond Angles for $HC(SO_3H)_3$–II.

Atom	Atom	Atom	Angle/°	Atom	Atom	Atom	Angle/°
O12	S1	O11	112.20(6)	C1	S2	O23	104.09(5)
O13	S1	O11	107.79(5)	O32	S3	O31	109.45(6)
O13	S1	O12	119.38(6)	O33	S3	O31	110.50(5)
C1	S1	O11	102.21(5)	O33	S3	O32	119.01(6)
C1	S1	O12	108.87(5)	C1	S3	O31	102.21(5)
C1	S1	O13	104.78(5)	C1	S3	O32	107.37(6)
O22	S2	O21	112.07(5)	C1	S3	O33	106.89(5)
O23	S2	O21	107.77(6)	S2	C1	S1	110.23(6)
O23	S2	O22	120.05(6)	S3	C1	S1	110.16(6)
C1	S2	O21	101.92(5)	S3	C1	S2	111.41(6)
C1	S2	O22	109.16(5)				

Table S20: Torsion Angles for $HC(SO_3H)_3$–II.

A	B	C	D	Angle/°	A	B	C	D	Angle/°
S1	C1	S2	O21	-156.02(6)	S2	C1	S3	O31	-156.63(6)
S1	C1	S2	O22	85.30(7)	S2	C1	S3	O32	-41.50(8)
S1	C1	S2	O23	-44.02(7)	S2	C1	S3	O33	87.26(7)
S1	C1	S3	O31	80.72(6)	S3	C1	S1	O11	-158.98(6)
S1	C1	S3	O32	-164.15(6)	S3	C1	S1	O12	82.17(7)
S1	C1	S3	O33	-35.40(7)	S3	C1	S1	O13	-46.61(7)
S2	C1	S1	O11	77.68(6)	S3	C1	S2	O21	81.37(7)
S2	C1	S1	O12	-41.18(7)	S3	C1	S2	O22	-37.31(7)
S2	C1	S1	O13	-169.96(6)	S3	C1	S2	O23	-166.64(6)

Table S21: Hydrogen Atom Coordinates (Å×10^4) and Isotropic Displacement Parameters (Å^2×10^3) for $HC(SO_3H)_3$–II.

Atom	*x*	*y*	*z*	U(eq)
H11	4650(30)	659(6)	6373(12)	36(7)
H21	9314(3)	1640(30)	3204(10)	38(8)
H31	6441.6(14)	4960(20)	6130(20)	50(9)
H1	8090.2(15)	2448.1(9)	6399.1(13)	24(5)

$[H_3O]_3[HC(SO_3)_3]$ (4)

Table S22: Crystallographic data of $[H_3O]_3[HC(SO_3)_3]$ (**4**)

Empirical formula	$CH_{10}O_{12}S_3$
Formula weight	310.27 g/mol
Temperature	99 (2) K
Crystal system	Trigonal
Space group	*R*3*c* (No. 161)
Unit cell dimensions	*a* = 1322.13(4) pm<br>*b* = 1322.13(4) pm<br>*c* = 977.40(5) pm<br>α = 90<br>β = 90)°<br>γ = 120°
Volume	1479.6(1) $Å^3$
Z	6
ρ_{calc}	2.089 g/cm^3
μ	0.813 mm^{-1}
F(000)	960
Crystal size	0.297 × 0.207 × 0.142 mm^3
Radiation	MoK_α (λ = 0.71073 nm)
2Θ range for data collection	6.164 to 60.966
Index ranges	-18 ≤ h ≤ 18, -18 ≤ k ≤ 17, -13 ≤ l ≤ 13
Reflections collected	10309
Independent reflections	997 [R_{int} = 0.0383, R_σ = 0.0192]
Completeness	99.7%
Absorption correction	Multiscan
Min. and max. transmission	0.738 / 0.741
Data/restraints/parameters	997/1/62
Goodness-of-fit on F^2	1.084
Final *R* indexes [I≥2σ (I)]	R_1 = 0.0161, wR_2 = 0.0396
Final *R* indexes [all data]	R_1 = 0.0173, wR_2 = 0.0401
Largest diff. peak/hole	0.24/–0.20 e · $Å^{-3}$
CCDC-No.	2310267

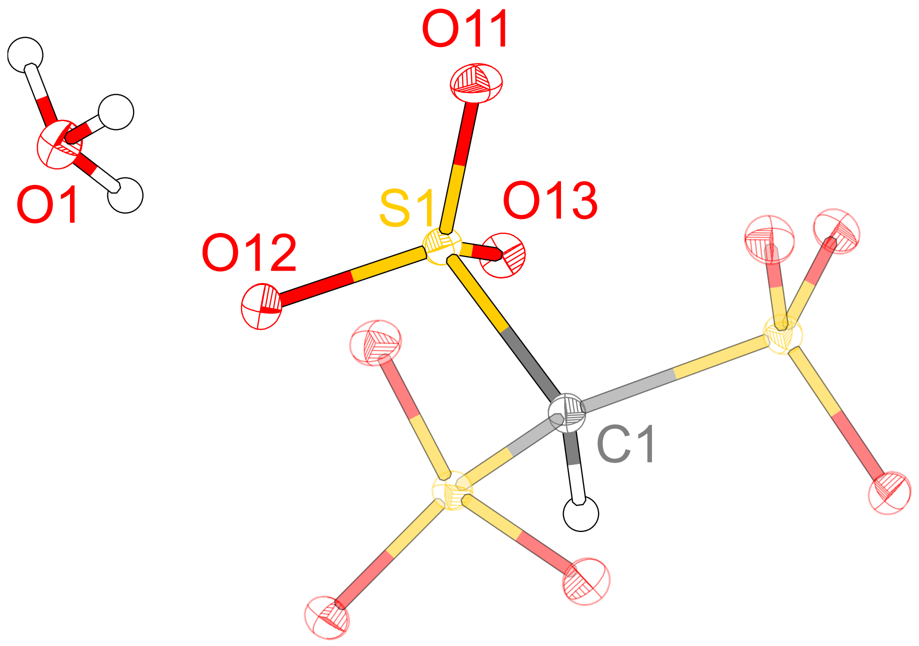

Figure S6: Thermal ellipsoid plot of the asymmetric unit $[H_3O]_3[HC(SO_3)_3]$. Thermal ellipsoids shown with 50% probability. Atoms generated due to symmetry for representation of the complete molecule are shown at 50% visibility.

Table S23: Fractional Atomic Coordinates (×10^4) and Equivalent Isotropic Displacement Parameters (Å^2×10^3) for $[H_3O]_3[HC(SO_3)_3]$. U_{eq} is defined as 1/3 of the trace of the orthogonalised U_{IJ} tensor.

Atom	*x*	*y*	*z*	U(eq)
S1	6574.9(3)	4592.5(3)	4506.6(4)	9.39(11)
O12	5339.3(10)	4240.3(10)	4344.3(11)	12.5(2)
O1	4134.4(11)	3819.7(11)	6506.6(13)	13.3(2)
O13	7278.7(10)	5509.8(10)	3504.9(11)	11.7(2)
O11	7006.7(12)	4852.7(11)	5881.9(12)	13.8(2)
C1	6666.67	3333.33	3933(3)	9.2(4)

Table S24: Anisotropic Displacement Parameters (Å^2×10^3) for $[H_3O]_3[HC(SO_3)_3]$. The anisotropic displacement factor exponent takes the form: $-2\pi^2[h^2a^{*2}U_{11}+2hka^*b^*U_{12}+\ldots]$.

Atom	U_{11}	U_{22}	U_{33}	U_{23}	U_{13}	U_{12}
S1	10.32(18)	9.54(18)	8.77(15)	-0.45(12)	-0.18(12)	5.32(15)
O12	11.4(5)	14.1(5)	13.3(5)	-0.5(4)	0.4(4)	7.3(4)
O1	13.8(6)	13.1(6)	14.3(5)	0.6(4)	1.6(4)	7.5(4)
O13	12.8(5)	10.9(5)	11.1(5)	1.3(4)	0.9(4)	5.8(4)
O11	17.4(6)	15.2(6)	9.5(5)	-2.2(4)	-1.8(4)	8.7(5)
C1	8.6(6)	8.6(6)	10.5(10)	0	0	4.3(3)

Table S25: Bond Lengths for $[H_3O]_3[HC(SO_3)_3]$ in [pm].

Atom	Atom	Length/pm	Atom	Atom	Length/pm
S1	O12	146.6(1)	S1	O11	143.3(1)
S1	O13	147.2(1)	S1	C1	181.72(9)

Table S26: Bond Angles for $[H_3O]_3[HC(SO_3)_3]$.

Atom	Atom	Atom	Angle/°	Atom	Atom	Atom	Angle/°
O12	S1	O13	109.55(7)	O11	S1	C1	107.94(9)
O12	S1	C1	104.47(5)	$S1^1$	C1	$S1^2$	110.94(7)
O13	S1	C1	104.21(8)	$S1^1$	C1	S1	110.94(7)
O11	S1	O12	115.12(7)	$S1^2$	C1	S1	110.94(7)
O11	S1	O13	114.46(8)				

[1]1+Y-X,1-X,+Z; [2]1-Y,+X-Y,+Z

Table S27: Torsion Angles for $[H_3O]_3[HC(SO_3)_3]$.

A	B	C	D	Angle/°	A	B	C	D	Angle/°
O12	S1	C1	$S1^1$	-163.50(9)	O13	S1	C1	$S1^2$	-154.66(10)
O12	S1	C1	$S1^2$	-39.71(14)	O11	S1	C1	$S1^1$	-40.52(12)
O13	S1	C1	$S1^1$	81.55(13)	O11	S1	C1	$S1^2$	83.27(11)

Table S28: Hydrogen Atom Coordinates (Å×10^4) and Isotropic Displacement Parameters (Å^2×10^3) for $[H_3O]_3[HC(SO_3)_3]$.

Atom	*x*	*y*	*z*	U(eq)
H1	6666.67	3333.33	2910.22	11
H1A	4290(30)	4540(30)	6750(40)	43(9)
H1B	4340(20)	3450(20)	7150(30)	17(6)
H1C	4540(30)	3990(30)	5790(50)	47(9)

Rb[$H_3C(SO_3)$](H_2O) (5)

Table S29: Crystallographic data of Rb[$H_3C(SO_3)$](H_2O).

Empirical formula	$C_2H_8O_7RbS_2$
Formula weight	379.14 g/mol
Temperature	100(2) K
Crystal system	Monoclinic
Space group	$P2_1/c$ (No. 14)
Unit cell dimensions	a = 606.22(4) pm<br>b = 1969.1(1) pm<br>c = 890.08(6) pm<br>β = 91.558(2)°
Volume	1062.1(1) Å^3
Z	4
ρ_{calc}	2.371 g/cm^3
μ	9.611 mm^{-1}
F(000)	728
Radiation	MoK_α (λ = 0.71073 nm)
Crystal size	0.297 × 0.207 × 0.142 mm^3
2Θ range for data collection	4.136 to 61.998
Index ranges	$-8 \leq h \leq 8$, $-28 \leq k \leq 28$, $-11 \leq l \leq 12$
Reflections collected	33318
Independent reflections	3377 [R_{int} = 0.0475, R_σ = 0.0203]
Completeness	99.6%
Absorption correction	multiscan
Min. and max. transmission	0.363 / 0.747
Data/restraints/parameters	3377/0/151
Goodness-of-fit on F^2	1.152
Final R indexes [I≥2σ (I)]	R_1 = 0.0152, wR_2 = 0.0385
Final R indexes [all data]	R_1 = 0.0155, wR_2 = 0.0387
Largest diff. peak/hole	0.57/–0.43 e · Å^{-3}
CCDC-No.	2305648

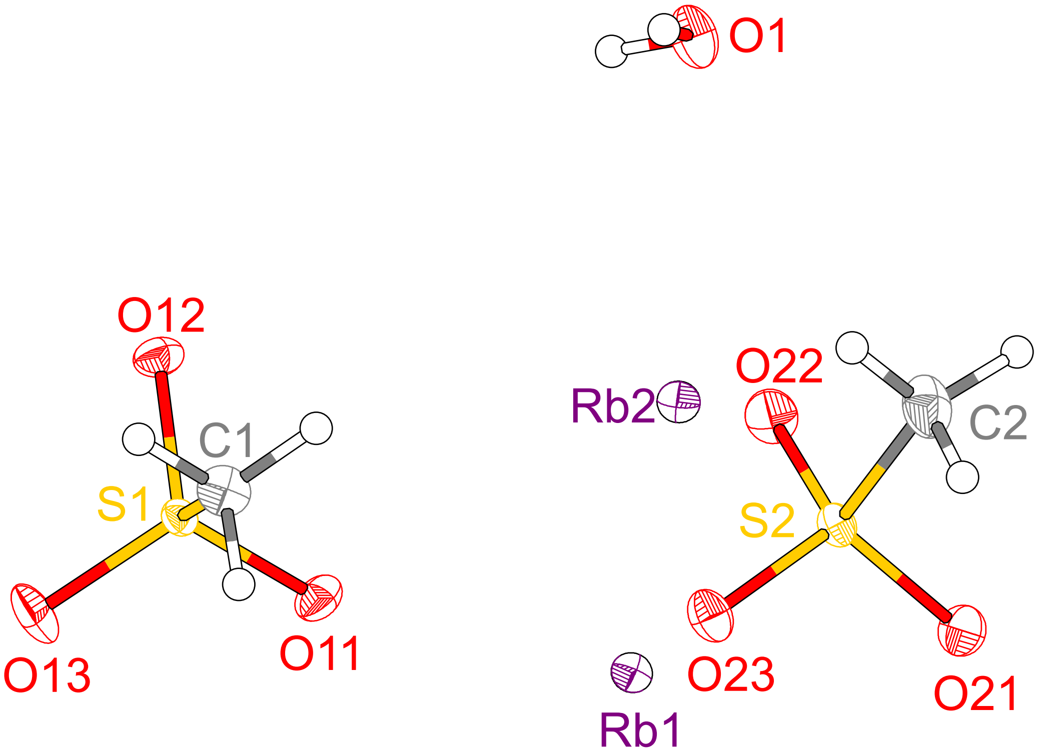

Figure S7: Thermal ellipsoid plot of the asymmetric unit of $Rb[H_3C(SO_3)](H_2O)$. Thermal ellipsoids shown with 50% probability.

Table S30: Fractional Atomic Coordinates (×10^4) and Equivalent Isotropic Displacement Parameters (Å^2×10^3) Rb[$H_3C(SO_3)$](H_2O). U_{eq} is defined as 1/3 of the trace of the orthogonalised U_{IJ} tensor.

Atom	*x*	*y*	*z*	U(eq)
Rb1	1699.3(2)	7399.7(2)	7696.0(2)	11.09(4)
Rb2	3548.2(2)	3951.5(2)	4278.7(2)	12.31(4)
S1	6699.4(4)	7888.6(2)	5035.6(3)	10.08(5)
S2	1492.7(5)	5378.0(2)	7359.4(3)	11.61(6)
O12	8655.2(15)	7452.8(5)	5132.5(10)	15.31(16)
O11	4679.5(14)	7501.4(5)	5261.4(10)	16.04(16)
O22	3100.3(16)	4934.1(5)	6684.4(11)	19.74(17)
O1	7871.6(18)	3805.1(5)	6484.6(12)	20.33(18)
O23	1428.9(16)	6048.3(4)	6636.9(10)	16.33(16)
O21	-709.6(15)	5085.4(5)	7447.3(11)	18.11(17)
O13	6594.1(18)	8288.8(5)	3662.4(10)	21.96(19)
C1	6930(2)	8460.6(6)	6558.3(14)	17.1(2)
C2	2413(2)	5526.8(8)	9225.1(15)	22.9(3)
Rb1	1699.3(2)	7399.7(2)	7696.0(2)	11.09(4)
Rb2	3548.2(2)	3951.5(2)	4278.7(2)	12.31(4)

Table S31: Anisotropic Displacement Parameters (Å^2×10^3) for Rb[$H_3C(SO_3)$](H_2O). The anisotropic displacement factor exponent takes the form: $-2\pi^2[h^2a^{*2}U_{11}+2hka^*b^*U_{12}+\ldots]$.

Atom	U_{11}	U_{22}	U_{33}	U_{23}	U_{13}	U_{12}
Rb1	1699.3(2)	7399.7(2)	7696.0(2)	11.09(4)	Rb1	1699.3(2)
Rb2	3548.2(2)	3951.5(2)	4278.7(2)	12.31(4)	Rb2	3548.2(2)
S1	6699.4(4)	7888.6(2)	5035.6(3)	10.08(5)	S1	6699.4(4)
S2	1492.7(5)	5378.0(2)	7359.4(3)	11.61(6)	S2	1492.7(5)
O12	8655.2(15)	7452.8(5)	5132.5(10)	15.31(16)	O12	8655.2(15)
O11	4679.5(14)	7501.4(5)	5261.4(10)	16.04(16)	O11	4679.5(14)
O22	3100.3(16)	4934.1(5)	6684.4(11)	19.74(17)	O22	3100.3(16)
O1	7871.6(18)	3805.1(5)	6484.6(12)	20.33(18)	O1	7871.6(18)
O23	1428.9(16)	6048.3(4)	6636.9(10)	16.33(16)	O23	1428.9(16)
O21	-709.6(15)	5085.4(5)	7447.3(11)	18.11(17)	O21	-709.6(15)
O13	6594.1(18)	8288.8(5)	3662.4(10)	21.96(19)	O13	6594.1(18)
C1	6930(2)	8460.6(6)	6558.3(14)	17.1(2)	C1	6930(2)
C2	2413(2)	5526.8(8)	9225.1(15)	22.9(3)	C2	2413(2)
Rb1	1699.3(2)	7399.7(2)	7696.0(2)	11.09(4)	Rb1	1699.3(2)
Rb2	3548.2(2)	3951.5(2)	4278.7(2)	12.31(4)	Rb2	3548.2(2)

Table S32: Bond Lengths and interatomic distances for Rb[$H_3C(SO_3)$](H_2O) in [pm].

Atom	Atom	Length/pm	Atom	Atom	Length/pm
Rb1	S1^{1}	376.69(3)	Rb2	O11^{5}	307.9(1)
Rb1	S1^{2}	391.54(3)	Rb2	O22	290.42(9)
Rb1	S1^{3}	367.47(3)	Rb2	O22^{5}	312.6(1)
Rb1	O12^{2}	289.71(9)	Rb2	O1	324.4(1)
Rb1	O12^{1}	290.02(9)	Rb2	O23^{5}	317.3(1)
Rb1	O11	286.58(9)	Rb2	O23^{6}	310.4(1)
Rb1	O11^{3}	287.95(9)	Rb2	O21^{6}	296.21(9)
Rb1	O1^{4}	287.2(1)	Rb2	O13^{7}	292.43(9)
Rb1	O23	282.63(9)	S1	O12	146.43(9)
Rb1	O13^{3}	335.3(1)	S1	O11	146.10(9)
Rb1	O13^{1}	350.7(1)	S1	O13	145.41(9)
Rb1	C1^{2}	368.6(1)	S1	C1	176.4(1)
Rb2	S1^{5}	367.81(4)	S2	O22	145.13(9)
Rb2	S2^{6}	360.08(3)	S2	O23	146.85(9)
Rb2	S2^{5}	362.57(3)	S2	O21	145.81(9)
Rb2	O12^{5}	312.1(1)	S2	C2	176.2(1)
Rb1	S1^{1}	376.69(3)	Rb2	O11^{5}	307.9(1)
Rb1	S1^{2}	391.54(3)	Rb2	O22	290.42(9)
Rb1	S1^{3}	367.47(3)	Rb2	O22^{5}	312.6(1)

^{1}-1+X,3/2-Y,1/2+Z; ^{2}-1+X,+Y,+Z; ^{3}+X,3/2-Y,1/2+Z; ^{4}1-X,1/2+Y,3/2-Z; ^{5}1-X,1-Y,1-Z; ^{6}-X,1-Y,1-Z; ^{7}1-X,-1/2+Y,1/2-Z

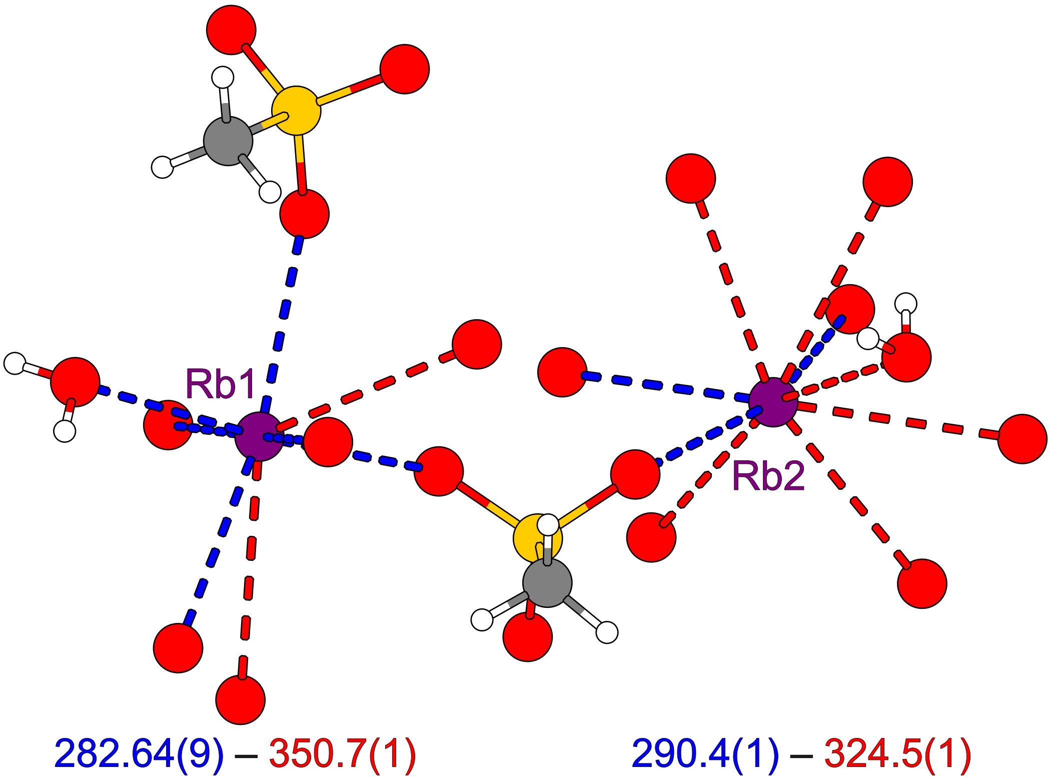

Figure S 8: Coordination environment around the cations within compound **5**. Red and blue dotted lines indicate interatomic coordination above and below 300 pm, respectively. The shortest and longest interatomic distances are given in [pm].

Table S33: : (Reduced) Bond Angles for Rb[$H_3C(SO_3)$](H_2O). Angles including Rubidium atoms were omitted for clarity.

Atom	Atom	Atom	Angle/°	Atom	Atom	Atom	Angle/°
O12	S1	C1	106.36(6)	O22	S2	C2	106.94(7)
O11	S1	O12	111.49(6)	O23	S2	C2	105.49(6)
O11	S1	C1	106.06(6)	O21	S2	O23	111.43(6)
O22	S2	O23	111.71(6)	O21	S2	C2	106.39(6)
O22	S2	O21	114.23(6)	O22	S2	C2	106.94(7)

Table S34: Torsion Angles for Rb[$H_3C(SO_3)$](H_2O).

A	B	C	D	Angle/°	A	B	C	D	Angle/°
Rb1^{1}	S1	O12	Rb1^{2}	-155.30(3)	O12	S1	O11	Rb2^{4}	-0.66(5)
Rb1^{2}	S1	O12	Rb1^{3}	-125.18(8)	O12	S1	O13	Rb1^{1}	121.47(5)
Rb1^{3}	S1	O12	Rb1^{2}	125.18(8)	O12	S1	O13	Rb1^{3}	-2.68(5)
Rb1^{1}	S1	O12	Rb1^{3}	79.52(5)	O12	S1	C1	Rb1^{2}	-7.34(5)
Rb1^{1}	S1	O12	Rb2^{4}	-50.78(5)	O11	S1	O12	Rb1^{3}	130.94(5)
Rb1^{3}	S1	O12	Rb2^{4}	-130.30(5)	O11	S1	O12	Rb1^{2}	-103.88(6)
Rb1^{2}	S1	O12	Rb2^{4}	104.53(6)	O11	S1	O12	Rb2^{4}	0.65(5)
Rb1^{1}	S1	O11	Rb1	-133.10(9)	O11	S1	O13	Rb1^{1}	-5.42(5)
Rb1^{3}	S1	O11	Rb1	152.57(4)	O11	S1	O13	Rb1^{3}	-129.57(4)
Rb1^{3}	S1	O11	Rb1^{1}	-74.32(6)	O11	S1	C1	Rb1^{2}	111.46(4)
Rb1^{2}	S1	O11	Rb1	65.27(7)	O22	S2	O23	Rb1	-128.44(7)
Rb1^{2}	S1	O11	Rb1^{1}	-161.62(2)	O22	S2	O23	Rb2^{4}	-29.77(6)
Rb1^{1}	S1	O11	Rb2^{4}	120.02(5)	O22	S2	O23	Rb2^{5}	123.06(5)
Rb1^{3}	S1	O11	Rb2^{4}	45.70(6)	O22	S2	O21	Rb2^{5}	-121.29(5)
Rb1^{2}	S1	O11	Rb2^{4}	-41.60(4)	O23	S2	O22	Rb2^{4}	30.43(6)
Rb1^{2}	S1	O13	Rb1^{3}	36.49(5)	O23	S2	O22	Rb2	-88.80(9)
Rb1^{1}	S1	O13	Rb1^{3}	-124.15(3)	O23	S2	O21	Rb2^{5}	6.48(6)
Rb1^{2}	S1	O13	Rb1^{1}	160.63(2)	O21	S2	O22	Rb2	38.83(10)
Rb1^{3}	S1	O13	Rb1^{1}	124.15(3)	O21	S2	O22	Rb2^{4}	158.06(4)
Rb1^{3}	S1	C1	Rb1^{2}	-53.56(4)	O21	S2	O23	Rb1	102.44(8)
Rb1^{1}	S1	C1	Rb1^{2}	159.46(2)	O21	S2	O23	Rb2^{4}	-158.89(4)
Rb1	S2	O22	Rb2^{4}	0.41(6)	O21	S2	O23	Rb2^{5}	-6.06(6)
Rb1	S2	O22	Rb2	-118.82(7)	O13	S1	O12	Rb1^{2}	128.76(6)
Rb1	S2	O23	Rb2^{5}	-108.50(8)	O13	S1	O12	Rb1^{3}	3.58(7)
Rb1	S2	O23	Rb2^{4}	98.67(7)	O13	S1	O12	Rb2^{4}	-126.71(5)
Rb1	S2	O21	Rb2^{5}	39.38(4)	O13	S1	O11	Rb1	-126.31(7)
Rb2^{4}	S1	O12	Rb1^{2}	-104.53(6)	O13	S1	O11	Rb1^{1}	6.79(7)
Rb2^{4}	S1	O12	Rb1^{3}	130.30(5)	O13	S1	O11	Rb2^{4}	126.81(5)
Rb2^{4}	S1	O11	Rb1	106.87(7)	O13	S1	C1	Rb1^{2}	-128.14(5)
Rb2^{4}	S1	O11	Rb1^{1}	-120.02(5)	C1	S1	O12	Rb1^{2}	11.30(8)
Rb2^{4}	S1	O13	Rb1^{3}	-67.36(5)	C1	S1	O12	Rb1^{3}	-113.88(6)
Rb2^{4}	S1	O13	Rb1^{1}	56.79(5)	C1	S1	O12	Rb2^{4}	115.82(5)
Rb2^{4}	S1	C1	Rb1^{2}	53.12(4)	C1	S1	O11	Rb1^{1}	123.96(5)
Rb2^{5}	S2	O22	Rb2	-22.17(11)	C1	S1	O11	Rb1	-9.15(9)
Rb2^{5}	S2	O22	Rb2^{4}	97.06(4)	C1	S1	O11	Rb2^{4}	-116.02(5)
Rb2^{4}	S2	O22	Rb2	-119.23(9)	C1	S1	O13	Rb1^{3}	114.12(5)
Rb2^{5}	S2	O23	Rb1	108.50(7)	C1	S1	O13	Rb1^{1}	-121.74(5)
Rb2^{4}	S2	O23	Rb1	-98.67(7)	C2	S2	O22	Rb2^{4}	-84.52(6)
Rb2^{5}	S2	O23	Rb2^{4}	-152.83(4)	C2	S2	O22	Rb2	156.25(8)
Rb2^{4}	S2	O23	Rb2^{5}	152.83(4)	C2	S2	O23	Rb1	-12.60(9)

Rb2[4]	S2	O21	Rb2[5]	-56.45(12)	C2	S2	O23	Rb2[5]	-121.10(6)
O12	S1	O11	Rb1	106.21(7)	C2	S2	O23	Rb2[4]	86.07(6)
O12	S1	O11	Rb1[1]	-120.69(5)	C2	S2	O21	Rb2[5]	120.97(6)

[1]+X,3/2-Y,-1/2+Z; [2]1+X,+Y,+Z; [3]1+X,3/2-Y,-1/2+Z; [4]1-X,1-Y,1-Z; [5]-X,1-Y,1-Z

Table S35: Hydrogen Atom Coordinates (Å×10^4) and Isotropic Displacement Parameters (Å^2×10^3) for Rb[$H_3C(SO_3)$](H_2O).

**Atom**	***x***	***y***	***z***	**U(eq)**
H1A	8270(30)	4156(11)	6800(20)	24(5)
H2A	2360(40)	5113(14)	9780(30)	47(7)
H1C	7020(30)	8209(10)	7450(20)	24(5)
H1B	8240(40)	3845(12)	5650(30)	35(6)
H1D	5780(40)	8734(11)	6560(20)	29(5)
H1E	8200(40)	8709(11)	6450(30)	37(6)
H2B	1420(30)	5836(11)	9630(20)	27(5)
H2C	3690(40)	5735(12)	9200(20)	33(6)

$Li_2[H_2C(SO_3)_2]$ (6)

Table S36: Crystallographic data of $Li_2[H_2C(SO_3)_2]$.

Empirical formula	$CH_2Li_2O_6S_2$
Formula weight	188.303 g/mol
Temperature	100(2) K
Crystal system	Orthorhombic
Space group	*Pnma* (No. 53)
Unit cell dimensions	a = 1352.00(8) pm<br>b = 806.87(6) pm<br>c = 489.87(3) pm
Volume	534.39(6) $Å^3$
Z	4
ρ_{calc}	2.337 g/cm^3
μ	0.955 mm^{-1}
F(000)	376
Radiation	MoK_α (λ = 0.71073 nm)
Crystal size	0.163 x 0.113 x 0.069 mm^3
2Θ range for data collection	6.026 to 63.018
Index ranges	$-19 \leq h \leq 18$, $-11 \leq k \leq 11$, $-7 \leq l \leq 7$
Reflections collected	9017
Independent reflections	914 [R_{int} = 0.0433, R_σ = 0.0243]
Completeness	99.6%
Absorption correction	multiscan
Min. and max. transmission	0.675 / 0.708
Data/restraints/parameters	914/0/62
Goodness-of-fit on F^2	1.136
Final R indexes [I≥2σ (I)]	R_1 = 0.0280, wR_2 = 0.0631
Final R indexes [all data]	R_1 = 0.0355, wR_2 = 0.0661
Largest diff. peak/hole	0.42/–0.53 e · $Å^{-3}$
CCDC-No.	2305645

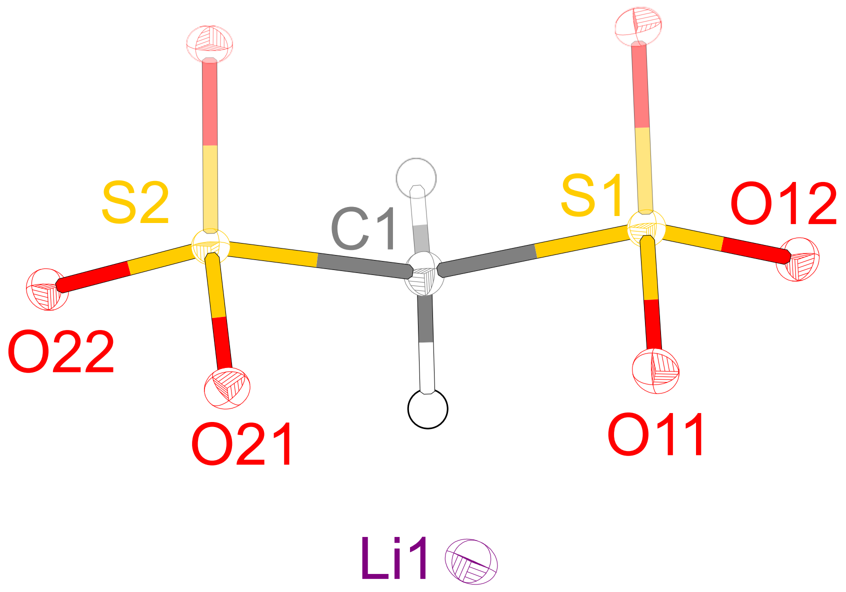

Figure S9: Thermal ellipsoid plot of the asymmetric unit of $Li_2[H_2C(SO_3)_2]$. Thermal ellipsoids shown with 50% probability. Atoms generated due to symmetry for representation of the complete molecule are shown at 50% visibility.

Table S37: Fractional Atomic Coordinates (×10^4) and Equivalent Isotropic Displacement Parameters (Å^2×10^3) for $Li_2[H_2C(SO_3)_2]$. U_{eq} is defined as 1/3 of the trace of the orthogonalised U_{IJ} tensor.

Atom	*x*	*y*	*z*	U(eq)
S1	2818.8(4)	2500	7778.8(11)	7.52(12)
S2	4981.3(4)	2500	8907.6(11)	7.00(12)
O11	2941.2(8)	994.9(15)	6193(2)	10.5(2)
O12	1912.2(12)	2500	9391(3)	9.6(3)
O22	5639.4(12)	2500	11268(3)	9.0(3)
O21	5070.3(8)	994.8(14)	7273(2)	8.9(2)
C1	3770.4(16)	2500	10292(5)	9.0(4)
Li1	3961(2)	-716(4)	5998(6)	12.6(6)

Table S38: Anisotropic Displacement Parameters (Å^2×10^3) for $Li_2[H_2C(SO_3)_2]$. The anisotropic displacement factor exponent takes the form: $-2\pi^2[h^2a^{*2}U_{11}+2hka^*b^*U_{12}+\ldots]$.

Atom	U_{11}	U_{22}	U_{33}	U_{23}	U_{13}	U_{12}
S1	7.2(2)	6.9(2)	8.4(2)	0	0.37(18)	0
S2	7.5(2)	6.4(2)	7.1(2)	0	-0.11(18)	0
O11	10.7(5)	8.5(5)	12.3(5)	-2.6(4)	-0.3(4)	1.4(4)
O12	7.8(7)	9.2(7)	11.7(8)	0	2.9(6)	0
O22	9.8(7)	8.6(7)	8.5(7)	0	-2.4(6)	0
O21	10.1(5)	7.5(5)	9.3(5)	-1.7(4)	0.6(4)	-0.1(4)
C1	8.0(9)	9.9(10)	9.2(9)	0	0.4(8)	0
Li1	14.0(13)	10.1(13)	13.8(13)	0.6(11)	-1.8(11)	1.6(11)

Table S39: Bond Lengths and interatomic distances for $Li_2[H_2C(SO_3)_2]$ in [pm].

Atom	Atom	Length/pm	Atom	Atom	Length/pm
S1	O11	145.1(1)	O12	Li1^2	202.1(3)
S1	O11^1	145.1(1)	O12	Li1^3	202.1(3)
S1	O12	145.8(2)	O22	Li1^4	203.9(3)
S1	C1	178.1(2)	O22	Li1^5	203.9(3)
S2	O22	145.9(2)	O21	Li1^6	208.2(3)
S2	O21	146.0(1)	O21	Li1	213.2(3)
S2	O21^1	146.0(1)	Li1	Li1^7	287.9(6)
S2	C1	177.2(2)	Li1	Li1^6	319.2(6)
O11	Li1	195.3(3)			

^{1}+X,1/2-Y,+Z; ^{2}1/2-X,-Y,1/2+Z; ^{3}1/2-X,1/2+Y,1/2+Z; ^{4}1-X,1/2+Y,2-Z; ^{5}1-X,-Y,2-Z; ^{6}1-X,-Y,1-Z; ^{7}+X,-1/2-Y,+Z

Table S40: Bond Angles for $Li_2[H_2C(SO_3)_2]$.

Atom	Atom	Atom	Angle/°	Atom	Atom	Atom	Angle/°
O11	S1	O11^1	113.64(10)	S2	C1	S1	113.75(13)
O11	S1	O12	112.69(6)	O11	Li1	O12^7	96.33(14)
O11^1	S1	O12	112.69(6)	O11	Li1	O22^5	130.82(17)
O11	S1	C1	106.72(6)	O11	Li1	O21	91.41(13)
O11^1	S1	C1	106.72(6)	O11	Li1	O21^6	123.94(16)
O12	S1	C1	103.46(11)	O11	Li1	Li1^8	134.97(10)
O22	S2	O21	112.61(6)	O11	Li1	Li1^6	112.37(17)
O22	S2	O21^1	112.61(6)	O12^7	Li1	O22^5	84.71(13)
O22	S2	C1	105.07(10)	O12^7	Li1	O21	170.44(18)
O21	S2	O21^1	112.62(10)	O12^7	Li1	O21^6	89.47(13)

O21	S2	C1	106.63(6)	O12^{7}	Li1	Li1^{8}	44.58(9)
O21^{1}	S2	C1	106.63(6)	O12^{7}	Li1	Li1^{6}	130.74(19)
S1	O11	Li1	134.29(12)	O22^{5}	Li1	O21	94.48(13)
S1	O12	Li1^{2}	134.57(9)	O22^{5}	Li1	O21^{6}	105.21(14)
S1	O12	Li1^{3}	134.57(9)	O22^{5}	Li1	Li1^{8}	45.09(9)
Li1^{2}	O12	Li1^{3}	90.84(18)	O22^{5}	Li1	Li1^{6}	102.90(15)
S2	O22	Li1^{4}	133.01(9)	O21^{6}	Li1	O21	81.54(12)
S2	O22	Li1^{5}	133.01(10)	O21	Li1	Li1^{8}	130.35(9)
Li1^{4}	O22	Li1^{5}	89.81(18)	O21^{6}	Li1	Li1^{6}	41.36(8)
S2	O21	Li1^{6}	124.33(11)	O21^{6}	Li1	Li1^{8}	83.79(9)
S2	O21	Li1	129.90(11)	O21	Li1	Li1^{6}	40.18(8)
Li1^{6}	O21	Li1	98.46(12)	Li1^{8}	Li1	Li1^{6}	111.22(11)

^{1}+X,1/2-Y,+Z; ^{2}1/2-X,-Y,1/2+Z; ^{3}1/2-X,1/2+Y,1/2+Z; ^{4}1-X,1/2+Y,2-Z; ^{5}1-X,-Y,2-Z; ^{6}1-X,-Y,1-Z; ^{7}1/2-X,-Y,-1/2+Z; ^{8}+X,-1/2-Y,+Z

Table S41: Torsion Angles for $Li_2[H_2C(SO_3)_2]$.

A	B	C	D	Angle/°	A	B	C	D	Angle/°
O11^{1}	S1	O11	Li1	108.19(16)	O21^{1}	S2	O22	Li1^{5}	40.8(2)
O11	S1	O12	Li1^{2}	25.8(2)	O21	S2	O22	Li1^{6}	-40.8(2)
O11	S1	O12	Li1^{3}	-156.01(17)	O21^{1}	S2	O21	Li1^{4}	37.96(16)
O11^{1}	S1	O12	Li1^{2}	156.01(17)	O21^{1}	S2	O21	Li1	-105.50(14)
O11^{1}	S1	O12	Li1^{3}	-25.8(2)	O21	S2	C1	S1	-60.27(6)
O11	S1	C1	S2	60.92(6)	O21^{1}	S2	C1	S1	60.27(6)
O11^{1}	S1	C1	S2	-60.92(6)	C1	S1	O11	Li1	-9.16(18)
O12	S1	O11	Li1	-122.06(17)	C1	S1	O12	Li1^{3}	89.11(18)
O12	S1	C1	S2	180.0	C1	S1	O12	Li1^{2}	-89.11(18)
O22	S2	O21	Li1	125.84(14)	C1	S2	O22	Li1^{6}	74.91(17)
O22	S2	O21	Li1^{4}	-90.71(14)	C1	S2	O22	Li1^{5}	-74.91(17)
O22	S2	C1	S1	180.000(0)	C1	S2	O21	Li1	11.11(16)
O21	S2	O22	Li1^{5}	169.43(16)	C1	S2	O21	Li1^{4}	154.57(13)
O21^{1}	S2	O22	Li1^{6}	-169.43(16)					

Table S42: Hydrogen Atom Coordinates (Å×10^4) and Isotropic Displacement Parameters (Å^2×10^3) for $Li_2[H_2C(SO_3)_2]$.

Atom	*x*	*y*	*z*	U(eq)
H1	3700(15)	1490(30)	11340(40)	14(5)

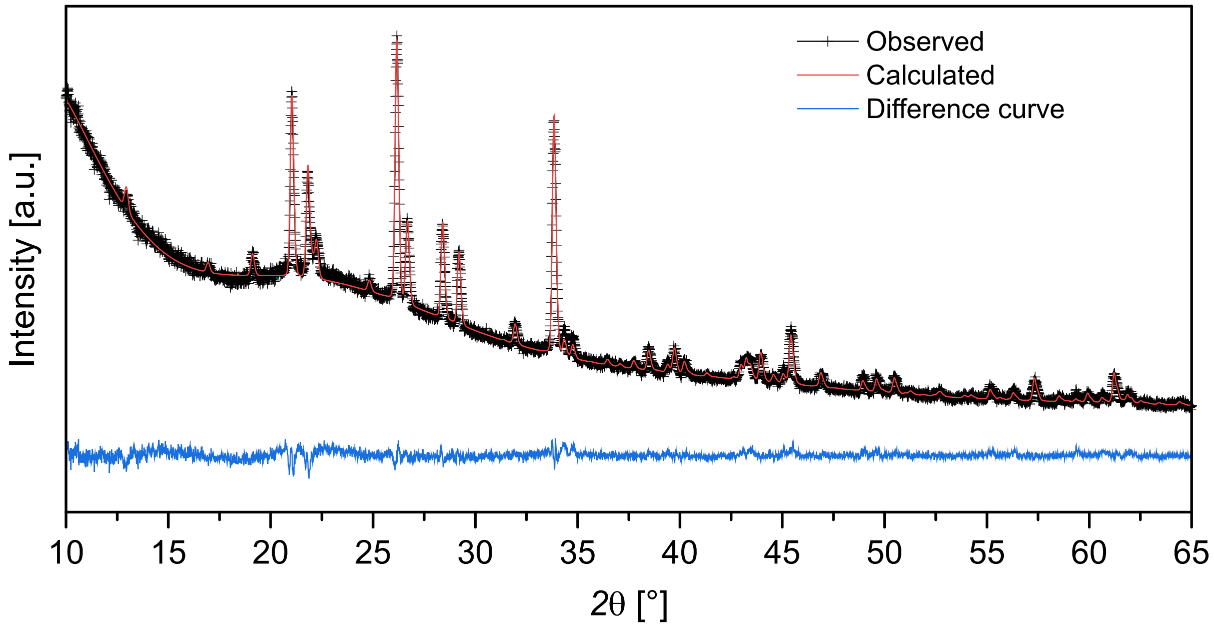

Figure S 10: X-ray powder diffraction pattern (black, cross) measured as a flat preparation with $CuK_{\alpha 1}$ radiation in Bragg-Brentano geometry, with Rietveld refinement (red) and difference curve (blue).

The final cell parameters after *Rietveld* refinement are *a* = 1358.03(8) pm, *b* = 811.58(4) pm, *c* = 491.77(2) pm, *V* = 542.15(4) Å^3 with *Rwp* = 3.139, *Rexp* = 1.845, Rp = 2.438 and *GooF* = 1.701.

$K_2[H_2C(SO_3)_2](H_2O)$ (7)

Table S43: Crystallographic data of $K_2[H_2C(SO_3)_2](H_2O)$.

Empirical formula	$CH_4K_2O_7S_2$
Formula weight	270.36 g/mol
Temperature	100(2) K
Crystal system	Orthorhombic
Space group	*Pnma* (No. 53)
	a = 815.5(1) pm
Unit cell dimensions	b = 1327.4(1) pm
	c = 728.00(8) pm
Volume	788.1(2) $Å^3$
Z	4
ρ_{calc}	2.279 g/cm^3
μ	1.729 mm^{-1}
F(000)	544
Radiation	MoK_α (λ = 0.71073 nm)
Crystal size	0.05 x 0.03 x 0.02 mm^3
2Θ range for data collection	6.138 to 57.38
Index ranges	$-11 \leq h \leq 11, -17 \leq k \leq 17, -9 \leq l \leq 9$
Reflections collected	10496
Independent reflections	1061 [R_{int} = 0.0257, R_σ = 0.0126]
Completeness	99.9%
Absorption correction	multiscan
Min. and max. transmission	0.641 / 0.746
Data/restraints/parameters	1061/0/64
Goodness-of-fit on F^2	1.151
Final R indexes [$I \geq 2\sigma$ (I)]	R_1 = 0.0410, wR_2 = 0.0950
Final R indexes [all data]	R_1 = 0.0529, wR_2 = 0.1085
Largest diff. peak/hole	0.71/–0.69 e · $Å^{-3}$
CCDC-No.	2517621

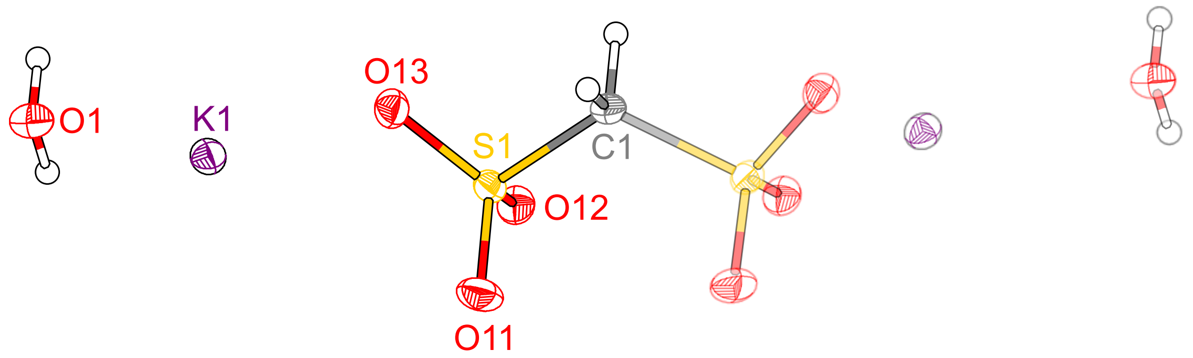

Figure S11: Thermal ellipsoid plot of the asymmetric unit of $K_2[H_2C(SO_3)_2](H_2O)$. Thermal ellipsoids shown with 50% probability. Atoms generated due to symmetry for representation of the complete molecule are shown at 50% visibility.

Table S44: Fractional Atomic Coordinates (×10^4) and Equivalent Isotropic Displacement Parameters (Å^2×10^3) for $K_2[H_2C(SO_3)_2](H_2O)$. U_{eq} is defined as 1/3 of the trace of the orthogonalised U_{IJ} tensor.

Atom	*x*	*y*	*z*	U(eq)
K1	3365.0(8)	4312.5(5)	1524.8(9)	18.4(2)
S1	5835.0(9)	6351.4(6)	3676.1(10)	15.6(2)
O1	5146(5)	2500	1811(5)	23.2(7)
O11	4302(3)	6368(2)	4698(3)	25.1(6)
O12	7272(3)	6312.5(17)	4866(3)	19.5(5)
O13	5872(3)	5585.4(17)	2248(3)	20.1(5)
C1	5923(5)	7500	2417(6)	16.0(8)

Table S45: Anisotropic Displacement Parameters (Å^2×10^3) for $K_2[H_2C(SO_3)_2](H_2O)$. The anisotropic displacement factor exponent takes the form: $-2\pi^2[h^2a^{*2}U_{11}+2hka^*b^*U_{12}+\ldots]$.

Atom	U_{11}	U_{22}	U_{33}	U_{23}	U_{13}	U_{12}
K1	20.2(4)	18.3(3)	16.5(3)	0.5(2)	-0.9(2)	-1.9(2)
S1	17.7(4)	14.9(4)	14.2(3)	1.4(3)	0.3(2)	-1.7(3)
O1	25.0(17)	27.2(18)	17.5(15)	0	3.5(14)	0
O11	22.0(12)	29.7(13)	23.5(12)	3.5(10)	9.2(9)	-1.1(10)
O12	23.8(11)	19.6(10)	15.2(10)	0.4(8)	-4.0(9)	-0.4(9)
O13	26.2(12)	16.7(10)	17.4(11)	-0.1(9)	-2.7(9)	-2.0(9)
C1	17.8(19)	17.7(19)	12.5(17)	0	0.7(15)	0

Table S46: Bond Lengths and interatomic distances for $K_2[H_2C(SO_3)_2](H_2O)$ in [pm].

Atom	Atom	Length/pm	Atom	Atom	Length/pm
K1	K1^1	4.3094(8)	S1	O12	1.459(2)
K1	K1^2	4.3177(6)	S1	O13	1.455(2)
S1	O11	1.455(2)	S1	C1	1.780(2)

^{1}1/2-X,1-Y,1/2+Z; ^{2}-1/2+X,+Y,1/2-Z

Table S47: Bond Angles for $K_2[H_2C(SO_3)_2](H_2O)$.

Atom	Atom	Atom	Angle/°	Atom	Atom	Atom	Angle/°
K1^1	K1	K1^2	54.06(2)	O13	S1	C1	103.28(15)
K1^3	S1	K1^4	75.039(19)	C1	S1	K1^3	129.34(13)
O11	S1	K1^4	70.66(11)	C1	S1	K1^4	133.64(13)
O11	S1	K1^3	123.69(11)	O1^5	O1	K1^5	0(10)
O11	S1	O12	112.79(15)	S1	O11	K1^1	159.57(16)
O11	S1	C1	106.53(17)	K1^4	O12	K1^3	96.33(7)
O12	S1	K1^4	44.08(10)	S1	O12	K1^4	114.71(13)
O12	S1	K1^3	60.69(9)	S1	O12	K1^3	94.02(11)
O12	S1	C1	107.68(17)	K1^3	O13	K1^6	100.39(8)
O13	S1	K1^3	53.23(9)	S1	O13	K1^3	102.09(11)
O13	S1	K1^4	120.67(10)	S1	O13	K1^6	131.86(13)
O13	S1	O11	113.23(15)	S1	C1	S1^7	117.8(2)
O13	S1	O12	112.52(14)				

^{1}1/2-X,1-Y,1/2+Z; ^{2}-1/2+X,+Y,1/2-Z; ^{3}1/2+X,+Y,1/2-Z; ^{4}1-X,1-Y,1-Z; ^{5}+X,1/2-Y,+Z; ^{6}1-X,1-Y,-Z; ^{7}+X,3/2-Y,+Z

Table S48: Torsion Angles for $K_2[H_2C(SO_3)_2](H_2O)$.

A	B	C	D	Angle/°	A	B	C	D	Angle/°
$K1^1$	S1	O11	$K1^2$	-17.3(5)	O12	S1	O11	$K1^2$	-86.3(5)
$K1^3$	S1	O11	$K1^2$	-73.1(4)	O12	S1	O13	$K1^1$	13.72(15)
$K1^1$	S1	O12	$K1^3$	-98.86(11)	O12	S1	O13	$K1^4$	-102.36(18)
$K1^3$	S1	O12	$K1^1$	98.86(11)	O12	S1	C1	$S1^5$	-62.8(3)
$K1^3$	S1	O13	$K1^4$	-151.19(11)	O13	S1	O11	$K1^2$	42.9(5)
$K1^1$	S1	O13	$K1^4$	-116.08(19)	O13	S1	O12	$K1^3$	-111.45(14)
$K1^3$	S1	O13	$K1^1$	-35.12(13)	O13	S1	O12	$K1^1$	-12.58(14)
$K1^3$	S1	C1	$S1^5$	-20.4(4)	O13	S1	C1	$S1^5$	177.9(2)
$K1^1$	S1	C1	$S1^5$	-129.06(18)	C1	S1	O11	$K1^2$	155.7(4)
O11	S1	O12	$K1^3$	18.15(18)	C1	S1	O12	$K1^3$	135.40(15)
O11	S1	O12	$K1^1$	117.01(13)	C1	S1	O12	$K1^1$	-125.74(14)
O11	S1	O13	$K1^4$	128.27(17)	C1	S1	O13	$K1^1$	129.55(15)
O11	S1	O13	$K1^1$	-115.65(13)	C1	S1	O13	$K1^4$	13.5(2)
O11	S1	C1	$S1^5$	58.4(3)					

Table S49: Hydrogen Atom Coordinates (Å×10^4) and Isotropic Displacement Parameters (Å^2×10^3) for $K_2[H_2C(SO_3)_2](H_2O)$.

Atom	*x*	*y*	*z*	U(eq)
H1C	5990(130)	2500	1210(150)	80(30)
H1D	5540(130)	2500	2970(160)	90(30)
H1A	5005.22	7500	1526.92	19
H1B	6953.1	7500	1697.63	19

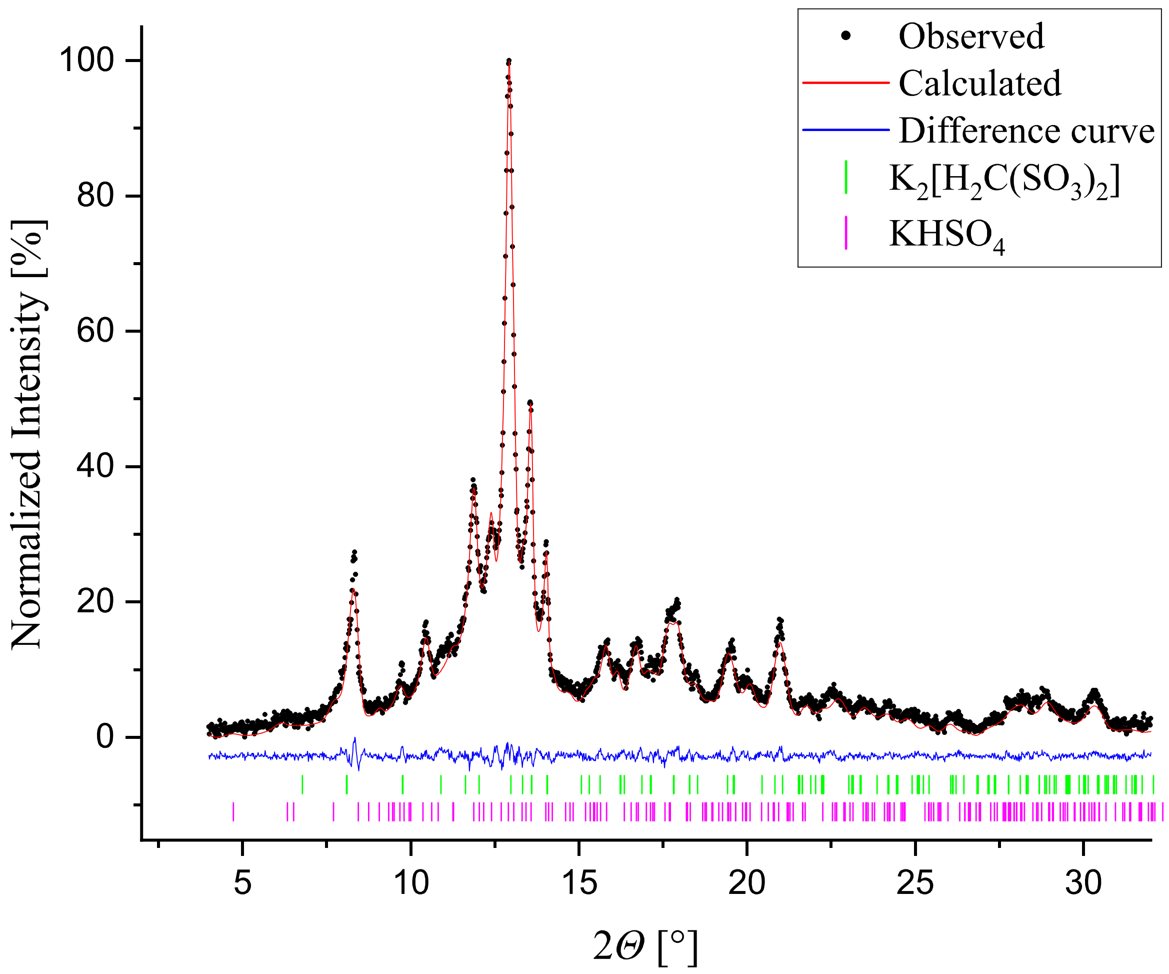

Figure S 12: Powder X-Ray Diffraction measurement of a dried sample of **7**. Observed reflexes, calculated pattern and the Bragg positions for the water free species $K_2[H_2C(SO_3)_2$ and $KHSO_4$ are shown as black dots, a red curve and green and magenta stripes, respectively. After the refinement, a product ratio of 69% **7** and 31% $KHSO_4$ was calculated by TOPAS.

$Rb_2[H_2C(SO_3)_2]$ (8)

Table S50: Crystallographic data of $Rb_2[H_2C(SO_3)_2]$.

Empirical formula	$CH_2O_6Rb_2S_2$
Formula weight	345.09 g/mol
Temperature	101(2) K
Crystal system	monoclinic
Space group	$C2/c$ (No. 15)
Unit cell dimensions	a = 1282.05(6) pm<br>b = 771.93(4) pm<br>c = 756.62(2) pm<br>β = 91.310(2)°
Volume	748.60(6) Å^3
Z	4
ρ_{calc}	3.062 g/cm^3
μ	13.610 mm^{-1}
F(000)	648
Radiation	MoK$_\alpha$ (λ = 0.71073 nm)
Crystal size	0.272 x 0.238 x 0.153 mm^3
2Θ range for data collection	6.162 to 54.996
Index ranges	$-16 \le h \le 16$, $-10 \le k \le 10$, $-9 \le l \le 9$
Reflections collected	8085
Independent reflections	852 [R_{int} = 0.0497, R_σ = 0.0383]
Completeness	99.6%
Absorption correction	multiscan
Min. and max. transmission	0.290 / 0.748
Data/restraints/parameters	852/0/56
Goodness-of-fit on F^2	1.136
Final R indexes [I≥2σ (I)]	R_1 = 0.0196, wR_2 = 0.0460
Final R indexes [all data]	R_1 = 0.0197, wR_2 = 0.0461
Largest diff. peak/hole	0.75/–0.54 e · Å^{-3}
CCDC-No.	2305644

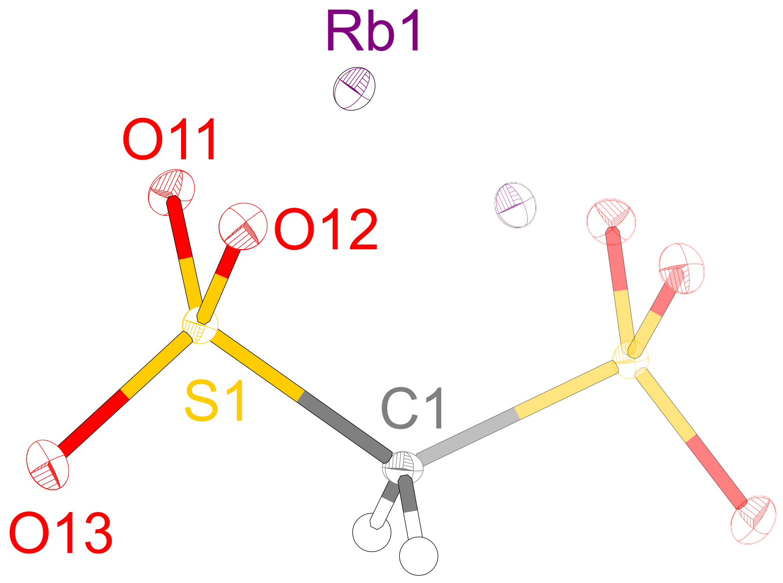

Figure S13: Thermal ellipsoid plot of the asymmetric unit of $Rb_2[H_2C(SO_3)_2]$. Thermal ellipsoids shown with 50% probability. Atoms generated due to symmetry for representation of the complete molecule are shown at 50% visibility.

Table S51: Fractional Atomic Coordinates (×10^4) and Equivalent Isotropic Displacement Parameters (Å^2×10^3) for $Rb_2[H_2C(SO_3)_2]$. U_{eq} is defined as 1/3 of the trace of the orthogonalised U_{IJ} tensor.

Atom	*x*	*y*	*z*	U(eq)
Rb1	6500.0(2)	8617.4(2)	3552.2(2)	8.58(11)
S1	6031.4(4)	6825.2(6)	8589.0(6)	6.34(13)
O12	6509.3(11)	7909.8(19)	7253.6(18)	10.0(3)
O11	5576.1(12)	7850.1(19)	10004.9(18)	9.9(3)
O13	6717.9(11)	5447(2)	9236(2)	11.9(3)
C1	5000	5667(3)	7500	7.5(5)

Table S52: Anisotropic Displacement Parameters (Å^2×10^3) for $Rb_2[H_2C(SO_3)_2]$. The anisotropic displacement factor exponent takes the form: $-2\pi^2[h^2a^{*2}U_{11}+2hka^*b^*U_{12}+\ldots]$.

Atom	U_{11}	U_{22}	U_{33}	U_{23}	U_{13}	U_{12}
Rb1	8.95(14)	10.07(14)	6.76(14)	1.24(5)	1.03(8)	0.48(6)
S1	6.6(2)	7.3(2)	5.1(2)	0.11(15)	0.90(16)	-0.16(15)
O12	11.0(6)	11.1(7)	8.0(6)	1.6(5)	2.7(5)	-2.0(5)
O11	11.3(6)	12.1(7)	6.5(6)	-3.7(5)	1.9(5)	0.8(5)
O13	10.5(7)	12.5(7)	12.7(7)	1.6(5)	-1.5(5)	2.4(5)
C1	9.7(12)	5.7(11)	7.1(12)	0	0.5(9)	0

Table S53: Bond lengths and interatomic distances for $Rb_2[H_2C(SO_3)_2]$ in [pm].

Atom	Atom	Length/pm	Atom	Atom	Length/pm
Rb1	Rb1^1	375.42(4)	Rb1	O11^4	296.9(1)
Rb1	S1^1	360.44(5)	Rb1	O13^5	319.1(1)
Rb1	S1^2	356.91(5)	Rb1	O13^6	313.7(1)
Rb1	O12	285.3(1)	Rb1	O13^1	322.7(2)
Rb1	O12^1	288.9(2)	S1	O12	145.8(1)
Rb1	O12^2	285.5(1)	S1	O11	146.4(1)
Rb1	O11^2	317.9(2)	S1	O13	145.8(2)
Rb1	O11^3	296.0(2)	S1	C1	178.3(1)

^{1}3/2-X,3/2-Y,1-Z; ^{2}+X,2-Y,-1/2+Z; ^{3}1-X,+Y,3/2-Z; ^{4}+X,+Y,-1+Z; ^{5}+X,1-Y,-1/2+Z; ^{6}3/2-X,1/2+Y,3/2-Z

Table S54: Bond Angles for $Rb_2[H_2C(SO_3)_2]$.

Atom	Atom	Atom	Angle/°	Atom	Atom	Atom	Angle/°
S1^1	Rb1	Rb1^1	67.899(9)	O11^4	Rb1	O13^1	74.18(4)
S1^2	Rb1	Rb1^1	124.140(10)	O11^2	Rb1	O13^6	145.01(4)
S1^2	Rb1	S1^1	104.277(12)	O11^4	Rb1	O13^6	88.97(4)
O12^2	Rb1	Rb1^1	128.62(3)	O13^6	Rb1	Rb1^1	52.95(3)
O12^1	Rb1	Rb1^1	48.76(3)	O13^1	Rb1	Rb1^1	90.00(3)
O12	Rb1	Rb1^1	49.59(3)	O13^5	Rb1	Rb1^1	54.27(3)
O12	Rb1	S1^1	116.04(3)	O13^5	Rb1	S1^1	69.28(3)
O12^1	Rb1	S1^2	123.61(3)	O13^1	Rb1	S1^1	23.84(3)
O12^2	Rb1	S1^2	22.97(3)	O13^1	Rb1	S1^2	84.62(3)
O12^1	Rb1	S1^1	22.70(3)	O13^6	Rb1	S1^2	169.11(3)
O12^2	Rb1	S1^1	85.62(3)	O13^5	Rb1	S1^2	70.90(3)
O12	Rb1	S1^2	100.25(3)	O13^6	Rb1	S1^1	84.56(3)
O12	Rb1	O12^2	121.17(4)	O13^5	Rb1	O11^2	72.66(4)
O12^2	Rb1	O12^1	107.45(3)	O13^6	Rb1	O13^1	105.39(4)
O12	Rb1	O12^1	98.35(4)	O13^5	Rb1	O13^1	74.34(3)
O12	Rb1	O11^3	65.37(4)	O13^5	Rb1	O13^6	107.22(3)

O12^{2}	Rb1	O11^{4}	83.16(4)	Rb1^{7}	S1	Rb1^{1}	86.575(11)
O12^{1}	Rb1	O11^{3}	143.36(4)	O12	S1	Rb1^{1}	49.89(6)
O12^{2}	Rb1	O11^{2}	47.01(4)	O12	S1	Rb1^{7}	49.84(6)
O12^{1}	Rb1	O11^{4}	93.66(4)	O12	S1	O11	112.19(9)
O12	Rb1	O11^{2}	79.52(4)	O12	S1	O13	113.07(9)
O12^{1}	Rb1	O11^{2}	139.55(4)	O12	S1	C1	106.67(7)
O12	Rb1	O11^{4}	147.34(4)	O11	S1	Rb1^{7}	62.81(6)
O12^{2}	Rb1	O11^{3}	109.01(4)	O11	S1	Rb1^{1}	138.51(6)
O12	Rb1	O13^{5}	64.90(4)	O11	S1	C1	107.78(7)
O12^{1}	Rb1	O13^{6}	63.80(4)	O13	S1	Rb1^{7}	128.42(6)
O12	Rb1	O13^{6}	69.77(4)	O13	S1	Rb1^{1}	63.41(6)
O12^{1}	Rb1	O13^{1}	46.47(4)	O13	S1	O11	113.35(9)
O12^{2}	Rb1	O13^{5}	75.65(4)	O13	S1	C1	102.98(10)
O12^{2}	Rb1	O13^{6}	167.93(4)	C1	S1	Rb1^{1}	113.23(3)
O12^{1}	Rb1	O13^{5}	70.13(4)	C1	S1	Rb1^{7}	127.89(8)
O12^{2}	Rb1	O13^{1}	63.66(4)	Rb1	O12	Rb1^{1}	81.65(4)
O12	Rb1	O13^{1}	134.22(4)	Rb1^{7}	O12	Rb1^{1}	117.80(5)
O11^{3}	Rb1	Rb1^{1}	107.50(3)	Rb1	O12	Rb1^{7}	99.09(4)
O11^{2}	Rb1	Rb1^{1}	116.57(3)	S1	O12	Rb1^{1}	107.40(7)
O11^{4}	Rb1	Rb1^{1}	133.55(3)	S1	O12	Rb1	142.92(8)
O11^{4}	Rb1	S1^{2}	98.05(3)	S1	O12	Rb1^{7}	107.19(7)
O11^{2}	Rb1	S1^{2}	24.18(3)	Rb1^{3}	O11	Rb1^{7}	107.53(4)
O11^{2}	Rb1	S1^{1}	125.07(3)	Rb1^{8}	O11	Rb1^{7}	89.85(4)
O11^{3}	Rb1	S1^{1}	162.38(3)	Rb1^{3}	O11	Rb1^{8}	88.29(4)
O11^{4}	Rb1	S1^{1}	84.89(3)	S1	O11	Rb1^{7}	93.01(7)
O11^{3}	Rb1	S1^{2}	92.38(3)	S1	O11	Rb1^{3}	139.04(8)
O11^{3}	Rb1	O11^{4}	87.13(4)	S1	O11	Rb1^{8}	127.73(8)
O11^{4}	Rb1	O11^{2}	109.86(4)	Rb1^{9}	O13	Rb1^{10}	72.78(3)
O11^{3}	Rb1	O11^{2}	72.47(4)	Rb1^{10}	O13	Rb1^{1}	100.07(4)
O11^{2}	Rb1	O13^{1}	108.00(4)	Rb1^{9}	O13	Rb1^{1}	86.08(4)
O11^{3}	Rb1	O13^{6}	79.60(4)	S1	O13	Rb1^{1}	92.75(7)
O11^{3}	Rb1	O13^{5}	122.92(4)	S1	O13	Rb1^{10}	127.81(8)
O11^{3}	Rb1	O13^{1}	160.41(4)	S1	O13	Rb1^{9}	159.09(9)
O11^{4}	Rb1	O13^{5}	147.45(4)	S1^{3}	C1	S1	119.78(15)

^{1}3/2-X,3/2-Y,1-Z; ^{2}+X,2-Y,-1/2+Z; ^{3}1-X,+Y,3/2-Z; ^{4}+X,+Y,-1+Z; ^{5}3/2-X,1/2+Y,3/2-Z; ^{6}+X,1-Y,-1/2+Z; ^{7}+X,2-Y,1/2+Z; ^{8}+X,+Y,1+Z; ^{9}3/2-X,-1/2+Y,3/2-Z; ^{10}+X,1-Y,1/2+Z

Table S55: Torsion Angles for $Rb_2[H_2C(SO_3)_2]$.

**A**	**B**	**C**	**D**	**Angle/°**	**A**	**B**	**C**	**D**	**Angle/°**
Rb1^{1}	S1	O12	Rb1	-133.15(17)	O11	S1	O12	Rb1^{1}	8.08(10)
Rb1^{2}	S1	O12	Rb1^{1}	-127.49(9)	O11	S1	O12	Rb1	-125.07(13)
Rb1^{2}	S1	O12	Rb1	99.35(15)	O11	S1	O12	Rb1^{2}	135.58(8)
Rb1^{1}	S1	O12	Rb1^{2}	127.49(9)	O11	S1	O13	Rb1^{5}	120.87(10)
Rb1^{1}	S1	O11	Rb1^{3}	121.38(13)	O11	S1	O13	Rb1^{2}	-134.13(7)
Rb1^{2}	S1	O11	Rb1^{4}	-45.17(14)	O11	S1	O13	Rb1^{6}	-47.9(3)
Rb1^{2}	S1	O11	Rb1^{1}	46.97(9)	O11	S1	C1	S1^{3}	61.96(7)
Rb1^{2}	S1	O11	Rb1^{3}	168.35(5)	O13	S1	O12	Rb1^{1}	-121.63(8)
Rb1^{1}	S1	O11	Rb1^{4}	-92.14(9)	O13	S1	O12	Rb1	105.21(14)
Rb1^{1}	S1	O13	Rb1^{2}	-61.16(7)	O13	S1	O12	Rb1^{2}	5.86(10)
Rb1^{2}	S1	O13	Rb1^{5}	-105.00(9)	O13	S1	O11	Rb1^{3}	-115.99(12)
Rb1^{1}	S1	O13	Rb1^{5}	-166.16(4)	O13	S1	O11	Rb1^{4}	30.49(13)
Rb1^{1}	S1	O13	Rb1^{6}	25.1(3)	O13	S1	O11	Rb1^{1}	122.63(7)
Rb1^{2}	S1	O13	Rb1^{6}	86.2(2)	O13	S1	C1	S1^{3}	-177.96(7)

Rb1[1]	S1	C1	S1[3]	-7.031(15)	C1	S1	O12	Rb1	-7.27(16)
Rb1[2]	S1	C1	S1[3]	-111.60(4)	C1	S1	O12	Rb1[2]	-106.62(8)
O12	S1	O11	Rb1[3]	114.44(12)	C1	S1	O12	Rb1[1]	125.88(8)
O12	S1	O11	Rb1[4]	-99.08(11)	C1	S1	O11	Rb1[4]	143.78(10)
O12	S1	O11	Rb1[1]	-6.94(8)	C1	S1	O11	Rb1[3]	-2.70(15)
O12	S1	O13	Rb1[2]	-5.01(9)	C1	S1	O11	Rb1[1]	-124.07(8)
O12	S1	O13	Rb1[5]	-110.01(10)	C1	S1	O13	Rb1[5]	4.71(10)
O12	S1	O13	Rb1[6]	81.2(2)	C1	S1	O13	Rb1[2]	109.71(3)
O12	S1	C1	S1[3]	-58.71(7)	C1	S1	O13	Rb1[6]	-164.1(2)

[1]+X,2-Y,1/2+Z; [2]3/2-X,3/2-Y,1-Z; [3]1-X,+Y,3/2-Z; [4]+X,+Y,1+Z; [5]+X,1-Y,1/2+Z; [6]3/2-X,-1/2+Y,3/2-Z

Table S56: Hydrogen Atom Coordinates (Å×10^4) and Isotropic Displacement Parameters (Å^2×10^3) for $Rb_2[H_2C(SO_3)_2]$.

**Atom**	***x***	***y***	***z***	**U(eq)**
H1	5250(20)	4970(40)	6700(40)	12(6)

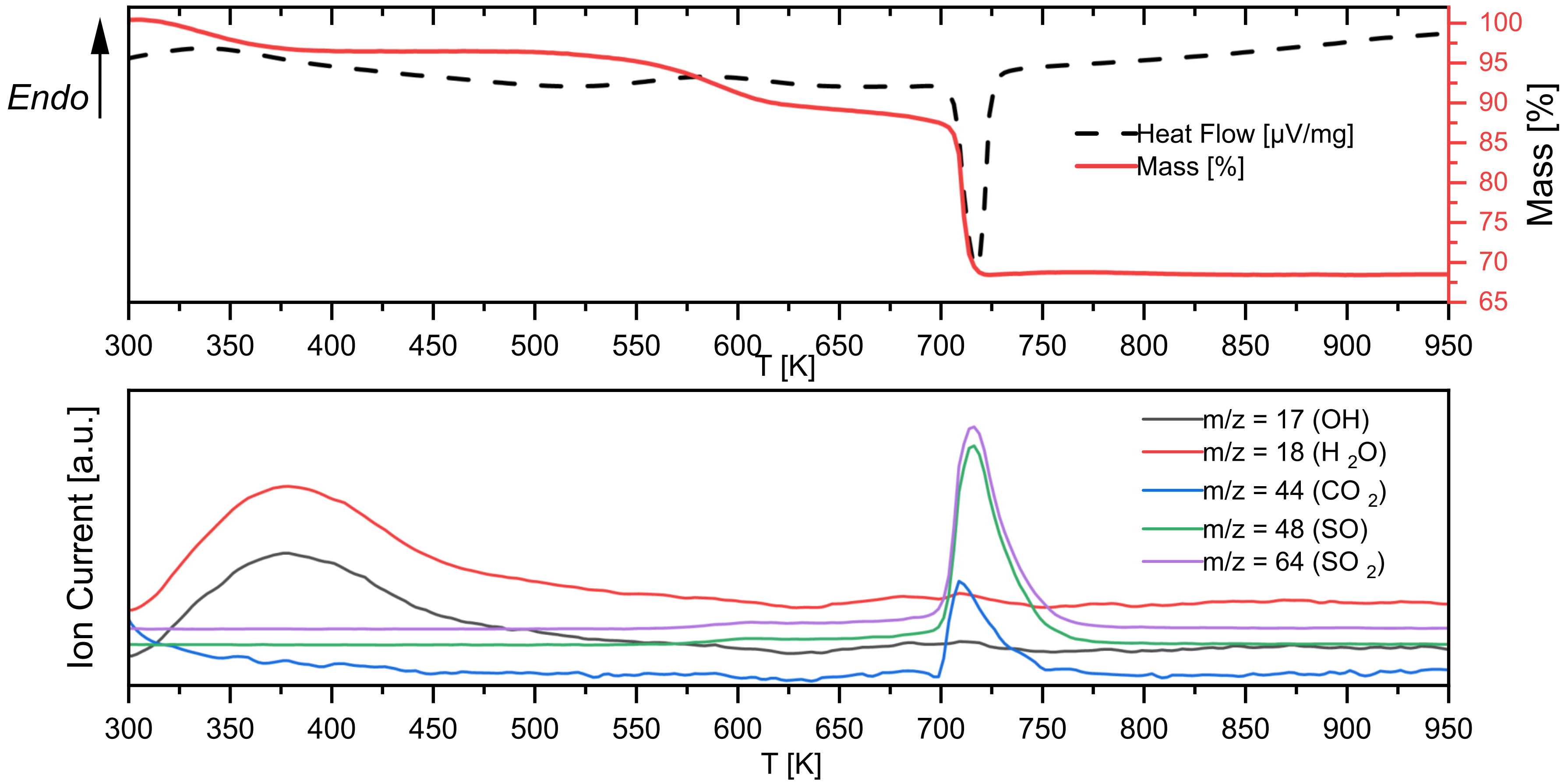

Figure S 14: DSC-TG/MS measurement of a sample of $Rb_2[H_2C(SO_3)_2]$,.heated with 5 K/min to a maximum temperature of 1260 K. The bottom part shows the detected masses of the decomposition fragments: (m/z = 17 (OH), m/z = 18 (H2O), m/z = 44 (CO2), m/z = 48 (SO), m/z = 64 (SO2). The mass los at about 575 K is not accompanied by a thermal signal or an observed fragment in the mass spectrum and probably an artefact.

.

$Sr[H_2C(SO_3)_2](H_2O)_2$ (9)

Table S57: Crystallographic data of $Sr[H_2C(SO_3)_2](H_2O)_2$.

Empirical formula	$CH_6O_8S_2Sr$
Formula weight	297.80 g/mol
Temperature	100(2) K
Crystal system	orthorhombic
Space group	*Cmce* (No. 64)
Unit cell dimensions	a = 1025.92(6) pm<br>b = 1659.19(6) pm<br>c = 910.16(4) pm
Volume	1549.3(1) Å^3
Z	8
ρ_{calc}	2.553 g/cm^3
μ	7.508 mm^{-1}
F(000)	1168
Radiation	MoK_α (λ = 0.71073 nm)
Crystal size	0.07 x 0.05 x 0.03 mm^3
2Θ range for data collection	4.91 to 52.73
Index ranges	$-12 \leq h \leq 10$, $-20 \leq k \leq 20$, $-11 \leq l \leq 11$
Reflections collected	6306
Independent reflections	841 [R_{int} = 0.0551, R_σ = 0.0319]
Completeness	99.7%
Absorption correction	multiscan
Min. and max. transmission	0.632 / 0.746
Data/restraints/parameters	841/0/64
Goodness-of-fit on F^2	1.106
Final R indexes [I≥2σ (I)]	R_1 = 0.0297, wR_2 = 0.0625
Final R indexes [all data]	R_1 = 0.0445, wR_2 = 0.0739
Largest diff. peak/hole	0.72/–0.75 e · Å^{-3}
CCDC-No.	2530504

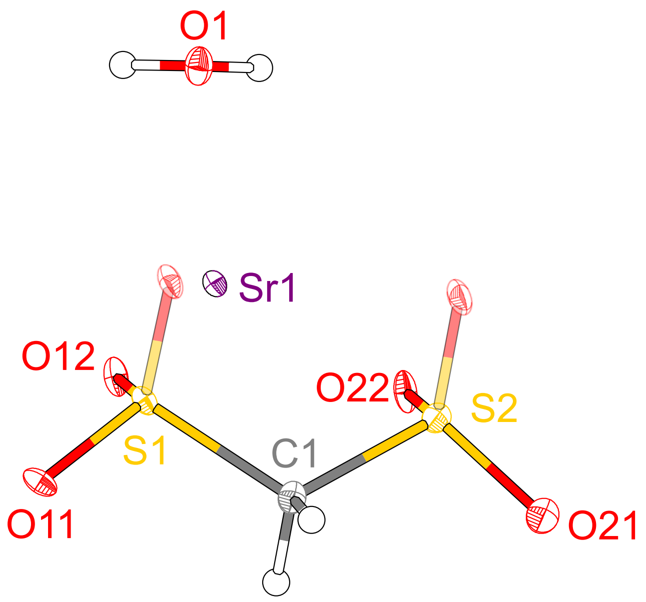

Figure S15: Thermal ellipsoid plot of the asymmetric unit of R $Sr[H_2C(SO_3)_2](H_2O)_2$. Thermal ellipsoids shown with 50% probability. Atoms generated due to symmetry for representation of the complete molecule are shown at 50% visibility.

Table S58: Fractional Atomic Coordinates (×10^4) and Equivalent Isotropic Displacement Parameters (Å^2×10^3) for $Sr[H_2C(SO_3)_2](H_2O)_2$. U_{eq} is defined as 1/3 of the trace of the orthogonalised U_{IJ} tensor.

Atom	*x*	*y*	*z*	U(eq)
Sr1	7500	7725.0(3)	7500	5.94(17)
S1	5000	6910.7(9)	4774.6(17)	6.9(3)
S2	5000	5953.2(9)	7572.7(18)	9.2(3)
O1	7947(3)	9030.5(18)	9023(3)	11.2(7)
O11	5000	6774(3)	3186(5)	9.9(9)
O12	6191(3)	7306.8(18)	5241(3)	9.0(7)
O21	5000	5104(3)	7973(5)	11.6(10)
O22	6185(3)	6368.5(18)	8026(3)	11.3(7)
C1	5000	5944(4)	5598(7)	9.9(13)

Table S59: Anisotropic Displacement Parameters (Å^2×10^3) for $Sr[H_2C(SO_3)_2](H_2O)_2$. The anisotropic displacement factor exponent takes the form: $-2\pi^2[h^2a^{*2}U_{11}+2hka^*b^*U_{12}+\ldots]$.

Atom	U_{11}	U_{22}	U_{33}	U_{23}	U_{13}	U_{12}
Sr1	4.8(3)	8.1(3)	4.9(3)	0	-0.5(2)	0
S1	5.2(7)	10.5(8)	5.2(7)	-0.2(6)	0	0
S2	9.1(7)	8.6(7)	10.0(8)	0.6(6)	0	0
O1	12.5(15)	12.5(16)	8.5(16)	2.3(13)	-2.5(13)	-2.2(13)
O11	10(2)	13(2)	6(2)	-4.1(18)	0	0
O12	6.0(14)	15.0(17)	6.2(15)	2.9(13)	-1.7(13)	-2.4(12)
O21	11(2)	12(2)	13(2)	1.5(18)	0	0
O22	12.3(16)	16.1(17)	5.6(15)	3.5(13)	-1.6(13)	-3.7(13)
C1	9(3)	10(3)	10(3)	2(3)	0	0

Table S60: Bond lengths and interatomic distances for $Sr[H_2C(SO_3)_2](H_2O)_2$ in [pm].

Atom	Atom	Length/pm	Atom	Atom	Length/pm
Sr1	S1^1	335.1(1)	S1	O11	146.4(5)
Sr1	O1	261.2(3)	S1	O12	145.1(3)
Sr1	O11^2	276.7(2)	S1	C1	177.0(6)
Sr1	O12	255.2(3)	S2	O21	145.5(5)
Sr1	O12^2	283.4(3)	S2	O22	145.7(3)
Sr1	O22^3	266.7(3)	S2	C1	179.7(7)

^{1}1-X,3/2-Y,1/2+Z; ^{2}3/2-X,3/2-Y,1-Z; ^{3}3/2-X,+Y,3/2-Z; ^{4}+X,3/2-Y,1/2+Z; ^{5}1-X,+Y,+Z

Table S61: Bond Angles for $Sr[H_2C(SO_3)_2](H_2O)_2$.

Atom	Atom	Atom	Angle/°	Atom	Atom	Atom	Angle/°
S1^1	Sr1	S1^2	159.22(6)	O12^3	Sr1	O12^1	117.05(10)
O1^3	Sr1	S1^2	92.52(7)	O12	Sr1	O22	69.45(9)
O1^3	Sr1	S1^1	69.93(7)	O12	Sr1	O22^3	83.80(9)
O1	Sr1	S1^2	69.93(7)	O12^3	Sr1	O22^3	69.46(9)
O1	Sr1	S1^1	92.52(7)	O12^3	Sr1	O22	83.80(9)
O1	Sr1	O1^3	67.98(14)	O22	Sr1	S1^2	69.77(7)
O1^3	Sr1	O11^2	73.02(11)	O22	Sr1	S1^1	130.56(7)
O1	Sr1	O11^1	73.02(11)	O22^3	Sr1	S1^2	130.56(8)
O1	Sr1	O11^2	78.11(11)	O22^3	Sr1	S1^1	69.77(7)
O1^3	Sr1	O11^1	78.11(11)	O22^3	Sr1	O11^2	139.69(11)
O1	Sr1	O12^4	68.37(9)	O22^3	Sr1	O11^1	75.18(11)
O1	Sr1	O12^1	113.55(9)	O22	Sr1	O11^1	139.69(11)

O1^3	Sr1	O12^1	68.37(9)	O22	Sr1	O11^2	75.18(11)
O1^3	Sr1	O12^4	113.55(9)	O22	Sr1	O12^1	112.46(9)
O1^3	Sr1	O22^3	133.88(10)	O22^3	Sr1	O12^1	65.58(9)
O1^3	Sr1	O22	134.92(9)	O22	Sr1	O12^4	65.58(9)
O1	Sr1	O22^3	134.92(9)	O22^3	Sr1	O12^4	112.46(9)
O1	Sr1	O22	133.88(10)	O22	Sr1	O22^3	64.91(14)
O11^1	Sr1	S1^1	25.47(9)	Sr1^5	S1	Sr1^1	99.88(4)
O11^1	Sr1	S1^2	142.61(9)	O11	S1	Sr1^5	54.38(6)
O11^2	Sr1	S1^1	142.61(9)	O11	S1	Sr1^1	54.38(6)
O11^2	Sr1	S1^2	25.47(9)	O11	S1	C1	106.1(3)
O11^1	Sr1	O11^2	145.05(17)	O12^6	S1	Sr1^1	138.04(14)
O11^2	Sr1	O12^4	50.79(11)	O12	S1	Sr1^5	138.04(14)
O11^1	Sr1	O12^4	130.04(11)	O12^6	S1	Sr1^5	56.95(12)
O11^1	Sr1	O12^1	50.79(11)	O12	S1	Sr1^1	56.95(12)
O11^2	Sr1	O12^1	130.04(11)	O12	S1	O11	111.06(16)
O12^3	Sr1	S1^2	87.37(7)	O12^6	S1	O11	111.05(16)
O12^3	Sr1	S1^1	98.28(7)	O12	S1	O12^6	114.7(3)
O12	Sr1	S1^1	87.37(7)	O12^6	S1	C1	106.64(17)
O12^4	Sr1	S1^1	155.50(6)	O12	S1	C1	106.64(17)
O12^4	Sr1	S1^2	25.41(6)	C1	S1	Sr1^1	115.15(12)
O12^1	Sr1	S1^2	155.50(6)	C1	S1	Sr1^5	115.15(12)
O12^1	Sr1	S1^1	25.41(6)	O21	S2	O22	112.76(16)
O12	Sr1	S1^2	98.28(7)	O21	S2	O22^6	112.76(16)
O12^3	Sr1	O1	72.87(9)	O21	S2	C1	104.0(3)
O12^3	Sr1	O1^3	138.20(10)	O22^6	S2	O22	113.1(3)
O12	Sr1	O1	138.20(10)	O22^6	S2	C1	106.69(17)
O12	Sr1	O1^3	72.87(9)	O22	S2	C1	106.69(17)
O12^3	Sr1	O11^1	77.03(11)	Sr1^1	O11	Sr1^5	135.88(17)
O12	Sr1	O11^2	77.03(11)	S1	O11	Sr1^5	100.15(11)
O12^3	Sr1	O11^2	112.82(11)	S1	O11	Sr1^1	100.15(11)
O12	Sr1	O11^1	112.82(11)	Sr1	O12	Sr1^1	117.70(11)
O12	Sr1	O12^4	117.05(10)	S1	O12	Sr1^1	97.64(15)
O12^4	Sr1	O12^1	177.86(12)	S1	O12	Sr1	143.34(18)
O12	Sr1	O12^1	62.29(11)	S2	O22	Sr1	140.36(19)
O12^3	Sr1	O12^4	62.29(11)	S1	C1	S2	114.6(4)
O12^3	Sr1	O12	148.44(13)				

^{1}3/2-X,3/2-Y,1-Z; ^{2}1-X,3/2-Y,1/2+Z; ^{3}3/2-X,+Y,3/2-Z; ^{4}+X,3/2-Y,1/2+Z; ^{5}1-X,3/2-Y,-1/2+Z; ^{6}1-X,+Y,+Z

Table S62: Torsion Angles for $Sr[H_2C(SO_3)_2](H_2O)_2$.

A	B	C	D	Angle/°	A	B	C	D	Angle/°
Sr1^1	S1	O11	Sr1^2	140.6(2)	O12^3	S1	O12	Sr1	62.4(4)
Sr1^2	S1	O11	Sr1^1	-140.6(2)	O12^3	S1	O12	Sr1^2	-132.62(17)
Sr1^1	S1	O12	Sr1^2	-65.3(2)	O12	S1	C1	S2	61.51(15)
Sr1^1	S1	O12	Sr1	129.7(2)	O12^3	S1	C1	S2	-61.51(15)
Sr1^2	S1	O12	Sr1	-165.0(4)	O21	S2	O22	Sr1	163.5(3)
Sr1^2	S1	C1	S2	122.27(9)	O21	S2	C1	S1	180.000(1)
Sr1^1	S1	C1	S2	-122.27(10)	O22^3	S2	O22	Sr1	-67.1(4)
O11	S1	O12	Sr1^2	-5.7(2)	O22^3	S2	C1	S1	60.58(15)
O11	S1	O12	Sr1	-170.7(3)	O22	S2	C1	S1	-60.58(15)
O11	S1	C1	S2	180.000(1)	C1	S1	O11	Sr1^1	109.69(12)
O12	S1	O11	Sr1^2	5.8(2)	C1	S1	O11	Sr1^2	-109.69(12)
O12^3	S1	O11	Sr1^1	-5.8(2)	C1	S1	O12	Sr1	-55.4(4)
O12^3	S1	O11	Sr1^2	134.78(15)	C1	S1	O12	Sr1^2	109.6(2)

O12	S1	O11	Sr1[1]	-134.78(15)	C1	S2	O22	Sr1	49.9(4)

[1]1-X,3/2-Y,-1/2+Z; [2]3/2-X,3/2-Y,1-Z; [3]1-X,+Y,+Z

Table S63: Hydrogen Atom Coordinates (Å×10^4) and Isotropic Displacement Parameters (Å^2×10^3) for $Sr[H_2C(SO_3)_2](H_2O)_2$.

**Atom**	***x***	***y***	***z***	**U(eq)**
H1A	8479.25	9341.49	8538.36	17
H1B	8371.63	8905.72	9820.77	17
H1C	5778.58	5646.14	5255.39	12
H1D	4221.42	5646.13	5255.39	12

$Cs_2[H_2C(SO_3)_2]$ (10)

Table S64: Crystallographic data of $Cs_2[H_2C(SO_3)_2]$.

Empirical formula	$CH_2O_6Cs_2S_2$
Formula weight	439.97 g/mol
Temperature	101(2) K
Crystal system	monoclinic
Space group	$C2/c$ (No. 15)
Unit cell dimensions	a = 1331.45(7) pm
	b = 798.52(4) pm
	c = 786.61(4) pm
	β = 91.791(2)°
Volume	835.91(7) Å^3
Z	4
ρ_{calc}	3.496 g/cm^3
μ	9.203 mm^{-1}
F(000)	792
Radiation	MoK$_\alpha$ (λ = 0.71073 nm)
Crystal size	0.329 x 0.157 x 0.1 mm^3
2Θ range for data collection	5.95 to 54.988
Index ranges	$-17 \leq h \leq 17$, $-10 \leq k \leq 10$, $-10 \leq l \leq 10$
Reflections collected	28440
Independent reflections	963 [R_{int} = 0.0631, R_σ = 0.0148]
Completeness	99.9%
Absorption correction	multiscan
Min. and max. transmission	0.340 / 0.746
Data/restraints/parameters	963/0/56
Goodness-of-fit on F^2	1.114
Final R indexes [I≥2σ (I)]	R_1 = 0.0134, wR_2 = 0.0350
Final R indexes [all data]	R_1 = 0.0152, wR_2 = 0.0355
Largest diff. peak/hole	0.61/–0.60 e · Å^{-3}
CCDC-No.	2305647

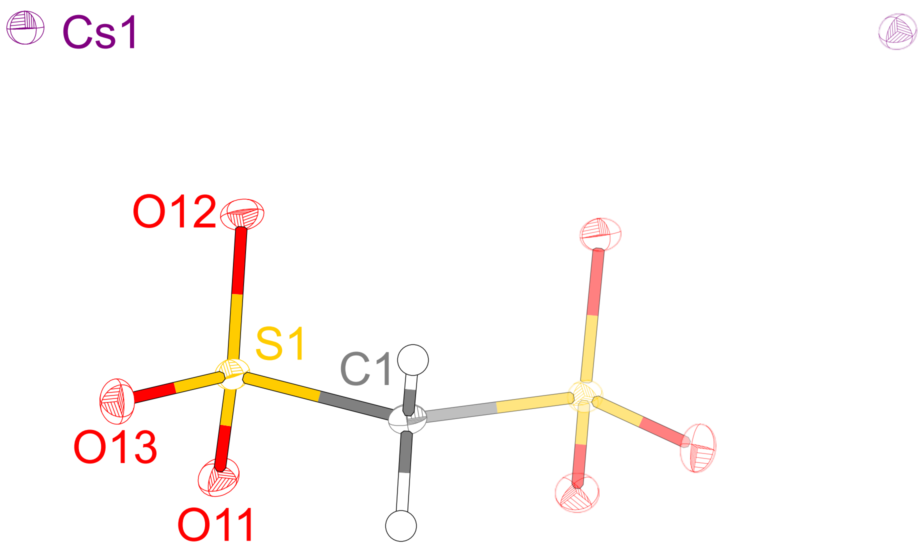

Figure S16: Thermal ellipsoid plot of the asymmetric unit of $Cs_2[H_2C(SO_3)_2]$. Thermal ellipsoids shown with 50% probability. Atoms generated due to symmetry for representation of the complete molecule are shown at 50% visibility.

Table S65: Fractional Atomic Coordinates (×10^4) and Equivalent Isotropic Displacement Parameters (Å^2×10^3) for $Cs_2[H_2C(SO_3)_2]$. U_{eq} is defined as 1/3 of the trace of the orthogonalised U_{IJ} tensor.

Atom	*x*	*y*	*z*	U(eq)
Cs1	3511.1(2)	8633.5(2)	6487.0(2)	10.63(8)
S1	4001.4(5)	6736.1(7)	1450.9(7)	9.23(14)
O12	3551.2(13)	7786(2)	2732(2)	12.7(4)
O11	4437.2(13)	7713(2)	96(2)	13.3(4)
O13	3334.8(14)	5419(2)	824(2)	14.2(4)
C1	5000	5604(4)	2500	9.8(6)

Table S66: Anisotropic Displacement Parameters (Å^2×10^3) for $Cs_2[H_2C(SO_3)_2]$. The anisotropic displacement factor exponent takes the form: $-2\pi^2[h^2a^{*2}U_{11}+2hka^*b^*U_{12}+\ldots]$.

Atom	U_{11}	U_{22}	U_{33}	U_{23}	U_{13}	U_{12}
Cs1	10.13(11)	11.73(11)	10.12(11)	-1.24(5)	1.46(6)	-0.25(5)
S1	8.6(3)	10.0(3)	9.1(3)	0.0(2)	1.5(2)	0.5(2)
O12	12.8(9)	13.5(8)	12.1(8)	-2.0(7)	3.2(7)	3.3(7)
O11	10.8(9)	17.1(9)	12.2(8)	3.5(7)	2.8(7)	0.2(7)
O13	11.7(9)	14.9(8)	15.8(9)	-2.5(7)	-3.2(7)	-0.7(7)
C1	9.8(17)	10.2(14)	9.6(15)	0	2.8(13)	0

Table S67: Bond lengths and interatomic distances for $Cs_2[H_2C(SO_3)_2]$ in [pm].

Atom	Atom	Length/pm	Atom	Atom	Length/pm
Cs1	Cs1^1	3.9504(3)	Cs1	O11^4	3.1466(16)
Cs1	S1^1	3.7747(7)	Cs1	O13^5	3.3312(17)
Cs1	S1^2	3.7549(6)	Cs1	O13^6	3.2849(18)
Cs1	O12^2	3.0218(17)	Cs1	O13^1	3.3784(19)
Cs1	O12^1	3.0509(18)	S1	O12	1.4547(17)
Cs1	O12	3.0322(16)	S1	O11	1.4560(17)
Cs1	O11^2	3.3636(18)	S1	O13	1.4527(18)
Cs1	O11^3	3.1250(18)	S1	C1	1.7883(18)

^{1}1/2-X,3/2-Y,1-Z; ^{2}+X,2-Y,1/2+Z; ^{3}1-X,+Y,1/2-Z; ^{4}+X,+Y,1+Z; ^{5}1/2-X,1/2+Y,1/2-Z; ^{6}+X,1-Y,1/2+Z

Table S68: Bond Angles for $Cs_2[H_2C(SO_3)_2]$.

Atom	Atom	Atom	Angle/°	Atom	Atom	Atom	Angle/°
S1^1	Cs1	Cs1^2	124.209(10)	O11^3	Cs1	O13^5	86.40(4)
S1^2	Cs1	Cs1^2	67.780(10)	O11^1	Cs1	O13^2	106.90(4)
S1^1	Cs1	S1^2	103.700(14)	O13^6	Cs1	Cs1^2	52.80(3)
O12^2	Cs1	Cs1^2	49.30(3)	O13^5	Cs1	Cs1^2	53.88(3)
O12^1	Cs1	Cs1^2	129.07(3)	O13^2	Cs1	Cs1^2	88.72(3)
O12	Cs1	Cs1^2	49.71(3)	O13^2	Cs1	S1^1	85.08(3)
O12^1	Cs1	S1^2	86.65(3)	O13^5	Cs1	S1^1	168.63(3)
O12^2	Cs1	S1^1	121.68(3)	O13^6	Cs1	S1^1	72.54(3)
O12	Cs1	S1^2	116.36(3)	O13^2	Cs1	S1^2	22.57(3)
O12	Cs1	S1^1	101.77(3)	O13^6	Cs1	S1^2	67.62(3)
O12^2	Cs1	S1^2	21.43(3)	O13^5	Cs1	S1^2	86.06(3)
O12^1	Cs1	S1^1	21.50(3)	O13^6	Cs1	O11^1	73.92(4)
O12^1	Cs1	O12	121.74(4)	O13^5	Cs1	O11^1	145.88(4)
O12^1	Cs1	O12^2	107.05(3)	O13^5	Cs1	O13^6	106.68(4)
O12	Cs1	O12^2	99.01(4)	O13^5	Cs1	O13^2	105.69(4)
O12^1	Cs1	O11^3	85.77(5)	O13^6	Cs1	O13^2	72.96(4)

O12	Cs1	O11^{4}	61.13(4)	Cs1^{7}	S1	Cs1^{2}	85.248(13)
O12^{2}	Cs1	O11^{3}	93.51(4)	O12	S1	Cs1^{2}	50.02(7)
O12^{2}	Cs1	O11^{1}	136.82(4)	O12	S1	Cs1^{7}	49.57(7)
O12^{1}	Cs1	O11^{1}	44.10(4)	O12	S1	O11	112.37(10)
O12^{1}	Cs1	O11^{4}	110.18(5)	O12	S1	C1	106.76(8)
O12	Cs1	O11^{1}	81.89(4)	O11	S1	Cs1^{7}	63.34(7)
O12^{2}	Cs1	O11^{4}	142.77(4)	O11	S1	Cs1^{2}	137.34(7)
O12	Cs1	O11^{3}	143.93(5)	O11	S1	C1	107.52(9)
O12	Cs1	O13^{5}	68.21(5)	O13	S1	Cs1^{7}	127.67(8)
O12^{2}	Cs1	O13^{5}	66.77(5)	O13	S1	Cs1^{2}	63.20(8)
O12	Cs1	O13^{6}	66.37(4)	O13	S1	O12	113.09(11)
O12^{1}	Cs1	O13^{6}	77.13(4)	O13	S1	O11	113.11(10)
O12^{1}	Cs1	O13^{2}	65.85(4)	O13	S1	C1	103.19(12)
O12^{2}	Cs1	O13^{2}	43.96(4)	C1	S1	Cs1^{2}	114.73(4)
O12^{2}	Cs1	O13^{6}	67.39(5)	C1	S1	Cs1^{7}	128.42(9)
O12^{1}	Cs1	O13^{5}	169.69(4)	Cs1	O12	Cs1^{2}	80.99(4)
O12	Cs1	O13^{2}	134.05(4)	Cs1^{7}	O12	Cs1^{2}	114.20(5)
O11^{4}	Cs1	Cs1^{2}	104.14(3)	Cs1^{7}	O12	Cs1	95.97(5)
O11^{1}	Cs1	Cs1^{2}	117.06(3)	S1	O12	Cs1^{7}	108.93(8)
O11^{3}	Cs1	Cs1^{2}	131.90(3)	S1	O12	Cs1	145.45(9)
O11^{3}	Cs1	S1^{1}	100.00(3)	S1	O12	Cs1^{2}	108.55(9)
O11^{1}	Cs1	S1^{2}	123.12(3)	Cs1^{4}	O11	Cs1^{8}	84.74(4)
O11^{1}	Cs1	S1^{1}	22.76(3)	Cs1^{4}	O11	Cs1^{7}	105.04(5)
O11^{3}	Cs1	S1^{2}	85.50(3)	Cs1^{8}	O11	Cs1^{7}	87.34(4)
O11^{4}	Cs1	S1^{2}	161.86(3)	S1	O11	Cs1^{4}	142.29(10)
O11^{4}	Cs1	S1^{1}	94.26(3)	S1	O11	Cs1^{7}	93.90(8)
O11^{4}	Cs1	O11^{3}	88.95(5)	S1	O11	Cs1^{8}	129.18(9)
O11^{3}	Cs1	O11^{1}	111.03(5)	Cs1^{9}	O13	Cs1^{10}	73.32(4)
O11^{4}	Cs1	O11^{1}	74.96(5)	Cs1^{9}	O13	Cs1^{2}	99.84(5)
O11^{4}	Cs1	O13^{6}	121.50(4)	Cs1^{10}	O13	Cs1^{2}	84.19(4)
O11^{3}	Cs1	O13^{2}	76.09(4)	S1	O13	Cs1^{2}	94.23(8)
O11^{4}	Cs1	O13^{2}	164.64(4)	S1	O13	Cs1^{10}	158.08(10)
O11^{3}	Cs1	O13^{6}	148.63(5)	S1	O13	Cs1^{9}	128.28(9)
O11^{4}	Cs1	O13^{5}	76.35(5)	S1	C1	S1^{4}	119.25(19)

^{1}+X,2-Y,1/2+Z; ^{2}1/2-X,3/2-Y,1-Z; ^{3}+X,+Y,1+Z; ^{4}1-X,+Y,1/2-Z; ^{5}+X,1-Y,1/2+Z; ^{6}1/2-X,1/2+Y,1/2-Z; ^{7}+X,2-Y,-1/2+Z; ^{8}+X,+Y,-1+Z; ^{9}+X,1-Y,-1/2+Z; ^{10}1/2-X,-1/2+Y,1/2-Z

Table S69: Torsion Angles for $Cs_2[H_2C(SO_3)_2]$.

A	B	C	D	Angle/°	A	B	C	D	Angle/°
Cs1^{2}	S1	O12	Cs1^{1}	124.90(10)	O11	S1	O13	Cs1^{5}	-47.9(3)
Cs1^{2}	S1	O11	Cs1^{3}	120.88(16)	O11	S1	O13	Cs1^{1}	-132.85(8)
Cs1^{1}	S1	O11	Cs1^{4}	-42.69(16)	O11	S1	O13	Cs1^{6}	121.08(12)
Cs1^{1}	S1	O11	Cs1^{2}	47.04(10)	O11	S1	C1	S1^{3}	62.22(8)
Cs1^{1}	S1	O11	Cs1^{3}	167.92(7)	O13	S1	O12	Cs1^{1}	4.28(12)
Cs1^{2}	S1	O11	Cs1^{4}	-89.74(10)	O13	S1	O12	Cs1	105.59(18)
Cs1^{2}	S1	O13	Cs1^{1}	-59.52(8)	O13	S1	O12	Cs1^{2}	-120.63(10)
Cs1^{1}	S1	O13	Cs1^{5}	84.9(3)	O13	S1	O11	Cs1^{3}	-117.17(16)
Cs1^{2}	S1	O13	Cs1^{5}	25.4(3)	O13	S1	O11	Cs1^{2}	121.95(9)
Cs1^{2}	S1	O13	Cs1^{6}	-165.59(5)	O13	S1	O11	Cs1^{4}	32.21(15)
Cs1^{1}	S1	O13	Cs1^{6}	-106.07(11)	O13	S1	C1	S1^{3}	-178.00(8)
Cs1^{2}	S1	C1	S1^{3}	-7.290(18)	C1	S1	O12	Cs1^{2}	126.58(10)
Cs1^{1}	S1	C1	S1^{3}	-111.70(5)	C1	S1	O12	Cs1^{1}	-108.51(10)
O12	S1	O11	Cs1^{3}	113.28(15)	C1	S1	O12	Cs1	-7.2(2)

O12	S1	O11	Cs1[4]	-97.34(13)	C1	S1	O11	Cs1[4]	145.47(12)
O12	S1	O11	Cs1[2]	-7.60(10)	C1	S1	O11	Cs1[3]	-3.92(19)
O12	S1	O13	Cs1[1]	-3.67(10)	C1	S1	O11	Cs1[2]	-124.79(9)
O12	S1	O13	Cs1[5]	81.3(3)	C1	S1	O13	Cs1[5]	-163.8(3)
O12	S1	O13	Cs1[6]	-109.74(12)	C1	S1	O13	Cs1[1]	111.28(4)
O12	S1	C1	S1[3]	-58.57(8)	C1	S1	O13	Cs1[6]	5.22(12)

[1]1/2-X,3/2-Y,1-Z; [2]+X,2-Y,-1/2+Z; [3]1-X,+Y,1/2-Z; [4]+X,+Y,-1+Z; [5]1/2-X,-1/2+Y,1/2-Z; [6]+X,1-Y,-1/2+Z

Table S70: Hydrogen Atom Coordinates (Å×10^4) and Isotropic Displacement Parameters (Å^2×10^3) for $Cs_2[H_2C(SO_3)_2]$.

Atom	*x*	*y*	*z*	U(eq)
H1	5250(20)	4970(40)	6700(40)	12(6)

$BaK_2[H_2C(SO_3)_2]_2$ (11)

Table S71: Crystallographic data of $BaK_2[H_2C(SO_3)_2]_2$.

Empirical formula	$C_2H_4BaK_2O_{12}S_4$
Formula weight	563.83 g/mol
Temperature	100(2) K
Crystal system	Orthorhombic
Space group	*Pbca* (No. 61)
	a = 957.54(5) pm
Unit cell dimensions	b = 1419.70(7) pm
	c = 1969.6(1) pm
Volume	2677.5(2) Å^3
Z	8
ρ_{calc}	2.797 g/cm^3
μ	4.271 mm^{-1}
F(000)	2160
Radiation	MoK$_\alpha$ (λ = 0.71073 nm)
Crystal size	0.314 x 0.282 x 0.194 mm^3
2Θ range for data collection	4.136 to 51.996
Index ranges	$-11 \leq h \leq 11$, $-17 \leq k \leq 17$, $-24 \leq l \leq 24$
Reflections collected	51233
Independent reflections	2610 [R_{int} = 0.0280, R_σ = 0.0145]
Completeness	99.4%
Absorption correction	multiscan
Min. and max. transmission	0.501 / 0.747
Data/restraints/parameters	2610/0/206
Goodness-of-fit on F^2	1.208
Final R indexes [I≥2σ (I)]	R_1 = 0.0151, wR_2 = 0.0373
Final R indexes [all data]	R_1 = 0.0152, wR_2 = 0.0374
Largest diff. peak/hole	0.48/–0.68 e · Å^{-3}
CCDC-No.	2120002

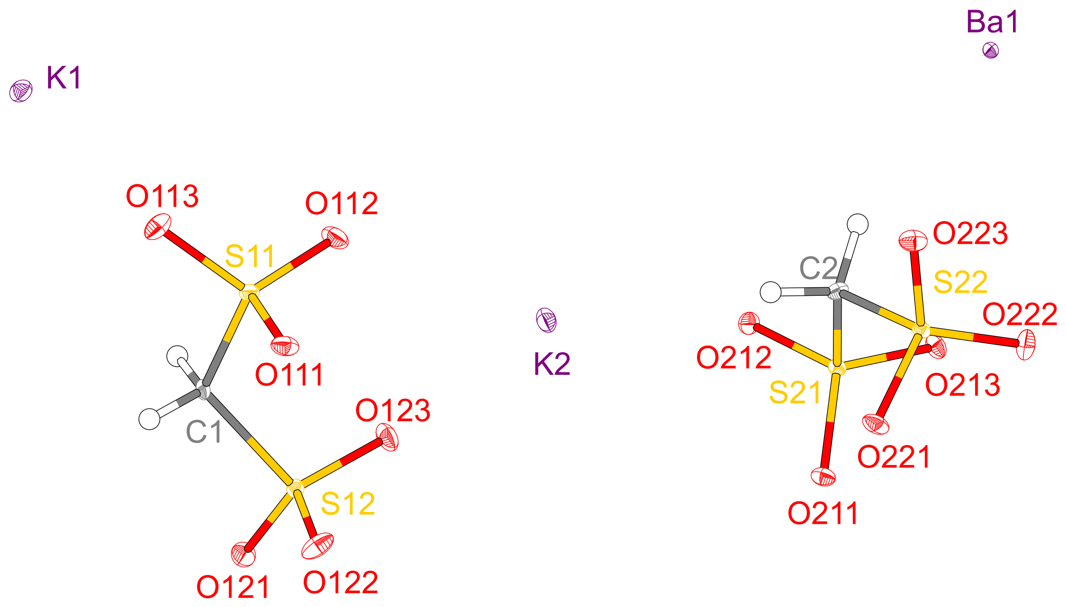

Figure S17: Thermal ellipsoid plot of the asymmetric unit of $BaK_2[H_2C(SO_3)_2]_2$. Thermal ellipsoids shown with 50% probability.

Table S72: Fractional Atomic Coordinates (×10[4]) and Equivalent Isotropic Displacement Parameters (Å^2×10^3) for $BaK_2[H_2C(SO_3)_2]_2$. U_{eq} is defined as 1/3 of the trace of the orthogonalised U_{IJ} tensor.

**Atom**	***x***	***y***	***z***	**U(eq)**
Ba1	7256.6(2)	6880.4(2)	1383.0(2)	4.46(5)
K2	7299.8(5)	1632.2(3)	3603.2(2)	8.98(10)
K1	986.4(5)	1012.7(3)	5661.1(2)	8.81(10)
S21	9859.8(5)	3142.9(3)	2473.5(2)	5.06(10)
S11	4847.8(5)	1850.0(3)	5284.5(3)	6.21(10)
S22	9953.7(5)	5301.9(3)	2399.8(2)	5.29(10)
S12	7423.4(5)	632.8(3)	5419.5(3)	5.6(1)
O213	10341.2(16)	2974.4(10)	1779.5(7)	8.1(3)
O111	5670.6(15)	2666.3(10)	5482.5(8)	10.0(3)
O211	10980.6(15)	3199.4(10)	2967.2(7)	7.8(3)
O221	10678.4(15)	5430.5(10)	3043.7(7)	9.5(3)
O122	8194.7(16)	1422.6(11)	5703.2(8)	12.2(3)
O112	4857.7(16)	1706.9(11)	4549.5(8)	11.3(3)
O121	7825.4(16)	-253.3(11)	5734.9(8)	11.0(3)
O212	8757.3(15)	2480.3(10)	2653.2(7)	7.9(3)
O222	10874.6(16)	5192.1(10)	1820.5(8)	10.7(3)
O113	3453.8(16)	1850.8(10)	5576.6(8)	11.8(3)
O223	8868.9(16)	6010.6(10)	2305.4(8)	9.5(3)
O123	7511.0(16)	573.7(11)	4683.9(8)	12.0(3)
C1	5647(2)	825.8(14)	5645.2(10)	6.0(4)
C2	8961(2)	4244.0(14)	2474.4(11)	6.8(4)

Table S73: Anisotropic Displacement Parameters (Å^2×10^3) for $BaK_2[H_2C(SO_3)_2]_2$. The anisotropic displacement factor exponent takes the form: $-2\pi^2[h^2a^{*2}U_{11}+2hka^*b^*U_{12}+\ldots]$.

**Atom**	**U_{11}**	**U_{22}**	**U_{33}**	**U_{23}**	**U_{13}**	**U_{12}**
Ba1	4.52(7)	5.06(7)	3.80(7)	-0.06(4)	-0.05(4)	-0.22(4)
K2	10.6(2)	9.2(2)	7.2(2)	2.01(16)	1.55(16)	1.84(17)
K1	6.8(2)	10.7(2)	8.9(2)	-1.43(16)	0.87(16)	0.63(16)
S21	5.6(2)	5.1(2)	4.5(2)	-0.24(17)	-0.36(18)	0.32(17)
S11	5.2(2)	7.3(2)	6.1(2)	-0.41(18)	-1.00(18)	0.16(18)
S22	5.9(2)	4.9(2)	5.1(2)	0.12(18)	-0.86(18)	-0.43(18)
S12	5.5(2)	5.8(2)	5.5(2)	0.08(17)	0.67(17)	0.47(18)
O213	11.2(7)	8.2(7)	4.8(7)	-1.1(5)	0.8(6)	1.4(6)
O111	9.5(7)	8.6(7)	11.9(7)	1.5(6)	-4.4(6)	-1.6(6)
O211	8.1(7)	8.6(7)	6.8(7)	-1.1(5)	-2.3(6)	0.9(5)
O221	11.4(7)	9.4(7)	7.8(7)	0.5(6)	-4.9(6)	-2.9(6)
O122	6.6(7)	10.0(7)	20.1(8)	-5.6(6)	0.4(6)	-1.0(6)
O112	12.6(8)	14.8(8)	6.6(7)	-0.4(6)	-2.4(6)	3.0(6)
O121	10.9(8)	9.3(7)	12.6(7)	4.2(6)	0.5(6)	1.9(6)
O212	8.0(7)	6.8(6)	8.9(7)	1.0(5)	0.1(6)	-1.2(6)
O222	12.0(7)	10.4(7)	9.8(7)	-0.6(6)	4.4(6)	-3.0(6)
O113	6.9(7)	12.1(8)	16.5(8)	-0.9(6)	2.4(6)	0.8(6)
O223	9.8(7)	5.8(7)	12.9(8)	0.5(6)	-2.8(6)	0.8(6)
O123	15.0(8)	14.8(8)	6.1(7)	1.3(6)	2.5(6)	5.3(6)
C1	6.7(9)	7.5(9)	3.7(10)	0.2(7)	1.4(7)	-0.2(8)
C2	4.7(9)	7.1(9)	8.5(10)	-0.2(8)	-0.8(8)	-0.6(7)

Table S74: Bond lengths and interatomic distances for $BaK_2[H_2C(SO_3)_2]_2$ in [pm].

Atom	Atom	Length/pm	Atom	Atom	Length/pm
Ba1	K2[1]	4.3773(5)	K1	S12[7]	3.5079(7)
Ba1	K2[2]	4.4077(5)	K1	S12[13]	3.4867(7)
Ba1	S21[2]	3.4545(5)	K1	O213[14]	2.7021(15)
Ba1	O213[2]	3.0348(15)	K1	O111[12]	2.9465(15)
Ba1	O111[3]	2.7384(15)	K1	O122[13]	2.7369(16)
Ba1	O211[4]	2.8272(14)	K1	O121[7]	3.1649(16)
Ba1	O221[5]	2.7921(14)	K1	O222[14]	2.8553(15)
Ba1	O122[3]	2.7899(15)	K1	O113	2.6506(16)
Ba1	O112[1]	2.7445(15)	K1	O123[7]	2.7577(16)
Ba1	O121[6]	2.6948(15)	S21	O213	1.4623(15)
Ba1	O212[2]	2.8155(15)	S21	O211	1.4505(15)
Ba1	O223	2.6850(15)	S21	O212	1.4576(15)
K2	K1[7]	4.3467(7)	S21	C2	1.785(2)
K2	K1[8]	3.8549(7)	S11	O111	1.4545(15)
K2	S22[9]	3.7930(7)	S11	O112	1.4620(16)
K2	S22[10]	3.7203(7)	S11	O113	1.4534(16)
K2	O213[5]	2.7778(15)	S11	C1	1.790(2)
K2	O112	2.9921(16)	S22	O221	1.4572(15)
K2	O212	2.6265(15)	S22	O222	1.4505(15)
K2	O222[9]	2.8164(15)	S22	O223	1.4580(15)
K2	O113[8]	2.9101(16)	S22	C2	1.784(2)
K2	O223[10]	2.9268(16)	S12	O122	1.4544(16)
K2	O123	2.6134(16)	S12	O121	1.4550(15)
K1	K1[11]	4.3150(9)	S12	O123	1.4536(16)
K1	S11[12]	3.7234(7)	S12	C1	1.780(2)

[1]1-X,1/2+Y,1/2-Z; [2]3/2-X,1/2+Y,+Z; [3]3/2-X,1-Y,-1/2+Z; [4]2-X,1/2+Y,1/2-Z; [5]-1/2+X,+Y,1/2-Z; [6]+X,1/2-Y,-1/2+Z; [7]1-X,-Y,1-Z; [8]1/2+X,1/2-Y,1-Z; [9]2-X,-1/2+Y,1/2-Z; [10]3/2-X,-1/2+Y,+Z; [11]-X,-Y,1-Z; [12]-1/2+X,1/2-Y,1-Z; [13]-1+X,+Y,+Z; [14]-1+X,1/2-Y,1/2+Z

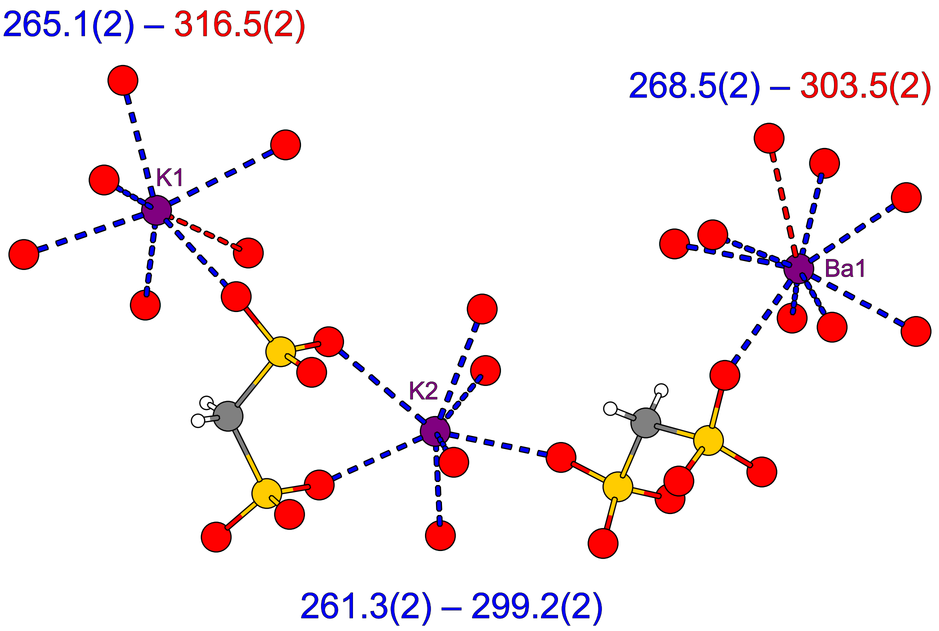

Figure S18: Coordination environment around the cations within compound **11**. Red and blue dotted lines indicate interatomic distances above and below 300 pm, respectively. The shortest and longest interatomic distances are given in [pm].

Table S75: Bond Angles for $BaK_2[H_2C(SO_3)_2]_2$.

Atom	Atom	Atom	Angle/°	Atom	Atom	Atom	Angle/°
K2^{1}	Ba1	K2^{2}	94.786(8)	O213^{15}	K1	K1^{13}	138.29(4)
S21^{2}	Ba1	K2^{1}	56.849(10)	O213^{15}	K1	S11^{12}	84.67(3)
S21^{2}	Ba1	K2^{2}	58.741(11)	O213^{15}	K1	S12^{14}	88.28(4)
O213^{2}	Ba1	K2^{2}	82.22(3)	O213^{15}	K1	S12^{7}	161.48(4)
O213^{2}	Ba1	K2^{1}	38.98(3)	O213^{15}	K1	O111^{12}	105.13(4)
O213^{2}	Ba1	S21^{2}	24.99(3)	O213^{15}	K1	O122^{14}	68.83(5)
O111^{3}	Ba1	K2^{1}	138.19(3)	O213^{15}	K1	O121^{7}	166.33(5)
O111^{3}	Ba1	K2^{2}	126.26(3)	O213^{15}	K1	O222^{15}	70.02(4)
O111^{3}	Ba1	S21^{2}	134.91(3)	O213^{15}	K1	O123^{7}	139.01(5)
O111^{3}	Ba1	O213^{2}	129.83(4)	O111^{12}	K1	K2^{12}	76.63(3)
O111^{3}	Ba1	O211^{4}	72.83(4)	O111^{12}	K1	K1^{13}	85.29(3)
O111^{3}	Ba1	O221^{5}	145.84(4)	O111^{12}	K1	S11^{12}	21.40(3)
O111^{3}	Ba1	O122^{3}	66.33(4)	O111^{12}	K1	S12^{7}	90.27(3)
O111^{3}	Ba1	O112^{1}	97.03(5)	O111^{12}	K1	S12^{14}	83.90(3)
O111^{3}	Ba1	O212^{2}	138.97(4)	O111^{12}	K1	O121^{7}	65.77(4)
O211^{4}	Ba1	K2^{1}	130.21(3)	O122^{14}	K1	K2^{12}	97.06(3)
O211^{4}	Ba1	K2^{2}	63.02(3)	O122^{14}	K1	K1^{13}	74.48(3)
O211^{4}	Ba1	S21^{2}	74.04(3)	O122^{14}	K1	S11^{12}	63.64(4)
O211^{4}	Ba1	O213^{2}	91.94(4)	O122^{14}	K1	S12^{14}	23.27(3)
O221^{5}	Ba1	K2^{1}	53.04(3)	O122^{14}	K1	S12^{7}	125.73(4)
O221^{5}	Ba1	K2^{2}	65.92(3)	O122^{14}	K1	O111^{12}	77.74(5)
O221^{5}	Ba1	S21^{2}	79.25(3)	O122^{14}	K1	O121^{7}	116.73(5)
O221^{5}	Ba1	O213^{2}	80.19(4)	O122^{14}	K1	O222^{15}	93.81(5)
O221^{5}	Ba1	O211^{4}	128.92(4)	O122^{14}	K1	O123^{7}	133.68(5)
O221^{5}	Ba1	O212^{2}	71.15(4)	O121^{7}	K1	K2^{12}	120.31(3)
O122^{3}	Ba1	K2^{2}	124.06(3)	O121^{7}	K1	K1^{13}	53.56(3)
O122^{3}	Ba1	K2^{1}	85.30(3)	O121^{7}	K1	S11^{12}	87.04(3)
O122^{3}	Ba1	S21^{2}	76.08(3)	O121^{7}	K1	S12^{14}	100.40(3)
O122^{3}	Ba1	O213^{2}	63.55(4)	O121^{7}	K1	S12^{7}	24.50(3)
O122^{3}	Ba1	O211^{4}	74.80(4)	O222^{15}	K1	K2^{12}	103.37(3)
O122^{3}	Ba1	O221^{5}	138.32(4)	O222^{15}	K1	K1^{13}	93.84(3)
O122^{3}	Ba1	O212^{2}	96.42(4)	O222^{15}	K1	S11^{12}	151.32(3)
O112^{1}	Ba1	K2^{2}	136.70(3)	O222^{15}	K1	S12^{7}	95.88(3)
O112^{1}	Ba1	K2^{1}	42.41(3)	O222^{15}	K1	S12^{14}	88.84(3)
O112^{1}	Ba1	S21^{2}	91.71(3)	O222^{15}	K1	O111^{12}	171.44(4)
O112^{1}	Ba1	O213^{2}	67.26(4)	O222^{15}	K1	O121^{7}	120.28(4)
O112^{1}	Ba1	O211^{4}	143.39(4)	O113	K1	K2^{12}	48.96(3)
O112^{1}	Ba1	O221^{5}	78.77(4)	O113	K1	K1^{13}	130.66(4)
O112^{1}	Ba1	O122^{3}	69.03(5)	O113	K1	S11^{12}	82.17(4)
O112^{1}	Ba1	O212^{2}	111.55(4)	O113	K1	S12^{14}	158.92(4)
O121^{6}	Ba1	K2^{1}	97.78(3)	O113	K1	S12^{7}	82.76(4)
O121^{6}	Ba1	K2^{2}	112.45(3)	O113	K1	O213^{15}	90.92(5)
O121^{6}	Ba1	S21^{2}	149.08(3)	O113	K1	O111^{12}	75.98(5)
O121^{6}	Ba1	O213^{2}	136.57(4)	O113	K1	O122^{14}	140.99(5)
O121^{6}	Ba1	O111^{3}	75.42(4)	O113	K1	O121^{7}	77.17(5)
O121^{6}	Ba1	O211^{4}	131.41(4)	O113	K1	O222^{15}	110.64(5)
O121^{6}	Ba1	O221^{5}	70.67(4)	O113	K1	O123^{7}	83.46(5)
O121^{6}	Ba1	O122^{3}	122.97(5)	O123^{7}	K1	K2^{12}	129.11(4)
O121^{6}	Ba1	O112^{1}	75.83(5)	O123^{7}	K1	K1^{13}	62.32(4)
O121^{6}	Ba1	O212^{2}	138.58(4)	O123^{7}	K1	S11^{12}	134.03(4)

O212^{2}	Ba1	K2^{1}	71.04(3)	O123^{7}	K1	S12^{14}	110.52(4)
O212^{2}	Ba1	K2^{2}	34.49(3)	O123^{7}	K1	S12^{7}	23.09(3)
O212^{2}	Ba1	S21^{2}	24.25(3)	O123^{7}	K1	O111^{12}	112.64(5)
O212^{2}	Ba1	O213^{2}	48.23(4)	O123^{7}	K1	O121^{7}	47.17(4)
O212^{2}	Ba1	O211^{4}	66.63(4)	O123^{7}	K1	O222^{15}	74.18(4)
O223	Ba1	K2^{2}	40.23(3)	O213	S21	Ba1^{10}	61.24(6)
O223	Ba1	K2^{1}	122.13(3)	O213	S21	C2	107.23(9)
O223	Ba1	S21^{2}	98.93(3)	O211	S21	Ba1^{10}	151.60(6)
O223	Ba1	O213^{2}	122.18(4)	O211	S21	O213	113.74(9)
O223	Ba1	O111^{3}	97.44(5)	O211	S21	O212	114.13(9)
O223	Ba1	O211^{4}	69.82(4)	O211	S21	C2	107.93(9)
O223	Ba1	O221^{5}	72.45(4)	O212	S21	Ba1^{10}	52.50(6)
O223	Ba1	O122^{3}	144.19(4)	O212	S21	O213	110.47(9)
O223	Ba1	O112^{1}	146.69(5)	O212	S21	C2	102.45(9)
O223	Ba1	O121^{6}	79.05(5)	C2	S21	Ba1^{10}	99.90(7)
O223	Ba1	O212^{2}	74.70(4)	O111	S11	K1^{9}	47.65(6)
K1^{7}	K2	Ba1^{8}	116.345(12)	O111	S11	O112	111.88(9)
K1^{9}	K2	Ba1^{8}	66.588(10)	O111	S11	C1	108.02(10)
K1^{9}	K2	K1^{7}	138.342(17)	O112	S11	K1^{9}	67.42(7)
S22^{10}	K2	Ba1^{8}	57.774(10)	O112	S11	C1	106.10(9)
S22^{11}	K2	Ba1^{8}	137.259(15)	O113	S11	K1^{9}	117.79(6)
S22^{11}	K2	K1^{7}	58.782(12)	O113	S11	O111	113.00(9)
S22^{10}	K2	K1^{7}	89.586(14)	O113	S11	O112	113.46(9)
S22^{10}	K2	K1^{9}	119.379(16)	O113	S11	C1	103.65(9)
S22^{11}	K2	K1^{9}	148.638(17)	C1	S11	K1^{9}	137.33(7)
S22^{10}	K2	S22^{11}	79.485(11)	K2^{2}	S22	K2^{4}	118.761(18)
O213^{5}	K2	Ba1^{8}	43.41(3)	O221	S22	K2^{4}	93.48(6)
O213^{5}	K2	K1^{7}	159.71(4)	O221	S22	K2^{2}	70.01(6)
O213^{5}	K2	K1^{9}	44.50(3)	O221	S22	O223	111.34(9)
O213^{5}	K2	S22^{11}	131.94(3)	O221	S22	C2	106.73(9)
O213^{5}	K2	S22^{10}	77.51(3)	O222	S22	K2^{2}	155.27(6)
O213^{5}	K2	O112	67.49(4)	O222	S22	K2^{4}	38.94(6)
O213^{5}	K2	O222^{11}	146.78(5)	O222	S22	O221	114.11(9)
O213^{5}	K2	O113^{9}	84.22(4)	O222	S22	O223	114.01(9)
O213^{5}	K2	O223^{10}	73.25(4)	O222	S22	C2	107.36(9)
O112	K2	Ba1^{8}	38.22(3)	O223	S22	K2^{2}	46.98(6)
O112	K2	K1^{9}	58.68(3)	O223	S22	K2^{4}	94.81(6)
O112	K2	K1^{7}	96.78(3)	O223	S22	C2	102.22(9)
O112	K2	S22^{11}	152.12(3)	C2	S22	K2^{2}	93.77(7)
O112	K2	S22^{10}	87.80(3)	C2	S22	K2^{4}	146.29(7)
O212	K2	Ba1^{8}	119.81(3)	K1^{16}	S12	K1^{7}	76.179(15)
O212	K2	K1^{9}	92.50(3)	O122	S12	K1^{7}	121.75(7)
O212	K2	K1^{7}	115.65(3)	O122	S12	K1^{16}	48.04(6)
O212	K2	S22^{10}	94.99(3)	O122	S12	O121	111.60(9)
O212	K2	S22^{11}	59.25(3)	O122	S12	C1	105.71(9)
O212	K2	O213^{5}	81.43(5)	O121	S12	K1^{7}	64.43(6)
O212	K2	O112	147.43(5)	O121	S12	K1^{16}	79.43(6)
O212	K2	O222^{11}	77.99(5)	O121	S12	C1	106.23(9)
O212	K2	O113^{9}	81.63(5)	O123	S12	K1^{16}	95.08(7)
O212	K2	O223^{10}	73.69(4)	O123	S12	K1^{7}	48.08(6)
O222^{11}	K2	Ba1^{8}	132.76(3)	O123	S12	O122	113.46(10)
O222^{11}	K2	K1^{7}	40.30(3)	O123	S12	O121	111.13(9)
O222^{11}	K2	K1^{9}	160.65(4)	O123	S12	C1	108.24(10)

O222^{11}	K2	S22^{10}	78.62(3)	C1	S12	K1^{16}	151.34(7)
O222^{11}	K2	S22^{11}	18.89(3)	C1	S12	K1^{7}	131.99(7)
O222^{11}	K2	O112	134.06(5)	K2^{17}	O213	Ba1^{10}	97.62(4)
O222^{11}	K2	O113^{9}	117.78(5)	K1^{18}	O213	Ba1^{10}	104.47(5)
O222^{11}	K2	O223^{10}	76.09(4)	K1^{18}	O213	K2^{17}	89.40(5)
O113^{9}	K2	Ba1^{8}	108.39(3)	S21	O213	Ba1^{10}	93.78(7)
O113^{9}	K2	K1^{7}	108.11(3)	S21	O213	K2^{17}	125.36(8)
O113^{9}	K2	K1^{9}	43.39(3)	S21	O213	K1^{18}	138.34(8)
O113^{9}	K2	S22^{11}	113.23(4)	Ba1^{19}	O111	K1^{9}	105.73(5)
O113^{9}	K2	S22^{10}	161.73(4)	S11	O111	Ba1^{19}	138.89(9)
O113^{9}	K2	O112	85.69(4)	S11	O111	K1^{9}	110.96(8)
O113^{9}	K2	O223^{10}	148.56(4)	S21	O211	Ba1^{11}	135.12(8)
O223^{10}	K2	Ba1^{8}	69.43(3)	S22	O221	Ba1^{17}	134.60(8)
O223^{10}	K2	K1^{9}	117.72(3)	K1^{16}	O122	Ba1^{19}	110.45(5)
O223^{10}	K2	K1^{7}	100.08(3)	S12	O122	Ba1^{19}	140.49(9)
O223^{10}	K2	S22^{10}	21.36(3)	S12	O122	K1^{16}	108.69(8)
O223^{10}	K2	S22^{11}	70.12(3)	Ba1^{8}	O112	K2	99.37(5)
O223^{10}	K2	O112	104.83(4)	S11	O112	Ba1^{8}	130.19(9)
O123	K2	Ba1^{8}	96.80(4)	S11	O112	K2	128.82(9)
O123	K2	K1^{7}	37.11(3)	Ba1^{20}	O121	K1^{7}	101.08(5)
O123	K2	K1^{9}	102.58(4)	S12	O121	Ba1^{20}	152.87(9)
O123	K2	S22^{11}	94.83(4)	S12	O121	K1^{7}	91.07(7)
O123	K2	S22^{10}	105.77(4)	K2	O212	Ba1^{10}	108.13(5)
O123	K2	O213^{5}	131.89(5)	S21	O212	Ba1^{10}	103.25(7)
O123	K2	O112	64.76(5)	S21	O212	K2	148.61(9)
O123	K2	O212	143.39(5)	K2^{4}	O222	K1^{18}	100.06(5)
O123	K2	O222^{11}	77.05(5)	S22	O222	K2^{4}	122.17(8)
O123	K2	O113^{9}	86.79(5)	S22	O222	K1^{18}	135.75(8)
O123	K2	O223^{10}	124.58(5)	K1	O113	K2^{12}	87.64(5)
K2^{12}	K1	K1^{13}	161.39(2)	S11	O113	K2^{12}	124.68(8)
S11^{12}	K1	K2^{12}	64.962(13)	S11	O113	K1	147.47(9)
S11^{12}	K1	K1^{13}	96.488(17)	Ba1	O223	K2^{2}	103.44(5)
S12^{7}	K1	K2^{12}	131.609(17)	S22	O223	Ba1	144.43(9)
S12^{14}	K1	K2^{12}	120.314(16)	S22	O223	K2^{2}	111.66(8)
S12^{14}	K1	K1^{13}	52.132(12)	K2	O123	K1^{7}	108.02(5)
S12^{7}	K1	K1^{13}	51.689(12)	S12	O123	K2	140.73(9)
S12^{7}	K1	S11^{12}	111.490(17)	S12	O123	K1^{7}	108.83(8)
S12^{14}	K1	S11^{12}	76.778(14)	S12	C1	S11	115.75(11)
S12^{14}	K1	S12^{7}	103.821(15)	S22	C2	S21	118.70(12)
O213^{15}	K1	K2^{12}	46.10(3)				

^{1}1-X,1/2+Y,1/2-Z; ^{2}3/2-X,1/2+Y,+Z; ^{3}3/2-X,1-Y,-1/2+Z; ^{4}2-X,1/2+Y,1/2-Z; ^{5}-1/2+X,+Y,1/2-Z; ^{6}+X,1/2-Y,-1/2+Z; ^{7}1-X,-Y,1-Z; ^{8}1-X,-1/2+Y,1/2-Z; ^{9}1/2+X,1/2-Y,1-Z; ^{10}3/2-X,-1/2+Y,+Z; ^{11}2-X,-1/2+Y,1/2-Z; ^{12}-1/2+X,1/2-Y,1-Z; ^{13}-X,-Y,1-Z; ^{14}-1+X,+Y,+Z; ^{15}-1+X,1/2-Y,1/2+Z; ^{16}1+X,+Y,+Z; ^{17}1/2+X,+Y,1/2-Z; ^{18}1+X,1/2-Y,-1/2+Z; ^{19}3/2-X,1-Y,1/2+Z; ^{20}+X,1/2-Y,1/2+Z

Table S76: Torsion Angles for $BaK_2[H_2C(SO_3)_2]_2$.

A	B	C	D	Angle/°	A	B	C	D	Angle/°
Ba1^{1}	S21	O213	K2^{2}	-102.11(9)	O112	S11	O111	Ba1^{8}	-129.54(13)
Ba1^{1}	S21	O213	K1^{3}	116.81(14)	O112	S11	O111	K1^{7}	22.16(11)
Ba1^{1}	S21	O211	Ba1^{4}	-2.8(2)	O112	S11	O113	K2^{10}	-124.62(10)
Ba1^{1}	S21	O212	K2	-178.6(2)	O112	S11	O113	K1	48.0(2)
Ba1^{1}	S21	C2	S22	-135.46(11)	O112	S11	C1	S12	62.80(14)
K2^{5}	S22	O221	Ba1^{2}	-83.31(10)	O121	S12	O122	Ba1^{8}	-120.33(14)

K2^{6}	S22	O221	Ba1^{2}	36.09(11)	O121	S12	O122	K1^{11}	51.58(11)
K2^{5}	S22	O222	K2^{6}	31.0(2)	O121	S12	O123	K2	-173.29(12)
K2^{5}	S22	O222	K1^{3}	-129.28(11)	O121	S12	O123	K1^{12}	-14.32(11)
K2^{6}	S22	O222	K1^{3}	-160.30(18)	O121	S12	C1	S11	-176.89(11)
K2^{6}	S22	O223	Ba1	64.35(14)	O212	S21	O213	Ba1^{1}	18.88(8)
K2^{5}	S22	O223	Ba1	-170.07(19)	O212	S21	O213	K2^{2}	-83.22(11)
K2^{6}	S22	O223	K2^{5}	-125.59(5)	O212	S21	O213	K1^{3}	135.69(12)
K2^{5}	S22	C2	S21	-142.41(12)	O212	S21	O211	Ba1^{4}	52.21(14)
K2^{6}	S22	C2	S21	52.2(2)	O212	S21	C2	S22	171.01(12)
K1^{7}	S11	O111	Ba1^{8}	-151.71(17)	O222	S22	O221	Ba1^{2}	70.34(14)
K1^{7}	S11	O112	Ba1^{9}	-94.55(10)	O222	S22	O223	Ba1	29.13(18)
K1^{7}	S11	O112	K2	67.88(8)	O222	S22	O223	K2^{5}	-160.80(8)
K1^{7}	S11	O113	K2^{10}	-48.77(11)	O222	S22	C2	S21	50.59(15)
K1^{7}	S11	O113	K1	123.87(15)	O113	S11	O111	Ba1^{8}	100.93(14)
K1^{7}	S11	C1	S12	-11.14(19)	O113	S11	O111	K1^{7}	-107.37(9)
K1^{11}	S12	O122	Ba1^{8}	-171.91(19)	O113	S11	O112	Ba1^{9}	17.17(14)
K1^{12}	S12	O122	Ba1^{8}	167.18(9)	O113	S11	O112	K2	179.60(9)
K1^{12}	S12	O122	K1^{11}	-20.91(10)	O113	S11	C1	S12	-177.43(11)
K1^{12}	S12	O121	Ba1^{13}	-117.2(2)	O223	S22	O221	Ba1^{2}	-60.45(14)
K1^{11}	S12	O121	Ba1^{13}	163.1(2)	O223	S22	O222	K2^{6}	66.10(12)
K1^{11}	S12	O121	K1^{12}	-79.62(3)	O223	S22	O222	K1^{3}	-94.19(13)
K1^{11}	S12	O123	K2	-92.67(14)	O223	S22	C2	S21	170.85(12)
K1^{12}	S12	O123	K2	-158.96(19)	O123	S12	O122	Ba1^{8}	113.19(14)
K1^{11}	S12	O123	K1^{12}	66.29(6)	O123	S12	O122	K1^{11}	-74.90(10)
K1^{11}	S12	C1	S11	85.40(18)	O123	S12	O121	Ba1^{13}	-105.5(2)
K1^{12}	S12	C1	S11	-106.94(11)	O123	S12	O121	K1^{12}	11.78(9)
O213	S21	O211	Ba1^{4}	-75.79(13)	O123	S12	C1	S11	-57.47(14)
O213	S21	O212	Ba1^{1}	-20.95(9)	C1	S11	O111	Ba1^{8}	-13.13(16)
O213	S21	O212	K2	160.49(15)	C1	S11	O111	K1^{7}	138.57(8)
O213	S21	C2	S22	-72.67(14)	C1	S11	O112	Ba1^{9}	130.31(11)
O111	S11	O112	Ba1^{9}	-112.12(11)	C1	S11	O112	K2	-67.26(12)
O111	S11	O112	K2	50.31(13)	C1	S11	O113	K2^{10}	120.77(10)
O111	S11	O113	K2^{10}	4.10(13)	C1	S11	O113	K1	-66.59(19)
O111	S11	O113	K1	176.74(15)	C1	S12	O122	Ba1^{8}	-5.27(17)
O111	S11	C1	S12	-57.31(14)	C1	S12	O122	K1^{11}	166.64(8)
O211	S21	O213	Ba1^{1}	148.74(7)	C1	S12	O121	Ba1^{13}	12.0(2)
O211	S21	O213	K2^{2}	46.63(12)	C1	S12	O121	K1^{12}	129.29(8)
O211	S21	O213	K1^{3}	-94.45(13)	C1	S12	O123	K2	70.43(16)
O211	S21	O212	Ba1^{1}	-150.60(7)	C1	S12	O123	K1^{12}	-130.61(9)
O211	S21	O212	K2	30.8(2)	C2	S21	O213	Ba1^{1}	-92.01(8)
O211	S21	C2	S22	50.24(15)	C2	S21	O213	K2^{2}	165.88(9)
O221	S22	O222	K2^{6}	-63.37(12)	C2	S21	O213	K1^{3}	24.79(16)
O221	S22	O222	K1^{3}	136.33(11)	C2	S21	O211	Ba1^{4}	165.37(11)
O221	S22	O223	Ba1	159.98(13)	C2	S21	O212	Ba1^{1}	93.02(9)
O221	S22	O223	K2^{5}	-29.95(10)	C2	S21	O212	K2	-85.54(18)
O221	S22	C2	S21	-72.16(14)	C2	S22	O221	Ba1^{2}	-171.23(11)
O122	S12	O121	Ba1^{13}	126.8(2)	C2	S22	O222	K2^{6}	178.57(9)
O122	S12	O121	K1^{12}	-115.97(8)	C2	S22	O222	K1^{3}	18.27(15)
O122	S12	O123	K2	-46.56(17)	C2	S22	O223	Ba1	-86.39(16)
O122	S12	O123	K1^{12}	112.40(9)	C2	S22	O223	K2^{5}	83.68(9)
O122	S12	C1	S11	64.41(14)					

[1]3/2-X,-1/2+Y,+Z; [2]1/2+X,+Y,1/2-Z; [3]1+X,1/2-Y,-1/2+Z; [4]2-X,-1/2+Y,1/2-Z; [5]3/2-X,1/2+Y,+Z; [6]2-X,1/2+Y,1/2-Z; [7]1/2+X,1/2-Y,1-Z; [8]3/2-X,1-Y,1/2+Z; [9]1-X,-1/2+Y,1/2-Z; [10]-1/2+X,1/2-Y,1-Z; [11]1+X,+Y,+Z; [12]1-X,-Y,1-Z; [13]+X,1/2-Y,1/2+Z

Table S77: Hydrogen Atom Coordinates (Å×10^4) and Isotropic Displacement Parameters (Å^2×10^3) for $BaK_2[H_2C(SO_3)_2]_2$.

**Atom**	***x***	***y***	***z***	**U(eq)**
H1A	5590(30)	884(17)	6080(14)	6(6)
H1B	5110(30)	294(18)	5536(12)	3(6)
H2A	8490(30)	4300(20)	2882(16)	19(7)
H2B	8360(30)	4220(20)	2107(15)	18(7)

$Li_3[HC(SO_3)_3](H_2O)_4$ (12)

Table S78: Crystallographic data of $Li_3[HC(SO_3)_3](H_2O)_4$.

Empirical formula	$CH_9Li_3O_{13}S_3$
Formula weight	346.08 g/mol
Temperature	101(2) K
Crystal system	monoclinic
Space group	$P2_1/c$ (No. 14)
Unit cell dimensions	a = 747.90(4) pm<br>b = 1006.63(5) pm<br>c = 1545.32(7) pm<br>β = 100.274(2)°
Volume	1144.75(2) Å^3
Z	4
ρ_{calc}	2.008 g/cm^3
μ	0.714 mm^{-1}
F(000)	704
Radiation	MoK_α (λ = 0.71073 nm)
Crystal size	0.156 x 0.13 x 0.114 mm^3
2Θ range for data collection	4.852 to 52.992
Index ranges	$-9 \leq h \leq 9$, $-12 \leq k \leq 12$, $-19 \leq l \leq 19$
Reflections collected	33544
Independent reflections	2376 [R_{int} = 0.0641, R_σ = 0.0244]
Completeness	99.9%
Absorption correction	multiscan
Min. and max. transmission	0.705 / 0.741
Data/restraints/parameters	2376/0/218
Goodness-of-fit on F^2	1.050
Final R indexes [I≥2σ (I)]	R_1 = 0.0240, wR_2 = 0.0634
Final R indexes [all data]	R_1 = 0.0258, wR_2 = 0.0651
Largest diff. peak/hole	0.36/–0.44 e · Å^{-3}
CCDC-No.	2305643

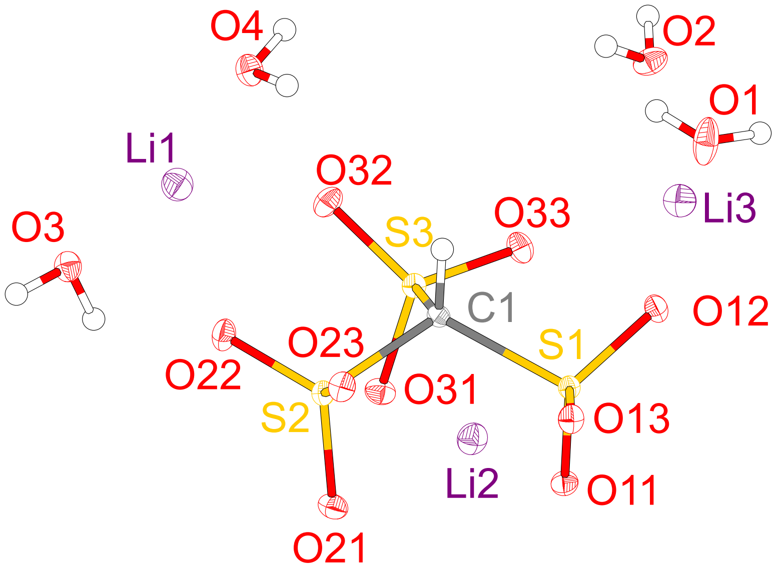

Figure S19: Thermal ellipsoid plot of the asymmetric unit of $Li_3[HC(SO_3)_3](H_2O)_4$. Thermal ellipsoids shown with 50% probability.

Table S79: Fractional Atomic Coordinates (×10^4) and Equivalent Isotropic Displacement Parameters (Å^2×10^3) for $Li_3[HC(SO_3)_3](H_2O)_4$. U_{eq} is defined as 1/3 of the trace of the orthogonalised U_{IJ} tensor.

Atom	*x*	*y*	*z*	U(eq)
S3	3965.3(5)	5541.9(4)	7480.8(2)	8.29(12)
S1	2227.1(5)	7555.4(4)	8504.9(2)	7.61(11)
S2	1156.8(5)	7442.0(4)	6542.7(2)	8.19(11)
O31	2417.9(15)	4630.8(11)	7345.9(8)	11.5(2)
O23	830.6(15)	8873.4(11)	6517.2(7)	10.7(2)
O12	3847.3(15)	7634.1(11)	9174.8(7)	10.2(2)
O13	1270.8(15)	8812.6(11)	8373.3(7)	10.1(2)
O11	1035.2(15)	6444.9(11)	8626.2(7)	10.3(2)
O21	-390.2(15)	6698.6(11)	6740.8(7)	11.5(2)
O33	5085.8(15)	5376.3(11)	8344.3(7)	11.9(2)
O22	1870.4(16)	6968.0(11)	5790.5(7)	12.3(2)
O3	3177.4(17)	4622.5(12)	4809.3(8)	12.0(2)
O32	4986.7(16)	5526.6(11)	6771.2(8)	12.5(2)
O4	5953.2(18)	7969.6(13)	5810.1(8)	12.8(3)
O2	7825.7(18)	7758.3(13)	9052.0(9)	13.7(3)
O1	7694.9(19)	5093.3(13)	9845.4(9)	19.5(3)
C1	2984(2)	7198.3(16)	7481.7(10)	7.8(3)
Li1	4402(4)	6231(3)	5471.0(19)	15.6(6)
Li3	6103(4)	6531(3)	9473.2(19)	14.9(6)
Li2	99(4)	10054(3)	7432.8(18)	14.0(6)

Table S80: Anisotropic Displacement Parameters (Å^2×10^3) for $Li_3[HC(SO_3)_3](H_2O)_4$. The anisotropic displacement factor exponent takes the form: $-2\pi^2[h^2a^{*2}U_{11}+2hka^*b^*U_{12}+\ldots]$.

Atom	U_{11}	U_{22}	U_{33}	U_{23}	U_{13}	U_{12}
S3	8.9(2)	7.13(19)	8.7(2)	0.05(13)	1.45(14)	0.97(14)
S1	8.0(2)	7.2(2)	7.48(19)	-0.26(13)	1.17(14)	0.28(13)
S2	9.2(2)	7.5(2)	7.71(19)	0.52(13)	1.03(14)	0.58(13)
O31	10.7(5)	8.6(5)	15.2(6)	-1.8(4)	2.2(4)	-1.0(4)
O23	13.2(5)	8.3(5)	10.6(5)	1.5(4)	2.1(4)	2.0(4)
O12	9.7(6)	11.0(5)	9.1(5)	-1.8(4)	-0.4(4)	1.5(4)
O13	11.8(5)	8.1(5)	10.2(5)	-0.5(4)	1.6(4)	2.6(4)
O11	10.8(5)	9.4(5)	10.8(5)	1.0(4)	2.8(4)	-1.8(4)
O21	9.6(5)	11.5(5)	13.0(6)	2.0(4)	0.5(4)	-1.4(4)
O33	12.7(6)	10.9(5)	10.7(6)	0.6(4)	-1.3(4)	1.4(4)
O22	14.6(6)	13.7(6)	8.8(5)	-1.6(4)	2.4(4)	2.7(5)
O3	14.2(6)	10.7(6)	10.7(6)	1.5(5)	1.4(5)	-0.1(5)
O32	13.8(6)	12.6(6)	12.3(6)	0.4(4)	5.6(4)	3.5(4)
O4	14.5(6)	11.3(6)	11.9(6)	-1.0(5)	0.7(5)	0.4(5)
O2	12.3(6)	13.5(6)	16.4(6)	3.5(5)	5.5(5)	3.0(5)
O1	29.3(7)	19.3(7)	9.0(6)	1.0(5)	1.3(5)	13.2(6)
C1	7.7(7)	7.3(7)	8.3(7)	-0.3(6)	1.0(6)	-0.6(6)
Li1	17.6(14)	13.7(14)	15.6(14)	-1.6(11)	3.4(11)	-2.4(11)
Li3	15.4(14)	13.2(14)	15.7(14)	2.1(11)	2.0(11)	1.6(11)
Li2	14.5(13)	13.7(14)	14.1(14)	-0.6(11)	3.3(11)	2.8(11)

Table S81: Bond lengths and interatomic distances for $Li_3[HC(SO_3)_3](H_2O)_4$ in [pm].

Atom	Atom	Length/pm	Atom	Atom	Length/pm
S3	O31	146.2(1)	O12	Li3	200.3(3)
S3	O33	145.3(1)	O13	Li2	199.7(3)
S3	O32	144.4(1)	O11	Li2^1	220.5(3)
S3	C1	182.2(2)	O21	Li2^1	207.9(3)
S1	O12	144.9(1)	O33	Li3	212.1(3)
S1	O13	145.0(1)	O22	Li1	217.1(3)
S1	O11	146.3(1)	O3	Li1^3	211.7(3)
S1	C1	180.8(2)	O3	Li1	204.2(3)
S2	O23	146.1(1)	O32	Li1	210.2(3)
S2	O21	145.6(1)	O4	Li1	211.4(3)
S2	O22	144.4(1)	O4	Li3^4	214.9(3)
S2	C1	182.4(2)	O2	Li3	197.7(3)
O31	Li2^1	201.8(3)	O1	Li3	189.7(3)
O23	Li2	199.7(3)	Li1	Li1^3	308.6(6)
O12	Li1^2	227.9(3)	Li1	Li3^4	312.6(4)

^{1}-X,-1/2+Y,3/2-Z; ^{2}+X,3/2-Y,1/2+Z; ^{3}1-X,1-Y,1-Z; ^{4}+X,3/2-Y,-1/2+Z

Table S82: Bond Angles for $Li_3[HC(SO_3)_3](H_2O)_4$.

Atom	Atom	Atom	Angle/°	Atom	Atom	Atom	Angle/°
O31	S3	C1	105.40(7)	O3	Li1	O32	102.10(13)
O33	S3	O31	111.78(7)	O3	Li1	O4	163.31(17)
O33	S3	C1	105.70(7)	O3^3	Li1	Li1^3	41.18(8)
O32	S3	O31	113.43(7)	O3	Li1	Li1^3	43.05(8)
O32	S3	O33	113.55(7)	O3	Li1	Li3^4	120.66(13)
O32	S3	C1	106.14(7)	O3^3	Li1	Li3^4	75.73(10)
O12	S1	O13	112.41(7)	O32	Li1	O12^4	169.61(15)
O12	S1	O11	113.67(7)	O32	Li1	O22	86.50(11)
O12	S1	C1	106.45(7)	O32	Li1	O3^3	91.09(12)
O13	S1	O11	112.86(7)	O32	Li1	O4	91.77(12)
O13	S1	C1	105.93(7)	O32	Li1	Li1^3	98.73(15)
O11	S1	C1	104.67(7)	O32	Li1	Li3^4	132.93(13)
O23	S2	C1	104.60(7)	O4	Li1	O12^4	78.96(11)
O21	S2	O23	112.19(7)	O4	Li1	O22	97.06(12)
O21	S2	C1	105.94(7)	O4	Li1	O3^3	86.29(12)
O22	S2	O23	112.84(7)	O4	Li1	Li1^3	126.12(17)
O22	S2	O21	114.91(7)	O4	Li1	Li3^4	43.27(8)
O22	S2	C1	105.29(7)	Li1^3	Li1	Li3^4	99.69(14)
S3	O31	Li2^1	125.61(11)	O12	Li3	O33	87.17(12)
S2	O23	Li2	129.28(11)	O12	Li3	O4^2	84.58(12)
S1	O12	Li1^2	130.80(10)	O12	Li3	Li1^2	46.69(9)
S1	O12	Li3	134.17(11)	O33	Li3	O4^2	147.82(16)
Li3	O12	Li1^2	93.54(12)	O33	Li3	Li1^2	133.07(13)
S1	O13	Li2	142.15(11)	O4^2	Li3	Li1^2	42.39(8)
S1	O11	Li2^1	123.57(10)	O2	Li3	O12	98.60(13)
S2	O21	Li2^1	122.59(10)	O2	Li3	O33	103.35(14)
S3	O33	Li3	139.09(10)	O2	Li3	O4^2	108.62(14)
S2	O22	Li1	139.34(10)	O2	Li3	Li1^2	93.36(12)
Li1	O3	Li1^3	95.77(12)	O1	Li3	O12	161.75(18)
S3	O32	Li1	132.40(11)	O1	Li3	O33	86.83(12)
Li1	O4	Li3^4	94.34(12)	O1	Li3	O4^2	91.40(13)

S3	C1	S2	111.55(8)	O1	Li3	O2	99.55(14)
S1	C1	S3	111.82(8)	O1	Li3	Li1^2	133.54(14)
S1	C1	S2	111.21(8)	O31^5	Li2	O11^5	90.72(11)
O12^4	Li1	Li1^3	90.61(13)	O31^5	Li2	O21^5	93.58(12)
O12^4	Li1	Li3^4	39.77(8)	O23	Li2	O31^5	112.11(14)
O22	Li1	O12^4	89.95(11)	O23	Li2	O11^5	88.27(11)
O22	Li1	Li1^3	136.03(17)	O23	Li2	O21^5	153.79(16)
O22	Li1	Li3^4	108.30(12)	O13	Li2	O31^5	92.65(13)
O3^3	Li1	O12^4	92.98(12)	O13	Li2	O23	90.20(12)
O3	Li1	O12^4	87.84(12)	O13	Li2	O11^5	176.62(16)
O3^3	Li1	O22	175.93(16)	O13	Li2	O21^5	93.84(12)
O3	Li1	O22	93.06(12)	O21^5	Li2	O11^5	86.23(11)
O3	Li1	O3^3	84.23(12)				

^{1}-X,-1/2+Y,3/2-Z; ^{2}+X,3/2-Y,1/2+Z; ^{3}1-X,1-Y,1-Z; ^{4}+X,3/2-Y,-1/2+Z; ^{5}-X,1/2+Y,3/2-Z

Table S83: Torsion Angles for $Li_3[HC(SO_3)_3](H_2O)_4$.

A	B	C	D	Angle/°	A	B	C	D	Angle/°
O31	S3	O33	Li3	-123.90(16)	O21	S2	C1	S3	74.70(9)
O31	S3	O32	Li1	71.31(15)	O21	S2	C1	S1	-50.87(10)
O31	S3	C1	S1	76.24(9)	O33	S3	O31	Li2^1	94.19(13)
O31	S3	C1	S2	-48.99(10)	O33	S3	O32	Li1	-159.65(13)
O23	S2	O21	Li2^1	-140.00(12)	O33	S3	C1	S1	-42.26(10)
O23	S2	O22	Li1	107.92(16)	O33	S3	C1	S2	-167.50(8)
O23	S2	C1	S3	-166.60(8)	O22	S2	O23	Li2	-173.25(13)
O23	S2	C1	S1	67.83(9)	O22	S2	O21	Li2^1	89.34(13)
O12	S1	O13	Li2	133.08(17)	O22	S2	C1	S3	-47.45(10)
O12	S1	O11	Li2^1	-131.43(11)	O22	S2	C1	S1	-173.03(8)
O12	S1	C1	S3	67.54(9)	O32	S3	O31	Li2^1	-135.86(12)
O12	S1	C1	S2	-167.04(8)	O32	S3	O33	Li3	106.22(16)
O13	S1	O12	Li1^2	37.46(15)	O32	S3	C1	S1	-163.15(8)
O13	S1	O12	Li3	-160.31(14)	O32	S3	C1	S2	71.61(10)
O13	S1	O11	Li2^1	99.04(12)	C1	S3	O31	Li2^1	-20.16(14)
O13	S1	C1	S3	-172.62(8)	C1	S3	O33	Li3	-9.73(18)
O13	S1	C1	S2	-47.19(10)	C1	S3	O32	Li1	-43.96(15)
O11	S1	O12	Li1^2	-92.30(13)	C1	S1	O12	Li1^2	153.01(13)
O11	S1	O12	Li3	69.94(16)	C1	S1	O12	Li3	-44.76(16)
O11	S1	O13	Li2	-96.75(18)	C1	S1	O13	Li2	17.21(19)
O11	S1	C1	S3	-53.13(10)	C1	S1	O11	Li2^1	-15.68(13)
O11	S1	C1	S2	72.29(9)	C1	S2	O23	Li2	-59.33(15)
O21	S2	O23	Li2	55.04(15)	C1	S2	O21	Li2^1	-26.45(14)
O21	S2	O22	Li1	-121.73(16)	C1	S2	O22	Li1	-5.57(18)

^{1}-X,-1/2+Y,3/2-Z; ^{2}+X,3/2-Y,1/2+Z

Table S84: Hydrogen Atom Coordinates (Å×10^4) and Isotropic Displacement Parameters (Å^2×10^3) for $Li_3[HC(SO_3)_3](H_2O)_4$.

Atom	*x*	*y*	*z*	U(eq)
H1	3880(30)	7790(20)	7423(12)	10(5)
H2A	8680(40)	7400(30)	9066(17)	29(7)
H1A	8120(40)	4720(30)	9488(18)	33(7)
H3A	2590(40)	4160(30)	5086(18)	32(7)
H3B	2450(40)	4810(30)	4380(20)	47(8)

H4A	5610(40)	8660(30)	6030(17)	35(7)
H4B	6840(40)	7740(30)	6110(19)	38(8)
H2B	7720(40)	8240(30)	8634(19)	43(8)
H1B	8160(40)	4780(30)	10315(19)	37(7)

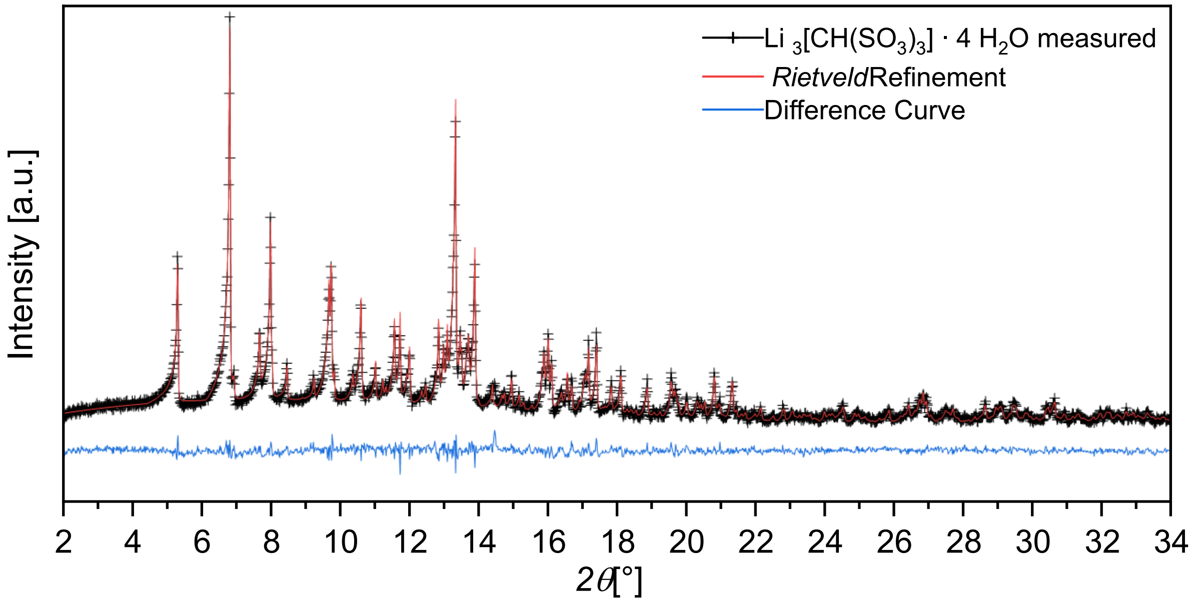

Figure S 20: X-ray powder diffraction pattern (black, cross), *Rietveld* refinement (red) and difference curve (blue) for $Li_3[HC(SO_3)_3](H_2O)_4$.

The final cell parameters after *Rietveld* refinement are a = 751.72(4) pm, b = 1011.96(4) pm, c = 1559.34(6) pm, β = 100.679(3), V = 765.3(3) Å^3 with *Rwp* = 6.466, *Rexp* = 5.779, Rp = 4.989 and *GooF* = 1.119.

$K_3[HC(SO_3)_3]$ (13)

Table S85: Crystallographic data of $K_3[HC(SO_3)_3]$.

Empirical formula	$CHK_3O_9S_3$
Formula weight	370.50 g/mol
Temperature	100(2) K
Crystal system	Orthorhombic
Space group	*Pbcm* (No. 57)
Unit cell dimensions	a = 871.20(5) pm<br>b = 913.50(5) pm<br>c = 1273.79(7) pm
Volume	1013.7(1) $Å^3$
Z	4
ρ_{calc}	2.428 g/cm^3
μ	1.993 mm^{-1}
F(000)	736
Radiation	MoK_α (λ = 0.71073 nm)
Crystal size	0.05 x 0.02 x 0.02 mm^3
2Θ range for data collection	4.676 to 61.098
Index ranges	$-11 \leq h \leq 12$, $-13 \leq k \leq 11$, $-17 \leq l \leq 17$
Reflections collected	14706
Independent reflections	1599 [R_{int} = 0.0416, R_σ = 0.0252]
Completeness	100%
Absorption correction	multiscan
Min. and max. transmission	0.611 / 0.746
Data/restraints/parameters	1599/0/80
Goodness-of-fit on F^2	1.131
Final R indexes [$I \geq 2\sigma$ (I)]	R_1 = 0.0746, wR_2 = 0.1593
Final R indexes [all data]	R_1 = 0.0882, wR_2 = 0.1739
Largest diff. peak/hole	1.20/–1.21 e · $Å^{-3}$
CCDC-No.	2517622

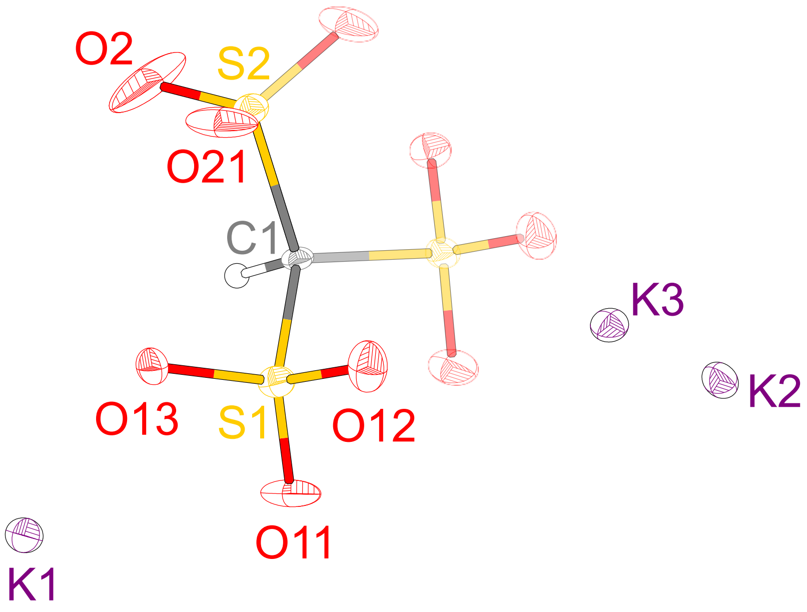

Figure S21: Thermal ellipsoid plot of the asymmetric unit of $K_3[HC(SO_3)_3]$. Thermal ellipsoids shown with 50% probability. Atoms generated due to symmetry for representation of the complete molecule are shown at 50% visibility.

Table S86: Fractional Atomic Coordinates (×10^4) and Equivalent Isotropic Displacement Parameters (Å^2×10^3) for $K_3[HC(SO_3)_3]$. U_{eq} is defined as 1/3 of the trace of the orthogonalised U_{IJ} tensor.

Atom	*x*	*y*	*z*	U(eq)
K1	4825(2)	2500	5000	18.4(4)
K2	-730(2)	2500	5000	18.1(4)
S1	2037.7(18)	513.1(17)	3712.7(12)	14.8(3)
O11	2009(7)	2100(5)	3686(4)	26.9(12)
O12	519(6)	-145(6)	3731(4)	25.2(11)
O13	3054(5)	-33(5)	4532(3)	17.1(9)
C1	2953(10)	-55(9)	2500	11.0(14)
K3	8006(2)	-512(2)	2500	16.6(4)
S2	6519(3)	2981(2)	2500	14.5(4)
O21	7130(8)	2302(5)	3424(4)	35.8(15)
O2	4888(11)	3067(10)	2500	71(4)

Table S87: Anisotropic Displacement Parameters (Å^2×10^3) for $K_3[HC(SO_3)_3]$. The anisotropic displacement factor exponent takes the form: $-2\pi^2[h^2a^{*2}U_{11}+2hka^*b^*U_{12}+\ldots]$.

Atom	U_{11}	U_{22}	U_{33}	U_{23}	U_{13}	U_{12}
K1	16.7(8)	18.0(8)	20.4(9)	-3.7(7)	0	0
K2	17.4(9)	22.0(9)	14.8(8)	4.4(7)	0	0
S1	19.7(7)	13.7(6)	11.1(6)	0.9(5)	1.5(6)	2.7(5)
O11	52(3)	12(2)	17(2)	-1.9(18)	8(2)	7(2)
O12	22(2)	36(3)	17(2)	4(2)	0(2)	1(2)
O13	20(2)	18(2)	13(2)	-0.2(17)	-4.2(17)	1.5(18)
C1	16(4)	7(3)	10(3)	0	0	3(3)
K3	17.1(9)	11.6(8)	21.2(9)	0	0	-1.2(7)
S2	16.1(9)	10.7(8)	16.6(10)	0	0	-1.3(8)
O21	78(4)	11(2)	18(2)	1.4(19)	-12(3)	-8(3)
O2	22(4)	14(4)	175(14)	0	0	-5(3)

Table S88: Bond lengths and interatomic distances for $K_3[HC(SO_3)_3]$ in [pm].

Atom	Atom	Length/pm	Atom	Atom	Length/pm
K1	O11^1	299.2(6)	K2	O12^1	310.4(6)
K1	O11	299.2(6)	K2	O13^5	308.8(5)
K1	O13	284.4(5)	K2	O13^4	308.8(5)
K1	O13^1	284.4(5)	K2	O21^6	274.6(6)
K1	O13^2	297.5(5)	K2	O21^7	274.6(6)
K1	O13^3	297.5(5)	S1	O11	145.0(5)
K1	S2^1	353.7(1)	S1	O12	145.4(5)
K1	S2	353.7(1)	S1	O13	145.7(5)
K1	O21	284.5(7)	S1	C1	181.4(5)
K1	O21^1	284.5(7)	O11	K3^2	265.4(5)
K1	O2	322.7(1)	O12	K3^6	271.4(5)
K1	O2^1	322.7(1)	C1	S2^8	185.3(8)
K2	S1^4	340.0(2)	K3	O21^9	292.9(6)
K2	S1^5	340.0(2)	K3	O21	292.9(6)
K2	O11^1	293.7(6)	K3	O2^8	284(1)
K2	O11	293.7(6)	S2	O21^9	143.3(5)
K2	O12	310.4(6)	S2	O21	143.3(5)
K2	O12^5	269.7(5)	S2	O2	142(1)

K2	O12^{4}	2.697(5)

^{1}+X,1/2-Y,1-Z; ^{2}1-X,1/2+Y,+Z; ^{3}1-X,-Y,1-Z; ^{4}-X,-Y,1-Z; ^{5}-X,1/2+Y,+Z; ^{6}-1+X,+Y,+Z; ^{7}-1+X,1/2-Y,1-Z; ^{8}1-X,-1/2+Y,+Z; ^{9}+X,+Y,1/2-Z

Table S89: Bond Angles for $K_3[HC(SO_3)_3]$.

Atom	Atom	Atom	Angle/°	Atom	Atom	Atom	Angle/°
O11	K1	O11^{1}	69.8(2)	O21^{6}	K2	O21^{7}	94.4(3)
O11	K1	S2^{1}	146.15(12)	K2^{4}	S1	K2	87.84(4)
O11	K1	S2	81.60(11)	O11	S1	K2^{4}	144.5(2)
O11^{1}	K1	S2^{1}	81.60(11)	O11	S1	K2	58.0(2)
O11^{1}	K1	S2	146.15(12)	O11	S1	O12	113.4(3)
O11^{1}	K1	O2^{1}	58.79(19)	O11	S1	O13	111.7(3)
O11	K1	O2	58.79(19)	O11	S1	C1	105.9(3)
O11^{1}	K1	O2	123.1(2)	O12	S1	K2^{4}	49.6(2)
O11	K1	O2^{1}	123.1(2)	O12	S1	K2	64.6(2)
O13	K1	O11	48.60(13)	O12	S1	O13	113.6(3)
O13^{2}	K1	O11^{1}	119.32(13)	O12	S1	C1	107.2(4)
O13^{1}	K1	O11^{1}	48.60(13)	O13	S1	K2^{4}	65.2(2)
O13^{3}	K1	O11	119.32(13)	O13	S1	K2	105.4(2)
O13	K1	O11^{1}	76.82(14)	O13	S1	C1	104.2(3)
O13^{1}	K1	O11	76.82(14)	C1	S1	K2	150.0(2)
O13^{3}	K1	O11^{1}	121.93(12)	C1	S1	K2^{4}	109.1(3)
O13^{2}	K1	O11	121.93(12)	K2	O11	K1	109.41(16)
O13^{1}	K1	O13^{2}	155.67(17)	S1	O11	K1	95.4(3)
O13^{3}	K1	O13^{2}	103.20(19)	S1	O11	K2	97.2(3)
O13^{1}	K1	O13^{3}	76.26(14)	S1	O11	K3^{3}	146.6(3)
O13	K1	O13^{3}	155.67(17)	K3^{3}	O11	K1	102.82(18)
O13	K1	O13^{2}	76.26(14)	K3^{3}	O11	K2	102.60(17)
O13	K1	O13^{1}	114.3(2)	K2^{4}	O12	K2	109.44(16)
O13^{2}	K1	S2^{1}	57.74(9)	K2^{4}	O12	K3^{6}	107.63(19)
O13	K1	S2^{1}	108.31(10)	S1	O12	K2	90.3(3)
O13	K1	S2	97.96(10)	S1	O12	K2^{4}	106.1(3)
O13^{1}	K1	S2	108.31(10)	S1	O12	K3^{6}	140.9(3)
O13^{1}	K1	S2^{1}	97.96(10)	K3^{6}	O12	K2	96.56(16)
O13^{3}	K1	S2	57.74(9)	K1	O13	K1^{2}	103.74(14)
O13^{3}	K1	S2^{1}	90.88(10)	K1	O13	K2^{4}	155.36(17)
O13^{2}	K1	S2	90.88(10)	K1^{2}	O13	K2^{4}	79.37(12)
O13	K1	O21	100.56(14)	S1	O13	K1	101.6(2)
O13	K1	O21^{1}	125.62(13)	S1	O13	K1^{2}	141.3(3)
O13^{1}	K1	O21^{1}	100.56(14)	S1	O13	K2^{4}	89.4(2)
O13^{1}	K1	O21	125.62(14)	S1^{8}	C1	S1	116.7(5)
O13	K1	O2	86.15(18)	S1^{8}	C1	S2^{9}	112.7(3)
O13^{1}	K1	O2^{1}	86.15(18)	S1	C1	S2^{9}	112.7(3)
O13^{3}	K1	O2^{1}	107.99(19)	K2^{2}	K3	K1^{10}	131.14(5)
O13^{3}	K1	O2	70.72(18)	K2^{11}	K3	K1^{10}	158.27(6)
O13^{2}	K1	O2	107.99(19)	K2^{12}	K3	K1^{10}	82.74(2)
O13^{1}	K1	O2	94.92(19)	K2^{10}	K3	K1^{10}	66.84(3)
O13	K1	O2^{1}	94.92(19)	K2^{11}	K3	K2^{10}	131.24(5)
O13^{2}	K1	O2^{1}	70.73(18)	K2^{2}	K3	K2^{10}	93.64(5)
S2	K1	S2^{1}	130.67(9)	K2^{12}	K3	K2^{2}	131.24(5)
O21^{1}	K1	O11^{1}	100.16(16)	K2^{11}	K3	K2^{12}	94.12(4)
O21^{1}	K1	O11	168.83(16)	K2^{12}	K3	K2^{10}	65.899(18)
O21	K1	O11	100.16(16)	K2^{11}	K3	K2^{2}	65.899(19)

O21	K1	O11^{1}	168.83(16)	O11^{9}	K3	K1^{10}	94.00(12)
O21	K1	O13^{3}	57.87(13)	O11^{13}	K3	K1^{10}	41.32(13)
O21	K1	O13^{2}	69.82(14)	O11^{13}	K3	K2^{2}	94.35(13)
O21^{1}	K1	O13^{2}	57.87(13)	O11^{9}	K3	K2^{11}	95.99(10)
O21^{1}	K1	O13^{3}	69.82(15)	O11^{13}	K3	K2^{12}	95.99(10)
O21	K1	S2^{1}	109.44(14)	O11^{13}	K3	K2^{10}	41.02(12)
O21^{1}	K1	S2	109.44(14)	O11^{9}	K3	K2^{10}	94.35(13)
O21	K1	S2	22.81(12)	O11^{9}	K3	K2^{2}	41.02(12)
O21^{1}	K1	S2^{1}	22.81(12)	O11^{9}	K3	K2^{12}	159.71(14)
O21^{1}	K1	O21	90.2(2)	O11^{13}	K3	K2^{11}	159.71(14)
O21^{1}	K1	O2	132.4(2)	O11^{13}	K3	O11^{9}	69.4(2)
O21	K1	O2	45.7(2)	O11^{13}	K3	O12^{12}	77.07(17)
O21	K1	O2^{1}	132.4(2)	O11^{9}	K3	O12^{11}	77.07(17)
O21^{1}	K1	O2^{1}	45.7(2)	O11^{13}	K3	O12^{11}	115.75(19)
O2^{1}	K1	S2	154.32(19)	O11^{9}	K3	O12^{12}	115.75(19)
O2	K1	S2^{1}	154.32(19)	O11^{9}	K3	O21^{8}	161.66(19)
O2	K1	S2	23.72(17)	O11^{9}	K3	O21	119.44(15)
O2^{1}	K1	S2^{1}	23.72(17)	O11^{13}	K3	O21	161.66(19)
O2^{1}	K1	O2	178.0(3)	O11^{13}	K3	O21^{8}	119.44(15)
S1^{4}	K2	S1^{5}	140.85(8)	O11^{13}	K3	O2^{9}	67.63(19)
O11^{1}	K2	S1^{5}	116.50(10)	O11^{9}	K3	O2^{9}	67.63(19)
O11^{1}	K2	S1^{4}	95.63(10)	O12^{12}	K3	K1^{10}	94.84(11)
O11	K2	S1^{5}	95.63(10)	O12^{11}	K3	K1^{10}	156.57(13)
O11	K2	S1^{4}	116.50(10)	O12^{11}	K3	K2^{10}	91.97(12)
O11^{1}	K2	O11	71.3(2)	O12^{12}	K3	K2^{10}	36.05(12)
O11	K2	O12	47.29(14)	O12^{11}	K3	K2^{12}	98.09(12)
O11^{1}	K2	O12	96.24(15)	O12^{11}	K3	K2^{11}	45.14(12)
O11^{1}	K2	O12^{1}	47.29(14)	O12^{11}	K3	K2^{2}	36.05(12)
O11	K2	O12^{1}	96.24(15)	O12^{12}	K3	K2^{12}	45.14(12)
O11^{1}	K2	O13^{4}	120.90(13)	O12^{12}	K3	K2^{2}	91.97(12)
O11^{1}	K2	O13^{5}	123.50(13)	O12^{12}	K3	K2^{11}	98.09(12)
O11	K2	O13^{4}	123.50(13)	O12^{11}	K3	O12^{12}	70.6(2)
O11	K2	O13^{5}	120.90(13)	O12^{12}	K3	O21	109.52(18)
O12^{1}	K2	S1^{5}	74.83(10)	O12^{11}	K3	O21^{8}	109.52(18)
O12^{5}	K2	S1^{4}	163.24(13)	O12^{11}	K3	O21	82.51(18)
O12	K2	S1^{4}	74.83(10)	O12^{12}	K3	O21^{8}	82.51(18)
O12^{5}	K2	S1^{5}	24.25(11)	O12^{12}	K3	O2^{9}	140.69(14)
O12^{4}	K2	S1^{4}	24.25(11)	O12^{11}	K3	O2^{9}	140.69(14)
O12	K2	S1^{5}	119.77(10)	O21^{8}	K3	K1^{10}	85.74(12)
O12^{1}	K2	S1^{4}	119.77(10)	O21	K3	K1^{10}	120.34(13)
O12^{4}	K2	S1^{5}	163.24(13)	O21	K3	K2^{11}	38.43(12)
O12^{5}	K2	O11	72.69(15)	O21^{8}	K3	K2^{12}	38.43(12)
O12^{5}	K2	O11^{1}	100.76(15)	O21^{8}	K3	K2^{10}	102.34(11)
O12^{4}	K2	O11^{1}	72.69(15)	O21^{8}	K3	K2^{11}	78.76(12)
O12^{4}	K2	O11	100.76(15)	O21	K3	K2^{12}	78.76(12)
O12^{5}	K2	O12	106.56(15)	O21^{8}	K3	K2^{2}	143.11(13)
O12^{5}	K2	O12^{1}	70.56(16)	O21	K3	K2^{10}	143.11(13)
O12^{4}	K2	O12^{1}	106.56(15)	O21	K3	K2^{2}	102.34(11)
O12^{4}	K2	O12	70.56(16)	O21	K3	O21^{8}	47.4(2)
O12^{5}	K2	O12^{4}	172.2(2)	O2^{9}	K3	K1^{10}	46.81(4)
O12	K2	O12^{1}	139.0(2)	O2^{9}	K3	K2^{11}	120.97(10)
O12^{5}	K2	O13^{5}	49.22(14)	O2^{9}	K3	K2^{10}	107.02(12)
O12^{4}	K2	O13^{4}	49.22(14)	O2^{9}	K3	K2^{2}	107.02(12)

O12^{4}	K2	O13^{5}	137.96(15)	O2^{9}	K3	K2^{12}	120.97(10)
O12^{5}	K2	O13^{4}	137.96(15)	O2^{9}	K3	O21^{8}	99.8(2)
O12^{5}	K2	O21^{6}	70.16(15)	O2^{9}	K3	O21	99.8(2)
O12^{4}	K2	O21^{6}	115.60(16)	K1	S2	K1^{8}	128.38(9)
O12^{5}	K2	O21^{7}	115.60(16)	C1^{3}	S2	K1	102.93(11)
O12^{4}	K2	O21^{7}	70.16(15)	C1^{3}	S2	K1^{8}	102.93(11)
O13^{4}	K2	S1^{4}	25.36(9)	O21^{8}	S2	K1	147.2(2)
O13^{4}	K2	S1^{5}	117.66(11)	O21^{8}	S2	K1^{8}	50.3(3)
O13^{5}	K2	S1^{4}	117.66(11)	O21	S2	K1	50.3(3)
O13^{5}	K2	S1^{5}	25.36(9)	O21	S2	K1^{8}	147.2(2)
O13^{5}	K2	O12^{1}	76.24(13)	O21^{8}	S2	C1^{3}	109.1(3)
O13^{5}	K2	O12	134.23(13)	O21	S2	C1^{3}	109.1(3)
O13^{4}	K2	O12	76.24(13)	O21	S2	O21^{8}	110.5(5)
O13^{4}	K2	O12^{1}	134.23(13)	O2	S2	K1	65.81(7)
O13^{4}	K2	O13^{5}	98.05(18)	O2	S2	K1^{8}	65.81(7)
O21^{6}	K2	S1^{4}	94.12(12)	O2	S2	C1^{3}	101.2(5)
O21^{6}	K2	S1^{5}	58.21(11)	O2	S2	O21^{8}	113.3(3)
O21^{7}	K2	S1^{5}	94.12(12)	O2	S2	O21	113.3(3)
O21^{7}	K2	S1^{4}	58.21(11)	K1	O21	K3	121.55(19)
O21^{7}	K2	O11^{1}	97.30(18)	K2^{11}	O21	K1	87.67(16)
O21^{6}	K2	O11	97.30(18)	K2^{11}	O21	K3	100.1(2)
O21^{6}	K2	O11^{1}	167.52(18)	S2	O21	K1	106.9(4)
O21^{7}	K2	O11	167.52(18)	S2	O21	K2^{11}	145.5(3)
O21^{6}	K2	O12^{1}	132.06(14)	S2	O21	K3	98.4(3)
O21^{7}	K2	O12	132.06(15)	K1^{8}	O2	K1	161.4(3)
O21^{6}	K2	O12	78.83(16)	K3^{3}	O2	K1	93.34(18)
O21^{7}	K2	O12^{1}	78.83(16)	K3^{3}	O2	K1^{8}	93.34(18)
O21^{7}	K2	O13^{5}	69.39(15)	S2	O2	K1	90.47(19)
O21^{7}	K2	O13^{4}	57.42(14)	S2	O2	K1^{8}	90.47(19)
O21^{6}	K2	O13^{5}	57.42(14)	S2	O2	K3^{3}	155.9(5)
O21^{6}	K2	O13^{4}	69.39(15)				

^{1}+X,1/2-Y,1-Z; ^{2}1-X,-Y,1-Z; ^{3}1-X,1/2+Y,+Z; ^{4}-X,-Y,1-Z; ^{5}-X,1/2+Y,+Z; ^{6}-1+X,+Y,+Z; ^{7}-1+X,1/2-Y,1-Z; ^{8}+X,+Y,1/2-Z; ^{9}1-X,-1/2+Y,+Z; ^{10}1-X,-Y,-1/2+Z; ^{11}1+X,+Y,+Z; ^{12}1+X,+Y,1/2-Z; ^{13}1-X,-1/2+Y,1/2-Z

Table S90: Torsion Angles for K$_3$[HC(SO$_3$)$_3$].

A	B	C	D	Angle/°	A	B	C	D	Angle/°
K1^{1}	S2	O21	K1	-101.6(5)	O12	S1	O13	K1^{6}	83.6(5)
K1	S2	O21	K2^{2}	-111.5(7)	O12	S1	O13	K2^{4}	11.6(3)
K1^{1}	S2	O21	K2^{2}	146.8(3)	O12	S1	C1	S1^{1}	61.9(5)
K1	S2	O21	K3	126.7(3)	O12	S1	C1	S2^{7}	-70.9(5)
K1^{1}	S2	O21	K3	25.0(6)	O13	S1	O11	K1	15.3(3)
K1	S2	O2	K1^{1}	-161.4(3)	O13	S1	O11	K2	-95.1(3)
K1^{1}	S2	O2	K1	161.4(3)	O13	S1	O11	K3^{3}	138.6(6)
K1^{1}	S2	O2	K3^{3}	-99.28(16)	O13	S1	O12	K2	96.5(3)
K1	S2	O2	K3^{3}	99.28(16)	O13	S1	O12	K2^{4}	-13.8(4)
K2	S1	O11	K1	110.4(2)	O13	S1	O12	K3^{5}	-162.6(4)
K2^{4}	S1	O11	K1	92.6(4)	O13	S1	C1	S1^{1}	-177.4(4)
K2^{4}	S1	O11	K2	-17.8(5)	O13	S1	C1	S2^{7}	49.8(5)
K2	S1	O11	K3^{3}	-126.3(7)	C1	S1	O11	K1	-97.5(3)
K2^{4}	S1	O11	K3^{3}	-144.0(4)	C1	S1	O11	K2	152.1(3)
K2	S1	O12	K2^{4}	-110.4(2)	C1	S1	O11	K3^{3}	25.9(8)
K2^{4}	S1	O12	K2	110.4(2)	C1	S1	O12	K2	-149.0(2)
K2^{4}	S1	O12	K3^{5}	-148.8(6)	C1	S1	O12	K2^{4}	100.7(3)

K2	S1	O12	K3^{5}	100.8(5)	C1	S1	O12	K3^{5}	-48.1(6)
K2^{4}	S1	O13	K1	-157.8(2)	C1	S1	O13	K1	97.5(3)
K2^{4}	S1	O13	K1^{6}	72.0(4)	C1	S1	O13	K1^{6}	-32.7(5)
K2	S1	O13	K1^{6}	152.2(3)	C1	S1	O13	K2^{4}	-104.7(3)
K2	S1	O13	K1	-77.59(17)	C1^{3}	S2	O21	K1	91.2(3)
K2	S1	O13	K2^{4}	80.21(11)	C1^{3}	S2	O21	K2^{2}	-20.3(8)
K2	S1	C1	S1^{1}	-6.9(9)	C1^{3}	S2	O21	K3	-142.1(3)
K2^{4}	S1	C1	S1^{1}	114.3(4)	C1^{3}	S2	O2	K1	-99.28(16)
K2	S1	C1	S2^{7}	-139.7(3)	C1^{3}	S2	O2	K1^{1}	99.28(16)
K2^{4}	S1	C1	S2^{7}	-18.5(5)	C1^{3}	S2	O2	K3^{3}	0.0
O11	S1	O12	K2	-32.4(3)	O21^{1}	S2	O21	K1	-148.9(3)
O11	S1	O12	K2^{4}	-142.8(3)	O21^{1}	S2	O21	K2^{2}	99.6(6)
O11	S1	O12	K3^{5}	68.4(6)	O21^{1}	S2	O21	K3	-22.2(5)
O11	S1	O13	K1^{6}	-146.5(4)	O21^{1}	S2	O2	K1	144.1(2)
O11	S1	O13	K1	-16.4(3)	O21	S2	O2	K1	17.3(4)
O11	S1	O13	K2^{4}	141.4(3)	O21	S2	O2	K1^{1}	-144.1(2)
O11	S1	C1	S1^{1}	-59.5(6)	O21^{1}	S2	O2	K1^{1}	-17.3(4)
O11	S1	C1	S2^{7}	167.7(4)	O21^{1}	S2	O2	K3^{3}	-116.6(3)
O12	S1	O11	K1	145.2(2)	O21	S2	O2	K3^{3}	116.6(3)
O12	S1	O11	K2	34.8(3)	O2	S2	O21	K1	-20.6(5)
O12	S1	O11	K3^{3}	-91.4(7)	O2	S2	O21	K2^{2}	-132.2(7)
O12	S1	O13	K1	-146.2(3)	O2	S2	O21	K3	106.0(4)

^{1}+X,+Y,1/2-Z; ^{2}1+X,+Y,+Z; ^{3}1-X,1/2+Y,+Z; ^{4}-X,-Y,1-Z; ^{5}-1+X,+Y,+Z; ^{6}1-X,-Y,1-Z; ^{7}1-X,-1/2+Y,+Z

Table S91: Hydrogen Atom Coordinates (Å×10^4) and Isotropic Displacement Parameters (Å^2×10^3) for $K_3[HC(SO_3)_3]$.

Atom	*x*	*y*	*z*	U(eq)
H1	3959.95	471.52	2500	13

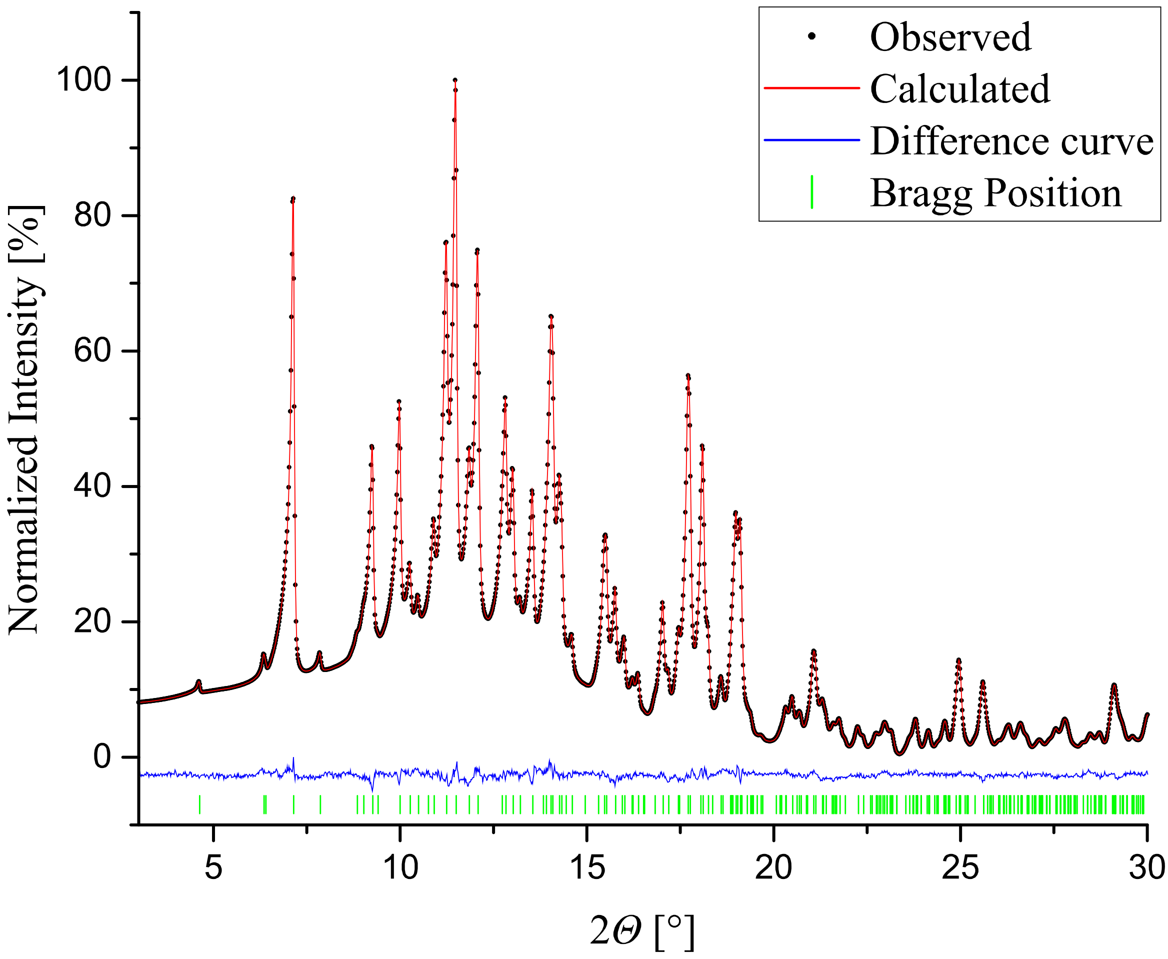

Figure S 22: Powder X-Ray Diffraction measurement of a dried sample of **13**. Observed reflexes, calculated pattern and the Bragg positions are shown as black dots, a red curve and green stripes, respectively.

The final cell parameters after *Rietveld* refinement are $a = 878.2(3)$ pm, $b = 918.7(3)$ pm, $c = 1279.4(4)$ pm, $V = 1032.3(6)$ Å^3 with $Rwp = 2.068$, $Rexp = 1.289$, $Rp = 1.650$ and $GooF = 1.605$.

$Rb_3[HC(SO_3)_3](H_2O)$ (14)

Table S92: Crystallographic data of $Rb_3[HC(SO_3)_3](H_2O)$.

Empirical formula	$CH_3O_{10}Rb_3S_3$
Formula weight	527.62 g/mol
Temperature	100(2) K
Crystal system	Orthorhombic
Space group	$P2_12_12_1$ (No. 19)
Unit cell dimensions	a = 963.94(5) pm<br>b = 966.25(5) pm<br>c = 1250.87(6) pm
Volume	1165.1(1) Å^3
Z	4
ρ_{calc}	3.008 g/cm^3
μ	13.127 mm^{-1}
F(000)	992
Radiation	MoK_α (λ = 0.71073 nm)
Crystal size	0.258 x 0.219 x 0.111 mm^3
2Θ range for data collection	5.328 to 61.008
Index ranges	$-13 \le h \le 13$, $-13 \le k \le 13$, $-17 \le l \le 17$
Reflections collected	34673
Independent reflections	3538 [R_{int} = 0.0545, R_σ = 0.0336]
Completeness	99.4%
Absorption correction	multiscan
Min. and max. transmission	0.382 / 0.746
Data/restraints/parameters	3538/0/168
Goodness-of-fit on F^2	1.040
Final R indexes [I≥2σ (I)]	R_1 = 0.0163, wR_2 = 0.0356
Final R indexes [all data]	R_1 = 0.0185, wR_2 = 0.0369
Largest diff. peak/hole	0.54/–0.81 e · Å^{-3}
CCDC-No.	2154779

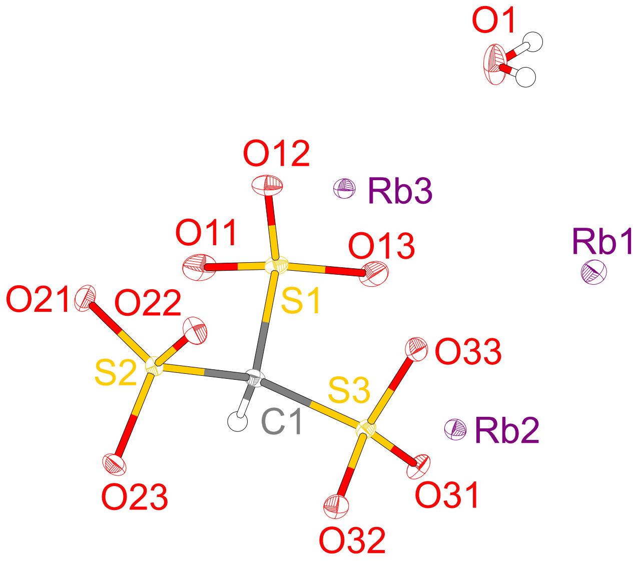

Figure S23: Thermal ellipsoid plot of the asymmetric unit of $Rb_3[HC(SO_3)_3](H_2O)$. Thermal ellipsoids shown with 50% probability.

Table S93: Fractional Atomic Coordinates (×10^4) and Equivalent Isotropic Displacement Parameters (Å^2×10^3) for $Rb_3[HC(SO_3)_3](H_2O)$. U_{eq} is defined as 1/3 of the trace of the orthogonalised U_{IJ} tensor.

Atom	*x*	*y*	*z*	U(eq)
Rb3	2458.8(3)	3403.5(3)	6678.9(2)	8.95(6)
Rb2	4891.4(3)	10023.7(3)	5618.4(2)	8.82(6)
Rb1	2090.0(3)	7716.3(3)	7781.3(3)	15.10(7)
S2	5834.8(7)	3923.2(8)	5001.4(5)	7.67(13)
S3	5436.5(7)	6147.4(7)	6706.8(6)	7.57(13)
S1	3808.9(7)	6271.1(7)	4630.2(5)	9.04(13)
O33	4118(2)	5695(2)	7152.9(17)	11.5(4)
O12	2835(2)	5131(2)	4724.1(16)	12.8(4)
O32	6619(2)	5425(2)	7170.6(17)	11.4(4)
O31	5611(2)	7639(2)	6717.8(17)	10.9(4)
O23	7342(2)	3862(2)	5078.0(16)	11.5(4)
O21	5346(2)	3660(2)	3918.2(17)	13.1(4)
O11	4266(2)	6537(3)	3537.1(17)	16.8(5)
O13	3356(2)	7522(2)	5173.9(18)	16.4(5)
O22	5156(2)	3062(2)	5797.9(16)	11.9(4)
O1	-28(3)	5340(3)	6772(2)	22.2(5)
C1	5405(3)	5720(3)	5290(2)	7.5(5)

Table S94: Anisotropic Displacement Parameters (Å^2×10^3) for $Rb_3[HC(SO_3)_3](H_2O)$. The anisotropic displacement factor exponent takes the form: $-2\pi^2[h^2a^{*2}U_{11}+2hka^*b^*U_{12}+\ldots]$.

Atom	U_{11}	U_{22}	U_{33}	U_{23}	U_{13}	U_{12}
Rb3	7.91(12)	10.30(13)	8.65(11)	-0.28(10)	-0.07(9)	-1.05(9)
Rb2	8.52(12)	8.53(13)	9.40(11)	0.46(10)	-0.29(9)	0.48(10)
Rb1	12.40(13)	14.14(14)	18.76(14)	-5.13(12)	-0.38(11)	-0.49(11)
S2	7.0(3)	8.2(3)	7.8(3)	-1.0(2)	0.4(2)	0.5(2)
S3	8.6(3)	7.6(3)	6.5(3)	0.0(3)	-1.2(2)	-0.1(2)
S1	8.4(3)	9.2(3)	9.5(3)	0.7(3)	-2.7(2)	-0.1(2)
O33	11.1(10)	13.4(11)	10.1(10)	-1.2(9)	1.7(8)	-1.6(8)
O12	9.7(9)	11.4(10)	17.4(10)	2.1(9)	-2.4(8)	-2.5(8)
O32	12.0(9)	12.5(10)	9.7(9)	1.0(8)	-2.9(8)	2.5(8)
O31	15.1(10)	8.1(10)	9.5(9)	-1.6(8)	-2.6(8)	-0.9(8)
O23	7.2(9)	11.8(10)	15.3(9)	-1.2(8)	1.0(7)	1.4(8)
O21	15.1(10)	13.7(11)	10.4(9)	-4.4(8)	-2.1(8)	2.6(8)
O11	15.8(10)	25.2(13)	9.4(9)	6.3(9)	-3.5(7)	-1.9(10)
O13	14.9(10)	12.0(11)	22.4(11)	-4.3(9)	-7.9(8)	5.4(8)
O22	12.6(10)	7.8(10)	15.3(10)	1.0(8)	3.9(8)	-0.3(8)
O1	28.7(13)	28.0(14)	9.9(10)	-1.7(10)	-0.7(10)	14.8(11)
C1	5.7(11)	9.8(13)	7.1(11)	1.5(10)	-0.2(9)	0.1(10)

Table S95: Bond lengths and interatomic distances for $Rb_3[HC(SO_3)_3](H_2O)$ in [pm].

Atom	Atom	Length/pm	Atom	Atom	Length/pm
Rb3	S2^1	345.29(8)	Rb1	S2^5	361.30(8)
Rb3	S3^2	359.81(7)	Rb1	S3	380.92(8)
Rb3	S1^3	390.14(7)	Rb1	O33	287.3(2)
Rb3	O33	279.5(2)	Rb1	O32^5	289.9(2)
Rb3	O12	298.3(2)	Rb1	O23^5	294.9(2)
Rb3	O32^2	333.9(2)	Rb1	O21^3	305.0(2)

Rb3	O31^{2}	283.4(2)	Rb1	O11^{7}	326.3(2)
Rb3	O23^{1}	310.4(2)	Rb1	O13	348.7(2)
Rb3	O21^{1}	294.6(2)	Rb1	O22^{5}	321.2(2)
Rb3	O11^{3}	285.9(2)	Rb1	O1	332.2(3)
Rb3	O22	284.3(2)	Rb1	O1^{8}	327.0(3)
Rb3	O1	304.3(3)	S2	O23	145.7(2)
Rb2	S2^{4}	395.22(8)	S2	O21	145.7(2)
Rb2	S3^{5}	353.18(8)	S2	O22	145.4(2)
Rb2	S1^{6}	399.02(8)	S2	C1	182.1(3)
Rb2	S1	397.04(8)	S3	O33	145.5(2)
Rb2	O33^{5}	301.8(2)	S3	O32	145.7(2)
Rb2	O12^{6}	287.3(2)	S3	O31	145.1(2)
Rb2	O32^{5}	314.9(2)	S3	C1	182.0(3)
Rb2	O31	277.2(2)	S1	O12	145.2(2)
Rb2	O23^{7}	282.1(2)	S1	O11	145.9(2)
Rb2	O13	288.8(2)	S1	O13	145.4(2)
Rb2	O22^{4}	295.5(2)	S1	C1	182.6(3)
Rb2	O1^{6}	301.2(2)			

^{1}-1/2+X,1/2-Y,1-Z; ^{2}1-X,-1/2+Y,3/2-Z; ^{3}1/2-X,1-Y,1/2+Z; ^{4}+X,1+Y,+Z; ^{5}1-X,1/2+Y,3/2-Z; ^{6}1/2+X,3/2-Y,1-Z; ^{7}-1/2+X,3/2-Y,1-Z; ^{8}-X,1/2+Y,3/2-Z

Table S96: Bond Angles for $Rb_3[HC(SO_3)_3](H_2O)$.

Atom	Atom	Atom	Angle/°	Atom	Atom	Atom	Angle/°
S2^{1}	Rb3	S3^{2}	101.698(18)	O21^{3}	Rb1	Rb2^{2}	92.26(4)
S2^{1}	Rb3	S1^{3}	119.108(17)	O21^{3}	Rb1	S2^{6}	102.06(4)
S3^{2}	Rb3	S1^{3}	72.204(16)	O21^{3}	Rb1	S3	130.02(4)
O33	Rb3	S2^{1}	154.74(5)	O21^{3}	Rb1	O11^{7}	71.93(6)
O33	Rb3	S3^{2}	92.19(5)	O21^{3}	Rb1	O13	133.00(6)
O33	Rb3	S1^{3}	85.10(5)	O21^{3}	Rb1	O22^{6}	115.07(6)
O33	Rb3	O12	70.18(6)	O21^{3}	Rb1	O1	53.32(6)
O33	Rb3	O32^{2}	116.05(6)	O21^{3}	Rb1	O1^{8}	77.90(6)
O33	Rb3	O31^{2}	71.39(6)	O11^{7}	Rb1	Rb2^{2}	156.21(5)
O33	Rb3	O23^{1}	136.86(6)	O11^{7}	Rb1	S2^{6}	141.11(5)
O33	Rb3	O21^{1}	170.19(6)	O11^{7}	Rb1	S3	128.03(4)
O33	Rb3	O11^{3}	98.30(7)	O11^{7}	Rb1	O13	80.27(5)
O33	Rb3	O22	69.57(6)	O11^{7}	Rb1	O1	56.50(6)
O33	Rb3	O1	87.44(7)	O11^{7}	Rb1	O1^{8}	53.69(6)
O12	Rb3	S2^{1}	85.44(4)	O13	Rb1	Rb2^{2}	99.37(4)
O12	Rb3	S3^{2}	136.94(4)	O13	Rb1	S2^{6}	122.79(4)
O12	Rb3	S1^{3}	140.23(4)	O13	Rb1	S3	49.63(4)
O12	Rb3	O32^{2}	143.47(5)	O22^{6}	Rb1	Rb2^{2}	42.35(4)
O12	Rb3	O23^{1}	79.56(6)	O22^{6}	Rb1	S2^{6}	23.67(4)
O12	Rb3	O1	77.46(6)	O22^{6}	Rb1	S3	62.40(4)
O32^{2}	Rb3	S2^{1}	79.74(4)	O22^{6}	Rb1	O11^{7}	161.08(6)
O32^{2}	Rb3	S3^{2}	23.87(4)	O22^{6}	Rb1	O13	103.53(5)
O32^{2}	Rb3	S1^{3}	75.23(4)	O22^{6}	Rb1	O1	142.20(6)
O31^{2}	Rb3	S2^{1}	123.96(5)	O22^{6}	Rb1	O1^{8}	109.10(6)
O31^{2}	Rb3	S3^{2}	22.27(4)	O1	Rb1	Rb2^{2}	99.86(5)
O31^{2}	Rb3	S1^{3}	63.70(5)	O1^{8}	Rb1	Rb2^{2}	141.74(5)
O31^{2}	Rb3	O12	130.27(6)	O1^{8}	Rb1	S2^{6}	87.43(5)
O31^{2}	Rb3	O32^{2}	45.18(6)	O1	Rb1	S2^{6}	148.78(5)
O31^{2}	Rb3	O23^{1}	109.94(6)	O1^{8}	Rb1	S3	152.08(5)
O31^{2}	Rb3	O21^{1}	117.18(6)	O1	Rb1	S3	96.40(5)

O31^{2}	Rb3	O11^{3}	79.14(6)	O1^{8}	Rb1	O13	114.46(6)
O31^{2}	Rb3	O22	69.13(6)	O1	Rb1	O13	79.78(6)
O31^{2}	Rb3	O1	130.57(6)	O1^{8}	Rb1	O1	103.13(2)
O23^{1}	Rb3	S2^{1}	24.96(4)	Rb3^{9}	S2	Rb3	128.41(2)
O23^{1}	Rb3	S3^{2}	89.46(4)	Rb3^{9}	S2	Rb2^{10}	66.574(14)
O23^{1}	Rb3	S1^{3}	135.68(4)	Rb3	S2	Rb2^{10}	65.207(12)
O23^{1}	Rb3	O32^{2}	72.96(5)	Rb3^{9}	S2	Rb1^{2}	90.351(18)
O21^{1}	Rb3	S2^{1}	24.73(4)	Rb1^{2}	S2	Rb3	90.423(16)
O21^{1}	Rb3	S3^{2}	97.01(5)	Rb1^{2}	S2	Rb2^{10}	70.716(14)
O21^{1}	Rb3	S1^{3}	94.47(4)	O23	S2	Rb3	142.22(9)
O21^{1}	Rb3	O12	104.77(6)	O23	S2	Rb3^{9}	63.99(9)
O21^{1}	Rb3	O32^{2}	73.14(6)	O23	S2	Rb2^{10}	100.24(9)
O21^{1}	Rb3	O23^{1}	47.02(5)	O23	S2	Rb1^{2}	52.00(9)
O21^{1}	Rb3	O1	83.19(7)	O23	S2	C1	104.62(13)
O11^{3}	Rb3	S2^{1}	104.14(5)	O21	S2	Rb3	102.00(9)
O11^{3}	Rb3	S3^{2}	83.33(5)	O21	S2	Rb3^{9}	57.76(9)
O11^{3}	Rb3	S1^{3}	17.59(5)	O21	S2	Rb2^{10}	86.59(10)
O11^{3}	Rb3	O12	136.55(6)	O21	S2	Rb1^{2}	146.77(10)
O11^{3}	Rb3	O32^{2}	79.73(6)	O21	S2	O23	112.11(12)
O11^{3}	Rb3	O23^{1}	124.67(6)	O21	S2	C1	106.12(13)
O11^{3}	Rb3	O21^{1}	79.49(6)	O22	S2	Rb3^{9}	104.39(9)
O11^{3}	Rb3	O1	59.88(7)	O22	S2	Rb3	35.20(9)
O22	Rb3	S2^{1}	95.91(5)	O22	S2	Rb2^{10}	38.45(8)
O22	Rb3	S3^{2}	68.38(4)	O22	S2	Rb1^{2}	62.51(9)
O22	Rb3	S1^{3}	131.50(4)	O22	S2	O23	112.36(13)
O22	Rb3	O12	68.66(6)	O22	S2	O21	113.06(13)
O22	Rb3	O32^{2}	79.83(5)	O22	S2	C1	107.91(12)
O22	Rb3	O23^{1}	71.14(6)	C1	S2	Rb3	80.01(9)
O22	Rb3	O21^{1}	117.08(6)	C1	S2	Rb3^{9}	147.62(9)
O22	Rb3	O11^{3}	148.16(6)	C1	S2	Rb2^{10}	144.88(9)
O22	Rb3	O1	143.76(7)	C1	S2	Rb1^{2}	106.34(9)
O1	Rb3	S2^{1}	93.82(6)	Rb3^{6}	S3	Rb3	146.13(2)
O1	Rb3	S3^{2}	142.67(5)	Rb3^{6}	S3	Rb1	92.200(17)
O1	Rb3	S1^{3}	70.58(5)	Rb2^{2}	S3	Rb3^{6}	72.848(15)
O1	Rb3	O32^{2}	136.34(7)	Rb2^{2}	S3	Rb3	74.581(14)
O1	Rb3	O23^{1}	115.61(7)	Rb2^{2}	S3	Rb1	73.280(15)
S2^{4}	Rb2	S1	150.497(16)	Rb1	S3	Rb3	69.581(13)
S2^{4}	Rb2	S1^{5}	93.781(16)	O33	S3	Rb3	32.66(9)
S3^{6}	Rb2	S2^{4}	84.979(16)	O33	S3	Rb3^{6}	117.34(9)
S3^{6}	Rb2	S1^{5}	104.768(16)	O33	S3	Rb2^{2}	57.74(9)
S3^{6}	Rb2	S1	123.517(17)	O33	S3	Rb1	40.94(9)
S1	Rb2	S1^{5}	86.453(11)	O33	S3	O32	112.74(13)
O33^{6}	Rb2	S2^{4}	84.41(4)	O33	S3	C1	106.90(13)
O33^{6}	Rb2	S3^{6}	24.07(4)	O32	S3	Rb3^{6}	68.03(9)
O33^{6}	Rb2	S1	124.55(4)	O32	S3	Rb3	104.65(9)
O33^{6}	Rb2	S1^{5}	80.79(4)	O32	S3	Rb2^{2}	62.96(9)
O33^{6}	Rb2	O32^{6}	46.25(5)	O32	S3	Rb1	135.44(9)
O12^{5}	Rb2	S2^{4}	78.07(4)	O32	S3	C1	106.97(13)
O12^{5}	Rb2	S3^{6}	104.22(4)	O31	S3	Rb3^{6}	47.73(9)
O12^{5}	Rb2	S1	99.53(4)	O31	S3	Rb3	139.40(9)
O12^{5}	Rb2	S1^{5}	15.75(4)	O31	S3	Rb2^{2}	107.86(9)
O12^{5}	Rb2	O33^{6}	80.59(6)	O31	S3	Rb1	72.53(9)
O12^{5}	Rb2	O32^{6}	126.42(6)	O31	S3	O33	113.33(13)

O12^{5}	Rb2	O13	115.71(6)	O31	S3	O32	112.42(13)
O12^{5}	Rb2	O22^{4}	88.74(6)	O31	S3	C1	103.69(13)
O12^{5}	Rb2	O1^{5}	79.65(7)	C1	S3	Rb3^{6}	133.89(10)
O32^{6}	Rb2	S2^{4}	99.24(4)	C1	S3	Rb3	79.93(9)
O32^{6}	Rb2	S3^{6}	24.33(4)	C1	S3	Rb2^{2}	148.34(10)
O32^{6}	Rb2	S1^{5}	122.98(4)	C1	S3	Rb1	114.83(9)
O32^{6}	Rb2	S1	105.33(4)	Rb3^{11}	S1	Rb3	124.142(18)
O31	Rb2	S2^{4}	146.29(4)	Rb3^{11}	S1	Rb2^{7}	69.825(12)
O31	Rb2	S3^{6}	78.93(5)	Rb3^{11}	S1	Rb2	107.657(17)
O31	Rb2	S1	57.39(4)	Rb2	S1	Rb3	121.309(18)
O31	Rb2	S1^{5}	62.68(5)	Rb2^{7}	S1	Rb3	61.570(12)
O31	Rb2	O33^{6}	68.97(6)	Rb2	S1	Rb2^{7}	124.009(19)
O31	Rb2	O12^{5}	77.49(6)	O12	S1	Rb3^{11}	86.28(9)
O31	Rb2	O32^{6}	77.43(6)	O12	S1	Rb3	37.89(8)
O31	Rb2	O23^{7}	133.53(6)	O12	S1	Rb2^{7}	32.49(8)
O31	Rb2	O13	61.84(6)	O12	S1	Rb2	146.87(9)
O31	Rb2	O22^{4}	140.05(6)	O12	S1	O11	113.87(13)
O31	Rb2	O1^{5}	112.89(7)	O12	S1	O13	113.48(13)
O23^{7}	Rb2	S2^{4}	77.07(5)	O12	S1	C1	106.70(12)
O23^{7}	Rb2	S3^{6}	95.58(4)	O11	S1	Rb3	145.29(11)
O23^{7}	Rb2	S1^{5}	156.94(4)	O11	S1	Rb3^{11}	36.29(9)
O23^{7}	Rb2	S1	91.35(4)	O11	S1	Rb2	92.95(10)
O23^{7}	Rb2	O33^{6}	118.62(6)	O11	S1	Rb2^{7}	105.55(9)
O23^{7}	Rb2	O12^{5}	146.52(6)	O11	S1	C1	102.72(13)
O23^{7}	Rb2	O32^{6}	79.72(6)	O13	S1	Rb3^{11}	106.35(9)
O23^{7}	Rb2	O13	79.25(6)	O13	S1	Rb3	100.31(10)
O23^{7}	Rb2	O22^{4}	73.70(6)	O13	S1	Rb2^{7}	90.74(9)
O23^{7}	Rb2	O1^{5}	76.14(7)	O13	S1	Rb2	34.32(9)
O13	Rb2	S2^{4}	151.42(5)	O13	S1	O11	112.49(15)
O13	Rb2	S3^{6}	113.19(5)	O13	S1	C1	106.51(13)
O13	Rb2	S1	16.49(5)	C1	S1	Rb3	77.41(9)
O13	Rb2	S1^{5}	101.98(4)	C1	S1	Rb3^{11}	135.67(9)
O13	Rb2	O33^{6}	121.33(6)	C1	S1	Rb2	84.50(9)
O13	Rb2	O32^{6}	92.02(6)	C1	S1	Rb2^{7}	137.86(9)
O13	Rb2	O22^{4}	152.89(6)	Rb3	O33	Rb2^{2}	101.93(7)
O13	Rb2	O1^{5}	74.00(7)	Rb3	O33	Rb1	101.96(6)
O22^{4}	Rb2	S2^{4}	17.81(4)	Rb1	O33	Rb2^{2}	96.24(6)
O22^{4}	Rb2	S3^{6}	68.31(4)	S3	O33	Rb3	131.02(12)
O22^{4}	Rb2	S1	162.46(4)	S3	O33	Rb2^{2}	98.18(10)
O22^{4}	Rb2	S1^{5}	103.64(4)	S3	O33	Rb1	119.67(12)
O22^{4}	Rb2	O33^{6}	71.88(6)	Rb2^{7}	O12	Rb3	88.46(5)
O22^{4}	Rb2	O32^{6}	81.43(6)	S1	O12	Rb3	124.71(11)
O22^{4}	Rb2	O1^{5}	100.91(7)	S1	O12	Rb2^{7}	131.76(12)
O1^{5}	Rb2	S2^{4}	84.92(5)	Rb2^{2}	O32	Rb3^{6}	81.41(5)
O1^{5}	Rb2	S3^{6}	168.17(6)	Rb1^{2}	O32	Rb3^{6}	130.92(7)
O1^{5}	Rb2	S1	65.86(5)	Rb1^{2}	O32	Rb2^{2}	93.97(6)
O1^{5}	Rb2	S1^{5}	82.05(6)	S3	O32	Rb3^{6}	88.09(10)
O1^{5}	Rb2	O33^{6}	159.10(7)	S3	O32	Rb2^{2}	92.70(10)
O1^{5}	Rb2	O32^{6}	153.92(7)	S3	O32	Rb1^{2}	140.98(12)
S2^{6}	Rb1	Rb2^{2}	58.258(13)	Rb2	O31	Rb3^{6}	107.38(7)
S2^{6}	Rb1	S3	86.013(17)	S3	O31	Rb3^{6}	110.00(11)
S3	Rb1	Rb2^{2}	50.450(12)	S3	O31	Rb2	142.36(12)
O33	Rb1	Rb2^{2}	43.14(4)	Rb2^{5}	O23	Rb3^{9}	91.09(5)

O33	Rb1	S2^{6}	93.03(5)	Rb2^{5}	O23	Rb1^{2}	105.19(6)
O33	Rb1	S3	19.39(4)	Rb1^{2}	O23	Rb3^{9}	111.81(7)
O33	Rb1	O32^{6}	109.10(6)	S2	O23	Rb3^{9}	91.05(10)
O33	Rb1	O23^{6}	112.17(6)	S2	O23	Rb2^{5}	146.38(13)
O33	Rb1	O21^{3}	110.80(6)	S2	O23	Rb1^{2}	105.08(10)
O33	Rb1	O11^{7}	125.42(6)	Rb3^{9}	O21	Rb1^{11}	96.84(6)
O33	Rb1	O13	57.96(6)	S2	O21	Rb3^{9}	97.51(10)
O33	Rb1	O22^{6}	70.11(6)	S2	O21	Rb1^{11}	139.35(13)
O33	Rb1	O1	81.05(6)	Rb3^{11}	O11	Rb1^{5}	94.01(6)
O33	Rb1	O1^{8}	170.94(6)	S1	O11	Rb3^{11}	126.13(12)
O32^{6}	Rb1	Rb2^{2}	103.96(4)	S1	O11	Rb1^{5}	139.85(12)
O32^{6}	Rb1	S2^{6}	56.97(4)	Rb2	O13	Rb1	87.38(6)
O32^{6}	Rb1	S3	90.17(4)	S1	O13	Rb2	129.18(13)
O32^{6}	Rb1	O23^{6}	64.06(6)	S1	O13	Rb1	125.95(12)
O32^{6}	Rb1	O21^{3}	135.60(6)	Rb3	O22	Rb2^{10}	93.78(6)
O32^{6}	Rb1	O11^{7}	99.73(6)	Rb3	O22	Rb1^{2}	123.60(7)
O32^{6}	Rb1	O13	85.28(6)	Rb2^{10}	O22	Rb1^{2}	90.57(6)
O32^{6}	Rb1	O22^{6}	62.60(6)	S2	O22	Rb3	127.65(12)
O32^{6}	Rb1	O1^{8}	63.73(6)	S2	O22	Rb2^{10}	123.74(11)
O32^{6}	Rb1	O1	153.62(6)	S2	O22	Rb1^{2}	93.82(10)
O23^{6}	Rb1	Rb2^{2}	71.64(4)	Rb3	O1	Rb1^{12}	90.49(7)
O23^{6}	Rb1	S2^{6}	22.92(4)	Rb3	O1	Rb1	87.44(7)
O23^{6}	Rb1	S3	108.21(4)	Rb2^{7}	O1	Rb3	84.86(6)
O23^{6}	Rb1	O21^{3}	83.29(6)	Rb2^{7}	O1	Rb1	118.28(9)
O23^{6}	Rb1	O11^{7}	122.06(6)	Rb2^{7}	O1	Rb1^{12}	93.63(8)
O23^{6}	Rb1	O13	143.56(5)	Rb1^{12}	O1	Rb1	147.64(8)
O23^{6}	Rb1	O22^{6}	45.98(5)	S2	C1	S1	112.34(15)
O23^{6}	Rb1	O1^{8}	70.53(6)	S3	C1	S2	113.94(15)
O23^{6}	Rb1	O1	135.94(6)	S3	C1	S1	112.83(15)

^{1}-1/2+X,1/2-Y,1-Z; ^{2}1-X,-1/2+Y,3/2-Z; ^{3}1/2-X,1-Y,1/2+Z; ^{4}+X,1+Y,+Z; ^{5}1/2+X,3/2-Y,1-Z; ^{6}1-X,1/2+Y,3/2-Z; ^{7}-1/2+X,3/2-Y,1-Z; ^{8}-X,1/2+Y,3/2-Z; ^{9}1/2+X,1/2-Y,1-Z; ^{10}+X,-1+Y,+Z; ^{11}1/2-X,1-Y,-1/2+Z; ^{12}-X,-1/2+Y,3/2-Z

Table S97: Torsion Angles for $Rb_3[HC(SO_3)_3](H_2O)$.

A	B	C	D	Angle/°	A	B	C	D	Angle/°
Rb3	S2	O23	Rb3^{1}	119.93(11)	Rb1	S3	O32	Rb2^{3}	11.83(14)
Rb3	S2	O23	Rb2^{2}	-146.53(13)	Rb1	S3	O32	Rb1^{3}	111.52(17)
Rb3^{1}	S2	O23	Rb2^{2}	93.5(2)	Rb1	S3	O31	Rb3^{6}	109.90(9)
Rb3	S2	O23	Rb1^{3}	6.98(19)	Rb1	S3	O31	Rb2	-77.10(18)
Rb3^{1}	S2	O23	Rb1^{3}	-112.94(9)	Rb1	S3	C1	S2	-124.65(12)
Rb3	S2	O21	Rb3^{1}	-128.08(5)	Rb1	S3	C1	S1	5.01(18)
Rb3^{1}	S2	O21	Rb1^{4}	109.7(2)	O33	S3	O32	Rb3^{6}	-111.59(11)
Rb3	S2	O21	Rb1^{4}	-18.34(19)	O33	S3	O32	Rb2^{3}	-30.28(12)
Rb3^{1}	S2	O22	Rb3	-138.50(10)	O33	S3	O32	Rb1^{3}	69.4(2)
Rb3	S2	O22	Rb2^{5}	128.0(2)	O33	S3	O31	Rb3^{6}	106.49(13)
Rb3^{1}	S2	O22	Rb2^{5}	-10.51(14)	O33	S3	O31	Rb2	-80.5(2)
Rb3	S2	O22	Rb1^{3}	-138.76(15)	O33	S3	C1	S2	-81.47(17)
Rb3^{1}	S2	O22	Rb1^{3}	82.74(5)	O33	S3	C1	S1	48.18(19)
Rb3^{1}	S2	C1	S3	-142.90(10)	O12	S1	O11	Rb3^{4}	43.7(2)
Rb3	S2	C1	S3	62.91(13)	O12	S1	O11	Rb1^{2}	-138.07(18)
Rb3	S2	C1	S1	-66.99(13)	O12	S1	O13	Rb2	169.39(13)
Rb3^{1}	S2	C1	S1	87.2(2)	O12	S1	O13	Rb1	48.17(16)
Rb3^{6}	S3	O33	Rb3	-157.74(9)	O12	S1	C1	S2	40.94(18)
Rb3	S3	O33	Rb2^{3}	113.70(16)	O12	S1	C1	S3	-89.52(17)

Rb3^{6}	S3	O33	Rb2^{3}	-44.04(10)	O32	S3	O33	Rb3	-81.62(18)
Rb3	S3	O33	Rb1	-144.3(2)	O32	S3	O33	Rb2^{3}	32.08(13)
Rb3^{6}	S3	O33	Rb1	57.99(13)	O32	S3	O33	Rb1	134.11(12)
Rb3	S3	O32	Rb3^{6}	-145.08(3)	O32	S3	O31	Rb3^{6}	-22.80(15)
Rb3	S3	O32	Rb2^{3}	-63.78(6)	O32	S3	O31	Rb2	150.19(18)
Rb3^{6}	S3	O32	Rb2^{3}	81.31(5)	O32	S3	C1	S2	39.53(18)
Rb3^{6}	S3	O32	Rb1^{3}	-179.0(2)	O32	S3	C1	S1	169.18(14)
Rb3	S3	O32	Rb1^{3}	35.91(19)	O31	S3	O33	Rb3	149.24(14)
Rb3	S3	O31	Rb3^{6}	131.58(9)	O31	S3	O33	Rb2^{3}	-97.05(12)
Rb3	S3	O31	Rb2	-55.4(3)	O31	S3	O33	Rb1	4.97(17)
Rb3^{6}	S3	O31	Rb2	173.0(3)	O31	S3	O32	Rb3^{6}	18.02(12)
Rb3	S3	C1	S2	-62.85(13)	O31	S3	O32	Rb2^{3}	99.32(11)
Rb3^{6}	S3	C1	S2	115.10(13)	O31	S3	O32	Rb1^{3}	-160.99(17)
Rb3^{6}	S3	C1	S1	-115.25(13)	O31	S3	C1	S2	158.52(14)
Rb3	S3	C1	S1	66.80(13)	O31	S3	C1	S1	-71.82(18)
Rb3^{4}	S1	O12	Rb3	177.70(11)	O23	S2	O21	Rb3^{1}	35.35(14)
Rb3^{4}	S1	O12	Rb2^{7}	-57.22(13)	O23	S2	O21	Rb1^{4}	145.09(16)
Rb3	S1	O12	Rb2^{7}	125.1(2)	O23	S2	O22	Rb3	154.08(13)
Rb3	S1	O11	Rb3^{4}	72.5(2)	O23	S2	O22	Rb2^{5}	-77.93(16)
Rb3^{4}	S1	O11	Rb1^{2}	178.2(3)	O23	S2	O22	Rb1^{3}	15.32(13)
Rb3	S1	O11	Rb1^{2}	-109.3(2)	O23	S2	C1	S3	-78.67(17)
Rb3	S1	O13	Rb2	132.10(12)	O23	S2	C1	S1	151.44(15)
Rb3^{4}	S1	O13	Rb2	-97.53(13)	O21	S2	O23	Rb3^{1}	-32.99(13)
Rb3^{4}	S1	O13	Rb1	141.25(9)	O21	S2	O23	Rb2^{2}	60.6(2)
Rb3	S1	O13	Rb1	10.88(12)	O21	S2	O23	Rb1^{3}	-145.93(11)
Rb3^{4}	S1	C1	S2	-61.2(2)	O21	S2	O22	Rb3	-77.79(17)
Rb3	S1	C1	S2	65.28(13)	O21	S2	O22	Rb2^{5}	50.20(17)
Rb3^{4}	S1	C1	S3	168.35(6)	O21	S2	O22	Rb1^{3}	143.45(10)
Rb3	S1	C1	S3	-65.18(13)	O21	S2	C1	S3	162.63(14)
Rb2^{5}	S2	O23	Rb3^{1}	57.48(5)	O21	S2	C1	S1	32.73(19)
Rb2^{5}	S2	O23	Rb2^{2}	151.02(19)	O11	S1	O12	Rb3	153.50(13)
Rb2^{5}	S2	O23	Rb1^{3}	-55.46(8)	O11	S1	O12	Rb2^{7}	-81.42(18)
Rb2^{5}	S2	O21	Rb3^{1}	-64.32(6)	O11	S1	O13	Rb2	-59.50(18)
Rb2^{5}	S2	O21	Rb1^{4}	45.42(17)	O11	S1	O13	Rb1	179.28(12)
Rb2^{5}	S2	O22	Rb3	-128.0(2)	O11	S1	C1	S2	-79.12(17)
Rb2^{5}	S2	O22	Rb1^{3}	93.25(12)	O11	S1	C1	S3	150.42(16)
Rb2^{5}	S2	C1	S3	55.0(2)	O13	S1	O12	Rb3	-76.08(16)
Rb2^{5}	S2	C1	S1	-74.9(2)	O13	S1	O12	Rb2^{7}	49.00(19)
Rb2^{3}	S3	O33	Rb3	-113.70(16)	O13	S1	O11	Rb3^{4}	-87.18(18)
Rb2^{3}	S3	O33	Rb1	102.02(12)	O13	S1	O11	Rb1^{2}	91.0(2)
Rb2^{3}	S3	O32	Rb3^{6}	-81.31(5)	O13	S1	C1	S2	162.46(15)
Rb2^{3}	S3	O32	Rb1^{3}	99.69(19)	O13	S1	C1	S3	32.00(19)
Rb2^{3}	S3	O31	Rb3^{6}	44.63(10)	O22	S2	O23	Rb3^{1}	95.64(11)
Rb2^{3}	S3	O31	Rb2	-142.37(16)	O22	S2	O23	Rb2^{2}	-170.82(18)
Rb2^{3}	S3	C1	S2	-26.2(3)	O22	S2	O23	Rb1^{3}	-17.30(14)
Rb2^{3}	S3	C1	S1	103.42(18)	O22	S2	O21	Rb3^{1}	-92.91(12)
Rb2	S1	O12	Rb3	-65.1(2)	O22	S2	O21	Rb1^{4}	16.8(2)
Rb2^{7}	S1	O12	Rb3	-125.1(2)	O22	S2	C1	S3	41.17(18)
Rb2	S1	O12	Rb2^{7}	59.9(2)	O22	S2	C1	S1	-88.73(17)
Rb2	S1	O11	Rb3^{4}	-116.29(14)	C1	S2	O23	Rb3^{1}	-147.55(10)
Rb2^{7}	S1	O11	Rb3^{4}	10.27(17)	C1	S2	O23	Rb2^{2}	-54.0(2)
Rb2	S1	O11	Rb1^{2}	61.91(19)	C1	S2	O23	Rb1^{3}	99.51(12)
Rb2^{7}	S1	O11	Rb1^{2}	-171.53(16)	C1	S2	O21	Rb3^{1}	148.99(10)

$Rb2^{7}$	S1	O13	Rb2	-166.69(13)	C1	S2	O21	$Rb1^{4}$	-101.27(19)
Rb2	S1	O13	Rb1	-121.2(2)	C1	S2	O22	Rb3	39.25(17)
$Rb2^{7}$	S1	O13	Rb1	72.09(11)	C1	S2	O22	$Rb2^{5}$	167.24(12)
$Rb2^{7}$	S1	C1	S2	52.1(2)	C1	S2	O22	$Rb1^{3}$	-99.51(10)
Rb2	S1	C1	S2	-170.91(14)	C1	S3	O33	Rb3	35.64(19)
Rb2	S1	C1	S3	58.63(13)	C1	S3	O33	$Rb2^{3}$	149.35(10)
$Rb2^{7}$	S1	C1	S3	-78.34(19)	C1	S3	O33	Rb1	-108.63(13)
$Rb1^{3}$	S2	O23	$Rb3^{1}$	112.94(9)	C1	S3	O32	$Rb3^{6}$	131.19(10)
$Rb1^{3}$	S2	O23	$Rb2^{2}$	-153.5(3)	C1	S3	O32	$Rb2^{3}$	-147.50(10)
$Rb1^{3}$	S2	O21	$Rb3^{1}$	-18.3(2)	C1	S3	O32	$Rb1^{3}$	-47.8(2)
$Rb1^{3}$	S2	O21	$Rb1^{4}$	91.4(2)	C1	S3	O31	$Rb3^{6}$	-137.98(11)
$Rb1^{3}$	S2	O22	Rb3	138.76(15)	C1	S3	O31	Rb2	35.0(2)
$Rb1^{3}$	S2	O22	$Rb2^{5}$	-93.25(12)	C1	S1	O12	Rb3	40.91(17)
$Rb1^{3}$	S2	C1	S3	-24.58(16)	C1	S1	O12	$Rb2^{7}$	165.99(14)
$Rb1^{3}$	S2	C1	S1	-154.47(11)	C1	S1	O11	$Rb3^{4}$	158.69(15)
Rb1	S3	O33	Rb3	144.3(2)	C1	S1	O11	$Rb1^{2}$	-23.1(2)
Rb1	S3	O33	$Rb2^{3}$	-102.02(12)	C1	S1	O13	Rb2	52.29(19)
Rb1	S3	O32	$Rb3^{6}$	-69.47(11)	C1	S1	O13	Rb1	-68.92(16)

[1]1/2+X,1/2-Y,1-Z; [2]1/2+X,3/2-Y,1-Z; [3]1-X,-1/2+Y,3/2-Z; [4]1/2-X,1-Y,-1/2+Z; [5]+X,-1+Y,+Z; [6]1-X,1/2+Y,3/2-Z; [7]-1/2+X,3/2-Y,1-Z

Table S98: Hydrogen Atom Coordinates (Å×10^4) and Isotropic Displacement Parameters (Å^2×10^3) for $Rb_3[HC(SO_3)_3](H_2O)$.

Atom	*x*	*y*	*z*	U(eq)
H1	6060(40)	6240(40)	5020(30)	21(10)
H1A	-540(60)	6030(60)	6720(40)	45(16)
H1B	-30(60)	5140(60)	7330(50)	52(18)

$Rb_3Ag_3[HC(SO_3)_3]_2$ (15)

Table S99: Crystallographic data of $Rb_3Ag_3[HC(SO_3)_3]_2$.

Empirical formula	$CHAg_3O_9Rb_3S_3$
Formula weight	543.21 g/mol
Temperature	100(2) K
Crystal system	triclinic
Space group	*P*-1 (No. 2)
Unit cell dimensions	*a* = 750.17(5) pm
	b = 754.08(5) pm
	c = 973.20(6) pm
	α = 89.447(2)°
	β = 76.436(2)°
	γ = 62.861(2)°
Volume	473.23(5) $Å^3$
Z	1
ρ_{calc}	3.812 g/cm^3
μ	11.492 mm^{-1}
F(000)	506
Radiation	MoK_α (λ = 0.71073 nm)
Crystal size	0.226 x 0.068 x 0.063 mm^3
2Θ range for data collection	4.332 to 54.986
Index ranges	$-9 \leq h \leq 9$, $-9 \leq k \leq 9$, $-12 \leq l \leq 12$
Reflections collected	67407
Independent reflections	2180 [R_{int} = 0.0404, R_σ = 0.00097]
Completeness	100%
Absorption correction	multiscan
Min. and max. transmission	0.544 / 0.748
Data/restraints/parameters	2180/0/153
Goodness-of-fit on F^2	1.140
Final *R* indexes [I≥2σ (I)]	R_1 = 0.0098, wR_2 = 0.0252
Final *R* indexes [all data]	R_1 = 0.0099, wR_2 = 0.0252
Largest diff. peak/hole	0.39/–0.39 e · $Å^{-3}$
CCDC-No.	2310269

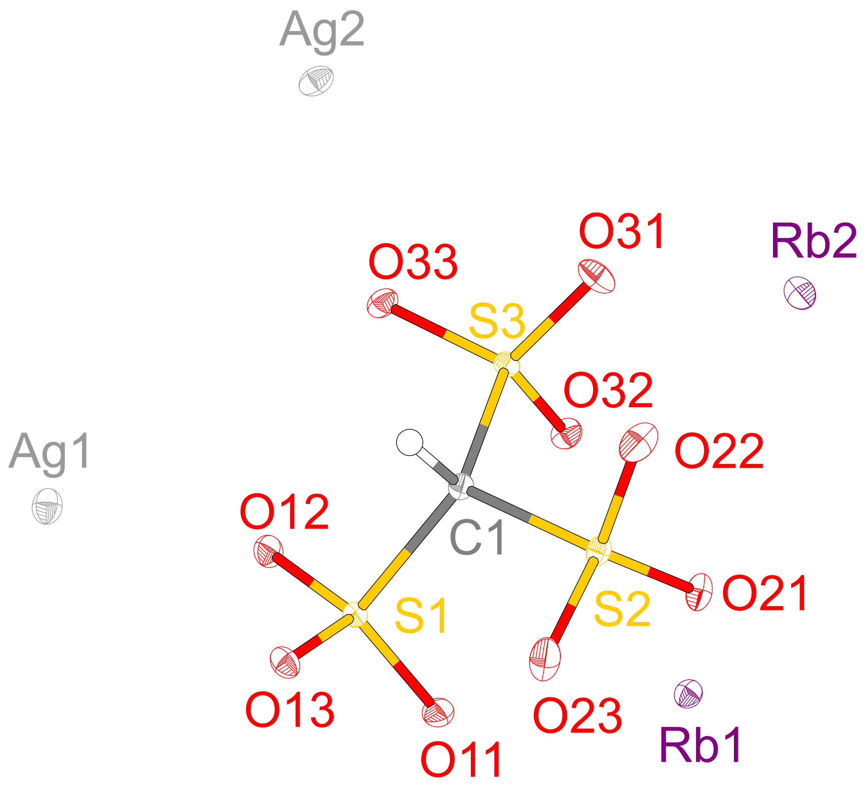

Figure S24: Thermal ellipsoid plot of the asymmetric unit of $Rb_3Ag_3[HC(SO_3)_3]_2$. Thermal ellipsoids shown with 50% probability.

Table S100: Fractional Atomic Coordinates (×10^4) and Equivalent Isotropic Displacement Parameters (Å^2×10^3) for $Rb_3Ag_3[HC(SO_3)_3]_2$. U_{eq} is defined as 1/3 of the trace of the orthogonalised U_{IJ} tensor.

Atom	*x*	*y*	*z*	U(eq)
Ag2	10000	5000	0	10.62(5)
Ag1	7197.2(2)	-259.5(2)	245.9(2)	11.60(4)
Rb1	1379.9(2)	6874.5(2)	6237.2(2)	8.56(4)
Rb2	5000	10000	5000	11.64(5)
S3	4841.9(6)	6656.6(6)	2229.0(4)	6.87(7)
S1	3181.5(6)	3625.5(5)	2445.9(4)	7.05(7)
S2	281.2(6)	8047.7(6)	2405.5(4)	7.84(8)
O32	4662.3(18)	6613.8(17)	3746.3(12)	9.8(2)
O21	-143.8(18)	8788.6(17)	3868.6(12)	11.1(2)
O11	2074.5(18)	4002.2(17)	3932.0(12)	10.9(2)
O13	2249.3(18)	2989.2(17)	1518.3(12)	10.2(2)
O33	6796.7(17)	5084.9(17)	1357.5(12)	9.9(2)
O22	228.9(19)	9522.3(19)	1415.1(13)	15.3(2)
O23	-1023.3(18)	7153.4(19)	2206.1(14)	16.4(3)
O12	5397.2(18)	2289.0(17)	2206.2(13)	11.2(2)
O31	4328.0(19)	8635.8(18)	1772.2(13)	13.0(2)
C1	2928(2)	6019(2)	1850.0(16)	7.4(3)

Table S101: Anisotropic Displacement Parameters (Å^2×10^3) for $Rb_3Ag_3[HC(SO_3)_3]_2$. The anisotropic displacement factor exponent takes the form: $-2\pi^2[h^2a^{*2}U_{11}+2hka^*b^*U_{12}+\ldots]$.

Atom	U_{11}	U_{22}	U_{33}	U_{23}	U_{13}	U_{12}
Ag2	7.76(8)	15.47(9)	9.04(8)	0.45(6)	-2.04(6)	-5.81(7)
Ag1	12.73(7)	11.13(6)	9.69(6)	0.14(4)	-3.16(4)	-4.39(5)

Rb1	8.45(7)	8.86(7)	7.72(7)	1.19(5)	-2.07(5)	-3.50(6)
Rb2	13.29(11)	10.29(10)	12.27(10)	1.54(8)	-3.78(8)	-6.07(8)
S3	7.04(16)	7.34(17)	7.46(17)	1.11(13)	-2.77(13)	-3.97(14)
S1	7.27(17)	6.62(16)	7.42(17)	0.50(13)	-1.72(13)	-3.46(14)
S2	6.02(16)	8.33(17)	7.71(17)	-0.45(13)	-2.35(13)	-1.83(14)
O32	10.1(5)	12.7(5)	7.3(5)	0.0(4)	-2.7(4)	-5.8(4)
O21	11.2(5)	9.7(5)	8.6(5)	-1.1(4)	-2.4(4)	-2.0(4)
O11	13.1(6)	11.9(5)	8.3(5)	1.3(4)	-1.4(4)	-7.0(5)
O13	12.7(6)	9.7(5)	10.6(5)	0.6(4)	-4.3(4)	-6.6(5)
O33	7.1(5)	12.5(5)	9.5(5)	-0.4(4)	-1.2(4)	-4.6(4)
O22	12.2(6)	14.4(6)	11.2(6)	5.8(5)	-2.1(4)	-0.1(5)
O23	7.9(5)	16.9(6)	22.9(7)	-8.1(5)	-3.2(5)	-4.7(5)
O12	7.8(5)	8.0(5)	15.9(6)	0.8(4)	-2.6(4)	-2.3(4)
O31	15.8(6)	9.4(5)	18.0(6)	5.5(4)	-8.1(5)	-7.6(5)
C1	6.5(7)	7.8(7)	7.6(7)	0.0(5)	-2.0(5)	-3.1(6)

Table S102: Bond lengths and interatomic distances for $Rb_3Ag_3[HC(SO_3)_3]_2$ in [pm].

Atom	Atom	Length/pm	Atom	Atom	Length/pm
Ag2	Ag1^{1}	319.06(2)	Rb1	O23^{11}	347.2(1)
Ag2	Ag1^{2}	319.06(2)	Rb1	O12^{3}	341.6(1)
Ag2	Rb1^{3}	399.21(2)	Rb2	O32	297.8(1)
Ag2	Rb1^{4}	399.21(2)	Rb2	O32^{12}	297.8(1)
Ag2	O13^{5}	247.4(1)	Rb2	O21^{6}	322.8(1)
Ag2	O13^{6}	247.4(1)	Rb2	O21^{10}	322.8(1)
Ag2	O33	242.8(1)	Rb2	O11^{2}	315.8(1)
Ag2	O33^{7}	242.8(1)	Rb2	O11^{3}	315.8(1)
Ag2	O23^{6}	244.8(1)	Rb2	O23^{6}	337.1(1)
Ag2	O23^{5}	244.8(1)	Rb2	O23^{10}	337.1(1)
Ag1	Ag1^{8}	328.77(3)	Rb2	O12^{2}	323.4(1)
Ag1	Rb1^{9}	423.92(3)	Rb2	O12^{3}	323.4(1)
Ag1	O13^{8}	251.2(1)	Rb2	O31^{12}	353.0(1)
Ag1	O22^{5}	248.0(1)	Rb2	O31	352.30(1)
Ag1	O12	237.7(1)	S3	O32	145.2(12
Ag1	O31^{5}	252.9(1)	S3	O33	145.8(1)
Rb1	S3^{3}	351.78(4)	S3	O31	145.5(1)
Rb1	O32^{3}	293.8(1)	S3	C1	182.0(2)
Rb1	O32	295.7(1)	S1	O11	144.6(1)
Rb1	O21^{10}	297.3(1)	S1	O13	146.5(1)
Rb1	O21	289.3(1)	S1	O12	145.9(1)
Rb1	O11	291.7(1)	S1	C1	183.0(2)
Rb1	O11^{11}	299.1(1)	S2	O21	144.3(1)
Rb1	O13^{11}	302.6(1)	S2	O22	145.8(1)
Rb1	O33^{3}	302.1(1)	S2	O23	146.4(1)
Rb1	O22^{10}	314.6(1)	S2	C1	182.1(2)

^{1}2-X,-Y,-Z; ^{2}+X,1+Y,+Z; ^{3}1-X,1-Y,1-Z; ^{4}1+X,+Y,-1+Z; ^{5}1-X,1-Y,-Z; ^{6}1+X,+Y,+Z; ^{7}2-X,1-Y,-Z; ^{8}1-X,-Y,-Z; ^{9}1+X,-1+Y,-1+Z; ^{10}-X,2-Y,1-Z; ^{11}-X,1-Y,1-Z; ^{12}1-X,2-Y,1-Z

Table S103: Bond Angles for $Rb_3Ag_3[HC(SO_3)_3]_2$.

Atom	Atom	Atom	Angle/°	Atom	Atom	Atom	Angle/°
Ag1^{1}	Ag2	Ag1^{2}	180.0	O32^{13}	Rb2	O12^{4}	99.50(3)
Ag1^{1}	Ag2	Rb1^{3}	108.650(5)	O32^{13}	Rb2	O12^{2}	80.50(3)
Ag1^{1}	Ag2	Rb1^{4}	71.350(5)	O32	Rb2	O12^{4}	80.50(3)
Ag1^{2}	Ag2	Rb1^{3}	71.350(5)	O32^{13}	Rb2	O31^{13}	42.79(3)
Ag1^{2}	Ag2	Rb1^{4}	108.650(5)	O32	Rb2	O31	42.79(3)
Rb1^{4}	Ag2	Rb1^{3}	180.0	O32^{13}	Rb2	O31	137.21(3)
O13^{5}	Ag2	Ag1^{2}	50.74(3)	O32	Rb2	O31^{13}	137.21(3)
O13^{6}	Ag2	Ag1^{1}	50.74(3)	O21^{11}	Rb2	O21^{6}	180.0
O13^{6}	Ag2	Ag1^{2}	129.26(3)	O21^{6}	Rb2	O23^{11}	137.05(3)
O13^{5}	Ag2	Ag1^{1}	129.26(3)	O21^{11}	Rb2	O23^{6}	137.05(3)
O13^{5}	Ag2	Rb1^{4}	130.79(3)	O21^{6}	Rb2	O23^{6}	42.95(3)
O13^{6}	Ag2	Rb1^{3}	130.79(3)	O21^{11}	Rb2	O23^{11}	42.95(3)
O13^{5}	Ag2	Rb1^{3}	49.21(3)	O21^{6}	Rb2	O12^{4}	105.86(3)
O13^{6}	Ag2	Rb1^{4}	49.21(3)	O21^{11}	Rb2	O12^{2}	105.86(3)
O13^{5}	Ag2	O13^{6}	180.00(4)	O21^{11}	Rb2	O12^{4}	74.14(3)
O33^{7}	Ag2	Ag1^{1}	83.50(3)	O21^{6}	Rb2	O12^{2}	74.14(3)
O33	Ag2	Ag1^{2}	83.50(3)	O21^{6}	Rb2	O31	97.32(3)
O33^{7}	Ag2	Ag1^{2}	96.50(3)	O21^{11}	Rb2	O31	82.68(3)
O33	Ag2	Ag1^{1}	96.50(3)	O21^{11}	Rb2	O31^{13}	97.32(3)
O33^{7}	Ag2	Rb1^{3}	49.05(3)	O21^{6}	Rb2	O31^{13}	82.68(3)
O33	Ag2	Rb1^{4}	49.05(3)	O11^{2}	Rb2	O21^{6}	113.24(3)
O33	Ag2	Rb1^{3}	130.95(3)	O11^{4}	Rb2	O21^{11}	113.24(3)
O33^{7}	Ag2	Rb1^{4}	130.95(3)	O11^{4}	Rb2	O21^{6}	66.76(3)
O33	Ag2	O13^{6}	97.81(4)	O11^{2}	Rb2	O21^{11}	66.76(3)
O33	Ag2	O13^{5}	82.19(4)	O11^{4}	Rb2	O11^{2}	180.0
O33^{7}	Ag2	O13^{5}	97.81(4)	O11^{4}	Rb2	O23^{6}	71.78(3)
O33^{7}	Ag2	O13^{6}	82.19(4)	O11^{2}	Rb2	O23^{11}	71.78(3)
O33^{7}	Ag2	O33	180.0	O11^{2}	Rb2	O23^{6}	108.22(3)
O33	Ag2	O23^{6}	74.82(4)	O11^{4}	Rb2	O23^{11}	108.22(3)
O33	Ag2	O23^{5}	105.18(4)	O11^{4}	Rb2	O12^{4}	44.42(3)
O33^{7}	Ag2	O23^{6}	105.18(4)	O11^{4}	Rb2	O12^{2}	135.58(3)
O33^{7}	Ag2	O23^{5}	74.82(4)	O11^{2}	Rb2	O12^{4}	135.58(3)
O23^{5}	Ag2	Ag1^{1}	58.34(3)	O11^{2}	Rb2	O12^{2}	44.42(3)
O23^{6}	Ag2	Ag1^{2}	58.34(3)	O11^{4}	Rb2	O31	107.01(3)
O23^{5}	Ag2	Ag1^{2}	121.66(3)	O11^{2}	Rb2	O31^{13}	107.01(3)
O23^{6}	Ag2	Ag1^{1}	121.66(3)	O11^{4}	Rb2	O31^{13}	72.99(3)
O23^{6}	Ag2	Rb1^{4}	59.65(3)	O11^{2}	Rb2	O31	72.99(3)
O23^{5}	Ag2	Rb1^{4}	120.35(3)	O23^{11}	Rb2	O23^{6}	180.0
O23^{6}	Ag2	Rb1^{3}	120.35(3)	O23^{6}	Rb2	O31	56.57(3)
O23^{5}	Ag2	Rb1^{3}	59.65(3)	O23^{11}	Rb2	O31^{13}	56.57(3)
O23^{5}	Ag2	O13^{5}	73.00(4)	O23^{6}	Rb2	O31^{13}	123.43(3)
O23^{6}	Ag2	O13^{5}	107.00(4)	O23^{11}	Rb2	O31	123.43(3)
O23^{6}	Ag2	O13^{6}	73.00(4)	O12^{4}	Rb2	O23^{11}	65.32(3)
O23^{5}	Ag2	O13^{6}	107.00(4)	O12^{4}	Rb2	O23^{6}	114.68(3)
O23^{5}	Ag2	O23^{6}	180.0	O12^{2}	Rb2	O23^{6}	65.32(3)

Ag2^{8}	Ag1	Ag1^{9}	103.790(6)	O12^{2}	Rb2	O23^{11}	114.68(3)
Ag2^{8}	Ag1	Rb1^{10}	63.160(4)	O12^{4}	Rb2	O12^{2}	180.0
Ag1^{9}	Ag1	Rb1^{10}	101.813(6)	O12^{4}	Rb2	O31^{13}	57.37(3)
O13^{9}	Ag1	Ag2^{8}	49.70(3)	O12^{2}	Rb2	O31^{13}	122.63(3)
O13^{9}	Ag1	Ag1^{9}	67.95(3)	O12^{2}	Rb2	O31	57.37(3)
O13^{9}	Ag1	Rb1^{10}	44.74(3)	O12^{4}	Rb2	O31	122.63(3)
O13^{9}	Ag1	O31^{5}	71.93(4)	O31^{13}	Rb2	O31	180.0
O22^{5}	Ag1	Ag2^{8}	97.31(3)	Rb1^{4}	S3	Rb2	78.840(9)
O22^{5}	Ag1	Ag1^{9}	126.43(3)	O32	S3	Rb1^{4}	55.06(5)
O22^{5}	Ag1	Rb1^{10}	47.42(3)	O32	S3	Rb2	47.19(5)
O22^{5}	Ag1	O13^{9}	91.72(4)	O32	S3	O33	113.45(7)
O22^{5}	Ag1	O31^{5}	71.89(4)	O32	S3	O31	113.33(7)
O12	Ag1	Ag2^{8}	133.01(3)	O32	S3	C1	107.10(7)
O12	Ag1	Ag1^{9}	91.21(3)	O33	S3	Rb1^{4}	58.40(5)
O12	Ag1	Rb1^{10}	156.25(3)	O33	S3	Rb2	118.57(5)
O12	Ag1	O13^{9}	157.07(4)	O33	S3	C1	103.10(7)
O12	Ag1	O22^{5}	108.97(4)	O31	S3	Rb1^{4}	136.79(5)
O12	Ag1	O31^{5}	104.54(4)	O31	S3	Rb2	69.41(5)
O31^{5}	Ag1	Ag2^{8}	120.64(3)	O31	S3	O33	113.15(7)
O31^{5}	Ag1	Ag1^{9}	54.91(3)	O31	S3	C1	105.64(7)
O31^{5}	Ag1	Rb1^{10}	68.65(3)	C1	S3	Rb1^{4}	117.57(5)
O32^{4}	Rb1	S3^{4}	23.91(2)	C1	S3	Rb2	136.77(5)
O32	Rb1	S3^{4}	90.28(2)	Rb1^{12}	S1	Rb2^{8}	81.410(9)
O32^{4}	Rb1	O32	70.14(4)	O11	S1	Rb1^{12}	55.94(5)
O32	Rb1	O21^{11}	80.37(3)	O11	S1	Rb2^{8}	57.72(5)
O32^{4}	Rb1	O21^{11}	132.38(3)	O11	S1	O13	113.39(7)
O32^{4}	Rb1	O11^{12}	116.09(3)	O11	S1	O12	112.65(7)
O32	Rb1	O11^{12}	124.17(3)	O11	S1	C1	107.70(7)
O32^{4}	Rb1	O13^{12}	112.89(3)	O13	S1	Rb1^{12}	57.45(5)
O32	Rb1	O13^{12}	171.84(3)	O13	S1	Rb2^{8}	111.72(5)
O32^{4}	Rb1	O33^{4}	48.17(3)	O13	S1	C1	103.40(7)
O32	Rb1	O33^{4}	110.95(3)	O12	S1	Rb1^{12}	134.73(5)
O32^{4}	Rb1	O22^{11}	115.90(3)	O12	S1	Rb2^{8}	60.82(5)
O32	Rb1	O22^{11}	115.10(3)	O12	S1	O13	112.41(7)
O32^{4}	Rb1	O23^{12}	64.18(3)	O12	S1	C1	106.50(7)
O32	Rb1	O23^{12}	125.94(3)	C1	S1	Rb1^{12}	118.75(5)
O32^{4}	Rb1	O12^{4}	63.21(3)	C1	S1	Rb2^{8}	144.87(5)
O32	Rb1	O12^{4}	77.82(3)	Rb1^{11}	S2	Rb2^{14}	71.567(8)
O21^{11}	Rb1	S3^{4}	128.28(2)	O21	S2	Rb1^{11}	53.47(5)
O21	Rb1	S3^{4}	153.68(2)	O21	S2	Rb2^{14}	56.90(5)
O21	Rb1	O32	65.84(3)	O21	S2	O22	113.58(7)
O21	Rb1	O32^{4}	129.90(3)	O21	S2	O23	112.70(7)
O21	Rb1	O21^{11}	61.10(4)	O21	S2	C1	108.87(7)
O21^{11}	Rb1	O11^{12}	111.34(3)	O22	S2	Rb1^{11}	60.50(5)
O21	Rb1	O11^{12}	73.32(3)	O22	S2	Rb2^{14}	108.97(5)
O21	Rb1	O11	67.29(3)	O22	S2	O23	111.48(8)
O21^{11}	Rb1	O13^{12}	101.61(3)	O22	S2	C1	104.38(7)
O21	Rb1	O13^{12}	108.01(3)	O23	S2	Rb1^{11}	127.53(5)
O21	Rb1	O33^{4}	176.46(3)	O23	S2	Rb2^{14}	62.72(5)
O21^{11}	Rb1	O33^{4}	117.47(3)	O23	S2	C1	105.09(7)
O21	Rb1	O22^{11}	103.77(3)	C1	S2	Rb1^{11}	127.37(5)
O21^{11}	Rb1	O22^{11}	46.63(3)	C1	S2	Rb2^{14}	146.65(5)
O21	Rb1	O23^{12}	129.11(3)	Rb1^{4}	O32	Rb1	109.85(4)

O21^{11}	Rb1	O23^{12}	153.38(3)	Rb1	O32	Rb2	93.31(3)
O21	Rb1	O12^{4}	125.86(3)	Rb1^{4}	O32	Rb2	103.02(3)
O21^{11}	Rb1	O12^{4}	74.79(3)	S3	O32	Rb1^{4}	101.04(6)
O11	Rb1	S3^{4}	94.58(2)	S3	O32	Rb1	134.34(6)
O11^{12}	Rb1	S3^{4}	115.68(2)	S3	O32	Rb2	111.85(6)
O11	Rb1	O32^{4}	75.50(3)	Rb1	O21	Rb1^{11}	118.90(4)
O11	Rb1	O32	68.72(3)	Rb1^{11}	O21	Rb2^{14}	88.13(3)
O11	Rb1	O21^{11}	127.13(3)	Rb1	O21	Rb2^{14}	100.73(3)
O11	Rb1	O11^{12}	61.11(4)	S2	O21	Rb1^{11}	103.58(6)
O11^{12}	Rb1	O13^{12}	47.69(3)	S2	O21	Rb1	132.40(6)
O11	Rb1	O13^{12}	104.27(3)	S2	O21	Rb2^{14}	101.12(6)
O11	Rb1	O33^{4}	113.34(3)	Rb1	O11	Rb1^{12}	118.89(4)
O11^{12}	Rb1	O33^{4}	110.12(3)	Rb1	O11	Rb2^{8}	99.24(3)
O11	Rb1	O22^{11}	168.54(3)	Rb1^{12}	O11	Rb2^{8}	100.22(3)
O11^{12}	Rb1	O22^{11}	110.24(3)	S1	O11	Rb1	132.08(6)
O11	Rb1	O23^{12}	73.11(3)	S1	O11	Rb1^{12}	100.45(6)
O11^{12}	Rb1	O23^{12}	59.90(3)	S1	O11	Rb2^{8}	99.50(6)
O11^{12}	Rb1	O12^{4}	157.41(3)	Ag2^{14}	O13	Ag1^{9}	79.56(3)
O11	Rb1	O12^{4}	133.63(3)	Ag2^{14}	O13	Rb1^{12}	92.53(4)
O13^{12}	Rb1	S3^{4}	94.56(2)	Ag1^{9}	O13	Rb1^{12}	99.49(4)
O13^{12}	Rb1	O22^{11}	70.94(3)	S1	O13	Ag2^{14}	128.69(7)
O13^{12}	Rb1	O23^{12}	53.03(3)	S1	O13	Ag1^{9}	145.51(7)
O13^{12}	Rb1	O12^{4}	110.34(3)	S1	O13	Rb1^{12}	98.47(6)
O33^{4}	Rb1	S3^{4}	24.27(2)	Ag2	O33	Rb1^{4}	93.58(4)
O33^{4}	Rb1	O13^{12}	75.34(3)	S3	O33	Ag2	134.06(7)
O33^{4}	Rb1	O22^{11}	76.05(3)	S3	O33	Rb1^{4}	97.34(5)
O33^{4}	Rb1	O23^{12}	53.75(3)	Ag1^{5}	O22	Rb1^{11}	97.10(4)
O33^{4}	Rb1	O12^{4}	51.01(3)	S2	O22	Ag1^{5}	136.60(7)
O22^{11}	Rb1	S3^{4}	96.17(2)	S2	O22	Rb1^{11}	95.72(6)
O22^{11}	Rb1	O23^{12}	109.96(3)	Ag2^{14}	O23	Rb1^{12}	82.87(3)
O22^{11}	Rb1	O12^{4}	57.15(3)	Ag2^{14}	O23	Rb2^{14}	144.84(5)
O23^{12}	Rb1	S3^{4}	56.06(2)	Rb2^{14}	O23	Rb1^{12}	85.15(3)
O12^{4}	Rb1	S3^{4}	53.56(2)	S2	O23	Ag2^{14}	118.75(7)
O12^{4}	Rb1	O23^{12}	104.48(3)	S2	O23	Rb1^{12}	127.78(7)
O32^{13}	Rb2	O32	180.0	S2	O23	Rb2^{14}	94.57(6)
O32^{13}	Rb2	O21^{6}	76.01(3)	Ag1	O12	Rb1^{4}	113.29(4)
O32	Rb2	O21^{11}	76.01(3)	Ag1	O12	Rb2^{8}	105.84(4)
O32	Rb2	O21^{6}	103.99(3)	Rb2^{8}	O12	Rb1^{4}	80.90(3)
O32^{13}	Rb2	O21^{11}	103.99(3)	S1	O12	Ag1	120.39(7)
O32	Rb2	O11^{4}	71.43(3)	S1	O12	Rb1^{4}	124.73(6)
O32	Rb2	O11^{2}	108.57(3)	S1	O12	Rb2^{8}	95.99(5)
O32^{13}	Rb2	O11^{4}	108.57(3)	Ag1^{5}	O31	Rb2	139.62(4)
O32^{13}	Rb2	O11^{2}	71.43(3)	S3	O31	Ag1^{5}	130.94(7)
O32	Rb2	O23^{11}	114.83(3)	S3	O31	Rb2	87.89(5)
O32	Rb2	O23^{6}	65.17(3)	S3	C1	S1	113.24(8)
O32^{13}	Rb2	O23^{11}	65.17(3)	S3	C1	S2	113.68(8)
O32^{13}	Rb2	O23^{6}	114.83(3)	S2	C1	S1	113.05(8)
O32	Rb2	O12^{2}	99.50(3)				

^{1}2-X,-Y,-Z; ^{2}+X,1+Y,+Z; ^{3}1+X,+Y,-1+Z; ^{4}1-X,1-Y,1-Z; ^{5}1-X,1-Y,-Z; ^{6}1+X,+Y,+Z; ^{7}2-X,1-Y,-Z; ^{8}+X,-1+Y,+Z; ^{9}1-X,-Y,-Z; ^{10}1+X,-1+Y,-1+Z; ^{11}-X,2-Y,1-Z; ^{12}-X,1-Y,1-Z; ^{13}1-X,2-Y,1-Z; ^{14}-1+X,+Y,+Z

Table S104: Torsion Angles for $Rb_3Ag_3[HC(SO_3)_3]_2$.

**A**	**B**	**C**	**D**	**Angle/°**	**A**	**B**	**C**	**D**	**Angle/°**

Rb1^{1}	S3	O32	Rb1	-132.41(10)	O13	S1	O11	Rb1	-146.50(8)
Rb1^{1}	S3	O32	Rb2	108.99(6)	O13	S1	O11	Rb1^{3}	-0.58(7)
Rb1^{1}	S3	O33	Ag2	-102.25(9)	O13	S1	O11	Rb2^{4}	101.76(6)
Rb1^{1}	S3	O31	Ag1^{2}	146.84(5)	O13	S1	O12	Ag1	9.11(10)
Rb1^{1}	S3	O31	Rb2	-45.43(7)	O13	S1	O12	Rb1^{1}	173.74(6)
Rb1^{1}	S3	C1	S1	3.64(10)	O13	S1	O12	Rb2^{4}	-103.20(6)
Rb1^{1}	S3	C1	S2	134.45(6)	O13	S1	C1	S3	-155.20(8)
Rb1^{3}	S1	O11	Rb1	-145.92(10)	O13	S1	C1	S2	73.68(9)
Rb1^{3}	S1	O11	Rb2^{4}	102.34(5)	O33	S3	O32	Rb1^{1}	-1.01(7)
Rb1^{3}	S1	O13	Ag2^{5}	100.17(8)	O33	S3	O32	Rb1	-133.41(8)
Rb1^{3}	S1	O13	Ag1^{6}	-120.74(13)	O33	S3	O32	Rb2	107.99(7)
Rb1^{3}	S1	O12	Ag1	74.86(9)	O33	S3	O31	Ag1^{2}	79.00(10)
Rb1^{3}	S1	O12	Rb1^{1}	-120.51(5)	O33	S3	O31	Rb2	-113.26(6)
Rb1^{3}	S1	O12	Rb2^{4}	-37.45(7)	O33	S3	C1	S1	64.59(9)
Rb1^{3}	S1	C1	S3	144.81(6)	O33	S3	C1	S2	-164.60(8)
Rb1^{3}	S1	C1	S2	13.69(10)	O22	S2	O21	Rb1^{7}	7.22(8)
Rb1^{7}	S2	O21	Rb1	-153.37(11)	O22	S2	O21	Rb1	-146.15(8)
Rb1^{7}	S2	O21	Rb2^{5}	90.76(5)	O22	S2	O21	Rb2^{5}	97.98(7)
Rb1^{7}	S2	O22	Ag1^{2}	106.66(11)	O22	S2	O23	Ag2^{5}	67.39(9)
Rb1^{7}	S2	O23	Ag2^{5}	135.99(4)	O22	S2	O23	Rb1^{3}	171.64(7)
Rb1^{7}	S2	O23	Rb1^{3}	-119.76(6)	O22	S2	O23	Rb2^{5}	-100.96(6)
Rb1^{7}	S2	O23	Rb2^{5}	-32.36(7)	O22	S2	C1	S3	70.67(10)
Rb1^{7}	S2	C1	S3	7.02(11)	O22	S2	C1	S1	-158.43(8)
Rb1^{7}	S2	C1	S1	137.93(6)	O23	S2	O21	Rb1	85.84(10)
Rb2	S3	O32	Rb1	118.60(10)	O23	S2	O21	Rb1^{7}	-120.79(7)
Rb2	S3	O32	Rb1^{1}	-108.99(6)	O23	S2	O21	Rb2^{5}	-30.03(8)
Rb2	S3	O33	Ag2	-48.67(10)	O23	S2	O22	Ag1^{2}	-131.37(10)
Rb2	S3	O33	Rb1^{1}	53.57(5)	O23	S2	O22	Rb1^{7}	121.97(6)
Rb2	S3	O31	Ag1^{2}	-167.73(10)	O23	S2	C1	S3	-171.90(9)
Rb2	S3	C1	S1	-100.04(8)	O23	S2	C1	S1	-40.99(10)
Rb2	S3	C1	S2	30.77(12)	O12	S1	O11	Rb1	84.41(10)
Rb2^{4}	S1	O11	Rb1	111.74(9)	O12	S1	O11	Rb1^{3}	-129.67(6)
Rb2^{4}	S1	O11	Rb1^{3}	-102.34(5)	O12	S1	O11	Rb2^{4}	-27.33(7)
Rb2^{4}	S1	O13	Ag2^{5}	163.74(5)	O12	S1	O13	Ag2^{5}	-130.05(8)
Rb2^{4}	S1	O13	Ag1^{6}	-57.16(13)	O12	S1	O13	Ag1^{6}	9.05(15)
Rb2^{4}	S1	O13	Rb1^{3}	63.57(4)	O12	S1	O13	Rb1^{3}	129.79(6)
Rb2^{4}	S1	O12	Ag1	112.31(7)	O12	S1	C1	S3	-36.56(10)
Rb2^{4}	S1	O12	Rb1^{1}	-83.06(6)	O12	S1	C1	S2	-167.68(8)
Rb2^{4}	S1	C1	S3	25.88(14)	O31	S3	O32	Rb1^{1}	-131.83(6)
Rb2^{4}	S1	C1	S2	-105.24(9)	O31	S3	O32	Rb1	95.76(9)
Rb2^{5}	S2	O21	Rb1	115.88(9)	O31	S3	O32	Rb2	-22.84(8)
Rb2^{5}	S2	O21	Rb1^{7}	-90.76(5)	O31	S3	O33	Ag2	29.64(12)
Rb2^{5}	S2	O22	Ag1^{2}	161.30(8)	O31	S3	O33	Rb1^{1}	131.89(6)
Rb2^{5}	S2	O22	Rb1^{7}	54.65(4)	O31	S3	C1	S1	-176.44(8)
Rb2^{5}	S2	O23	Ag2^{5}	168.35(9)	O31	S3	C1	S2	-45.63(10)
Rb2^{5}	S2	O23	Rb1^{3}	-87.40(7)	C1	S3	O32	Rb1^{1}	112.06(6)
Rb2^{5}	S2	C1	S3	-108.89(9)	C1	S3	O32	Rb1	-20.34(11)
Rb2^{5}	S2	C1	S1	22.02(15)	C1	S3	O32	Rb2	-138.94(6)
O32	S3	O33	Ag2	-101.28(10)	C1	S3	O33	Ag2	143.25(9)
O32	S3	O33	Rb1^{1}	0.97(7)	C1	S3	O33	Rb1^{1}	-114.50(6)
O32	S3	O31	Ag1^{2}	-150.02(8)	C1	S3	O31	Ag1^{2}	-33.06(11)
O32	S3	O31	Rb2	17.71(7)	C1	S3	O31	Rb2	134.67(5)
O32	S3	C1	S1	-55.34(10)	C1	S1	O11	Rb1^{3}	113.19(6)

O32	S3	C1	S2	75.47(10)	C1	S1	O11	Rb1	-32.73(10)
O21	S2	O22	Ag1^{2}	99.99(11)	C1	S1	O11	Rb2^{4}	-144.47(5)
O21	S2	O22	Rb1^{7}	-6.66(7)	C1	S1	O13	Ag2^{5}	-15.59(10)
O21	S2	O23	Ag2^{5}	-163.51(7)	C1	S1	O13	Ag1^{6}	123.51(12)
O21	S2	O23	Rb1^{3}	-59.26(9)	C1	S1	O13	Rb1^{3}	-115.76(6)
O21	S2	O23	Rb2^{5}	28.15(7)	C1	S1	O12	Ag1	-103.44(8)
O21	S2	C1	S3	-50.94(10)	C1	S1	O12	Rb1^{1}	61.19(8)
O21	S2	C1	S1	79.97(10)	C1	S1	O12	Rb2^{4}	144.24(6)
O11	S1	O13	Ag2^{5}	100.74(9)	C1	S2	O21	Rb1	-30.33(11)
O11	S1	O13	Ag1^{6}	-120.16(12)	C1	S2	O21	Rb1^{7}	123.04(6)
O11	S1	O13	Rb1^{3}	0.57(7)	C1	S2	O21	Rb2^{5}	-146.21(6)
O11	S1	O12	Ag1	138.71(7)	C1	S2	O22	Ag1^{2}	-18.44(13)
O11	S1	O12	Rb1^{1}	-56.66(9)	C1	S2	O22	Rb1^{7}	-125.10(6)
O11	S1	O12	Rb2^{4}	26.39(7)	C1	S2	O23	Ag2^{5}	-45.09(9)
O11	S1	C1	S3	84.51(9)	C1	S2	O23	Rb1^{3}	59.15(9)
O11	S1	C1	S2	-46.61(10)	C1	S2	O23	Rb2^{5}	146.56(5)

^{1}1-X,1-Y,1-Z; ^{2}1-X,1-Y,-Z; ^{3}-X,1-Y,1-Z; ^{4}+X,-1+Y,+Z; ^{5}-1+X,+Y,+Z; ^{6}1-X,-Y,-Z; ^{7}-X,2-Y,1-Z

Table S105: Hydrogen Atom Coordinates (Å×10^{4}) and Isotropic Displacement Parameters (Å^{2}×10^{3}) for $Rb_3Ag_3[HC(SO_3)_3]_2$.

Atom	*x*	*y*	*z*	U(eq)
H1	3220(30)	5830(30)	850(20)	7(5)

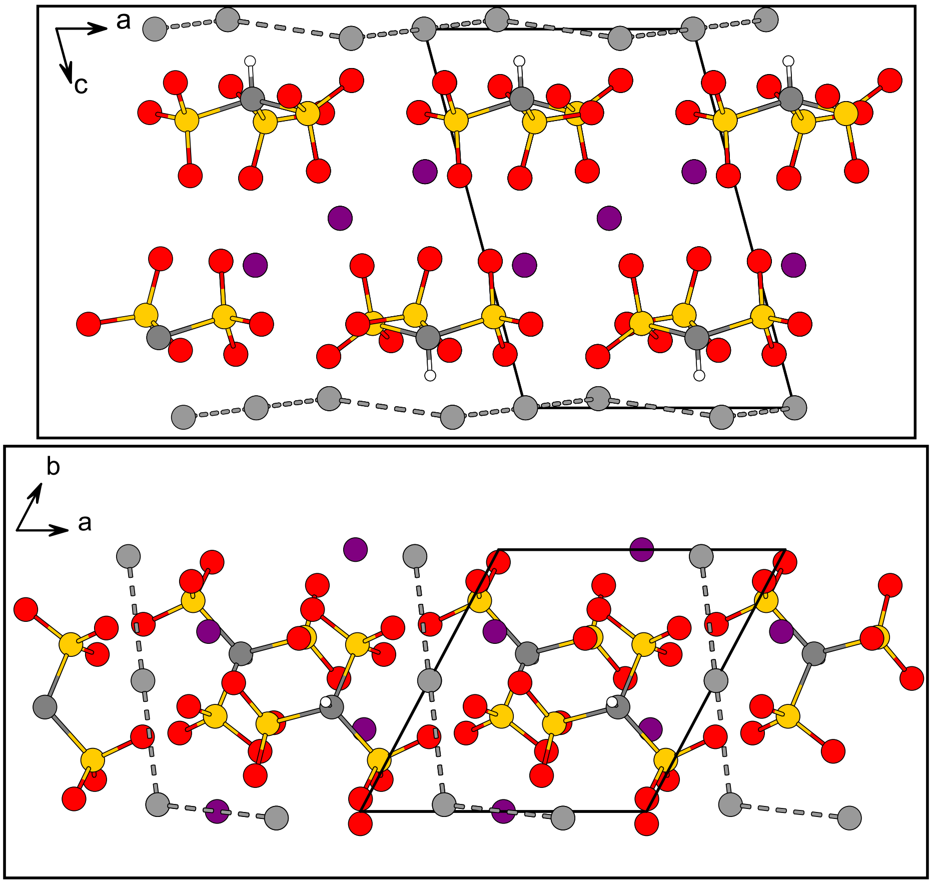

Figure S 25: Extended unit cells of $Rb_3Ag_3[HC(SO_3)_3]_2$., viewed along the crystallographic b-axis (top) and c-axis (bottom). Ag···Ag contacts are indicated by dotted grey lines.

$Rb_5Ag[HC(SO_3)_3]_2(H_2O)_2$ (16)

Table S106: Crystallographic data of $Rb_5Ag[HC(SO_3)_3]_2(H_2O)_2$.

Empirical formula	$C_2H_6AgO_{20}Rb_5S_6$
Formula weight	1077.65 g/mol
Temperature	100(2) K
Crystal system	Tetragonal
Space group	$P4_12_12$ (No. 92)
	a = 735.32(2) pm
Unit cell dimensions	b = 735.32(2) pm
	c = 4166.3(2) pm
Volume	2252.7(2) Å^3
Z	4
ρ_{calc}	3.177 g/cm^3
μ	12.275 mm^{-1}
F(000)	2024
Radiation	MoK_α (λ = 0.71073 nm)
Crystal size	0.186 x 0.185 x 0.056 mm^3
2Θ range for data collection	3.91 to 51.948
Index ranges	$-9 \leq h \leq 9$, $-8 \leq k \leq 8$, $-51 \leq l \leq 51$
Reflections collected	27796
Independent reflections	2208 [R_{int} = 0.0592, R_σ = 0.0287]
Completeness	100%
Absorption correction	multiscan
Min. and max. transmission	0.555 / 0.747
Data/restraints/parameters	2208/0/168
Goodness-of-fit on F^2	1.041
Final R indexes [I≥2σ (I)]	R_1 = 0.0177, wR_2 = 0.0367
Final R indexes [all data]	R_1 = 0.0188, wR_2 = 0.0372
Largest diff. peak/hole	0.49/–0.37 e · Å^{-3}
Flack parameter	0.057(8)
CCDC-No.	2310268

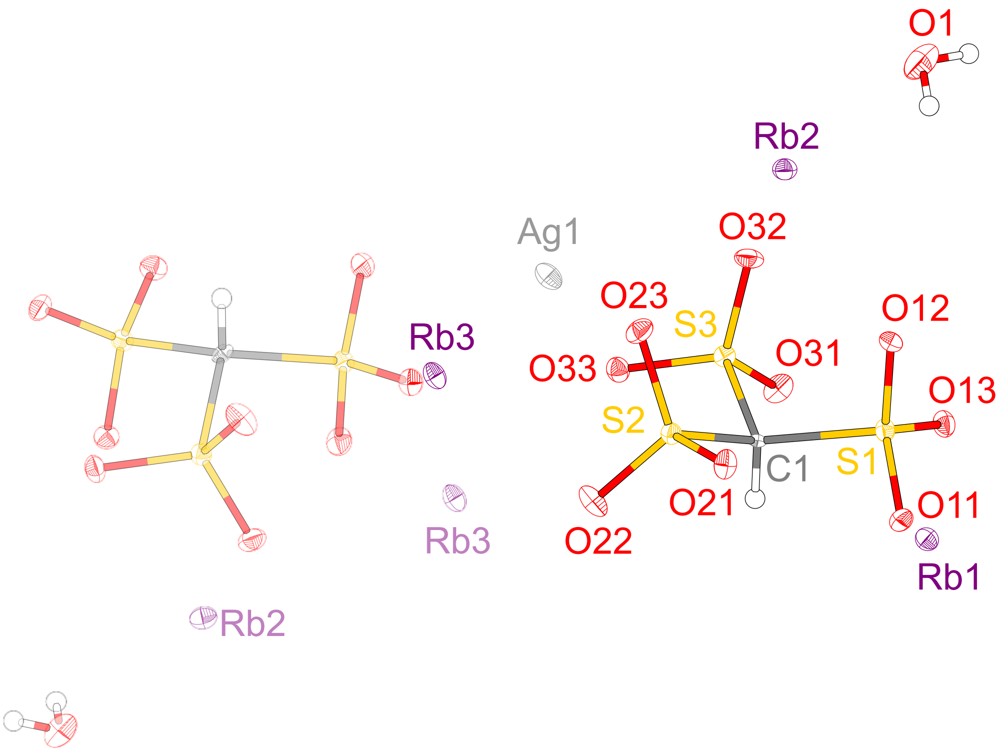

Figure S26: Thermal ellipsoid plot of the asymmetric unit of $Rb_5Ag[HC(SO_3)_3]_2(H_2O)_2$. Thermal ellipsoids shown with 50% probability. Atoms generated due to symmetry for representation of the complete ASU are shown at 50% visibility.

Table S107: Fractional Atomic Coordinates ($\times 10^4$) and Equivalent Isotropic Displacement Parameters ($Å^2\times 10^3$) for $Rb_5Ag[HC(SO_3)_3]_2(H_2O)_2$. U_{eq} is defined as 1/3 of the trace of the orthogonalised U_{IJ} tensor.

Atom	*x*	*y*	*z*	U(eq)
Ag1	607.8(5)	9392.2(5)	2500	12.27(12)
Rb1	2421.6(6)	-567.0(6)	3727.0(2)	10.14(10)
Rb3	-2695.6(6)	2695.6(6)	2500	10.81(13)
Rb2	5120.9(6)	7642.1(6)	2851.3(2)	11.08(10)
S1	2746.7(15)	4718.1(15)	3554.7(2)	8.8(2)
S3	2441.6(16)	2642.3(14)	2921.9(2)	8.4(2)
S2	128.7(16)	6007.0(15)	3039.1(3)	8.4(2)
O12	3918(4)	6164(4)	3434.2(7)	10.2(7)
O31	2827(5)	919(4)	3077.2(7)	13.8(7)
O21	29(4)	7410(4)	3284.7(7)	11.6(7)
O32	4027(4)	3651(4)	2819.0(7)	12.8(7)
O33	1095(4)	2441(4)	2663.5(7)	11.2(7)
O22	-1646(4)	5324(5)	2950.2(8)	15.4(8)
O23	1244(5)	6557(4)	2764.6(7)	13.1(7)
O11	1463(4)	5336(5)	3797.2(7)	11.6(7)
O13	3757(4)	3107(4)	3648.4(7)	11.9(7)
O1	7790(7)	10491(6)	3077.1(9)	25.6(9)
C1	1288(6)	4050(6)	3221.1(10)	6.9(9)

Table S108: Anisotropic Displacement Parameters ($Å^2\times 10^3$) for $Rb_5Ag[HC(SO_3)_3]_2(H_2O)_2$. The anisotropic displacement factor exponent takes the form: $-2\pi^2[h^2a^{*2}U_{11}+2hka^*b^*U_{12}+\ldots]$.

Atom	U_{11}	U_{22}	U_{33}	U_{23}	U_{13}	U_{12}
Ag1	9.18(16)	9.18(16)	18.5(2)	-1.01(15)	-1.01(15)	0.5(2)

Rb1	9.7(2)	9.4(2)	11.3(2)	-0.16(17)	-0.16(17)	-0.33(17)
Rb3	9.15(19)	9.15(19)	14.1(3)	-2.21(18)	-2.21(18)	1.1(3)
Rb2	9.6(2)	12.2(2)	11.4(2)	2.28(18)	1.07(17)	-0.14(19)
S1	9.4(6)	8.2(6)	8.9(5)	0.4(4)	-1.8(4)	-0.7(5)
S3	9.1(5)	7.2(5)	8.9(5)	-0.4(4)	0.4(4)	0.8(5)
S2	8.3(6)	8.1(6)	8.8(5)	-0.2(4)	-0.2(4)	1.1(4)
O12	7.7(17)	11.6(18)	11.4(15)	0.4(13)	0.4(13)	-2.0(13)
O31	19.8(19)	9.2(17)	12.3(16)	-0.5(13)	1.8(14)	3.0(15)
O21	15.1(17)	9.0(16)	10.8(15)	-2.7(13)	-2.4(12)	2.9(14)
O32	9.4(18)	15.4(18)	13.7(16)	-2.3(14)	4.6(13)	-1.4(14)
O33	10.1(16)	11.8(17)	11.8(15)	-0.9(14)	-0.8(12)	1.7(15)
O22	9.2(17)	12.8(19)	24.3(18)	-2.3(15)	-3.7(14)	-0.2(14)
O23	15.8(19)	12.9(18)	10.5(16)	3.5(14)	2.1(13)	2.7(15)
O11	12.3(17)	14.5(18)	8.0(15)	-1.0(14)	1.1(13)	-0.8(14)
O13	11.3(17)	11.6(17)	12.8(16)	2.0(13)	-2.8(13)	0.0(14)
O1	35(3)	28(2)	13.4(18)	1.2(17)	2.2(18)	-2(2)
C1	4(2)	11(3)	6(2)	1.3(18)	1.2(18)	-1.0(18)

Table S109: Bond lengths and interatomic distances for $Rb_5Ag[HC(SO_3)_3]_2(H_2O)_2$ in [pm].

Atom	Atom	Length/pm	Atom	Atom	Length/pm
Ag1	Rb3^{1}	3.4352(8)	Rb3	O22	2.802(3)
Ag1	Rb2	3.8486(6)	Rb3	O1^{2}	2.922(4)
Ag1	Rb2^{2}	3.8487(6)	Rb3	O1^{9}	2.922(4)
Ag1	O33^{3}	2.370(3)	Rb2	S3^{2}	3.6176(11)
Ag1	O33^{1}	2.370(3)	Rb2	S2	3.9412(12)
Ag1	O23^{2}	2.404(3)	Rb2	O12	2.804(3)
Ag1	O23	2.404(3)	Rb2	O31^{1}	3.088(3)
Rb1	S1^{4}	3.5486(12)	Rb2	O32	3.046(3)
Rb1	S1^{5}	3.6102(12)	Rb2	O32^{2}	3.181(3)
Rb1	O12^{5}	2.970(3)	Rb2	O33^{2}	2.946(3)
Rb1	O12^{4}	2.911(3)	Rb2	O22^{10}	2.954(3)
Rb1	O31	2.935(3)	Rb2	O23	2.982(3)
Rb1	O21^{4}	2.951(3)	Rb2	O23^{2}	2.963(3)
Rb1	O21^{6}	3.107(3)	Rb2	O1	3.021(4)
Rb1	O22^{6}	3.501(3)	S1	O12	1.457(3)
Rb1	O11^{4}	3.108(3)	S1	O11	1.455(3)
Rb1	O11^{6}	2.978(3)	S1	O13	1.452(3)
Rb1	O13	2.893(3)	S1	C1	1.823(5)
Rb1	O13^{5}	3.287(3)	S3	O31	1.451(3)
Rb3	S3^{3}	3.9846(12)	S3	O32	1.447(3)
Rb3	S2^{7}	3.9098(11)	S3	O33	1.470(3)
Rb3	S2	3.9098(11)	S3	C1	1.829(5)
Rb3	O32^{8}	2.840(3)	S2	O21	1.455(3)
Rb3	O32^{3}	2.840(3)	S2	O22	1.446(3)
Rb3	O33	2.876(3)	S2	O23	1.464(3)
Rb3	O33^{7}	2.876(3)	S2	C1	1.837(5)
Rb3	O22^{7}	2.802(3)			

1+X,1+Y,+Z; 2 1-Y,1-X,1/2-Z; 3 -Y,1-X,1/2-Z; 4 +X,-1+Y,+Z; 5 -1/2+X,1/2-Y,3/4-Z; 6 1/2+X,1/2-Y,3/4-Z; 7 -Y,-X,1/2-Z; 8 -1+X,+Y,+Z; 9 -1+X,-1+Y,+Z; 10 1+X,+Y,+Z

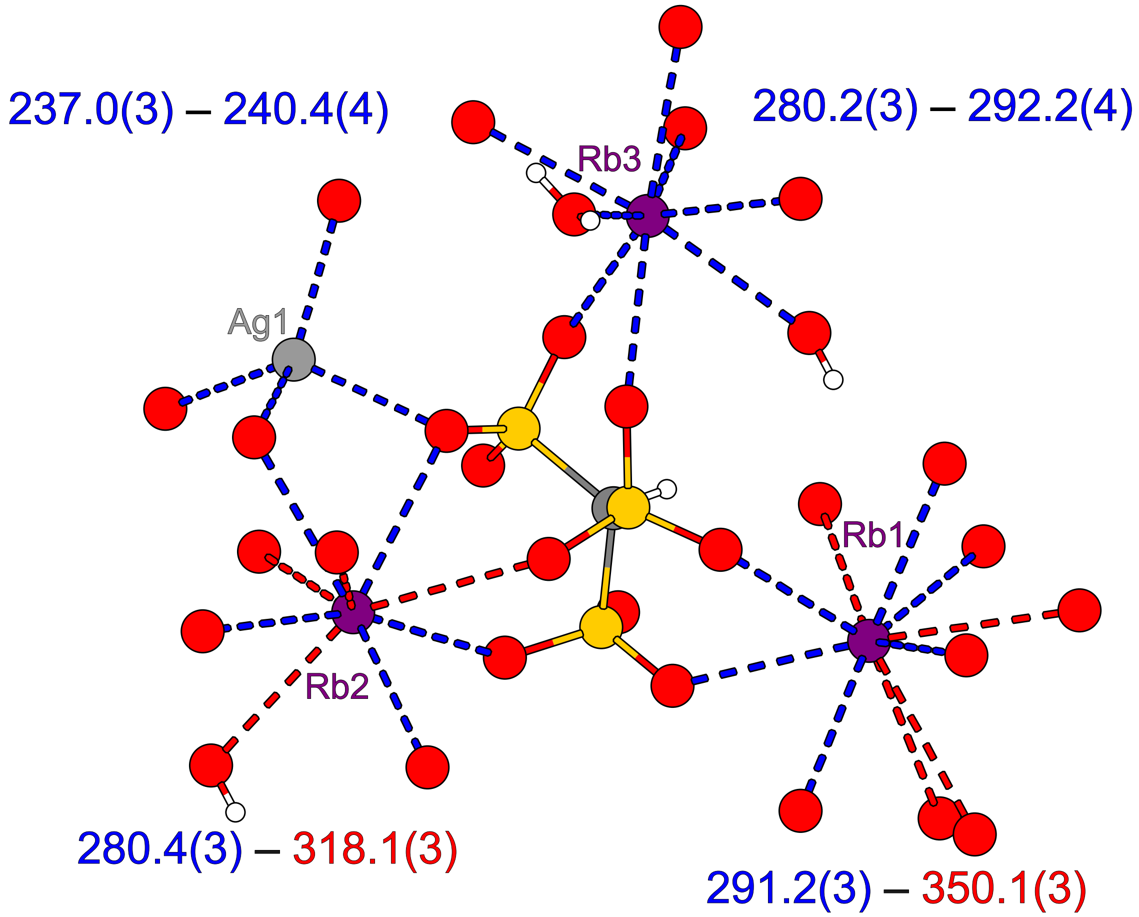

Figure S27: Coordination environment around the cations within compound **16**. Red, blue and orange dotted lines indicate interatomic distances above and below 300 pm and Ag···Ag distances, respectively. The shortest and longest interatomic distances, as well as the Ag···Ag distances are given in [pm].

Table S110: Bond Angles for $Rb_3Ag_3[HC(SO_3)_3]_2$.

Atom	Atom	Atom	Angle/°	Atom	Atom	Atom	Angle/°
Rb3[1]	Ag1	Rb2	147.799(8)	O12	Rb2	O23	72.52(9)
Rb3[1]	Ag1	Rb2[2]	147.798(8)	O12	Rb2	O1	101.77(10)
Rb2	Ag1	Rb2[2]	64.403(15)	O31[1]	Rb2	Ag1	51.98(6)
O33[3]	Ag1	Rb3[1]	55.82(8)	O31[1]	Rb2	S3[2]	122.21(6)
O33[1]	Ag1	Rb3[1]	55.82(8)	O31[1]	Rb2	S2	70.67(7)
O33[3]	Ag1	Rb2[2]	94.39(7)	O31[1]	Rb2	O32[2]	136.35(8)
O33[3]	Ag1	Rb2	150.96(8)	O32[2]	Rb2	Ag1	92.28(6)
O33[1]	Ag1	Rb2	94.39(8)	O32	Rb2	Ag1	94.45(6)
O33[1]	Ag1	Rb2[2]	150.96(8)	O32	Rb2	S3[2]	93.69(6)
O33[3]	Ag1	O33[1]	111.63(15)	O32[2]	Rb2	S3[2]	23.46(6)
O33[1]	Ag1	O23	131.21(10)	O32	Rb2	S2	57.92(6)
O33[3]	Ag1	O23[2]	131.21(10)	O32[2]	Rb2	S2	108.72(6)
O33[1]	Ag1	O23[2]	100.65(12)	O32	Rb2	O31[1]	128.40(9)
O33[3]	Ag1	O23	100.65(12)	O32	Rb2	O32[2]	70.39(10)
O23	Ag1	Rb3[1]	138.66(8)	O33[2]	Rb2	Ag1	98.19(6)
O23[2]	Ag1	Rb3[1]	138.66(8)	O33[2]	Rb2	S3[2]	23.10(6)
O23[2]	Ag1	Rb2	50.33(7)	O33[2]	Rb2	S2	143.86(6)
O23	Ag1	Rb2[2]	50.33(7)	O33[2]	Rb2	O31[1]	107.96(8)

O23	Ag1	Rb2	50.79(8)	O33^{2}	Rb2	O32^{2}	46.34(8)
O23^{2}	Ag1	Rb2^{2}	50.79(8)	O33^{2}	Rb2	O32	115.61(8)
O23^{2}	Ag1	O23	82.69(16)	O33^{2}	Rb2	O22^{10}	78.08(9)
S1^{4}	Rb1	S1^{5}	106.50(3)	O33^{2}	Rb2	O23^{2}	62.34(8)
O12^{4}	Rb1	S1^{5}	127.45(6)	O33^{2}	Rb2	O23	125.30(9)
O12^{5}	Rb1	S1^{5}	23.06(6)	O33^{2}	Rb2	O1	67.22(9)
O12^{4}	Rb1	S1^{4}	23.53(6)	O22^{10}	Rb2	Ag1	160.04(6)
O12^{5}	Rb1	S1^{4}	90.58(6)	O22^{10}	Rb2	S3^{2}	75.43(7)
O12^{4}	Rb1	O12^{5}	113.88(6)	O22^{10}	Rb2	S2	123.09(7)
O12^{4}	Rb1	O31	83.26(9)	O22^{10}	Rb2	O31^{1}	147.90(9)
O12^{4}	Rb1	O21^{6}	71.41(8)	O22^{10}	Rb2	O32^{2}	70.82(9)
O12^{5}	Rb1	O21^{6}	99.18(8)	O22^{10}	Rb2	O32	70.30(9)
O12^{4}	Rb1	O21^{4}	63.09(9)	O22^{10}	Rb2	O23	129.20(9)
O12^{5}	Rb1	O22^{6}	73.27(8)	O22^{10}	Rb2	O23^{2}	127.99(9)
O12^{4}	Rb1	O22^{6}	112.23(8)	O22^{10}	Rb2	O1	80.42(12)
O12^{5}	Rb1	O11^{6}	152.87(8)	O23	Rb2	Ag1	38.65(6)
O12^{4}	Rb1	O11^{6}	69.88(9)	O23^{2}	Rb2	Ag1	38.65(6)
O12^{4}	Rb1	O11^{4}	47.53(8)	O23^{2}	Rb2	S3^{2}	54.75(6)
O12^{5}	Rb1	O11^{4}	67.37(8)	O23	Rb2	S3^{2}	108.79(6)
O12^{4}	Rb1	O13^{5}	146.14(8)	O23	Rb2	S2	18.58(6)
O12^{5}	Rb1	O13^{5}	45.18(8)	O23^{2}	Rb2	S2	82.43(7)
O31	Rb1	S1^{4}	99.81(7)	O23	Rb2	O31^{1}	73.96(9)
O31	Rb1	S1^{5}	105.36(7)	O23^{2}	Rb2	O31^{1}	79.68(8)
O31	Rb1	O12^{5}	125.57(9)	O23^{2}	Rb2	O32^{2}	57.63(9)
O31	Rb1	O21^{4}	70.87(9)	O23^{2}	Rb2	O32	96.78(9)
O31	Rb1	O21^{6}	134.74(9)	O23	Rb2	O32	58.97(9)
O31	Rb1	O22^{6}	149.82(9)	O23	Rb2	O32^{2}	93.54(8)
O31	Rb1	O11^{4}	118.10(8)	O23^{2}	Rb2	O23	64.58(11)
O31	Rb1	O11^{6}	81.14(9)	O23	Rb2	O1	147.60(12)
O31	Rb1	O13^{5}	91.39(9)	O23^{2}	Rb2	O1	110.41(10)
O21^{6}	Rb1	S1^{4}	70.42(6)	O1	Rb2	Ag1	116.54(10)
O21^{4}	Rb1	S1^{4}	54.63(6)	O1	Rb2	S3^{2}	89.64(8)
O21^{6}	Rb1	S1^{5}	119.86(6)	O1	Rb2	S2	139.02(9)
O21^{4}	Rb1	S1^{5}	71.14(6)	O1	Rb2	O31^{1}	73.67(12)
O21^{4}	Rb1	O12^{5}	72.84(8)	O1	Rb2	O32	148.59(11)
O21^{4}	Rb1	O21^{6}	123.86(4)	O1	Rb2	O32^{2}	110.89(10)
O21^{4}	Rb1	O22^{6}	138.95(8)	Rb1^{6}	S1	Rb1	100.46(3)
O21^{6}	Rb1	O22^{6}	42.26(8)	Rb1^{1}	S1	Rb1^{6}	81.04(2)
O21^{4}	Rb1	O11^{6}	127.06(9)	Rb1^{1}	S1	Rb1	156.67(3)
O21^{6}	Rb1	O11^{4}	69.85(8)	O12	S1	Rb1^{6}	52.99(12)
O21^{4}	Rb1	O11^{4}	55.58(8)	O12	S1	Rb1^{1}	52.90(13)
O21^{4}	Rb1	O13^{5}	83.52(8)	O12	S1	Rb1	144.51(13)
O21^{6}	Rb1	O13^{5}	129.97(8)	O12	S1	C1	106.37(19)
O22^{6}	Rb1	S1^{5}	86.04(6)	O11	S1	Rb1^{1}	60.73(14)
O22^{6}	Rb1	S1^{4}	103.57(6)	O11	S1	Rb1	98.12(14)
O11^{6}	Rb1	S1^{4}	88.95(7)	O11	S1	Rb1^{6}	112.87(13)
O11^{4}	Rb1	S1^{4}	24.11(6)	O11	S1	O12	113.25(19)
O11^{6}	Rb1	S1^{5}	161.57(7)	O11	S1	C1	103.39(19)
O11^{4}	Rb1	S1^{5}	85.87(6)	O13	S1	Rb1^{6}	65.56(14)
O11^{6}	Rb1	O21^{6}	55.35(8)	O13	S1	Rb1	35.02(13)
O11^{4}	Rb1	O22^{6}	90.09(8)	O13	S1	Rb1^{1}	141.02(14)
O11^{6}	Rb1	O22^{6}	80.49(8)	O13	S1	O12	112.69(19)
O11^{6}	Rb1	O11^{4}	106.55(8)	O13	S1	O11	113.55(19)

O11^{6}	Rb1	O13^{5}	142.37(9)	O13	S1	C1	106.6(2)
O11^{4}	Rb1	O13^{5}	109.43(8)	C1	S1	Rb1^{1}	112.20(15)
O13^{5}	Rb1	S1^{4}	128.67(6)	C1	S1	Rb1^{6}	143.04(15)
O13^{5}	Rb1	S1^{5}	23.71(6)	C1	S1	Rb1	80.68(15)
O13	Rb1	S1^{4}	150.07(7)	Rb2^{2}	S3	Rb3^{10}	67.54(2)
O13	Rb1	S1^{5}	100.96(7)	O31	S3	Rb3^{10}	91.71(14)
O13	Rb1	O12^{5}	119.08(9)	O31	S3	Rb2^{2}	142.90(13)
O13	Rb1	O12^{4}	126.54(9)	O31	S3	O33	111.74(19)
O13	Rb1	O31	60.88(9)	O31	S3	C1	106.31(19)
O13	Rb1	O21^{6}	105.78(9)	O32	S3	Rb3^{10}	30.82(13)
O13	Rb1	O21^{4}	127.06(9)	O32	S3	Rb2^{2}	61.10(13)
O13	Rb1	O22^{6}	89.81(8)	O32	S3	O31	115.0(2)
O13^{5}	Rb1	O22^{6}	88.73(8)	O32	S3	O33	112.17(18)
O13	Rb1	O11^{6}	66.69(9)	O32	S3	C1	106.6(2)
O13	Rb1	O11^{4}	173.15(9)	O33	S3	Rb3^{10}	106.40(13)
O13	Rb1	O13^{5}	77.42(4)	O33	S3	Rb2^{2}	51.85(12)
Ag1^{4}	Rb3	S3^{3}	128.872(17)	O33	S3	C1	104.1(2)
Ag1^{4}	Rb3	S2^{7}	93.714(19)	C1	S3	Rb3^{10}	135.35(15)
Ag1^{4}	Rb3	S2	93.714(19)	C1	S3	Rb2^{2}	109.99(15)
S2^{7}	Rb3	S3^{3}	103.26(2)	Rb1^{5}	S2	Rb3	97.81(3)
S2	Rb3	S3^{3}	71.90(2)	Rb1^{5}	S2	Rb2^{8}	71.68(2)
S2^{7}	Rb3	S2	172.57(4)	Rb1^{5}	S2	Rb2	134.33(3)
O32^{8}	Rb3	Ag1^{4}	140.79(7)	Rb3	S2	Rb2^{8}	65.22(2)
O32^{3}	Rb3	Ag1^{4}	140.79(7)	Rb3	S2	Rb2	124.80(3)
O32^{8}	Rb3	S3^{3}	89.58(7)	Rb2	S2	Rb2^{8}	137.38(3)
O32^{3}	Rb3	S3^{3}	15.13(7)	O21	S2	Rb1^{5}	56.49(13)
O32^{8}	Rb3	S2^{7}	82.64(6)	O21	S2	Rb3	144.96(14)
O32^{3}	Rb3	S2^{7}	91.59(7)	O21	S2	Rb2^{8}	82.90(13)
O32^{3}	Rb3	S2	82.64(6)	O21	S2	Rb2	88.31(14)
O32^{8}	Rb3	S2	91.59(7)	O21	S2	O23	112.4(2)
O32^{3}	Rb3	O32^{8}	78.41(13)	O21	S2	C1	106.77(19)
O32^{3}	Rb3	O33^{7}	136.69(8)	O22	S2	Rb1^{5}	72.12(14)
O32^{3}	Rb3	O33	113.95(9)	O22	S2	Rb3	32.59(13)
O32^{8}	Rb3	O33^{7}	113.95(9)	O22	S2	Rb2^{8}	38.21(14)
O32^{8}	Rb3	O33	136.69(8)	O22	S2	Rb2	153.55(15)
O32^{3}	Rb3	O1^{2}	81.72(12)	O22	S2	O21	112.4(2)
O32^{8}	Rb3	O1^{2}	152.39(11)	O22	S2	O23	113.6(2)
O32^{8}	Rb3	O1^{9}	81.72(12)	O22	S2	C1	104.6(2)
O32^{3}	Rb3	O1^{9}	152.39(11)	O23	S2	Rb1^{5}	168.74(15)
O33^{7}	Rb3	Ag1^{4}	42.99(7)	O23	S2	Rb3	91.20(14)
O33	Rb3	Ag1^{4}	42.99(7)	O23	S2	Rb2	40.45(13)
O33^{7}	Rb3	S3^{3}	139.88(6)	O23	S2	Rb2^{8}	106.45(14)
O33	Rb3	S3^{3}	98.83(7)	O23	S2	C1	106.2(2)
O33	Rb3	S2^{7}	134.85(7)	C1	S2	Rb1^{5}	80.69(14)
O33^{7}	Rb3	S2^{7}	52.38(7)	C1	S2	Rb3	89.75(15)
O33^{7}	Rb3	S2	134.84(7)	C1	S2	Rb2	83.62(15)
O33	Rb3	S2	52.38(7)	C1	S2	Rb2^{8}	138.81(15)
O33	Rb3	O33^{7}	85.98(13)	Rb1^{1}	O12	Rb1^{6}	104.52(9)
O33^{7}	Rb3	O1^{9}	69.46(11)	Rb2	O12	Rb1^{6}	101.26(9)
O33	Rb3	O1^{9}	69.53(11)	Rb2	O12	Rb1^{1}	99.33(10)
O33^{7}	Rb3	O1^{2}	69.53(11)	S1	O12	Rb1^{1}	103.57(15)
O33	Rb3	O1^{2}	69.46(11)	S1	O12	Rb1^{6}	103.94(15)
O22	Rb3	Ag1^{4}	107.04(7)	S1	O12	Rb2	140.14(17)

O22[7]	Rb3	Ag1[4]	107.04(7)	Rb1	O31	Rb2[4]	92.63(9)
O22[7]	Rb3	S3[3]	87.64(7)	S3	O31	Rb1	135.80(18)
O22	Rb3	S3[3]	70.94(7)	S3	O31	Rb2[4]	130.72(16)
O22	Rb3	S2	16.14(7)	Rb1[1]	O21	Rb1[5]	100.31(8)
O22[7]	Rb3	S2	157.31(7)	S2	O21	Rb1[5]	100.53(16)
O22	Rb3	S2[7]	157.31(7)	S2	O21	Rb1[1]	140.06(18)
O22[7]	Rb3	S2[7]	16.14(7)	Rb3[10]	O32	Rb2	92.00(9)
O22[7]	Rb3	O32[3]	75.51(9)	Rb3[10]	O32	Rb2[2]	89.24(8)
O22	Rb3	O32[8]	75.51(9)	Rb2	O32	Rb2[2]	82.37(8)
O22	Rb3	O32[3]	78.23(9)	S3	O32	Rb3[10]	134.06(19)
O22[7]	Rb3	O32[8]	78.23(9)	S3	O32	Rb2[2]	95.44(15)
O22[7]	Rb3	O33[7]	67.62(9)	S3	O32	Rb2	133.93(18)
O22	Rb3	O33	67.62(9)	Ag1[4]	O33	Rb3	81.19(9)
O22[7]	Rb3	O33	144.04(9)	Ag1[4]	O33	Rb2[2]	114.45(11)
O22	Rb3	O33[7]	144.04(9)	Rb3	O33	Rb2[2]	95.36(9)
O22	Rb3	O22[7]	145.93(13)	S3	O33	Ag1[4]	114.06(18)
O22[7]	Rb3	O1[2]	78.36(11)	S3	O33	Rb3	145.06(17)
O22[7]	Rb3	O1[9]	118.83(11)	S3	O33	Rb2[2]	105.05(15)
O22	Rb3	O1[2]	118.83(11)	Rb3	O22	Rb1[5]	129.54(11)
O22	Rb3	O1[9]	78.36(11)	Rb3	O22	Rb2[8]	94.76(9)
O1[2]	Rb3	Ag1[4]	61.39(10)	Rb2[8]	O22	Rb1[5]	87.10(8)
O1[9]	Rb3	Ag1[4]	61.39(10)	S2	O22	Rb1[5]	84.73(15)
O1[2]	Rb3	S3[3]	75.00(10)	S2	O22	Rb3	131.26(19)
O1[9]	Rb3	S3[3]	149.29(9)	S2	O22	Rb2[8]	124.17(18)
O1[2]	Rb3	S2	104.72(9)	Ag1	O23	Rb2	90.56(10)
O1[9]	Rb3	S2	78.93(9)	Ag1	O23	Rb2[2]	91.02(9)
O1[9]	Rb3	S2[7]	104.72(9)	Rb2[2]	O23	Rb2	87.25(9)
O1[2]	Rb3	S2[7]	78.93(9)	S2	O23	Ag1	119.26(19)
O1[2]	Rb3	O1[9]	122.8(2)	S2	O23	Rb2	120.98(17)
Ag1	Rb2	S2	51.270(17)	S2	O23	Rb2[2]	135.74(19)
S3[2]	Rb2	Ag1	93.386(19)	Rb1[5]	O11	Rb1[1]	99.65(10)
S3[2]	Rb2	S2	126.52(3)	S1	O11	Rb1[1]	95.16(15)
O12	Rb2	Ag1	100.77(6)	S1	O11	Rb1[5]	129.90(17)
O12	Rb2	S3[2]	155.25(7)	Rb1	O13	Rb1[6]	140.73(11)
O12	Rb2	S2	54.26(7)	S1	O13	Rb1[6]	90.73(15)
O12	Rb2	O31[1]	82.31(9)	S1	O13	Rb1	128.24(18)
O12	Rb2	O32[2]	134.45(9)	Rb3[11]	O1	Rb2	92.81(11)
O12	Rb2	O32	65.26(8)	S1	C1	S3	113.5(2)
O12	Rb2	O33[2]	160.89(9)	S1	C1	S2	112.1(2)
O12	Rb2	O22[10]	84.81(9)	S3	C1	S2	112.2(2)
O12	Rb2	O23[2]	136.54(9)				

[1]+X,1+Y,+Z; [2]1-Y,1-X,1/2-Z; [3]-Y,1-X,1/2-Z; [4]+X,-1+Y,+Z; [5]-1/2+X,1/2-Y,3/4-Z; [6]1/2+X,1/2-Y,3/4-Z; [7]-Y,-X,1/2-Z; [8]-1+X,+Y,+Z; [9]-1+X,-1+Y,+Z; [10]1+X,+Y,+Z; [11]1+X,1+Y,+Z

Table S111: Torsion Angles for $Rb_5Ag[HC(SO_3)_3]_2(H_2O)_2$.

A	B	C	D	Angle/°	A	B	C	D	Angle/°
Rb1[1]	S1	O12	Rb1[2]	-109.00(15)	O31	S3	O32	Rb2	-137.0(2)
Rb1	S1	O12	Rb1[2]	-157.27(12)	O31	S3	O32	Rb2[5]	138.41(15)
Rb1[2]	S1	O12	Rb1[1]	109.00(15)	O31	S3	O33	Ag1[7]	-15.0(2)
Rb1	S1	O12	Rb1[1]	-48.3(2)	O31	S3	O33	Rb3	95.2(3)
Rb1[1]	S1	O12	Rb2	127.7(3)	O31	S3	O33	Rb2[5]	-141.14(16)
Rb1	S1	O12	Rb2	79.4(3)	O31	S3	C1	S1	67.9(3)
Rb1[2]	S1	O12	Rb2	-123.3(3)	O31	S3	C1	S2	-163.8(2)

Rb1	S1	O11	Rb1^{2}	169.54(6)	O21	S2	O22	Rb1^{3}	-40.52(17)
Rb1	S1	O11	Rb1^{3}	-83.3(2)	O21	S2	O22	Rb3	179.1(2)
Rb1^{2}	S1	O11	Rb1^{3}	107.2(2)	O21	S2	O22	Rb2^{4}	42.4(3)
Rb1^{1}	S1	O11	Rb1^{2}	64.52(11)	O21	S2	O23	Ag1	-52.7(3)
Rb1^{1}	S1	O11	Rb1^{3}	171.68(14)	O21	S2	O23	Rb2^{5}	-179.9(2)
Rb1^{2}	S1	O13	Rb1	141.28(13)	O21	S2	O23	Rb2	57.7(2)
Rb1^{1}	S1	O13	Rb1	174.8(2)	O21	S2	C1	S1	-24.6(3)
Rb1^{2}	S1	O13	Rb1^{1}	-33.5(2)	O21	S2	C1	S3	-153.7(2)
Rb1	S1	O13	Rb1^{1}	-174.8(2)	O32	S3	O31	Rb1	112.7(3)
Rb1	S1	C1	S3	-68.0(2)	O32	S3	O31	Rb2^{7}	-53.8(3)
Rb1^{2}	S1	C1	S3	132.28(19)	O32	S3	O33	Ag1^{7}	115.8(2)
Rb1^{1}	S1	C1	S3	27.1(4)	O32	S3	O33	Rb3	-133.9(3)
Rb1^{1}	S1	C1	S2	-101.3(3)	O32	S3	O33	Rb2^{5}	-10.3(2)
Rb1^{2}	S1	C1	S2	3.9(3)	O32	S3	C1	S1	-55.3(3)
Rb1	S1	C1	S2	163.6(2)	O32	S3	C1	S2	73.1(3)
Rb1^{3}	S2	O21	Rb1^{2}	120.4(3)	O33	S3	O31	Rb1	-118.0(2)
Rb1^{3}	S2	O22	Rb3	-140.3(3)	O33	S3	O31	Rb2^{7}	75.6(3)
Rb1^{3}	S2	O22	Rb2^{4}	82.88(17)	O33	S3	O32	Rb3^{6}	-84.9(3)
Rb1^{3}	S2	O23	Ag1	-42.5(8)	O33	S3	O32	Rb2	93.9(3)
Rb1^{3}	S2	O23	Rb2	68.0(8)	O33	S3	O32	Rb2^{5}	9.3(2)
Rb1^{3}	S2	O23	Rb2^{5}	-169.6(5)	O33	S3	C1	S1	-174.0(2)
Rb1^{3}	S2	C1	S1	-75.3(2)	O33	S3	C1	S2	-45.6(3)
Rb1^{3}	S2	C1	S3	155.6(2)	O22	S2	O21	Rb1^{3}	47.9(2)
Rb3^{6}	S3	O31	Rb1	133.6(2)	O22	S2	O21	Rb1^{2}	168.2(2)
Rb3^{6}	S3	O31	Rb2^{7}	-32.9(2)	O22	S2	O23	Ag1	76.4(2)
Rb3^{6}	S3	O32	Rb2^{5}	94.2(2)	O22	S2	O23	Rb2	-173.18(18)
Rb3^{6}	S3	O33	Ag1^{7}	83.65(14)	O22	S2	O23	Rb2^{5}	-50.8(3)
Rb3^{6}	S3	O33	Rb3	-166.1(3)	O22	S2	C1	S1	-143.9(2)
Rb3^{6}	S3	O33	Rb2^{5}	-42.47(14)	O22	S2	C1	S3	87.0(3)
Rb3^{6}	S3	C1	S1	-42.1(3)	O23	S2	O21	Rb1^{3}	177.61(15)
Rb3^{6}	S3	C1	S2	86.3(3)	O23	S2	O21	Rb1^{2}	-62.0(3)
Rb3	S2	O21	Rb1^{3}	48.7(2)	O23	S2	O22	Rb1^{3}	-169.64(16)
Rb3	S2	O21	Rb1^{2}	169.03(9)	O23	S2	O22	Rb3	50.0(3)
Rb3	S2	O22	Rb1^{3}	140.3(3)	O23	S2	O22	Rb2^{4}	-86.8(2)
Rb3	S2	O22	Rb2^{4}	-136.8(4)	O23	S2	C1	S1	95.6(3)
Rb3	S2	O23	Ag1	100.77(16)	O23	S2	C1	S3	-33.6(3)
Rb3	S2	O23	Rb2	-148.79(15)	O11	S1	O12	Rb1^{2}	-7.1(2)
Rb3	S2	O23	Rb2^{5}	-26.4(2)	O11	S1	O12	Rb1^{1}	101.88(18)
Rb3	S2	C1	S1	-173.3(2)	O11	S1	O12	Rb2	-130.4(3)
Rb3	S2	C1	S3	57.6(2)	O11	S1	O13	Rb1^{1}	-105.52(17)
Rb2^{5}	S3	O31	Rb1	-172.87(8)	O11	S1	O13	Rb1	69.3(3)
Rb2^{5}	S3	O31	Rb2^{7}	20.7(4)	O11	S1	C1	S3	-164.2(2)
Rb2^{5}	S3	O32	Rb3^{6}	-94.2(2)	O11	S1	C1	S2	67.4(3)
Rb2^{5}	S3	O32	Rb2	84.6(2)	O13	S1	O12	Rb1^{2}	-137.75(16)
Rb2^{5}	S3	O33	Ag1^{7}	126.1(2)	O13	S1	O12	Rb1^{1}	-28.7(2)
Rb2^{5}	S3	O33	Rb3	-123.6(4)	O13	S1	O12	Rb2	98.9(3)
Rb2^{5}	S3	C1	S1	-119.9(2)	O13	S1	O11	Rb1^{2}	136.70(16)
Rb2^{5}	S3	C1	S2	8.5(3)	O13	S1	O11	Rb1^{3}	-116.1(2)
Rb2	S2	O21	Rb1^{2}	-28.7(2)	O13	S1	C1	S3	-44.2(3)
Rb2	S2	O21	Rb1^{3}	-149.10(8)	O13	S1	C1	S2	-172.6(2)
Rb2^{4}	S2	O21	Rb1^{2}	-166.9(3)	C1	S1	O12	Rb1^{1}	-145.23(17)
Rb2^{4}	S2	O21	Rb1^{3}	72.70(8)	C1	S1	O12	Rb1^{2}	105.77(18)
Rb2	S2	O22	Rb1^{3}	-179.6(3)	C1	S1	O12	Rb2	-17.6(3)

Rb2^{4}	S2	O22	Rb1^{3}	-82.88(17)	C1	S1	O11	Rb1^{3}	-1.0(3)
Rb2^{4}	S2	O22	Rb3	136.8(4)	C1	S1	O11	Rb1^{2}	-108.17(17)
Rb2	S2	O22	Rb3	40.1(5)	C1	S1	O13	Rb1	-43.9(3)
Rb2	S2	O22	Rb2^{4}	-96.7(3)	C1	S1	O13	Rb1^{1}	141.28(16)
Rb2	S2	O23	Ag1	-110.4(3)	C1	S3	O31	Rb1	-5.0(3)
Rb2^{4}	S2	O23	Ag1	36.3(2)	C1	S3	O31	Rb2^{7}	-171.5(2)
Rb2^{4}	S2	O23	Rb2^{5}	-90.9(2)	C1	S3	O32	Rb3^{6}	161.7(2)
Rb2	S2	O23	Rb2^{5}	122.4(3)	C1	S3	O32	Rb2^{5}	-104.06(17)
Rb2^{4}	S2	O23	Rb2	146.74(12)	C1	S3	O32	Rb2	-19.4(3)
Rb2	S2	C1	S1	61.6(2)	C1	S3	O33	Ag1^{7}	-129.36(19)
Rb2^{4}	S2	C1	S1	-123.2(2)	C1	S3	O33	Rb3	-19.1(4)
Rb2^{4}	S2	C1	S3	107.7(2)	C1	S3	O33	Rb2^{5}	104.53(18)
Rb2	S2	C1	S3	-67.5(2)	C1	S2	O21	Rb1^{3}	-66.28(19)
O12	S1	O11	Rb1^{3}	113.7(2)	C1	S2	O21	Rb1^{2}	54.1(3)
O12	S1	O11	Rb1^{2}	6.51(18)	C1	S2	O22	Rb1^{3}	74.93(16)
O12	S1	O13	Rb1^{1}	24.95(17)	C1	S2	O22	Rb3	-65.4(3)
O12	S1	O13	Rb1	-160.24(18)	C1	S2	O22	Rb2^{4}	157.8(2)
O12	S1	C1	S3	76.3(3)	C1	S2	O23	Ag1	-169.14(19)
O12	S1	C1	S2	-52.1(3)	C1	S2	O23	Rb2^{5}	63.7(3)
O31	S3	O32	Rb3^{6}	44.2(3)	C1	S2	O23	Rb2	-58.7(2)

^{1}1/2+X,1/2-Y,3/4-Z; ^{2}+X,1+Y,+Z; ^{3}-1/2+X,1/2-Y,3/4-Z; ^{4}-1+X,+Y,+Z; ^{5}1-Y,1-X,1/2-Z; ^{6}1+X,+Y,+Z; ^{7}+X,-1+Y,+Z

Table S112: Hydrogen Atom Coordinates (Å×10^{4}) and Isotropic Displacement Parameters (Å^{2}×10^{3}) for $Rb_5Ag[HC(SO_3)_3]_2(H_2O)_2$.

Atom	*x*	*y*	*z*	U(eq)
H1	500(70)	3350(70)	3311(10)	7(13)
H1A	7130(90)	10970(90)	3201(15)	40(20)
H1B	8520(180)	9980(190)	3170(30)	220(80)

E. Spectroscopic investigations

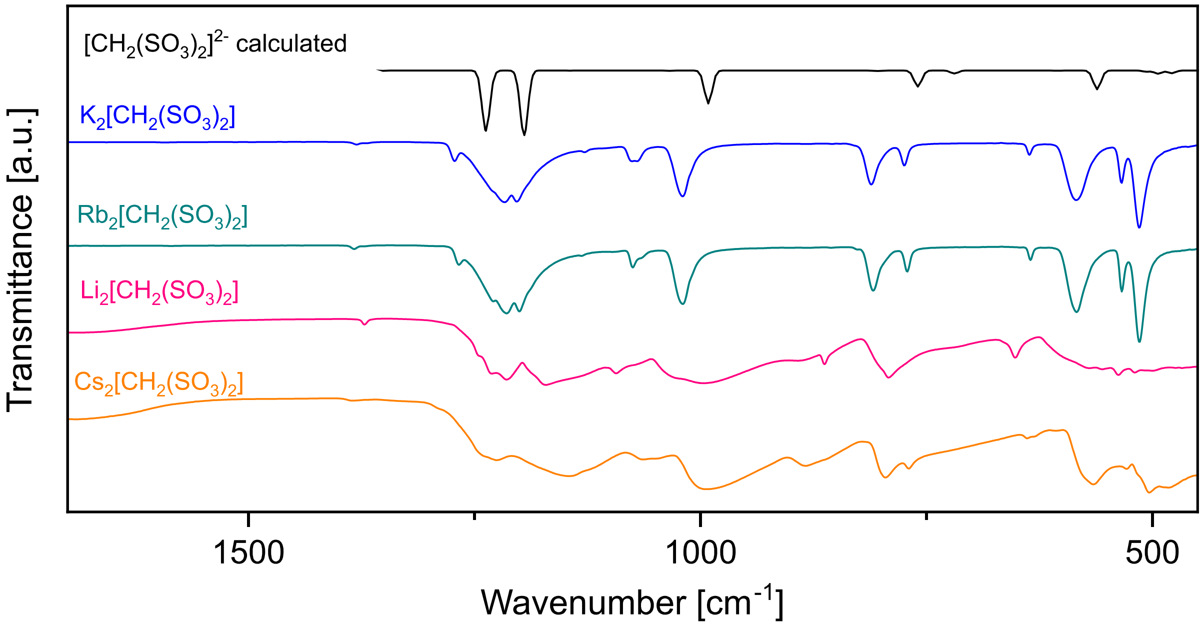

Figure S 28: Measured IR-spectra of KMDS, RbMDS, LiMDS, and CsMDS with calculated data for the Anion at the PBE0-cc-pVTZ level of theory in black at the top. Spectra are normed and an offset is applied for clearer portrayal.

References

[1] G. Sheldrick, *Acta Crystallographica Section A: Foundations of Crystallography* **2015**, *71*, 3-8.

[2] O. V. Dolomanov, L. J. Bourhis, R. J. Gildea, J. A. K. Howard, H. Puschmann, *Journal of Applied Crystallography* **2009**, *42*, 339-341.

[3] F. Kleemiss, O. V. Dolomanov, M. Bodensteiner, N. Peyerimhoff, L. Midgley, L. J. Bourhis, A. Genoni, L. A. Malaspina, D. Jayatilaka, J. L. Spencer, F. White, B. Grundkötter-Stock, S. Steinhauer, D. Lentz, H. Puschmann, S. Grabowsky, *Chemical Science* **2021**, *12*, 1675-1692.
